# Assessing the usability of Accessercise to increase physical activity in adults with physical disabilities: A qualitative think-aloud study

**DOI:** 10.1371/journal.pone.0321109

**Published:** 2025-04-01

**Authors:** James A. Haley, Daniel J. A. Rhind, David W. Maidment

**Affiliations:** School of Sport, Exercise and Health Sciences, Loughborough University, Loughborough, United Kingdom; Muhimbili National Hospital, TANZANIA, UNITED REPUBLIC OF

## Abstract

**Background:**

Mobile health (mHealth) applications (apps) offer a convenient way to increase physical activity to people with disabilities. While several mHealth apps have been developed for this population, there is limited evidence assessing app usability and how this might impact physical activity.

**Objective:**

To investigate the usability of a novel mHealth app, Accessercise, that aims to increase physical activity in people with physical disabilities.

**Methods:**

Twelve adults with different physical disabilities participated in a face-to-face think-aloud interview. Interviews were analysed using deductive content analysis based on the User Version of the Mobile Application Rating Scale (uMARS).

**Findings:**

Data mapped onto 12 uMARS domains; most participants expressed positive views concerning Accessercise, namely, entertainment, customisation, tailoring to the target group, ease of use and navigation, and visual information. Some additional elements were viewed positively but required modification to improve usability, perceived credibility, and relevance, including the app’s layout, visual appeal, interactivity, and quality/quantity of information.

**Conclusion:**

This study provides an example of how the think-aloud method can be employed to evaluate mHealth apps that aims to increase physical activity in people with physical disabilities. Therefore, developers and researchers could use this study to inform future usability evaluations in this area.

## Introduction

Adults with physical disabilities, defined as a loss of physical function or mobility that restrict important life activities [1], experience substantial barriers to physical activity [2]. These barriers include those related to the individual (e.g., negative attitudes/beliefs, employment status) and/or environment (e.g., transportation, inaccessible facilities) [3,4]. Moreover, during the COVID-19 pandemic, national lockdown restrictions added further barriers to participating in physical activities for this population [5]. For example, it was challenging to access specific adapted equipment and it was more difficult for individuals to obtain professional guidance on how to exercise safely at home [6]. As a result, these barriers prevent adults with physical disabilities from participating in physical activity, which in turn, can increase their risk of developing non-communicable diseases, as well as increase anxiety and depression [7]. Therefore, physical activity interventions that effectively address the barriers experienced by adults with disabilities are needed as a priority [8].

One potential solution to the aforementioned barriers is health-based interventions delivered through applications (or apps) on mobile and wireless devices, such as smartphones, tablets or computers. This can be known as mobile-enabled healthcare (mHealth), defined as medical and public health support to healthcare consumers via mobile-based devices (e.g., phones, tablets) [9]. MHealth apps may present a convenient way to deliver physical activity to people with disabilities, mainly due to the sparse and geographically distant number of individuals with disabilities who experience mobility difficulties, which can make the delivery of in-person interventions difficult. MHealth apps could relieve transportation and environmental barriers to physical activity, which are significant for people with physical disabilities [4]. Additionally, use of a mHealth apps by people with disabilities can also alleviate social and health inequalities [10].

MHealth apps are cost-effective, easily accessible at any time, and allow users to personalise their experience [11]. Further, users are not required to consult a personal trainer in-person [12]. Among adults without disabilities, mHealth apps have been reported to be efficacious at increasing physical activity behaviours with small-to-moderate effects [13]. These results may indicate the potential value of using mHealth formatsto deliver physical activity interventions to individuals with physical disabilities. However, people with disabilities have not typically been a primary target for mHealth apps, with very few mHealth apps being designed specifically with (i.e., co-produced) and for people with physical disabilities [14]. This may explain, in part, why there are increasing health care service disparities between people with disabilities and the general population [15].

Some of the existing evidence-based physical activity mHealth apps for adults with physical disabilities include WHEELS [16], ParaSportAPP [17], Fisiofriend [18], SCI Step Together [19], and 9zest Parkinson’s Therapy [20]. However, most existing interventions target a specific disability (e.g., amputee, spinal cord injury). For example, WHEELS [16] is aimed at wheelchair users with SCI or lower-limb amputation, while the 9zest Parkinson’s Therapy app was created to promote physical activity in people with Parkinson’s disease. Few mHealth apps focus on multiple physical disabilities. This could restrict access to physical activity mHealth apps for people with different physical disabilities, potentially increasing sedentary behaviours and reducing physical activity participation. More recently, the Accessercise app has been developed for adults with different physical disabilities (e.g., achondroplasia, amputee). Features of the app include building your own custom workout from a library of exercises according to the user’s physical disabilities, a social hub to interact with other users, and a directory of gyms and fitness facilities based in the United Kingdom (UK) ranked by users for accessibility. A detailed description of the Accessercise app has previously been reported [21]. The focus of Accessercise is to help people with disabilities get fit, strong, and healthy, providing users with several primary functions. First, the Accessercise app features a video library tailored to participants’ needs and disabilities to help illustrate suitable exercises that are demonstrated by a role model with the same impairment. Second, the app offers monitoring function where users can log workouts, track progress, and meet or exceed their goals. Third, an explore section exists where users can search a directory of UK fitness facilities ranked for accessibility. Fourth, a social hub, where users can connect with others, share their progress with followers and groups, and be part of a diverse, supportive, and passionate community.

To ensure new mHealth apps, including, Accessercise, operate as intended, a critical first step is establishing their usability [22]. Usability relates to the formal assessment of the extent to which interaction with a product or system (i.e., mHealth app) is effective, efficient, and viewed as satisfactory by the user [23]. Usability testing aims to detect possible issues when using an app, leading to further developments to improve its functionality and increase the possibility of technology acceptance among end-users [24]. Nevertheless, poor usability is often hindered by hardware issues, such as small screens and limited input opportunities [25], which can lead to low adherence or app rejection [26]. If an end-user does stop using a mHealth app, certain health parameters are no longer controlled for and the app will no longer “facilitate the target behaviour” [27]. Therefore, undertaking usability testing is imperative in designing and (re)developing mHealth apps [28,29]. However, the usability of mHealth apps has received limited attention in the context of increasing physical activity in people with physical disabilities [30]. In the literature, only several mHealth apps for people with disabilities have been assessed for usability, including WHEELS [16], ParaSportAPP [17] and 9zest Parkinson’s Therapy [20]. Overall, these studies reported positive satisfaction with the apps’ usability, potentially leading to increased adherence, accessibility, and effectiveness in improving physical activity in these populations.

To uncover usability issues in mHealth apps, a highly effective method is to use/employ questionnaires, interviews, focus groups, think-aloud methods, and eye tracking [31]. Among these models, the think-aloud method has been widely used for identifying usability problems in digital health tools and is commonly used for evaluating health behaviour change interventions [32,33]. This method requires asking users to continuously verbalise their thoughts and feelings when performing a given task (e.g., navigating through an app) [34]. The advantages of think-aloud interviews are that participants’ thoughts and needs can be elicited during testing, and the ongoing verbalised information collected allows the researcher to better identify the source of possible problems [31]. While methods such as questionnaires and surveys are easy to use, cost-effective and can be used on a large sample, such methods cannot provide specific information on usability issues that need to be addressed and rely on user perception [35].

Unlike other usability testing methods (e.g., observations, questionnaires), think-aloud helps understand how users think about the problem independently and provides a clear picture of the reasoning process in real-time [36]. As a result, think-aloud can help discover usability issues and identify improvements, providing direct information on a user’s thinking and cognitive processes as they happen [37]. The think-aloud approach helps overcome memory/recall bias in methods like questionnaires and interviews by collecting real-time data, preventing data loss that can occur when data is collected retrospectively. [38,39]. Think-aloud interviews also reveal the mental steps users take in real-time, helping designers make informed decisions to improve usability, functionality, and user satisfaction. Additionally, the think-aloud method performed individually offers clear, authentic insights into each user’s experience, allowing them to express their thoughts without group influence or peer pressure, ensuring accurate and detailed data while reducing the risk of group bias. On this basis, the current study employed think-aloud interviews to examine the usability of the Accessercise app, specifically designed for people with different physical disabilities.

## Methods

The methods are reported in accordance with the Standards for Reporting Qualitative Research (SRQR) checklist [40].

### Qualitative approach and research paradigm

The current study aimed to gain a broad understanding of the usability of Accessercise. The research was grounded in the qualitative, relativist epistemology, whereby multiple realities are socially constructed, and meaning is created through the interaction between the researcher and participants [41].

### Researcher characteristics and reflexivity

Qualitative data collection and analysis were undertaken by the first author (JH), who has previous research and experience working with people with disabilities (i.e., physical and sensory) in a physical activity context. Reflexivity has become an integral component of qualitative research [42], recognising that the researcher’s unconscious biases, and conscious reactions and responses can impact on the conduction and interpretation of data [43]. Therefore, reflexivity was considered throughout the research process; hand-written field notes were made by JH in a reflexive diary, which included reflexive and subjective comments after each interview. JH debated and contested the interpretations and conclusions present in the data analysis and report these to all co-authors (DJAR, DWM – who were JH’s PhD supervisors and who both had expertise in qualitative methods, including think-aloud) acting as “critical friends” to maximise reflexivity [44].

### Context

One-to-one think-aloud interviews were undertaken between 1 February and 31 May 2023. Interviews were conducted at a mutually convenient time and in a private location within the School of Sport Exercise and Health Sciences at Loughborough University (LU) that was secluded, quiet, and free from distractions. All interviews were facilitated by JH to maintain consistency. The interviewer had no connection with the participants before the study, and no repeat interviews were undertaken with any participants.

### Sampling strategy and participants

Between December 2022 and January 2023, twelve participants were recruited through social media platforms (e.g., Facebook, X), word of mouth, and through contacting local sports clubs. The inclusion criteria were: (i) adults aged ≥ 18 years, (ii) living with a physical disability, (iii) a resident living in the UK, and (iv) had access to a compatible internet-enabled smartphone to use the Accessercise app. Individuals were recruited using a purposive sampling strategy [45]. The demographic information of the sample is presented in Table 1. This sample size was deemed sufficient, given that a sample size of 10 to 15 participants is deemed necessary to understand usability [46].

**Table 1:** Demographic characteristics of each participant who took part in the study.

Participant	Gender	Age	Ethnicity	Physical disability
P01	Female	24	White British	Spinal cord injury
P02	Male	44	White British	Sacral agenesis
P03	Female	42	White British	Spinal cord injury
P04	Male	38	White British	Spinal cord injury
P05	Male	54	White British	Cerebellum ataxia SCA 13
P06	Male	50	White British	Spinal cord injury
P07	Male	23	White British	Amputee
P08	Male	52	White British	Motor neuron disease
P09	Male	45	White British	Cauda equina syndrome
P10	Male	31	White British	Dwarfism
P11	Male	58	White Irish	Spinal cord injury
P12	Male	42	White British	Spinal cord injury & amputee

### Ethical issues pertaining to human subjects

Full ethical approval was granted by Loughborough University Ethics Review (Human Participants) Sub-Committee (Ref: 2022-8262-9840). Participants received an information sheet and provided written informed consent prior to taking part in the research. Data was stored on a password protected server with anonymity and confidentiality upheld by replacing participants’ names with numbers (e.g., participant P1, P2), as well as removing identifying data from the transcripts.

### Data collection methods

Data was collected using qualitative think-aloud interviews [34] to ascertain participants’ views on the usability of the Accessercise app. Think-aloud interviews allow participants to verbalise aloud in real-time everything they think about when completing a task [47]. Therefore, participants communicate their thoughts that would have otherwise remain silent [34]. One-to-one interviews, lasting between 42 and 113 minutes, were audio recorded. The data collect were stored on LU’s password-encrypted Microsoft OneDrive system that was only accessible by the research team.

### Data collection instruments and technologies

An interview guide was developed based on a discussion amongst all co-authors and a review of the literature (Supplemental Materials, S1 Table). The guide included broad, open-ended questions, allowing free expression to clarify the participants’ experiences. The interview guide was pilot tested on one participant to confirm that questions were naturally framed and to identify ambiguities or irrelevant questions. No amendments were needed, although some questions were rephrased to improve clarity. Once each interview was completed, participants were given time for open discussion. Furthermore, brief field notes were undertaken by the first author in a dedicated journal throughout each interview. The notes were later used to help contextualise the data generated during analysis. Data saturation was reached after 12 interviews, as no new themes emerged, and further data were unnecessary to answer the research question.

### Data processing

Audio recordings were transcribed verbatim within 24 hours of completion of the interview by JH. To confirm immersion and familiarisation with the data set, and to verify accuracy, each transcript was checked for accuracy against the audio recording by JH and was then forwarded to the interviewees for approval, further comments, and/or corrections. The transcripts were then printed, ready for hand-written analysis using highlighters to differentiate between codes (See Supplemental Materials, S2 Table). Despite the possibility of overlooking or mis-categorising analysis by hand due to human bias, fatigue or subjective judgement [48], thematic sorting by hand was chosen because it allows researchers to engage directly with the data, ensuring that subtle or complex patterns are not overlooked [49]. Hand-analysis provides a deeper, more flexible understanding of the data, allowing the researcher full control over the identification and interpretation process [49].

### Data analysis

Deductive content analysis, which involves assigning the text to one or more domains, was employed to analyse the data [50]. Content analysis, which is one of the most used techniques to analyse qualitative data [51], was deemed most suitable due to its systematic and objective approach for identifying what content and information is contained in the discourse [52,53]. The analysis was completed in several phases. The first phase began by creating a formative categorisation matrix with four pre-set categories based on components within the User Version of the Mobile Application Rating Scale (uMARS) [54] to identify patterns of meaning in the data and support researcher understanding. The categorisation matrix is a technique used to organise and categorise data into specific themes. This process involved sorting individual quotes from the dataset into specific themes, with each passage of text being coded and assigned to one or more themes based on its content. The uMARS was selected as it provides simplified readability and a reduced number of items, which better allows mHealth evaluation by end users of differing education backgrounds, ensuring a fully inclusive analysis [55]. The uMARS is a simple and validated tool that can be used by end-users to assess the quality of an mHealth app across four objective quality subscales: engagement, functionality, aesthetics, and information quality [56].

In the second phase of the analysis, the JH immersed himself in the data by reading and then re-reading all the interview transcripts to maximise familiarisation and make sense of the data. Additionally, JH read through his reflexive diary on several occasions and met three times with the co-authors to critically review and discuss any reflexive field notes. In the third step, specific relevant passages of text were highlighted and then colour coded using highlighter pens. In the fourth phase, colour-coded passages were clustered, and a descriptive code was produced. In the fifth phase, any codes were grouped and categorised based on their meanings, similarities and differences and then aligned to the prearranged categorisations within different themes and second-order themes highlighting the reappeared basic thoughts typical of the participants descriptions. Lastly, a content analysis map was constructed to share a visual presentation of themes, codes, and their relationships, involving a detailed account and description of each quotation. The presentation of the findings is based on the four pre-determined themes based on the uMARS.

### Strategies to enhance trustworthiness

Trustworthiness is one of the essential components of ‘rigour’ in qualitative research [57]. Even though there is no universal definition, trustworthiness relates to the extent to which one can have confidence in the quality and appropriateness of the data and its interpretation [58]. It is the overall impression of quality associated with a research project [59]. Four criteria are widely used to appraise the trustworthiness of qualitative research: credibility, dependability, confirmability and transferability [60]. Nevertheless, this method has been critiqued in recent years, as it has been proposed to violate the philosophical underpinnings of qualitative research [44,58,61]. Researchers, including Burke [61] and Smith and McGannon [44] argue that the trustworthiness criteria are rooted in positivist paradigms, which conflict with the epistemological foundations of qualitative research that emphasises subjectivity, context, and interpretation. They contend that the criteria impose rigid standards that undermine the relational and dynamic nature of qualitative inquiry. In contrast, reflexivity is seen as a superior approach because it acknowledges the researcher’s subjectivity and the co-constructed nature of knowledge.

More recently, the phrase ‘reflexivity’ has been considered vital. Therefore, a “critical friend” technique was used to increase the rigour of the analytical process. The purpose of critical friends is ‘not to agree’ or ‘achieve consensus’ but rather to ‘encourage reflexivity’ [45]. This technique allows researchers to challenge one another’s construction of knowledge and interpretation, reducing research bias [62], which is a critical practice for ensuring qualitative goodness. All co-authors met three times to critically review and discuss the codes, sub-themes, and the hierarchy of the themes, alongside any reflexive field notes. This phase of analysis resulted in the development of several further codes and the merging of existing codes. Trustworthiness was further upheld by pilot testing the interview schedule to review its relevance and to remove ambiguity and irrelevant questions, with no amendments needed. In addition, each transcript and reflexive field notes were emailed to participants, with all participant’s being content, and no amendments were made to the final transcripts.

## Findings and discussion

Four higher-order-themes (derived from the uMARS), each with several second-order-themes (generated by the research team) corresponding to the usability of the Accessercise app were reported (Table 2): (i) Engagement (e.g., entertainment, customisation), (ii) Functionality (e.g., ease of use), (iii) Aesthetics (e.g., layout), and (iv) Information (e.g., quantity and quality of information). Each higher-order theme and associated second-order themes with illustrative quotes are presented. Additional quotes are presented in the Supplemental Materials S3 Table. Significance was established through a comparative analysis with existing mHealth literature for people with disabilities. Using the uMARS scale, the study identified app features that enhanced physical activity, extending research on accessibility and user-centered design.

**Table 2:** Higher- and second-order themes from the Content Analysis of interview data.

Higher-order theme	Second-order theme
Engagement	Entertainment Customisation Interactivity Target group
Functionality	Performance Ease of Use Navigation
Aesthetics	Layout Visual appeal
Information	Quality of information Quantity of information Visual information

### Engagement

#### Entertainment.

Entertainment was identified as a strength of Accessercise. For example, P09 (male, cauda equina syndrome) commented, *“Nutrition, blogs, shops and podcasts are all amazing features on the app.”* Generally, podcasts are becoming increasingly popular to gain health information [63]. Regular engagement with podcasts can lead to listeners forming a relationship with the host, leading to a level of trust [64]. As such, integrating entertainment into apps may enhance user trust, ultimately promoting sustained engagement for people with disabilities.

However, other participants shared disinterest in such entertainment features. One participant explained, *“I never look really at blogs and podcasts. I’m not really into them. I’m not that interested”* (P03; female, spinal cord injury). This finding could be due to people with disabilities using smartphone apps and the internet much less than the general population [65]. Such a disparity could arise from the expenses associated with smartphones, higher living costs and inherent physical difficulties, such as manual dexterity [66,67]. Therefore, people with disabilities may be reluctant to use interactive features on mHealth apps, which may result in decreased use.

#### Customisation.

The option to customise workouts was valued across participants, with one participant, P01 (female, spinal cord injury) stating, *“I appreciate that you can set a goal and it’s not just about increasing muscle or losing weight”* and *“I really like that you can choose where you complete your workouts”*. This finding is significant given that limited apps include customisable exercise content [68]. Customisation enables users to modify their technology, resulting in a personalised user experience [69]. The ability to exercise at home is valuable, given time and accessibility are common barriers to physical activity for people with disabilities [4]. However, users are only half as likely to engage with mHealth fitness apps with limited customisation [70]. Therefore, to enhance user engagement, app developers should provide goal customisation options, empowering users to personalise content according to their individual needs, which will likely increase user engagement.

Participants also appreciated options for adapted equipment, with one participant stating, *“The ability to customise and choose adapted equipment for your workouts is an amazing idea”* (P04; male, spinal cord injury). This finding is significant, considering that inadequate equipment is a prevalent obstacle to physical activity for individuals with disabilities [71]. Users exercising at home find value in the ability to choose adapted equipment, which may not be readily available in traditional gyms or may lack accommodations for specific needs [72]. As a result, adaptive equipment options may enable users to engage in environments where equipment access is limited, ultimately increasing physical activity participation.

#### Interactivity.

Several participants argued that Accessercise lacked any form of feedback. For instance, one participant stated, *“I think Accessercise is missing logistics data. I want to review my progress”* (P07; male, amputee). To overcome this, one participant stressed, *“Yeah, monitoring progression through graphs and statistics would be good”* (P09; male, cauda equina syndrome). Users believe feedback can encourage them to undertake more physical activity [73]. Feedback on users’ behaviour is crucial, as it allows them to align progress with their goals [74,75]. Therefore, implementing feedback on mHealth apps can foster progress towards goals, enhancing self-efficacy [76], and potentially encouraging further uptake/adherence in people with physical disabilities.

Most participants liked the option to share workouts on Accessercise. One participant mentioned, *“I like the fact that you can share your goals or ideas it’s bit like you know like the Strava fitness app”* (P01; female, spinal cord injury). It is essential to create social connections in mHealth apps, as this is likely to encourage participation in an activity [77,78]. This may be particularly important for individuals with disabilities, given they commonly experience loneliness, low perceived social support, and social isolation [79]. Consequently, by integrating social features including peer support and mentorship and real-time interaction (e.g., live chats/video calls) that foster connections in physical activity, mHealth apps for people with disabilities may enhance psychological wellbeing.

Notifications were appreciated by most participants, with one participant (P09; male, cauda equina syndrome) commenting, *“Notifications or nudges would really help me undertake more exercise.”* Despite too frequent notifications being annoying to some users and potentially discouraging them from using apps [80], push notifications have shown promise for motivating initial enrolment in physical activity interventions [81] and evoking repeated invention use [82]. Utilising push notifications enable users to easily self-monitor their behaviour to achieve their intended goals [83]. As a result, mHealth app developers could include notifications, as these may enhance uptake, use, and adherence apps, thereby facilitating increases in physical activity.

#### Target group.

The language used within Accessercise was discussed regularly by participants. Some of the key terms included ‘stretching’, ‘building muscle,’ and ‘increasing strength’ when selecting a workout goal. For example, one participant remarked, *“The terminology they have used for each goal is good”* (P10; male, achondroplasia). Consistency and clarity in terminology are crucial for an app’s success [84]. Plain language improves user experiences, making apps more functional by providing action-oriented information devoid of technical jargon [85]. Thus, when developing mHealth apps, developers should consider aligning the language in the app with everyday user language to maximise content understanding, facilitating take-up and adherence.

Selecting a disability type was highly valued, especially after one participant remarked, *“I like the fact that the app is adapted for people with disabilities”* (P01; female, spinal cord injury). This finding is significant given that mHealth apps rarely focus on personalisation [86]. Personalisation and tailoring may increase user engagement and adherence to mHealth apps [87]. Therefore, mHealth apps that allow personalisation may foster long-term engagement among people with disabilities as they meet end-users needs.

In addition, participants praised the map function as it allowed them to search for facilities that are accessible using their location (i.e., post/ZIP code). Users could also add accessibility rankings (from 1 [low accessibility to 5 stars [high accessibility]) for gyms and leisure centres in the UK. Specifically, one participant who noted, *“I like how you can find local gyms with the explore section, that’s a really useful feature”* (P03; female, spinal cord injury). This finding is helpful, given that people with physical disabilities commonly report limited knowledge of where to exercise [71], potentially restricting opportunities to engage in physical activity. On this basis, mHealth apps should consider incorporating information to enable people with disabilities to overcome knowledge and environmental barriers to physical activity. This valuable option is likely to enhance users’ knowledge, fostering positive behaviour and adherence to mHealth apps.

### Functionality

#### Performance.

Most participants experienced technological challenges when using Accessercise due to ongoing developments. As one participant highlighted, *“Obviously it’s [blogs, achievements, podcasts, online shop] still coming soon, which is a weakness of the app”* (P09; male, cauda equina syndrome). Many mHealth apps are of low quality since they are designed by researchers with limited software development skills [88]. For this reason, mHealth apps can experience technical ‘bugs’, be underdeveloped, and improperly designed [89,90], with these technical challenges negatively impacting take-up and adherence [91]. Therefore, people with physical disabilities may be unlikely to adhere to mHealth apps if their development is still ongoing or poorly designed.

Additionally, one participant expressed hesitancy to purchase the premium paid-for version of the app after claiming*, “I do think sixty pounds for the membership on the Accessercise app is quite expensive”* (P03). This finding is not surprising given that pricing and costs associated with using apps are a noteworthy issue that impacts their adoption [92]. Therefore, mHealth apps with high subscription costs may result in low adoption rates among people with physical disabilities, especially as this population can have low incomes and are often dependent on state benefits. App developers should design flexible and affordable membership plans, including necessary fee waivers, to assist individuals facing financial obstacles [93].

#### Ease of use.

Participants highlighted that the user-friendliness of Accessercise played a pivotal role in its perceived ease of use. For instance, one participant stated, *“It’s all clear and easy to pick this app up and use it even if you’re a beginner”* (P11; male, spinal cord injury). Easy-to-use and convenient mHealth apps that align with users’ desired learning style can contribute to higher levels of user engagement [94]. Furthermore, participants valued the videos provided for each exercise, which may help people with disabilities overcome the barrier of having limited knowledge of physical activity [95]. To demonstrate, one participant remarked, *“I also really like the videos because they show you how to actually do the exercise because some people don’t know how to do it properly”* (P02; male, sacral agenesis). Critically, videos can aid individuals with limited education, literacy, or enthusiasm, as they involve less reading, scrolling, or swiping through written content [96].

#### Navigation.

The ability to navigate smoothly through the app was perceived to be helpful by most participants. For example, one participant (P07; male, amputee) highlighted, *“I really like the Accessercise app. I think it will be really easy to follow when you are starting the session.”* Participants also found the four features at the bottom of the home screen (i.e., social, exercises, explore, and more) particularly helpful for navigation. To illustrate, one participant (P07; male, amputee) said, “*It’s quite easy to navigate mainly with the four tabs down the bottom of the screen.”* To maintain user engagement, it’s important that the interface in mHealth apps is easy to navigate [97]. Poor design features, including complex navigation, are poorly accepted by users in real-world settings [98]. Apps that provide simple and logical navigation between screens can encourage continued use [99]. Therefore, for widespread use among people with physical disabilities, mHealth apps should adopt simple navigation strategies, enabling successful user engagement and promoting adherence.

### Aesthetics

#### Layout.

Participants generally reported mixed feelings regarding the layout of Accessercise with several positives and negatives presented. Most participants appreciated the simplistic layout that Accessercise provided. For example, one participant commented, *“The layout of the app is good they [Accessercise developers] have done a good job!”* (P03; female, spinal cord injury). A possible explanation is due to a core group of users being involved in the app development. Apps that are user-centred in design are recommended in the development of mHealth interventions, ensuring that they meet the needs of the end user, resulting in improved usability and lower risk of failure, thus enhancing optimal uptake and adherence among end-users [100].

However, some participants reported concerns with the layout that required improvements. For instance, one participant (P12; male, spinal cord injury & amputee) said, *“You want to make an app as simple as possible to use at the moment the layout is a bit dull and it involves a lot of scrolling.”* A consistent layout can support users to manoeuvre around the app without getting confused [85]. To overcome this, one participant (P07; male, amputee) recommended presenting the content in alphabetical order to make it easier for users to identify content more quickly, with another (P12; male, spinal cord injury & amputee) suggesting the use of bullet points as well as numbered guides to improve layout *“rather than just a block of text”*. Overall, app developers should strive to implement solutions (e.g., numbered guides) that are universally designed, accessible, and usable for everyone, including people with physical disabilities [101], which may enable users to locate content quickly without confusion and difficulties.

#### Visual appeal.

Several participants reported positive first impressions of Accessercise, finding it visually appealing. For example, one participant said, *“It comes across pleasant straight away and compared to the other fitness apps there are only four options at the bottom and it’s straight forward and clean”* (P04; male, spinal cord injury). This finding is crucial as the design of apps, such as visual displays, can significantly impact users’ experience and their willingness to engage [102], as well as their trust in the content [103]. Consequently, an appealing visual design of an mHealth app will likely foster long-term user engagement.

However, some participants disliked the colour scheme, describing it as *“bland”* (P12; male, spinal cord injury & amputee), or *“excessively monotone”* (P01; female, spinal cord injury). Use of colour in an efficient manner is important to maintain user engagement, making mHealth apps more visually appealing and easier to use [104]. According to Holzinger and Errath [105], there should be a consistent colour pattern within the whole app. To address this, one participant suggested, *“an area for development for the Accessercise app is having the option to change colours on the app”* (P02; male, sacral agenesis).

On this basis, it is apparent that app interfaces should be customised to users’ preferences, such as high-contrast colours [106]. Colour preferences can change as people get older due to colour vision, contrast sensitivity, and visual acuity all declining with age [107]. Thus, for older individuals or those with sight impairments, colour contrasts on mHealth apps should be carefully considered to improve accessibility and acceptance. Incorporating varied colour contrasts is essential for ensuring successful engagement and reliability.

### Information

#### Quality of information.

Participants shared appreciation for the quality of information on Accessercise. One participant expressed satisfaction regarding one section, stating, *“The information about the opening hours and contact details [of local gyms/leisure facilities] is good”* (P03; female, spinal cord injury). However, other participants felt the usefulness of this information was somewhat limited. For example, one participant remarked, *“Yes, a phone number and opening hours are good, but it’s missing information [e.g., reviews on accessibility, cleanliness and tidiness]”* (P10; male, achondroplasia). Limited information can hinder people with physical disabilities from obtaining the necessary details for engaging with and adhering to mHealth apps, which may result in low up-take rates [99].

#### Quantity of information.

Several participants appreciated the quantity of information provided on Accessercise, with one participant remarking, *“The fact the Accessercise app has basic information and the pages are short, that’s good.”* However, some users found the quantity of information excessive, a weakness needing improvement, *“There is too much information per exercise with the big block of text”* (P10; male, achondroplasia). To address this issue, one participant proposed recommendations to improve the layout of Accessercise after noting, *“lastly on that filter section just like removing the muscle group section if it’s not like you’re doing flexibility or stretching or something like that”* (P01; female, spinal cord injury).

Overall, a good quantity of information is imperative for users of mHealth apps to find reliable, accurate, and timely information with minimal effort [108]. Excess information can overwhelm users, leading to cognitive load and potential disinterest in the apps. For example, as smartphones apps contain a lot of redundant material, searching for information can often take time and effort, resulting in user tiredness due to cognitive overload [109]. Therefore, to optimise user engagement with mHealth apps, information could be presented clearly and concisely in accessible terms [110].

#### Visual information.

The visual information on Accessercise played a vital role in its perceived appearance; participants emphasised the significance of appealing images, *“The images they have used on the app is good”* (P04; male, spinal cord injury). Notably, participants shared an interest in images depicting muscles targeted during exercises and the logos used throughout Accessercise, with two participants affirming, *“I like the pictures of the muscle components being worked”* (P03; female, spinal cord injury), and *“The little logo they have used next to each goal is creative and helpful”* (P07; male, amputee). In summary, these findings underscore the significance of integrating visually appealing information on mHealth apps for individuals with physical disabilities, as it is well-received by this population. Thus, mHealth physical activity apps could persist in incorporating impactful logos and images to enhance uptake, use, and adherence among users.

### Final reflections

Our study is one of the first to understand the perception of end-users with physical disabilities using think-aloud to explore the usability of a novel physical activity mHealth app, Accessercise. This study demonstrated that the Accessercise app provides a simple platform that, despite several troubleshooting/performance challenges, can be efficiently operated by various users with different physical disabilities. People with physical disabilities are expected to overcome multiple barriers (e.g., cost, time, lack of adapted equipment) to engage in regular physical activities. However, our findings indicate that Accessercise could be an effective intervention to help overcome some of these previously identified barriers. For example, the app offers high-quality exercise videos that are customised for people with varying physical disabilities using a range of easily accessible equipment (e.g., water bottle, rucksack), which may act as facilitators in the long-term to encourage regular physical activity among this population.

Therefore, these findings have helped to further our understanding of how a mHealth physical activity app can be designed and configured to meet the needs of people with physical disabilities. Such findings could help app developers reconsider the ways in which apps are currently designed based on user’s preferences, which might, in turn, increase the uptake and adherence and, potentially, result in successfully increasing their participation in physical activity.

### Study limitations and future research

First, the sample population were predominantly White British, English-speaking, male, all under the age of 58 years, and had sufficient internet skills, which limits the generalisability of experiences identified to other people with physical disabilities. To ensure that Accessercise is accessible to a diverse range of users, future research that involves a more heterogenous sample is warranted. Second, participants completed a think-aloud interview under the observation of a researcher in an unfamiliar setting (i.e., a university laboratory), which is not analogous to how they would engage with the app in real-world settings (e.g., home, gym). Consequently, valuable data on the app’s performance in a more ecologically valid environment could have been overlooked. For future studies, researchers may want to supplement think-aloud interviews with extensive field testing to address such challenges.

Third, the controlled laboratory setting and audio-voice recording interviews may have influenced participants’ responses, potentially leading to overly positive feedback and reduced willingness to criticise the Accessercise app. Despite building rapport with participants, informing them they could be open and honest and that the researchers were independent from the app, such experiences may have induced or increased anxiety and impacted the participants’ ability to use the app efficiently. Future studies must consider the setting where usability tests occur more cautiously; the physical environment should be the least stressful. Fourth, some app functionalities (such as blogs, podcasts, and nutrition) were under development during the interview phase and therefore were not provided to the participants regardless of the target group’s interest, which could have affected the user’s satisfaction with the Accessercise app. Future studies should consider incorporating such functions to gather rich data on user intention and acceptance of these and whether they will support usability from its end-users.

Fifth, this study is susceptible to confirmation bias, as the researchers may have favoured data supporting pre-existing assumptions about the mHealth app’s effectiveness, while underreporting or overlooking critical usability issues. Future studies could mitigate confirmation bias by engaging a diverse group of researchers external to the primary research team when analysing the data, ensuring that both positive and negative feedback are equally weighted. Sixth, JH conducted the testing and independently analysed the data, which increased the risk of observational bias. Both roles may have influenced the interpretation of participants behaviours and feedback, introducing a subjective lens that may have impacted the objectivity and reliability of the conclusions drawn. Future studies could benefit from involving multiple researchers in both data collection and analysis to reduce potential bias and enhance objectivity.

## Conclusion

This qualitative think-aloud study offers a unique contribution to the literature, as it adds to the limited number of usability studies evaluating mHealth apps in people with physical disabilities [14,30]. These studies are highly valued and needed to understand the utility of such interventions [111]. Specifically, this usability evaluation of Accessercise, using a think-aloud method, has revealed high acceptance and the potential to provide people with physical disabilities with an easy-to-use, accessible intervention to increase physical activity. Characteristics that promoted usability included ease of navigation, good aesthetics (e.g., layout, colour), high-quality information, and customisable features (e.g., selecting an impairment, tailored workout videos, and workout locations). Consequently, given limited usability studies in this area, we have shown that this method is acceptable and can be informative as we have provided an evidence-based protocol that other researchers and app developers could use to inform their usability evaluations. Future developers of physical activity interventions aimed at this population should also consider leveraging the insights acknowledged here to maximise their usability, improving user acceptance, as well as increasing health behaviour change, namely, physical activity participation. This could also have further impacts, such as reducing the risk of developing associated conditions, including diabetes, obesity and cardiovascular disease.

## Supporting information

S1 Table Interview schedule. (DOCX)

S2 Table Transcripts of interviews undertaken with participants 1-12. (DOCX)

S3 Table Content analysis map. (DOCX)
