# Supplementary material for: Assessing the usability of Accessercise to increase physical activity in adults with physical disabilities: A qualitative think-aloud study
Source: PLoS One. 2025 Apr 1;20(4):e0321109. doi: 10.1371/journal.pone.0321109 (PMC11960980; doi:10.1371/journal.pone.0321109)
Supplement: S1 Table — (DOCX) [file pone.0321109.s001.docx]

**SUPPLEMENTARY MATERIALS**

Contents

[**Table S1.** 2](#_Toc192049193)

[Interview Schedule 2](#_Toc192049194)

# **Table S1.**

## Interview Schedule

| Interview Overview |
| --- |
| - Who I am (James Haley) and what my role is within this research project (Lead Researcher). - The aims, importance, and purpose of undertaking this research project and the benefits of participating. - What University do I attend (Loughborough), what is my position (Doctoral Researcher), faculty that I am involved in (School of Sport, Exercise and Health Sciences) and others working on the project (Sam Breary; Director of Accessercise, Ali Jawad; Co-Director of Accessercise, David Maidment; Supervisor and Daniel Rhind; Supervisor). - Briefly outline all the ethical considerations in this study (e.g., consent forms, information sheet, confidentiality, anonymity, withdrawing and participants’ rights within this project). - There are no right or wrong answers in this study, do you have any questions you would like to ask before we continue with the interview process? |
| Introductory Questions |
| - Please may you provide the following information: name, age, gender identity, ethnicity, county of residence? - To start, could you tell me how you first got involved in the ‘Accessercise’ application and why? - Could you please tell me your experience of using the ‘Accessercise’ app and how long you have been a member? |
| Accessercise Specific Questions |
| - Do you currently use Accessercise in your role? (If so, when? how?) - What were your thoughts when you first started using the Accessercise application? - Do you have the required equipment to use Accessercise in its full capacity (i.e., access to a mobile phone to use the application)? - Are there any improvements that you believe could help improve the Accessercise application? - Do you use any other similar applications outside of Accessercise (If so, what? Better/worse than Accessercise?) |
| Think Aloud Protocol |
| We are now going to move on to the main part of this study and ask you to participate in a Think Aloud Protocol whilst using the Accessercise application.  The instructions for a Think Aloud protocol are straightforward – I’d like you to say out loud whatever comes into your mind. There are no right or wrong answers. We’ll just run through a quick practice to get you used to the protocol. Could I ask you to say out loud everything that comes to your mind when you think about physical activity?  *Questions?*  I’d like you to share your screen and open the Accessercise application.   - What are your first impressions of the application?   I’d now like you to use the application as you would normally use it. Please continue to speak your thoughts as you move through the app. Please show me all the features that you use, and feel free to describe your actions, thoughts, and feelings as you do so.   - Have a look at the instructions that Accessercise have provided. Talk me through how you use these when navigating through the application. |
| Overall Thoughts |
| - What are the potential positives (if any) of using Accessercise for improving physical activity levels? - What are the potential negatives (if any) of using Accessercise for improving physical activity levels? - How do you think the Accessercise app could be improved (if anything) for its use in improving physical activity levels? |
| Final Questions |
| - Is there anything else that you would like to add/discuss? |
