# Supplementary material for: Assessing the usability of Accessercise to increase physical activity in adults with physical disabilities: A qualitative think-aloud study
Source: PLoS One. 2025 Apr 1;20(4):e0321109. doi: 10.1371/journal.pone.0321109 (PMC11960980; doi:10.1371/journal.pone.0321109)
Supplement: S2 Table — (DOCX) [file pone.0321109.s002.docx]

**SUPPLEMENTARY MATERIALS**

Contents

[**Table S2.** 2](#_Toc192050067)

[Transcript of interview undertaken with Participant 1 2](#_Toc192050068)

[Transcript of interview undertaken with Participant 2 30](#_Toc192050069)

[Transcript of interview undertaken with Participant 3 60](#_Toc192050070)

[Transcript of interview undertaken with Participant 4 105](#_Toc192050071)

[Transcript of interview undertaken with Participant 5 137](#_Toc192050072)

[Transcript of interview undertaken with Participant 6 154](#_Toc192050073)

[Transcript of interview undertaken with Participant 7 181](#_Toc192050074)

[Transcript of interview undertaken with Participant 8 217](#_Toc192050075)

[Transcript of interview undertaken with Participant 9 247](#_Toc192050076)

[Transcript of interview undertaken with Participant 10 282](#_Toc192050077)

[Transcript of interview undertaken with Participant 11 311](#_Toc192050078)

[Transcript of interview undertaken with Participant 12 333](#_Toc192050079)

# **Table S2.**

## Transcript of interview undertaken with Participant 1

**Key code:**

R: Researcher

P1: Participant 1

| R: So, thank you Abigail for taking part in this research so first of all I will go through the overview of the interview and I will go through who I am and the aims, importance and purpose of undertaking this research uhm the University I attend uhm who is in involved in the research project as well as any ethical considerations within this study uhm so first of all I am James Haley and my role within this research is that I’m the lead researcher uhm the aims, importance and purpose of undertaking this research is that during the COVID-19 pandemic uhm we noticed people with disabilities didn’t undertake much physical activity due to the lock down uhm we feel with the development and advancement of technology and apps we have noticed theirs a new app on the market that we can evaluate to see how good the app is at improving several outcomes regarding health uhm and we feel like this is a good opportunity to encourage and improve overall physical activity levels for people with disabilities uhm the benefits of participating is that you will be able to see how good the app is at improving your health uhm and hopefully over time it can encourage you to undertake more physical activity and feel more motivated to participate and the University that I attend is Loughborough University uhm I’m a Doctoral Researcher within the Peter Harrison Centre for Disability Sport within the School of Sport, Exercise and Health Sciences uhm I am currently being supervised by Drs David Maidment and Daniel Rhind uhm and we are also collaborating with the app developers called Ali Jawad and Sam Breary uhm all your data will be confidential and anyone outside of this research study will not be able to obtain any data uhm so you have read the information sheet and signed the consent forms which is perfect uhm if at any time you want to withdraw from the study and you do not want to participate please feel free to let me know that’s perfectly fine not a problem and finally there are no right or wrong answers within this study uhm do you have any questions that you would like to ask before we continue with the interview.  P1: No, I’m ok.  R: Perfect, awesome let’s start so the first section is the introductory question so the first question I have got is uhm please may you provide the following information so your name, age, gender identity, ethnicity, and county of residence.  P1: Gosh, uhm I have forgotten already.  R: Haha.  P1: Uhm, Abigail haha did you ask for my full name?  R: Yeah.  P1: Abigail uhm Abigail Bishop.  R: Your age?  P1: Twenty four.  R: Gender identity?  P1: Uhm, female uhm Northamptonshire.  R: And your ethnicity?  P1: British.  R: Perfect.  P1: White British.  R: White British yeah haha. Uhm, so to start could you tell me how you first got involved in the Accessercise application and why?  P1: Uhm haha.  R: So, how did you first get involved in this study and why?  P1: Uhm, ok. Uhm, so I had a diagnosis of cauda equina syndrome and I thought there was research into it and I thought that since having my injury it would be ugh good to get back into sport uhm but again uhm I know recognise that with some of my symptoms it’s not always easy as just going for a run especially uhm and I haven’t got it with me today but I normally use a walking stick most of the time and like that so uhm I guess it was interesting to see whether there was different ways I could go about getting back into routine exercise uhm without causing further injury I guess.  R: Perfect, ok and have you got any experience of using any fitness apps in the past before?  P1: Uhm, no other than pure gym and they had like a little uhm things on there but other than that no uhm not since my injury.  R: Ok and do you think that a fitness app would be something that would benefit you and would encourage you to workout at the gym or home and is something that you think you can use quite frequently.  P1: Uhm, yeah I think it would be interesting to uhm look into it today and trial the app and see whether it is something that would help me uhm I do like the location filters or the things where you know you can use adapted equipment like a tin of beans.  R: Oh, ok so you like the adapted equipment option?  P1: Yeah, I like the idea of that uhm that’s something that is really useful and the fact it seems uhm I don’t know what I’m trying to say haha.  R: Haha.  P1: And yeah, haha that’s all fine but yeah I like the fact that the app is adapted for people with disabilities if that makes sense and seems interesting for people to have specific focuses uhm especially uhm my physiotherapist will be very happy that there will be things like strength in there and building up strength which is important.  R: So, you mentioned that you liked the impairment filter section so what specifically do you like about that do you like the fact that it is very much tailored to the individual’s impairment and that the app is not very broad so it’s very much focused in on the individual?  P1: Yeah, I like the fact that you can filter everything down especially I guess your disability and the fact then again it does not necessarily mean that if you try something you have invested into a lot of equipment that again if it doesn’t quite work out uhm that is fine and you can use items around your house uhm and I like the fact that it knows that not everyone can do exercises standing up uhm especially for myself standing and sitting is quite difficult for example I can sit for fifteen minutes and then I will need to stand so the idea that it can be done in both as well and basic items that you can use around the house is definitely useful I guess.  R: Yeah, definitely because it is tackles some of the barriers in terms of cost and time of going to the gym and stuff like that uhm so that’s great. So, as mentioned earlier your experience of using Accessercise app uhm how long have you been a member for?  P1: Haha ten minutes.  R: Haha, ok and do you think Accessercise is something that you’ll likely to use long term over a long period of time do you think it is something that you’ll get into, and you’ll enjoy it?  P1: Yeah, I think uhm it is obviously something that you need to try out to find out different things that work for you uhm especially when you showed the different things like the blog uhm as well as the podcasts and nutrition uhm if they had those options come out soon that would be amazing uhm I think that would be really useful especially because I think health is something that is really important when you are experiencing an injury as well uhm like trying to maintain a good health is something that becomes more difficult I guess uhm so I really like the fact that you can tailor it for something like your home where you know you have accessibility to things like bathrooms when you need it and it’s just I don’t know I really like the fact that it makes it more specific to the person and therefore makes it more relaxed and less daunting I guess.  R: Yeah, that makes sense yeah. So, in terms of the shop what sort of things would you like to see pop up in the shop uhm would you like to see branded clothing of Accessercise or discounts in there when you have undertaken a certain number of exercises?  P1: Yeah, I think I think uhm yeah I guess that would be a good idea like if you have rewards like an incentive uhm because incentives always work uhm but just yeah that idea of hitting certain goals or meeting certain things would be good uhm it does not necessarily have to be monetarily discounts but like even if it was like here is a free protein shaker haha I don’t know for like doing your exercises haha not like everyone drinks protein shakes but if you did then yeah that would be a good reward because it would keep people motivated I guess.  R: So, you think having like an incentive in place for example if you do a certain amount of exercise on the app you get some sort of like uhm I don’t know like a discounted ticket to the cinema or something like that uhm you would like that?  P1: Oh yeah, people love the cinema haha absolutely uhm it is definitely a better reward than a protein shaker haha.  R: Haha so do you think that is something that they can add to the app so like the incentive options?  P1: Yeah, I think that would be really good yeah because everyone wants discounted cinema tickets or even if you could partner with food brands or something uhm that would be really interesting uhm you know when you have uhm I’m not sure if you have seen but the cards that you can get uhm I can’t remember what they are called haha uhm like taste cards.  R: Yeah, I know taste cards I have one!  P1: Yeah, so those sorts of schemes give you a certain amount of money off.  R: Exactly so for example you can get discounts on things like pizza express.  P1: Yeah or even like what Tesco’s do uhm like the vouchers uhm so if you collect a certain amount of points it turns the points into different vouchers for example if you collect fifty points it turns it into a pound or whatever.  R: That could be a really good idea because it’s always quite exciting when those vouchers appear through the post or on the app haha.  P1: Yeah, haha and you save up all your points so that you are able to get ten pound off haha.  R: Haha.  P1: I feel that is uhm brilliant marketing because everyone does it right and that would definitely be a great idea for the Accessercise app uhm for example if you did like I don’t know like a point system uhm where if you complete a certain amount of exercises per week or hit certain goals it does not necessarily need to be like Tescos but it could be a good idea something like that.  R: I think incentives are good because you want to first get people motivated to use the app.  P1: Yeah.  R: But they feel like they are benefiting in terms of their health but also benefiting from the reward so it works both ways and it’s helpful in a way.  P1: Yeah and I think yeah.  R: But in terms of nutrition what sort of things do you think ugh would be good on that?  P1: I think for a lot of people uhm even myself included if you are increasing the amount of exercise that you do uhm just like what you should be eating as well uhm well like ugh and it’s probably just because I don’t eat meat but also like alternatives as well.  R: Ok.  P1: So, like how do you increase your protein uhm without it necessarily meaning that you need to eat a steak or chicken or chicken and rice but haha like.  R: Haha.  P1: But like I was being very stereotypical there haha.  R: Haha.  P1: Uhm, but just like perhaps having different ways that people could increase certain nutrients or protein into their diets especially if they are changing or trying to improve their exercise or just in general how to maintain health like having an injury because I find that it can quite easily become something that you can become quite sedentary with like in terms of pain and fatigue and things like that so I guess nutrition that perhaps could increase uhm like iron levels or maybe make people less tired or vitamins that you might not be getting things like that.  R: Yeah, I think like an option where if you wanted to increase strength uhm what specific foods are good for increasing strength so for example when you design your workout and you choose your goal for example toning what food is maybe good to lose weight or maybe gaining weight if you’re a body builder or a power lifter or something like that so whether the goals could link into the nutrition would be quite useful but I think in terms of like food tips or maybe how you can cook a certain dish which is healthy or something like that uhm you know what food is good uhm in terms of like carbohydrates, proteins uhm and stuff like that to give users a bit more knowledge uhm because someone might use the app and look at the nutrition side and feel quite daunted and think oh gosh I’m not very good at understanding how to cook this or what to eat so if it has step to step uhm guides so like you know uhm have you ever seen those books for dummies.  P1: Haha yeah.  R: Something like that would be good.  P1: I also think you need to consider the accessibility that not everyone has uhm the same accessibility of like getting food or I guess like for myself uhm in terms of even like just using the cooker uhm I tend not to use the cooker these days because it involves bending done a lot more so instead we have like an air fryer which is on the work service which is easily accessible so a lot more gets cooked in the air fryer or on the hop like using gas because that is easier and more accessible for me to use than oven based things so I guess also then having an understanding that accessibility goes beyond I guess exercise as well uhm maybe if they were to add a section on the app on how to make a meal uhm and consider those sort of things as well would be great especially if they are looking at a range of disabilities.  R: Yeah, that makes sense I agree with that. Uhm, in terms of blogs uhm is there anything interesting that you would like to see from that uhm would you like to see other users make blogs or their workouts or even like monthly blogs?  P1: Uhm, I think it would be interesting to have it uhm like what they have done with the app you know where you can have it specifically tailored to your disability so I guess again like blogs on specific impairments would be really interesting.  R: Ok.  P1: Uhm, again I understand like for me spinal cord injuries is such like a broad thing because you can have a range of spinal cord injuries so I guess it would be interesting to hear from other people that have spinal cord injuries again I guess it’s uhm always useful to end up having like a sort of community especially because it’s not necessarily like a wide thing that happens uhm like not millions of people have spinal cord injuries.  R: Yeah.  P1: Uhm, especially in the UK each day not many people experience a spinal cord injury so I guess creating blogs would enable people to understand that they are not alone as well uhm I think like before I had my spinal cord injury like uhm it’s nothing that we would have to think about and because I got connected with like a charity called back up which is like a spinal cord injury charity and I got like a little buddy and like using that is like nothing is off the cards and everything that I experience and the symptoms I have like are not uhm uncommon for spinal cord injuries so having that thing where it’s like ok if you’ve uhm got this problem when you’re trying to work out and something happens like if you hear those stories from other people it makes it more accessible to use that app again.  R: Yeah.  P1: Again, it’s more like oh it’s fine like that is funny because that happened to me too or that’s not embarrassing and I shouldn’t stop going to the gym because those things happened or oh I faced this problem with accessibility of bathrooms in gyms like can anyone help uhm like having maybe like a forum like if you have people’s blogs to be able to comment on them or seek advice I think would be interesting.  R: Definitely!  P1: Uhm, it’s very much like nothing is off the cards type deal I guess haha like everyone has different struggles but it’s interesting that it’s a lot of common themes if that makes sense.  R: Yeah, makes sense. Perfect and what about podcasts would you like them?  P1: Again, uhm I think it would be interesting to hear from different people uhm and people like different uhm I wouldn’t say uhm different levels of disability so obviously like with a spinal cord injury uhm not everyone is in a wheelchair uhm like a lot of people ugh like for example I have spoken to people like my buddy is a wheelchair user and it turns out she’s a Paralympian uhm and I don’t know why they decided to put us together haha.  R: Haha.  P1: But she is a wheelchair user and I have also spoken to other people who are like I have a spinal cord injury but can walk entirely fine and then I am slightly in the middle where I will need to use a walking stick uhm I walk with a bit of a limp things like that just like a podcast would be interesting to hear from all those different types of people uhm how does sport or how can you incorporate sport with your symptoms I guess.  R: Yeah, makes sense.  P1: And like not having a generalised view I guess of what it means to have a spinal cord injury or have a disability because not everyone that has a spinal cord injury is in a wheelchair so that shouldn’t be the only representation of what you see of that disability uhm and yeah I think it would be interesting to have podcasts of maybe uhm difficulties or achievements or you could even include things like nutrition uhm things like that even uhm goals uhm it will definitely get a lot of people talking.  R: Uhm, yeah makes sense. Perfect, so we will move onto the Accessercise specific questions so some of these questions might not be relatable to you now but do you currently use Accessercise in your role and if so when and how?  P1: I found out about it like fifteen minutes ago haha so I’m going to go with no currently uhm yeah but in the future.  R: Perfect, ok so the second question I have got from me showing you the app earlier.  P1: Yeah.  R: Uhm, are what are your first thoughts when you first started using the Accessercise application uhm did anything stand out to you that you liked instantly for example the layout of the app?  P1: Uhm, I like the fact that you can share your goals or ideas it’s bit like uhm you know like the strava fitness app uhm where you can like uhm share like the walk that you’ve done uhm and it provides a map I like the fact that you can show your progress and show videos of you uhm.  R: So, you like that sort of element where other people can see what you’ve done and the social connectedness opportunities?  P1: Yeah, and I think it makes me a bit more motivated as well just to be like oh I’m still undertaking my streak and just like having that goal of like oh I need to do that today and it makes you incorporate exercise a bit more into your life.  R: So, do you think having a streak on the app is a good option?  P1: Yeah, but again I quite like the idea on the apple watch when you’ve completed a certain task like walking a hundred steps it notifies you with the rings.  R: Yeah, but I do find it quite annoying sometimes haha.  P1: Haha uhm It’s always telling you to achieve your daily goals for the rings on the watch I like the idea that it reminds you and just that goal or even if it was like oh you have like set this goal to I don’t know uhm do shrugs.  R: Yeah, I used to use an app called forest where like it incorporates the pomodoro technique where you have to study for fifty minutes but you have a ten minute break once you’ve completed it.  P1: Oh.  R: So, you have to leave your phone for fifty minutes and during that time the app is growing a plant but if you start using your phone during the session it starts killing the plant.  P1: Wow, that is brutal haha!  R: Yeah, haha I know so it like it notifys you that you have ten minutes left keep going and you will have a new plant and you then end up having like a farm of plants so it’s providing you the incentive where you don’t want to ruin the plant or kill the plant but you feel like you have to keep going.  P1: Yeah.  R: So, it’s quite similar to the streak option uhm where you might have one hundred days and you receive a notification saying congratulations you have reached one hundred days get a discount in the gym or get a discount from the shop or you know you don’t want to lose that streak because you have done so much and put so much energy into it.  P1: Even if you were to hit one hundred days I would definitely recommend having smaller ones as well like even for the first could of days because then people are more likely to fit into that habit so even if you had smaller ones where it’s like seven day streak uhm because I think it takes like thirty days to build in a new habit or something uhm so something for the first thirty days have more incentives to keep people more on track and then have it gradually go up so then it’s that fifty or one hundred days streak.  R: Yeah, makes sense.  P1: Just because I guess it’s more about getting people to use the app regularly and forming that new habit and I think that’s probably the crunch point normally at the start point because for example new year resolutions eighty one percent of people fail it by the end of January.  R: Yeah.  P1: It all comes down to what is the app going to do to keep people interested, I guess.  R: You have to find a way to encourage people to constantly use it.  P1: Yeah.  R: I think if someone uses the app and did not have those notifications or have those incentives it might be one of those things that people slip to the side for example new year resolutions uhm people do it for like two weeks and then stop using it because they don’t have any incentives to use it so I think the app developers need to find a way to keep people on the app and to find a way to motivate them to use it.  P1: Yeah, uhm yeah I would say that especially because you have got other like factors even if it’s just a regular person who gives up after two weeks uhm if you have got people who have days where it’s ugh difficult to get out of bed uhm because of pain or fatigue uhm you have also got to find ways that you can keep your streak if it’s on your worse day uhm not necessarily your best day.  R: Yeah, exactly something small at least it’s important that they do something.  P1: Yeah.  R: So, the third question I have got is do you have the required equipment to use Accessercise in it’s full capacity for example access to a mobile phone to use the application?  P1: Yep, uhm I do.  R: Perfect, so if you were at home are you more likely to use it on your phone or a tablet do you think?  P1: My phone.  R: Ok, uhm so from looking at the app so far from what I have showed you does anything stand out that you think ugh that the app developers need to improve for example oh sorry are there any improvements that you believe could help improve the Accessercise application uhm does anything stand out that you think gosh this could be good if they included this?  P1: Uhm, I guess obviously just the stuff that says coming soon would be nice to have uhm I really do like the uhm accessibility map feature uhm but I think you don’t necessarily need to just have gyms uhm I think it would be interesting if you could add things like uhm accessible sports groups maybe uhm especially like ugh I found it really difficult to find groups that are sport based that are accessible like if you google like spinal cord uhm wheelchair sports I can not find anything anywhere that like uhm means that I can meet people with a spinal cord injury uhm like I can play sports with.  R: Yeah, that’s one of the barriers to physical activity for people with disabilities uhm people find that if there are clubs they are normally too far away from them or they don’t know about it there’s a lack of advertisement for it.  P1: So, like I can’t find anywhere especially like I could potentially go to Cambridge which is like an hour away from me uhm like Loughborough is an hour away from me so like worse comes worse and it was something that I was interested in I could make that journey uhm but it’s the problem that I can’t find anything so maybe that would be an interesting thing to add.  R: Uhm, so to have like an option where you can see local clubs or local teams and stuff like that?  P1: Yeah especially for people perhaps or groups where it’s like sport related that perhaps uhm can allow people to meet people uhm I find that a lot of the stuff for spinal cord injuries uhm there are not many things where people can meet up that are close to people uhm and if so you have to apply or pay to attend uhm and it would be nice to have a sport group where I can meet other people with spinal cord injuries.  R: Yeah.  P1: And play sport.  R: Yeah, I feel like with the app it’s very much focused on like gyms exercises which is tailored for the individual and it’s not focused on much group work so it could be a function where you might want to work out with a friend together and it has the option where you can join a local group and you can go to the gym together uhm because we know for example if you have a spinal cord injury and going to the gym with a friend is more motivating and more rewarding than going alone so it would be good if they have an option you know locate a gym or socialising section where you can meet someone else to go to the gym with uhm I think that is something that they could add where you can find a local wheelchair basketball club or something like that uhm that has people with spinal cord injuries that want to do it with you because so far the app seems very much individualised but it’s very much on what the person wants to do which is good but then again people like doing things in groups sometimes with a bit of team work.  P1: Yeah and I think as well like I think they can probably not do this haha but it would be amazing if you did but even just like uhm it would be interesting if you were really interested in getting back into fitness uhm my local gym do uhm they have uhm personal trainers but personal trainers that are like uhm specifically trained in helping people that have had cardiac arrests or cardiac problems so that they can go to an accessible gym without it causing too much damage after a heart attack or so on and I thought oh that would be a really good idea for people with spinal cord injuries or a disability if you have people specifically trained or if there were personal trainers that had a specific knowledge or interest for example I’m really interested and this and now I know how to train or adapt workouts like that it would be interesting to have more of a say on like whether you’re doing it right uhm but I don’t think there’s many people out there that do that for example.  R: Haha it would be extremely helpful wouldn’t it!  P1: Haha it would be! Uhm, any other things ugh.  R: For example, if you were to flick through lets say the exercise options uhm if you select exercises and for example the descriptions uhm and then press show more do you think there is anything that they could add to this?  P1: Uhm, not everyone that has a spinal cord injury is in a wheelchair so even though it’s great that it’s wheelchair based it would be interesting if the app could provide videos of one person in a wheelchair and one person not in a wheelchair uhm if that makes sense.  R: Did you just say about the videos?  P1: Uhm, so not necessarily the videos but even for example the target muscle groups I guess it’s fine sitting down but even if you had an image of someone standing as well or uhm if you had a video uhm I guess you could do one standing but I guess it would depend uhm on the exercise because it would look different in a wheelchair compared to standing.  R: Right, ok.  P1: So, perhaps if people don’t have, I guess like that in depth knowledge of the gym or what they are meant to look at uhm maybe having both would help or having maybe a larger range of uhm representation I guess.  R: Yeah, makes sense.  P1: Uhm, but I like the videos I like the fact that it has captions uhm I’m definitely one of those people that has the subtitles on uhm I like the fact that the app tells me the key benefits of the exercise.  R: Do you feel like it has got enough uhm description do you think that it’s not too wordy it has got what you need there?  P1: Uhm, yeah, the only other thing I would recommend would be uhm if you could recommend uhm how many reps to do uhm.  R: Yeah, it has those in the exercise section.  P1: Oh, has it?  R: Yeah.  P1: Oh cool!  R: When you select your workout and you’re actually doing the workout it says how many reps.  P1: Ok, so how many to do perfect.  R: Yeah, it has that function already included.  P1: That’s cool.  R: Did you like the colour of the app so far?  P1: Uhm, the app is a bit too white haha.  R: Haha.  P1: Yeah, haha it’s very white and purple!  R: Do you think that is something that they could work on in terms of the colour scheme of the app?  P1: Yeah, unless they have done it for a specific reason uhm to make sure that it’s not too over stimulating I guess ugh but uhm again it could be more colourful but I don’t necessarily dislike the lack of colour uhm I guess uhm you know like on the iPhone you can have the option of putting it onto dark mode or light mode and it changes the screen?  R: Yeah.  P1: So, maybe they could incorporate something similar to that rather than changing the colours around.  R: Ok.  P1: Uhm, but other than that I think it’s pretty good uhm I think it just needs to have the rest of the stuff come out and then it would be good uhm.  R: Do you like the videos that they provide uhm do you think that’s quite useful?  P1: Yeah.  R: I think if I was to use the app and there weren’t any videos, I would be a bit sort of like uhm how do I do this.  P1: Oh yeah, I think unless you’ve been to the gym.  R: I think the visual demonstration of the exercises uhm you think like ok I can do this.  P1: You know when they give you options let’s say like you uhm do you like arm raises uhm do they only do it in the gym or is there videos where it shows someone using like a can or like a towel?  R: Uhm, that’s a good question! I haven’t looked too far into it but that could be something that the app developers could work on.  P1: You know how it has the option of like doing exercises at home with these like household things uhm maybe having a video of that rather than it always being in the gym because then you see what it looks like and it makes it seem a bit more relatable.  R: So, you like the videos not to just always be in the gym but actually in the park outside doing this or at home doing home exercises?  P1: Yeah.  R: Ok.  P1: Yeah, I think that would be interesting to see.  R: Yeah, I agree with that because a lot of the videos we are looking at our gym based whereas it would be good to see ok what is it like outside in a park uhm you know pushing something or doing something like that uhm I think they have to be diverse in the videos they are providing and not always just in the gym uhm but I’m the same as you I haven’t looked to far into the actual videos themselves but I’m sure there are videos of someone at home uhm you know.  P1: Ok.  R: I definitely think that would be a good recommendation is to provide a range of videos that you know are not always just in the gym because somebody may not use the gym and they look at that video and think ok the exercise is great but I haven’t got that equipment to use it.  P1: Yeah, and I think some people are visual learners and it would be interesting even if you have like you know videos that if you select the gym or home and for whatever option you select it like filters the videos so even if you had to do the videos in three different locations and it would not really be time efficient but if you have got it would be great uhm and it would be interesting if you had the location filter and that would decide or determine what videos you saw.  R: Ok.  P1: So, then if you clicked home, you would see all the ones from home and then if you press the one from the gym you would see all the ones for the gym and I think that would be a good way to incorporate everything in.  R: Do you like this section how it has got a way of working out you know the goal of your workout?  P1: Yeah, I really like that you can choose where you complete your workouts uhm I don’t think many apps have that option at all uhm I like the fact that you can choose a goal and the goal isn’t just like increase muscle uhm lose weight uhm I like the fact that it’s got a range of other things like stretching, mobility and flexibility because flexibility is a very good one haha because you’ll be surprised on how much your flexibility just disappears after a spinal cord injury.  R: Yeah, I like the option how you have got mobility, flexibility you know and stuff like that which is always pretty good.  P1: And I like the fact that then it doesn’t just show that exercise has to be something like being a body builder or losing weight it can be something as simple as flexibility and it then works around your goals uhm I like the fact that you can choose muscle groups uhm however it is not something necessarily I think would be used all the time I guess like if you’re trying to improve mobility, stretching or flexibility and you use yoga uhm you’re not necessarily then thinking I’m doing this to strengthen my shoulders.  R: Do you think for example if you press mobility, it would be good for the app developers to remove that section uhm because I think that is more focused on like muscular strength you know?  P1: I think when you know you can select your goal.  R: Yeah.  P1: And you click on something like build muscle, increase strength uhm or let’s say the top four on here uhm like then more options would appear uhm but if you click like flexibility uhm I don’t think the muscle component sections should be on there.  R: Yeah, I completely agree with that because at the moment I’m thinking that I have selected yoga but why am I looking at legs.  P1: Yeah, haha.  R: Uhm, why am I looking at like chest uhm so I think what they could do is you know you press it and it removes irrelevant options so there’s no confusion and you can go straight into you know like light yoga or easy yoga.  P1: Yeah.  R: You know that would be pretty good because at the moment the muscle components are still there.  P1: Uhm, I like the fact that it says require assistance or a carer I think that would be a great option to have.  R: What would you like to see from that function if you could if you were to use it for example it has got the option to have a carer there?  P1: I think yeah.  R: So, if you tick it then would you want it to notify the gym or would you want maybe someone to know in the gym that someone has entered the gym with a disability or something like that?  P1: I think that would be interesting in terms of like uhm if you were at a gym uhm I also kind of like the idea that if you clicked it shows you uhm maybe videos of like uhm for example not everyone who needs assistance has ugh like a carer like uhm I would say like in terms of for example my physio uhm well one of my physio involves like moving my foot like left and right on my left side and I cannot physically do that uhm so my partner helps me so even if they have videos where it was like showing how that person assists you.  R: Oh, ok I got you!  P1: Yeah, so it doesn’t necessarily mean that everyone has someone with them twenty-four seven or even a couple hours a day uhm and my partner is not a gym person in the slightest uhm he is the least sporty person that has ever existed uhm so if I asked him to do something uhm he probably wouldn’t want to cause injury.  R: Right, ok.  P1: So, I think it would be helpful to maybe include on the videos were the person providing assistance needs to stand or how to undertake the technique.  R: Oh, ok I see what you mean now. So, it gives them uhm advice and tips on how to support you achieve your goals?  P1: Yeah.  R: Basically, like tips and advice on what they need to do because I think when you press that option it may even be good if the gym is notified but it may actually be a good idea to get support on how the carer or assistant can actually help you achieve your goals.  P1: Yeah, especially if it notified the gym that someone coming in needs wider accessibility like uhm is everyone in that gym going to know how to support someone with a disability.  R: Yeah, a lot of the time they don’t due to a lack of knowledge.  P1: Uhm, and again it’s that preconception of what does a disability look like uhm is it someone in a wheelchair uhm is it an amputee is it someone who ugh uses a walking stick uhm is it someone with dwarfism like what does that look like.  R: Yeah.  P1: I do think it would be unrealistic to expect every gym worker to have a wide range of knowledge to help different types of disabilities uhm so I guess including tips or ways or even like me as a user being like oh I know I need support with this exercise uhm I can then tell that person how they can support me further.  R: Yeah, that makes complete sense.  P1: So, that I don’t have further injury.  R: I think at the moment the reason why when you press it uhm doesn’t do anything is because it’s such a new function and they are yet to update it.  P1: I’m so sorry haha I can talk for Britain.  R: Uhm, but I think that option is good to have but obviously at the moment it’s still like that because they are probably still thinking about what can we get out of this function but with the layout of these and the size of the text on the screen uhm do you think there is anything they can do to make it a bit more appealing?  P1: I think it would be so nice if the equipment they provide is in a row so like four per column instead of three and then the next row being four uhm it really bothers me haha but that is just me being picky.  R: I think sometimes it’s good to be like that because then you know you want people to look at it and think oh gosh that looks nice in terms of colours and so on.  P1: Yeah, of course.  R: At the moment, I look at that and I think it all looks a bit messy  P1: Haha, that’s what I was just thinking! Even like as well I guess uhm some of the things that they have are not necessarily appealing.  R: Do you think these options should be in alphabetical order?  P1: Uhm, yeah that would be really nice as well as more uhm I guess the only things you have got that aren’t necessarily things you would have if you weren’t a gym person so let’s say you have never been to the gym uhm you probably would have a towel uhm you would probably maybe have a bag uhm I can’t even see where it says water bottle uhm like the bottom three are the sort of things you would have at home like uhm I can understand why you have put prosthetic leg uhm but again maybe it’s something that could be tailored based on what you select or even if you have selected for example uhm like spinal cord injury uhm then it would remove things like uhm prosthetic arm, legs uhm potentially or maybe that’s things that could be hidden or if you were an amputee with a spinal cord injury then it could be something that you could add in or something rather than not all of it uhm because obviously you could add a very extensive list like uhm not all of it would apply.  R: Yeah, that makes sense.  P1: Uhm, again coffee table uhm I guess most people will have that.  R: Can?  P1: Haha but yeah then it goes home exercises.  R: I like the fact that they filter everything into home uhm equipment, gym equipment but at times it looks like too much on the screen.  P1: Yeah, and again like home exercises uhm but I was just thinking how many people have dumbbells at home or a kettle bell or a medicine ball like not me haha.  R: Haha.  P1: I’m saying that in the nicest possible way haha I definitely don’t have a treadmill at home.  R: Haha.  P1: Like I find it hard enough time walking straight as it is like.  R: I guess they should focus on filtering everything down a bit more.  P1: Yeah, I think maybe they need to be a bit more realistic about what people have at their house ugh and good on you for anybody out there that has a treadmill and dumbbells and all the other things because that’s definitely not me haha uhm especially like if you’re thinking about the app being for people with disabilities to access sport uhm with the problem of costs uhm like do you know how much a treadmill costs haha.  R: Yeah, I completely agree haha.  P1: Uhm, and then maybe again it just removes that idea of like oh should I have a treadmill uhm if I need to use the app uhm do I need to have a medicine ball uhm do I need to have dumbbells.  R: Yeah, there’s always an extra cost as well.  P1: Yeah, like do I need to get additional equipment to access the app and really in an ideal world it should be no.  R: Right, so let’s move onto the next question so uhm do you use any other similar applications outside of Accessercise if so, what is better or worse than Accessercise?  P1: Uhm.  R: So, I’m guessing you don’t use any other applications at the moment?  P1: No.  R: So, this is your first app?  P1: Yeah.  R: Ok, that’s fine. So, we will go onto the main section of the protocol, so we are now going to move onto the main part of this study and I’m going to ask you to participate in a think aloud protocol while you use the Accessercise application I promise you it won’t take that too long haha.  P1: Haha.  R: The instructions for the think aloud protocol are straight forward I would like you to say out loud whatever comes into your mind uhm there are no right or wrong answers uhm we will just run through a quick practice to get you used to the protocol so could I ask you to say out loud everything that comes to your mind when you think about physical activity?  P1: Uhm, so random words that come to my head?  R: Yeah.  P1: Uhm, sports uhm basketball ugh gym uhm gym shark haha.  R: Haha.  P1: Uhm, trainers.  R: Pain or fatigue?  P1: Uhm, yeah sore muscles uhm I’m trying to think haha but then that’s not a good idea because what sports do I actually do haha.  R: Haha.  P1: I guess like from the pool dancing I do uhm a lot of bruising uhm sweat uhm determination yeah.  R: Perfect!  P1: If that helps haha.  R: Haha. So, the main question is uhm I showed you the app you have looked through it a little bit uhm you know about the accessibility options uhm you know about the filters uhm you know about the exercises and so on but what were your first impressions or what are your first impressions of using the app uhm do you think that it is something that is in a good stage of progression or do you think there is more that they need to focus on?  P1: Uhm, I think it’s still in it’s early days uhm I think there’s a lot they can still include uhm I think that the videos uhm it’s good to get people starting and I would really like to see things uhm like the nutrition uhm the blogs uhm podcast stuff up and running uhm and I would also like to uhm you know the calendar function.  R: Yeah, so if you go back to the main section, it’s up there on the top left.  P1: Uhm, with the calendar section I’m not fully sure whether that links to my calendar on my phone uhm but I think that would be really good as well like having the workouts linked to the calendar on my phone because I use the calendar on my phone for everything.  R: Ok.  P1: So, maybe having the calendar function on the Accessercise app link to my personal calendar on my phone would be good so that will reduce having two calendars where I am like oh this is the exercises I need to do, and I then go onto the app and do it.  R: Oh, so you want it to link to your personal email like gmail, yahoo and so on?  P1: Yeah, if it could that would be great!  R: Yeah.  P1: Uhm, again I would like to go and have a go uhm and try things out haha uhm I would like to go and explore and things like that.  R: Do you like the fact that it’s focused on the impairment itself uhm it’s got the accessibility option uhm it’s got you know it’s trying to focus on different places where you can exercise.  P1: Yeah, I think that I like the fact that the app is primarily filtered based on a disability uhm I really like that uhm I like the fact that it shows the exercises based upon that uhm I think it needs work on how it visually shows that like uhm if I click outdoor uhm I would like to see someone doing the exercises outdoors uhm and not in a gym I think that would be nice uhm but I think me being me I probably would be able to relate to that because I used to go to the gym before my injury so I guess having that experience and having done some of these exercises it’s probably easier for me rather than someone that has never done it before uhm so yes I would like to give it a go.  R: Yeah, that makes sense and do you like the audio descriptions uhm do you like the people doing the exercises who are wheelchair users and stuff like that?  P1: Yeah.  R: What you can do is favourite exercises uhm so if you like a certain exercise uhm you star the exercise and then you press on the middle and you can view your favourite exercises so for example if you have a favourite workout that you like doing all the time uhm it can be added to that list which is great.  P1: That’s pretty cool.  R: Right, so I would now like you to use the application as you normally use it uhm please continue to speak ugh your thoughts as you move through the app uhm please show me all the features that you use and feel free to describe your actions, thoughts and feeling as you do so?  P1: Ok.  R: So, for example if you were to press onto Liv’s profile uhm.  P1: I will if I can get onto Liv’s profile.  R: Do you like the fact that it’s got a photo of her uhm you can follow her?  P1: Yeah, I think yeah I like the fact that you can follow people uhm I like the fact that you can make a group especially using that make friends function uhm it would be good to recommend local people in your area uhm I like the fact that you can see peoples achievements and followers uhm again what I think would be interesting uhm is to share the type of physical disability that you have.  R: Ok.  P1: So, like if they have a spinal cord injury they can share that.  R: So, it’s more relatable to other users?  P1: Yeah, so for example Liv could put that she has a spinal cord injury uhm it would then be interesting to see uhm and I think it would help people to find other people uhm that relate to them.  R: Hmm, do you think adding like a messaging function would be a bit too much in terms of privacy uhm because you don’t want the app to turn into like a dating app uhm where you can contact people and you can love heart their progress uhm do you think the fact that you can only comment on their workouts is a good idea?  P1: Yeah, yeah, I think that’s fine I don’t necessarily want to undertake a workout and someone can message me and say something weird haha.  R: Haha.  P1: I like the fact that like you know you can only react to workouts and send comments uhm maybe the idea of making groups would be good if that makes sense or working out in groups or for example if I make a workout.  R: I think it has a group session?  P1: Yeah, like if I made a workout uhm would I be able to share that workout with other people so if I made a workout that was like thirty minutes with all of these exercises uhm and I’m like this is really good for my arms uhm would I then be able to share that?  R: Uhm, at the moment no but that is a good point. Uhm, but going back to what you said about working out in groups what do you mean by that?  P1: Uhm, like.  R: Like a competition side or like leader boards with other users?  P1: So, there’s this thing in America and I have never done it because I don’t think I can win the money but they have this thing uhm and I can’t remember what it’s called but uhm maybe it’s called something like fat bet or something like that and basically you pay uhm put money in uhm to basically lose a certain amount of weight and you can join like ugh a competition and let’s say a thousand people put in ten pounds uhm and they wanted to lose a certain amount of weight uhm who ever loses the most weight wins everyone else’s money uhm and I’m not saying that is what needs to happen on this app haha but like having a bit of competition on the app uhm or a goal that people are trying to achieve uhm it doesn’t necessarily have to be weight lost uhm but even if it’s like oh uhm everyone is doing the same or similar workout to improve muscle strength or have groups based on similar interest so you where it’s got everything on the filter option uhm you know how you can filter your exercises.  R: Yeah.  P1: Uhm, let’s say perhaps you have a community which meant when you saw people’s profiles and you say you like yoga then everyone can see you like yoga.  R: Ok.  P1: Bless you.  R: Thank you.  P1: So, you can see like everyone who’s posted a yoga workout like their things if that makes sense.  R: Yeah, of course.  P1: You know like algorithms and all of that.  R: Yeah.  P1: It makes it more specific to you uhm I think that would be really interesting or people can see other people’s favourite’s uhm for example if I like doing light yoga then other’s can see that uhm or it could show me not just people that I’m following but random workouts uhm you know like an Instagram scroll.  R: Yeah, like Twitter where you can see other people’s posts on your feed?  P1: Yeah, but not necessarily something you want to be on for hours but it would be interesting to be like oh I did this workout and it’s brilliant uhm and I think what’s important for the app is all about bringing people’s lived experiences together.  R: What’s your thoughts on like Q and A for each gym uhm so sort of like having it available on each gym’s website so for example uhm if you weren’t too sure whether the gym was fully accessible uhm or even the equipment you could post it and the gym can respond allowing all users to see the answers?  P1: Yeah, like if there was like a comment option uhm so you can ask whether the place you are using has an accessible bathroom?  R: Yeah.  P1: Yeah, I think that would be extremely useful uhm and I think that would be interesting to see what other people think about that facility uhm and it’s overall accessibility ugh yeah I think a Q and A on the gym’s website would be great.  R: But uhm ok so if you were to go onto let’s say uhm the explore section now.  P1: Yeah.  R: And let’s say you zoom out uhm what you can also do is search the gyms using names for example if you search in the bar London up here.  P1: Yeah.  R: It comes up with a list of accessible gyms.  P1: Ok.  R: Do you like that?  P1: Yeah, I do.  R: You can also put a postcode in and it comes up with a list of accessible gyms uhm so it has got the option where you can zoom out and look at the map.  P1: Yeah, that’s cool.  R: Or it’s got this function where you can actually see it on the map uhm so if you were to press on it for example uhm a random one uhm it takes you to it uhm do you think the app developers could do more on this page uhm or do you think it’s a bit bland uhm could the gym do more on their behalf?  P1: Uhm, I guess if the gyms are saying they are accessible uhm it would be good to know what makes the gym accessible outside of what uhm another gym is like uhm just because you have like perhaps a ramp into the entrance and accessible toilet doesn’t make that an accessible gym like what makes it that accessible.  R: What’s your thoughts on having like a three sixty rotation of the gym so for example you can see the gym before you even use the gym?  P1: Uhm, I think that would be interesting obviously uhm again it’s like people’s privacy but if they can do that in an empty gym that would be great.  R: Haha I mean like an empty three sixty rotation of the gym.  P1: I think that would be interesting for people and I think as well like uhm since my injury I would definitely say that a lot of stuff comes down to a lot of pre planning uhm like if I wanted to join a gym I would need to know where bathrooms are uhm I need to know where I’m parking uhm I need to know how far am I going to walk uhm things like that uhm so I guess like having that three sixty would alleviate things like oh they have a door that is like uhm automatic and I won’t need to ask someone to open the door or they have a ramp and things like that so I guess it alleviates people’s concerns or worries about accessing those things that I might have that I guess loads of other people without an injury don’t consider.  R: I think that option would be good but if you were to press on it again uhm do you think so far that is ok or do you think there is anything more that they should think about including?  P1: Uhm.  R: They have the star option so you can see on this gym here that it has four out of five stars so it’s pretty accessible.  P1: Yeah, I guess if you could comment or like you know what makes it that four stars.  R: So, like a Q and A session for example?  P1: Yeah, I think that would be great uhm you know like trip adviser.  R: Yeah.  P1: You can like review and rate uhm I think that would be interesting like oh this is why it’s a good gym uhm and it’s accessible.  R: Ok, that makes sense.  P1: Or this is why I had a bad experience and I guess that would be interesting.  R: Ok, perfect. So, if you were to zoom out of that and then select the exercises option uhm so you have the notification section at the top right uhm do you like the fact that it can notify you if a user has done a workout or someone you’re following has done a workout uhm so that can even be like a little nudge so for example if you wanted to workout at eight on Monday morning uhm the app will then notify you to not forget that you have a strength workout today so it sort of nudges you.  P1: I really like that but I’m also one of those people where I really like that for myself uhm like I want to be notified when I need to workout and to motivate me uhm but do I want to follow one hundred people and have one hundred people tell me every time when they have done a workout uhm not necessarily like even if it’s like two separate notifications uhm like I can turn one off uhm one on if that makes sense.  R: Yeah.  P1: Uhm, I can be like oh I want the app to notify me when I am doing my workout or maybe people I favourited uhm but I don’t necessarily need it going off all the time if like I follow a lot of people.  R: Yeah, that makes complete sense I know what you’re saying.  P1: Sorry.  R: So, the last little things uhm so if you go to the more sections so we have got this one here.  P1: Yeah.  R: Do you like the colours on here do you think they could add more uhm to that section uhm so they have shop, nutrition, blogs, podcast uhm but do you think there is anything else that they could include?  P1: Uhm, not from the top of my head uhm I don’t necessarily know of anything else that they could include at the moment.  R: No worries.  P1: Uhm, it depends on how far you want to go with the app I guess uhm like under nutrition it could be the case where you have the opportunity to track that side of things or is it primarily focused on basic nutrition.  R: I just realised it has an invite friend section uhm I don’t think they had that the other day.  P1: Yeah, so you can share it.  R: Yeah, you can share it. Do you think that is quite a cool function that they have included?  P1: Uhm.  R: It would be pretty cool if you could invite friends but get like five percent discount on equipment from the app shop.  P1: Yeah, that would be cool but I guess it all comes down to different types of people because I wouldn’t necessarily invite the majority of people in my life who are able bodied uhm because they probably wouldn’t want to use an app that is primarily focused on disabled users.  R: Yeah.  P1: So, in my head I’m thinking what pool of people am I inviting uhm I could share it on support groups I guess but like I don’t know too many people that have a disability uhm like I know one friend uhm my brother is deaf so I could invite him haha but he can still access a lot of able bodied sports if that makes sense uhm yeah that’s fine he doesn’t want to be any different uhm you wouldn’t want to be seen as different uhm so I guess it all comes down to who you are inviting.  R: Ok.  P1: Haha.  R: So, overall it has got the social side uhm it’s got the exercises so you can design the workouts based on your goals uhm you can obviously set the workouts on certain days for using the calendar function so Friday morning I want to do this uhm I can then set it for the following Friday and the following Friday after that so what they are trying to do is make it very much a time saver so you don’t go to the gym play around too much uhm it’s very much this is what you want to do uhm favourite it uhm add it to your calendar which is good uhm so now we will move onto the overall thoughts of the app so the last few questions uhm so what are the potential positives if there are any of using Accessercise for improving physical activity levels?  P1: Uhm, I like the fact that it’s an accessible app uhm I like the fact that you filter the exercises based on what my disability is uhm therefore I’m more likely to use it because I’m seeing more representation and it’s more adapted to the needs of people that have an injury or disability.  R: Uhm, ok. Is there anything else uhm like any other positives of using the Accessercise app?  P1: Uhm.  R: Do you like the social side?  P1: Yeah, I like the social side uhm because you can add the workouts to your calendar, so it holds you accountable.  R: Perfect, ok. And what are the potential negatives if there are any of using Accessercise for improving physical activity levels uhm do you think there is anything that is negative of using the app?  P1: Uhm, not necessarily uhm I think that once you get used to the exercises uhm not much I think like if I had it on my phone trying to do an exercise uhm maybe it’s like where do you place your phone uhm but I think no uhm the only thing that may be an issue is if you start charging loads of money for the app.  R: Yeah.  P1: Uhm, I guess it then defeats the objective.  R: Do you think for example if someone wanted to use it and it’s like forty pound a year they would find that uhm a bit of ugh a negative of using the app because uhm the app might be amazing but obviously there is always a cost uhm because every company wants to make money out of you uhm let’s say for example uhm because I think it’s forty pound a year uhm that’s something that’s a reasonable price or do you think it’s something that is a bit too expensive for what it is uhm but I guess you could do like monthly payments for that uhm like two or three pound a month which is a bit more reasonable.  P1: Yeah, uhm I guess it’s considerably cheaper than a gym uhm.  R: I think uhm.  P1: I think I need to have time to like use it uhm and if I was like oh this is something that I have embedded because I have used it for thirty days uhm and I really like it then I probably would pay forty pounds.  R: Ok.  P1: But again, I wouldn’t pay forty pounds if they had a seven day free trial.  R: So, you would like a seven-day free trial of the app?  P1: I think for example uhm you know you can have some apps uhm where you get a free section that you can use uhm and then some have the paid bit uhm you can test that before you actually pay for everything.  R: I think a seven day free trial is always a good thing uhm because it gives people a better understanding of what is included on the app before they splash out forty pound on something that they may possibly not enjoy uhm but with the trial if they like it they may actually consider going for the full trial uhm because I think what happens is uhm people use the free trial uhm and then it expires and they are like ugh I want to continue using it they may consider the full membership uhm so I think it’s finding that balance uhm because we want them to use the app uhm so we will give them to free trial and see if they like it uhm and that’s important because you are trusting your users to have a go at using the app before they purchase it you know.  P1: And I think in the current state where the app hasn’t got everything available I don’t necessarily think asking people to spend forty pound a year when you can’t access things like nutrition or the shop and things like that would be quite unreasonable haha.  R: Yeah, makes sense.  P1: But maybe if there was like a different version and it had all those things and it’s offering that service then I don’t see why.  R: Ok, perfect and the last question is uhm how do you think the Accessercise app could be improved if anything for it’s use in improving physical activity levels for example if you had to name your top three areas for improvement what would they be so far from what you’re seeing?  P1: Uhm, get the shop, nutrition, podcast options up and running I would say uhm maybe uhm when it comes to the filter uhm having different videos uhm depending on your location I think would be my next improvement and lastly on that filter section just like removing the muscle group section if it’s not like you’re doing flexibility or stretching or something like that.  R: Ok.  P1: Those would probably be my biggest areas for improvement uhm and also having videos of someone undertaking exercise at home and not just at the gym in a wheelchair but uhm maybe having other representations based on the disability and not every disability on the app will mean people are in wheelchairs.  R: So, having videos of someone in the parking doing physical activity and not always based in the gym?  P1: Exactly.  R: Perfect, and is there anything else that you would like to add or discuss before we finish?  P1: Nope.  R: Well, perfect thank you so much for your interview, Abigail and I appreciate all your help.  P1: Haha, that’s alright! |
| --- |

## Transcript of interview undertaken with Participant 2

**Key code:**

R: Researcher

P2: Participant 2

| R: Perfect, thank you Paul for participating in our research uhm obviously coming here for the first phase but now coming here for the second phase to ugh get involved in this research so I do appreciate that so what I will do is I will start off the interview with doing an overview about the components that we are going to go through today so who I am is I am James Haley uhm and my role within this research project is that I’m the lead researcher so what that means is that I will be collecting all the data uhm analysing uhm writing the paper up and trying to publish the paper.  P2: Ok.  R: Uhm, the aims, importance and purpose of undertaking this research project and the benefits of participating is that we know during COVID-19 people with disabilities experienced limited physical activity as a result of ugh the covid lockdown restrictions and we found that the use of technology and the development of smartphones it could be a useful way to try and encourage and improve physical activity behaviours uhm the benefits of participating is that we will be able to evaluate a new app uhm see how good the app is but also find ways to improve the app so that more people can get involved which can improve physical activity levels but also reduce the burden on the NHS and reduce risk of other health complications.  P2: Great, ok.  R: Uhm, what University I attend so I attend Loughborough University uhm my position is that I’m a Doctoral Researcher ugh the faculty that I’m involved in is the Peter Harrison Centre for Disability Sport within the School of Sport, Exercise and Health Sciences and the other individuals that are working on the project with is Sam Breary which is the director of accessercise ugh Ali Jawad which is the co-director of accessercise and also my primary and secondary supervisors ugh Doctors David Maidment and Daniel Rhind uhm I will now quickly briefly outline all the ethical considerations in this study uhm so you have read the participant information sheet so you’re aware of what is involved in the study uhm you have signed the consent forms uhm in terms of confidentiality any data that you provide me today and last time will be deleted at the end of the study and the information that I will be analysing for this study uhm the qualitative data you will be provided a pseudonym so any information will be covered uhm but you are also provided the right to withdraw at any time if you feel like you don’t want to participate in this study anymore uhm and there are also no right or wrong answers in this study ugh do you have any questions that you would like to ask before we continue with the interview process?  P2: Nope.  R: Haha, perfect ok so the first introductory questions that I have got is please may you provide the following information so your name, age, gender identity, ethnicity and county of residence?  P2: Ok, my name is Hunt uhm forty four.  R: And your gender identity?  P2: Male.  R: Perfect, and your ethnicity?  P2: Ugh, White British.  R: White British. Ok, and your county of residence?  P2: Uhm, West Midlands.  R: West midlands ok perfect. So, to start could you tell me how you first got involved in the accessercise application and why?  P2: Uhm, I had an email forwarded onto me whilst I was at work uhm and then obviously got in contact with yourself.  R: Perfect, ok and why is this sort of research something that you would want to get involved with?  P2: Uhm, being a former athlete uhm I haven’t done much ugh exercise for the last ugh many years so uhm I have got a new hand bike uhm I have joined a gym ugh so it will be good to use this resource to have a look and use to motivate myself.  R: Perfect, ok and your experience of covid did you feel like it was restricted on how much physical activity you could have done?  P2: Uhm, no not really because I got out on my hand bike when the restrictions lifted a little bit uhm depending on what we could go and do uhm so the pandemic didn’t really impact me that much in terms of exercise uhm in fact I probably did more exercise haha compared to previous how many years haha to be fair uhm it was a horrible pandemic but it actually helped me get out and exercise.  R: Ok, so it benefited you.  P2: It was something to do.  R: I can imagine you got so bored sitting inside haha.  P2: Yeah, yeah haha.  R: Uhm, in terms of fitness apps in the past have you ever used any before?  P2: Uhm, I have tried.  R: Ok.  P2: And the reason for that is because of uhm my height and weight when you put that information into an app it doesn’t recognise it because I’m literally like two foot nine.  R: Right, ok.  P2: And I’m like eight and half stone and the app then goes like what haha.  R: Right, ok haha.  P2: So, something like this accessercise app uhm I imagine that would help because it’s connected to ugh the disability.  R: That’s an interesting point you have just mentioned so you have said about previous apps not really recognising your impairment uhm so this app is really much focused on the disability uhm do you like how this accessericse app is focused on a specific impairment.  P2: Yeah, definitely because it will be more accurate.  R: Ok.  P2: I have used apps in the past but they are not accurate uhm so it comes up red on the screen.  R: Right, ok.  P2: You can’t go any further uhm so because I’m like two foot and like eight stone the app gets confused and is like what is all of that about.  R: Haha, ok.  P2: Do you know what I mean haha which you can understand.  R: Yeah, so it doesn’t really recognise your weight and height.  P2: No, no uhm and the previous apps doesn’t recognise your disability does it.  R: What is quite interesting about this app uhm it doesn’t actually ask you for your height or weight.  P2: Oh, ok.  R: It just focuses specifically on your impairment.  P2: Right, ok.  R: So, it treats every impairment obviously as different but it categorises you within the impairment that you have selected.  P2: Yeah, yeah.  R: So, what it does is uhm if you had an amputee uhm you select the amputee then the app focuses itself around the amputee uhm and not your height and weight.  P2: Ok.  R: Uhm, so your goal could be like building strength and losing weight but then it really wants to focus on you as an individual and not your impairment of being you know weight and stuff like that so that’s something that is quite interesting about the app and interesting point that you have mentioned before so do you think that’s a bit of a limitation in the past of using apps?  P2: Yeah, other apps yeah yeah.  R: Yeah.  P2: Most definitely yes because you would have to put the different figures in uhm and it’s not accurate for me so uhm so most of the time I have had a go and then never used it again because it’s useless.  R: Ok, and in terms of obviously the apps you have used and the app that I have just showed you uhm do you think there is anything that you think could be added to this app that you have seen from previous apps so you mentioned about height and weight so obviously the other apps didn’t look at disability specific but did you like anything on previous apps that this app could maybe include?  P2: Uhm, on previous apps I like to see my sort of progression.  R: Ok.  P2: So, progression is important which you could do on other apps uhm and goals and things but as you have just gone through previously uhm you can do that on other apps which is what I like because that would motivate me to give me some targets to achieve.  R: So, you like the use of targets?  P2: Yeah, yeah absolutely!  R: Ok, so is that in terms of reps and sets uhm would you like to monitor your performance?  P2: Yeah, yeah I really like uhm so for example when I get back uhm after a ride on my hand bike uhm straight away I’m looking on the app uhm where was I at one point uhm how fast was I going and looking at the segments and things like that.  R: That’s pretty cool.  P2: I do like that.  R: Yeah, uhm I think on this app uhm when you are designing your workouts you can set the reps and sets but I think you can add it to your calander and look back on previous performances that way but I’m not sure if there is a way that you can monitor performance but I’m not too sure.  P2: Ok, so if you were to go back onto the calander uhm two weeks before?  R: You may be able to view your stats uhm.  P2: Yeah, so then you may be able to compare to a previous day can’t you?  R: Yeah.  P2: But I guess all the information you need will be on there won’t it.  R: Yeah, so reps uhm and sets but whether that’s laid out in the way that you would like it to be laid out.  P2: Right, ok.  R: So, what I mean by that is uhm I don’t think it has got a function where you can see frequencies and like graphs of how good you are over a period of weeks.  P2: Yeah, yeah.  R: So, maybe that could be something that could be included uhm it has all the functions but it isn’t laid out the way that most people may want it.  P2: Yeah, so you know with uhm the cardio stuff on there so if I do go on my hand bike uhm is there an option there to put that I have been on my hand bike and I have done so many miles?  R: Uhm, I think this app is more specific on like weights and also individual improvements on health uhm I don’t think it’s focused on types of sports if that makes sense?  P2: Ok.  R: It’s focused very much on like flexibility.  P2: Yeah, yeah.  R: But these are things that you could mention that could be included.  P2: I think that would be something that would be good.  R: So, would you like it to be adapted to other sports?  P2: Yeah, other means of exercise so that could be something like a racing chair uhm or a hand bike and then that’s all in one place then.  R: Right, ok.  P2: Because then I would do a gym session uhm alongside that as well.  R: Ok.  P2: So, you have got it all in one place then.  R: Yeah, I think maybe you could design your workout and then add something regarding a bike but I’m not too sure if that function is included but that could be something that gets developed and included so uhm that’s quite interesting.  P2: Yeah.  R: Uhm, so as I mentioned earlier ugh this question is difficult but could you please tell me your experience of using the accessercise app and how long you have been a member?  P2: Haha, literally ten minutes.  R: Haha.  P2: Uhm, I have been a member for haha uhm obviously I have not had that much time to have a look at it ugh and explore the app so it is a little bit difficult to answer really.  R: Ok, but have you heard of accessercise app before uhm is it something that you have heard from like friends or colleagues?  P2: No, uhm the only time I have heard about it is when I first came here to do your first phase of data collection.  R: Uhm, that’s quite interesting because I know one of the barriers to physical activity.  P2: I received your email uhm and then I thought I would get involved.  R: It’s interesting because one of the issues with physical activity for people with disabilities is a lack of advertisement.  P2: Yeah.  R: So, there’s a lack of opportunities so you may go from Birmingham or West Brom to travel to Manchester just to join a Wheelchair Basketball club uhm so with a lack of advertisement and a lack of opportunities uhm people don’t know about these opportunities uhm so it’s quite interesting that you said you have only just heard about this accessercise app from my email.  P2: Yeah.  R: This is something that they could develop and try to advertise more.  P2: I think a lot of it within the disability community is that when people here about it they tell others uhm so like word of mouth.  R: This is the focus uhm trying to get this app to a good standard so uhm more people use it.  P2: Yeah, yeah uhm it’s like the transport one for the trains uhm I have got it and I have told many people and they are now using it.  R: Oh, is there a transport app for disability sport?  P2: Yeah, yeah.  R: Ok, uhm does that show you what is accessible?  P2: Yeah, mainly for the trains.  R: Ok, so I’m guessing that shows you the time of the trains too?  P2: Yeah, yeah you click on find trains and the times come up.  R: Does it show you how accessible the train is?  P2: Uhm, the train will be accessible uhm but it may come up and tell you that a station is not accessible.  R: Right, ok I understand.  P2: And then it won’t let you book that train.  R: Oh, so is this like a disability specific?  P2: Yeah, it’s really good so going back to the question probably the word of mouth.  R: Yeah.  P2: The use of word mouth would be stronger I believe.  R: Ok.  P2: Because I have used that and I think it’s brilliant so I have told ten other people that will tell another ten people who will then start using it.  R: Yeah, and they will then tell another ten.  P2: Yeah, yeah.  R: That’s pretty good to have that uhm obviously uhm how can they find a way to encourage more people to use it and advertise that app you know would be interesting to know.  P2: Yeah.  R: That’s perfect so uhm these are very much specific ugh accessercise questions so do you currently use accessercise within your role if so when and how uhm so let’s flip that question ugh so if you were to use accessercise in your role how often do you think you would use it and if so when?  P2: I would use it every time I go to the gym.  R: Right, ok but would you also use it when you’re at home?  P2: Uhm, I have to admit I don’t do exercise at home.  R: Right.  P2: Uhm, I would rather go out to a gym.  R: So, do you treat your home as like your down time location?  P2: Yeah, yeah with family.  R: Right, ok.  P2: Uhm, if I started doing exercise at home uhm I would be divorced haha.  R: Haha, so do you normally go to the gym like once a day or is it once a week?  P2: I try to get there twice a week.  R: Right, ok.  P2: Obviously with work and personal commitments it’s harder some weeks uhm so last week I had no chance.  R: What’s your experience of using gyms do you go at certain times?  P2: Uhm.  R: Do you go when it’s not busy?  P2: I don’t like going busy times uhm and I think some gyms need probably more people to help really.  R: Right, ok.  P2: It’s mainly about reaching things.  R: Right, ok.  P2: Some gyms are adaptable so like you can move out the uhm you know the seats you can put your wheelchair in but that’s specific gyms like we have one Albury.  R: Ok.  P2: It’s all adapted and you can move things out uhm but my one at Sutton uhm you can’t move the seats out so if you can’t transfer you won’t be able to do it uhm you will be limited on what you can do in there uhm I think what is really important is getting the help in the gyms when you are there uhm it definitely needs to be made better.  R: It’s interesting that you say that because I showed you the function earlier about the carer and assistant and how you tick that and obviously at the moment it hasn’t been included but it’s something that has been included but they want to improve on uhm do you think that could be focused around uhm do you think maybe the gym could get more involved with the app or do you think the gym should be notified if you press that button you are going to the gym so someone can come over and support you uhm obviously it’s going to cost them more money maybe.  P2: It’s really annoying being disabled because you need to tell people like forty eighty hours in advance that you’re going.  R: Right, ok.  P2: Uhm, you don’t really need to do that do you haha.  R: No.  P2: You just go to the gym uhm and I know we are slightly different and we need help but I think people should be there already to help.  R: I think one of the issues with that is are these people trained in adapted physical activity and sport so for example if I was to teach wheelchair basketball uhm I wouldn’t know that because I have a lack of knowledge so whether these people could be trained on how to support someone with a disability could be another situation.  P2: Yeah.  R: But what I think is maybe notifications uhm so the gym are notified uhm so you just press it and it vibrates on their screens uhm that someone is coming in with a disability uhm they need additional support please keep an eye on them.  P2: Yeah, yeah.  R: Or what could be good is if you press that button then they get notified and they come over to you and help you.  P2: Yeah, yeah.  R: So, instead of like you having to tell them like forty eighty hours in advance uhm you just go in there as if you were more abled but when you actually need support uhm when you’re using the app and you’re looking at your performance uhm you just press the button and then someone comes over and supports you so that would be quite interesting.  P2: Yeah, I agree.  R: Uhm, but I think it’s good that they have already started thinking about this.  P2: Yeah, yeah definitely yeah.  R: I think that is quite a good thing uhm so obviously I showed you the app uhm I showed you different functions but what were your thoughts when you first started using the accessercise application.  P2: Uhm.  R: So, from what I showed you did you like the functions that I showed you?  P2: Yeah, yeah I liked the functions yeah uhm I liked the videos of each exercises because I would rather look at something being done than actually reading it myself.  R: So, what specifically do you like about the videos?  P2: Watching them uhm and seeing the technique of how to do the exercise correctly is a good thing they have included.  R: How about the person in the videos and someone with that specific disability?  P2: Uhm, I think it’s good that the accessercise app is focused on a specific disability so it could be a wheelchair user uhm you know and this is how they do it uhm I think the videos are really good because it shows you how other people do it uhm maybe you still need to adapt it a little but at least you have got the basic bits there uhm no I think the videos are great.  R: How about the filtering option where you can exercise at home uhm the gym or the park?  P2: Yeah, the location of workouts is good because it gives you options uhm if you can’t go out because it’s a snow day or something uhm you can then workout at home by using that filter.  R: I think that’s one of the problems uhm people think going to the gym you have to do physical activity and at home you can’t do physical activity uhm they are trying to give you the option that if you wanted to do it at home you can uhm so I think the app is very much focused around covid problems.  P2: Yeah, yeah.  R: Uhm where people need to exercise at home uhm so it’s very much adapting the techniques from then to now so that’s pretty good.  P2: Yeah, I agree.  R: Do you like the layout uhm so like the colour of the screen uhm do you like the white and purple?  P2: Yeah, the only thing is I’m thinking about visually impaired.  R: Right, ok.  P2: Uhm, so people that are visually impaired.  R: Right, ok.  P2: Is there an option there where they can change the background colour on it for the contrast?  R: That’s a good point uhm I don’t think they have that function yet but it’s funny because the app is focused on visual impairments uhm but I don’t know whether you can change uhm the colour settings uhm so do you think being able to change the settings in terms of colour could be better?  P2: It’s usually like font fourteen isn’t it uhm and like times new roman.  R: Right.  P2: That’s the usual uhm.  R: So, the option to include different fonts and sizes?  P2: Yeah, I think the option to be able to choose and change.  R: That’s interesting so uhm on the settings it would be good to have you know.  P2: Yeah, yeah like contrasts uhm I know visually impaired have got a voice over uhm option as well uhm and looking at include something like that would be good.  R: Ok.  P2: I don’t know if visually impaired will be able to see uhm the white but the black possibly they can see.  R: So, you think the option of having that you would like?  P2: Yeah, yeah.  R: Do you also like the calendar function where you can add workouts?  P2: Yeah, I like seeing what I have done and my progress.  R: Ok.  P2: Uhm, where I have get to so you can refer back to like uhm two weeks ago so I can see what I have done there uhm and yeah I really like the calendar setting.  R: That’s good uhm how about the social section uhm do you like how you can obviously follow people uhm they can then follow you.  P2: Yeah, it’s just like Facebook isn’t it haha uhm but I think it’s good having the social section uhm if people are ugh struggling with mental health as well and they are seeing other people sort of doing it uhm they can then chat with each other and maybe create like new friends or whatever uhm but can you do it privately?  R: So, that’s the group function so as you can see in the top right it says find groups.  P2: Yeah, yeah.  R: Uhm so that option you can actually follow and find groups.  P2: Ok.  R: Uhm, but do you like the function of where you can actually follow people uhm and follow groups uhm do you think the idea of like following I don’t know like wheelchair basketball teams could be something that you find beneficial?  P2: Uhm, I think it’s all about finding people with a similar disability to you.  R: Ok, so joining a group that’s focused on your disability?  P2: Yeah, I think it’s good because people are always on their phone aren’t they uhm messaging or whatever uhm socially I suppose uhm so maybe it’s good to ask questions.  R: Yeah.  P2: Uhm, how did you get on with that uhm how did you find that exercise and so on uhm is there any other ways that you have adapted yourself and other people can benefit from that.  R: Do you think the groups should not just be focused on physical activity and exercise but should be focused on other stuff in terms of like joining community groups where people talk about their impairment not just specifically on sport?  P2: Uhm.  R: So, you can join a group and people talk about like you know uhm I had this issues uhm I had this pain ugh what do you guys recommend or do you think that’s a bit too much?  P2: Would it be like a private option on there because you may want to do that but not want everyone else to be able to know if that makes sense.  R: Yeah, uhm at the moment it doesn’t have that option.  P2: I don’t know uhm if I followed Ellie Simmonds uhm and obviously I have some questions about something uhm I can then contact her uhm I don’t know uhm I think there should be like a private one on there.  R: Right, ok.  P2: So, if you were following somebody uhm you can message them.  R: Right.  P2: Instead of doing everything in big groups.  R: Yeah, that would be interesting uhm so a bit more communication there.  P2: Do you get what I mean?  R: Yeah.  P2: Yeah, yeah.  R: Do you like the idea of having groups?  P2: Yeah, I like it because I think it helps people.  R: Perfect, ok.  P2: Me personally I probably wouldn’t.  R: Ok.  P2: I think other people like that uhm so there needs to have an option on there like that.  R: Ok.  P2: Definitely yeah!  R: Yeah, that’s interesting but I think it would be a good option to be able to find people through sport for example if you like wheelchair rugby you can join a wheelchair rugby group.  P2: To be fair that would be good if someone in your area plays powerchair football you can find local opportunities through advertisement and so on.  R: So, the advertisement for local opportunities?  P2: Yeah, yeah.  R: Ok.  P2: Trying to get more people more active as well.  R: Yeah, that would be interesting. Uhm, so the next question I have got is do you have the required equipment to use accessercise in it’s full capacity in other words access to a mobile phone to use the application?  P2: Yeah.  R: So, if you were to use it in the gym uhm would you use your phone or would you use your tablet?  P2: It would be my phone.  R: Ok.  P2: A tablet would be too big haha.  R: Haha, yeah. Uhm, so from seeing the app is there any improvements that you believe could help improve the accessercise application uhm so is there anything that you think you know stood out that you think in terms of like the videos or you know the explore function where you can see the local gyms?  P2: I think the explore section with the map is good uhm so if you are aware with work or anything and you want some down time and you want to go and find a gym in Manchester which is accessible then that’s good.  R: Ok.  P2: Yeah, I think the map function is really good yeah.  R: But in terms of that map function, do you think anything else could be included?  P2: What do you mean?  R: Do you think anything could be added to that map function to make it better?  P2: Ugh, could you get up the map?  R: Yeah, so here you go.  P2: Ok.  R: So, if you were to zoom out you may be able to find some facilities.  P2: Sure.  R: So, obviously you have got all of that uhm you can get rate the gym at the bottom but do you think anymore information could be added there?  P2: I think if there is any equipment at the gym that is adapted.  R: So, whether there is adapted equipment?  P2: So, for the chair to come out.  R: Ok.  P2: So, you don’t have to transfer.  R: Right.  P2: Because some people can obviously not transfer.  R: Yeah, so having a list of the adapted equipment available at that gym?  P2: Yeah, uhm so is there adapted equipment.  R: Ok, interesting.  P2: But I think a three-sixty rotation video of the gym would be good to put on as well.  R: Right, ok.  P2: Uhm.  R: So, you can see the gym and facilities before hand?  P2: Cost maybe.  R: The cost of the gym?  P2: Yeah, yeah.  R: Uhm, but whether they have a link to the gym would also be good.  P2: Yeah, yeah a link would be good.  R: How about things like parking and stuff?  P2: Yeah, parking uhm also electric points for cars uhm I haven’t got an electric car but I’m thinking just overall really.  R: Right, ok.  P2: Yeah, yeah uhm whether disability parking is available.  R: Right, ok.  P2: You see it on hotels uhm.  R: Like trip advisor?  P2: Yeah, yeah uhm they have a list so like disabled parking tick uhm accessible rooms tick uhm you know what I mean uhm.  R: Yeah.  P2: Uhm, does the facility have guide dogs and things like that uhm they have to accept that anyway.  R: Yeah.  P2: It would be useful for them to definitely know about that.  R: In terms of the opening hours ugh the phone number do you think in terms of the rating system what could be done for the rating system so you have five stars ugh whether that could be laid out differently uhm so maybe whether you could see reviews?  P2: Yeah, yeah so like a trip advisor really.  R: Ok.  P2: So, like a review uhm so it says it’s accessible but is it.  R: Right, ok.  P2: You know.  R: So, what makes it accessible?  P2: Yeah, so if people go to the gym uhm they should review it to help other people.  R: Right, ok that makes sense uhm perfect so is there anything else on the app that you have seen that you think could be made better or improved?  P2: Uhm, not really because I haven’t really had a play to see everything really.  R: Right, ok.  P2: Uhm, first glance is really good I would definitely use it and I would encourage other people to use it as well.  R: Ok, and in terms of like the nutrition section uhm the shop do you think maybe that’s good?  P2: Uhm, the nutrition would be good I think because I don’t think people really understand much about nutrition really.  R: Ok.  P2: Including myself haha.  R: Haha.  P2: I’m always enjoying myself haha.  R: What sort of things would you like to be included on the nutrition section uhm do you think they could benefit from?  P2: Oh, that was another thing I noticed about previous apps uhm it does your height and weight on previous apps to understand how many calories you’re burning per day doesn’t it.  R: Yeah.  P2: But because it doesn’t recognise me I don’t know exactly what I’m burning.  R: Right, ok. So, that could be something added to the nutrition?  P2: Yeah, yeah.  R: So, when you put in your goals uhm so like increase strength it would be good to be able to put some information regarding your calories.  P2: Yeah, yeah it’s like the hand cycles in the gym and that uhm I’m not really sure if they are accurate really haha.  R: Haha.  P2: But I don’t know if something like that would help I don’t know.  R: Interesting.  P2: Podcasts uhm.  R: Is there anything interesting that you would like to see in the podcasts?  P2: Uhm, who would be doing the podcasts?  R: Maybe the users or the app developers uhm which could be interesting.  P2: I think podcasts regarding the accessibility of gyms could be good uhm but I’m actually unsure how they would do a podcast?  R: It could be like someone talking about their experience of using the app.  P2: Yeah.  R: Or their experience of doing exercise uhm so other people could listen to it and feel a bit more encouraged and motivated.  P2: Yeah, it’s a good option to have one there.  R: Sounds pretty good. Right, uhm.  P2: What was the shop uhm what was going to be in the shop?  R: So, at the moment the developers are thinking about having like accessercise t-shirts ugh water bottles.  P2: Oh, ok.  R: I was thinking whether people could be discounted if they do a certain amount of exercise per week.  P2: Yeah, discounts for the shop would be good to include.  R: So, if you used the app and you achieved your goals you get five percent of a water bottle.  P2: Yeah, yeah.  R: Or something like that just to get people motivated.  P2: Yeah, yeah.  R: It’s a way to get people convinced and involved on the app you know.  P2: I noticed here that you can invite friends which is really useful.  R: Yeah, you can invite friends.  P2: If I was to press onto that would it have to be friends that are on this app?  R: No, I think it’s invite friends from your phone contact list uhm so you will share the accessercise app link with them uhm so you know when you press it and you can share it?  P2: Oh, ok.  R: Do you like that option uhm where you can share it with other people?  P2: Yeah, because if you mention it uhm to one of your mates and then he may want to share it with someone else.  R: I agree that’s good. Perfect, ok ugh do you use any other similar applications outside of accessercise if so what is better/worse than accessercise?  P2: Strava.  R: Strava?  P2: Yeah.  R: So, your experience of using strava do you think maybe that’s got a lot better functions than maybe this app?  P2: Uhm.  R: So, what I find interesting about strava is that if you go running for example at the end of it uhm it shows you a map where you ran.  P2: Yeah.  R: So, if you were to do it outside I don’t know ugh let’s say you’re doing a gym workout at the park whether it could show you where you did that so something like that.  P2: So, here is my ride from Friday.  R: Right, ok.  P2: I love that uhm because it has segments do you know what I mean.  R: So, it shows you everywhere you have been?  P2: Yeah, yeah.  R: So, in terms of like strava the accessercise app is trying to do something similar to strava for people with disabilities uhm so whether they could use some of these functions.  P2: Like here on the strava app uhm you can save the route uhm so I can save this as a route and next time I do it can I beat these previous attempts.  R: Right, ok. So, it’s like an incentive uhm or motivation?  P2: Yeah, yeah that’s what I was going on about with the calendar function uhm really you know can you save an exercise?  R: Uhm, you can create one and add it to the calendar but I don’t think it has got all of these rewards like this one does.  P2: Yeah, but strava has been going a long time haha.  R: Haha, yeah.  P2: I suppose this app uhm accessercise is going to want to get it to this level don’t they?  R: Yeah.  P2: Hence why you’re doing this.  R: Yeah, that makes sense. Right, ok perfect. So, uhm we are now going to move onto the main part of this study uhm and ask you to participate in a think aloud protocol while using the accessercise application uhm the instructions for a think aloud protocol are straight forward I would like you to say out loud what ever comes into your mind uhm there are no right or wrong answers ugh we will just run through a quick practice to get you used to the protocol so could I ask you to say out loud everything that comes to your mind when you think about physical activity?  P2: Uhm.  R: So, what are the words that come to your mind?  P2: I’m not sure really.  R: Tough?  P2: Uhm, yeah fit haha.  R: Haha.  P2: Being fit haha.  R: Healthy?  P2: Yeah.  R: Hard, difficult?  P2: Yeah, yeah of course.  R: Perfect, ok so what I would like you to do now is show me your screen uhm so we will go through step by step ugh so if you were to go to the exercise section.  P2: Ugh.  R: Right at the bottom.  P2: I need glasses.  R: So, obviously at the top you have got your calander uhm you have got notifications do you like the notifications how you are notified or would you not like to be notified that often?  P2: So, what do the notifications do?  R: So, at the moment it just tells you that you know uhm you need to do your workout for today uhm so it sort of nudges you.  P2: So, like a reminder?  R: Yeah, do you like that function uhm do you think it’s something that is a bit off putting?  P2: Uhm, I could leave it or take it really.  R: Right, ok.  P2: Uhm, yeah.  R: And the exercises you see down here do you think they could be laid out differently like maybe uhm categorised uhm because at the moment you can see loads of ugh exercises ugh so whether you know could be laid out better maybe?  P2: So, where is the one where you can put in uhm so for the disability?  R: Oh, selecting your impairment?  P2: Yeah.  R: So, if you go off this haha and you scroll right down from the top uhm and go down to the bottom and press more.  P2: Yeah.  R: And press ugh account settings and then go to impairment filter at the top you can then select your impairment.  P2: Oh, ok.  R: At the moment it’s only got a couple at the moment.  P2: So, I would probably come into this category at the bottom really.  R: But if you press paraplegia and then save that and save in the top right it will then shape things around based on the impairment you selected.  P2: So, if I go back to the main screen will my exercises changes?  R: Yeah, so it will shift everything around uhm from someone with dwarfism to someone with a spinal cord injury so now you can see the videos they are in a wheelchair.  P2: These videos are good uhm they are brilliant.  R: It’s nice because they have done a video for every single exercise uhm there are over two hundred and fifty so you know uhm they have put some hard work into that.  P2: I really like that uhm so that’s the closest to me really.  R: Ok.  P2: You know what I mean?  R: Yeah.  P2: He’s in a wheelchair.  R: So, that’s something you would follow?  P2: Yeah, yeah the videos are really good.  R: That’s good. Uhm, so everything is pretty much filtered down so because you have selected spinal cord injury it’s then got someone in a wheelchair and the reason why they have done that is so that the person feels more related to the person in the video because if I’m sat doing it and you’re in a wheelchair uhm you’re not going to feel motivated.  P2: No, no I think that’s amazing those videos.  R: But if you were to press on the exercise.  P2: Yep.  R: If you were to press that and then you go to show more do you think there is anything that they could improve on uhm in terms of the descriptions do you think they could use bullet points or step by step instead of just a big block of text?  P2: Yeah, they could use bullet points for the exercise descriptions to make it more easier uhm or even step by step uhm because at the moment it’s just too much information.  R: Too much information?  P2: If that is for a visual impairment uhm a person with a visual impairment that would be too much uhm it needs to be spaced out a little.  R: Yeah, so spaced out but do you think less text maybe?  P2: Yeah, possibly.  R: Because even for me I find there’s too much text ugh it’s too heavy.  P2: Yeah, it is.  R: But do you like the key benefits section?  P2: Yeah, I like that the key benefits section.  R: Ugh, you have got like target groups as well.  P2: Yeah, so it shows you were you are working which is nice.  R: So, obviously the purple is what muscles you are working.  P2: Yeah, the photo of the muscle components I like that.  R: Do you think there is enough information there or do you think they could maybe narrow it down?  P2: Uhm, I don’t know because that’s probably what you’re going to be using in that exercise.  R: I think what is quite nice is that they don’t over complicate anything uhm they keep it simple uhm so there’s a few things on the page regarding the exercise uhm so if you were to scroll to the top uhm here it’s got back, arms and so on uhm you can also favourite as well.  P2: Ok.  R: You can favourite that and when you go to your favourite list uhm at the beginning you can then add it to your workouts uhm so that’s pretty good.  P2: Yeah, I like the favourite option that’s quite handy to have on the app.  R: But uhm what other ones are there ugh so what we will do now because we are coming towards the end is uhm what do you think are the potential positives if there are any of using the accessercise app for improving physical activity levels?  P2: Sorry can you say that again?  R: Haha, what are the potential positives if there are any of using accessercise for improving physical activity levels?  P2: Uhm.  R: Do you think there are any positives that people could benefit from using this app?  P2: Just using it as a motivation tool.  R: Ok.  P2: Uhm, which I think goes back to the point I made about setting targets and goals and things like that.  R: But going back to a point we discussed earlier so if you were to go onto social.  P2: Uhm, yep.  R: And you go to following.  P2: Yep.  R: And you press on Liv’s profile.  P2: Yeah.  R: And then you can see achievements uhm what sort of achievements do you think you could benefit from uhm do you think things like streaks ugh leader boards ugh competitions might help?  P2: I think you have to be quite careful doing sort of like competitions because somebody uhm I don’t think so I think the achievement section has to be personal to you.  R: Personal right ok.  P2: I’m not sure really.  R: So, you think personalised achievements?  P2: I just think maybe like uhm some people might be able to do it or not do it and it might demotivate them uhm because that person has done twenty five more exercises than me and so on.  R: Yeah.  P2: I don’t know because if they have done twenty-five more than me uhm that might be because they have less problems than me that’s why I am not at the top of the leader board.  R: Right, ok.  P2: I could still do the exercises but there might be other reasons that prevent me from making good progress.  R: So, do you think competition might be too much for users?  P2: No, I think personal achievements I think would be good.  R: Yeah, I know what you mean because you don’t want to go on there and someone is ten places above you and you feel like oh I can’t do this.  P2: Yeah, I can’t do it because my impairment is more severe than someone else which will hinder my performance if you know what I mean?  R: Yeah, so going back to the nutrition section so do you think the idea of having like educational videos in there about uhm what a protein is uhm what a carbohydrate is could help?  P2: Yeah, yeah educational videos for the nutrition could be good because you’re teaching people which is good practice really.  R: Yeah.  P2: Yeah, educational videos I think would be good.  R: Perfect, ok awesome.  P2: Maybe like someone doing ugh a food diary or something and there’s some examples of good food and so on.  R: Yeah, that makes sense but do you think the idea of maybe having frequency tables and like uhm where you can see how often you used the app so like statistics on there and stuff like that?  P2: Ugh, like they do on other apps?  R: Yeah.  P2: Possibly.  R: I think the calendar function is great you can add all your workouts but I don’t think you can statistically see how often you use the app and stuff like that.  P2: Yeah, yeah that would be great.  R: Uhm, so whether they could add that in would be great.  P2: Yeah.  R: Uhm, because I think that is one function that they are missing.  P2: The analytical stuff.  R: Yeah, obviously there is a reason behind why maybe they haven’t included it.  P2: Yeah, there’s obviously some reason.  R: They are probably just trying to keep the interface simple.  P2: Yeah, yeah.  R: There’s obviously so much stuff we could include but we need to make it better obviously.  P2: Yeah.  R: Perfect, ok so we will go onto the last two questions uhm so what are the potential negatives if there are any of using accessercise for improving physical activity levels uhm so do you think there are any uhm actually weaknesses of using it?  P2: Again, it’s a little bit difficult to comment on that because obviously I’m quite new to the app and have only briefly gone through it uhm but I can’t see any issues uhm the only thing I would say which I said at the start is possibly the fonts uhm and the size of the fonts uhm also the background and the contrasts uhm maybe ugh I don’t really know.  R: If you had to round everything up what would you say is your favourite part of the app?  P2: Uhm, I really like the calendar setting on the app uhm adding your workouts.  R: Oh, so where you can choose your workouts for the week and so on?  P2: Yeah, designing your own workouts yeah and I also really like the videos because they show you how to actually do the exercise because some people don’t know how to do it properly and the technique is so important to get it right.  R: Yeah.  P2: People put loads of weight on the bar but can’t lift it you know what I mean haha.  R: Yeah haha.  P2: It’s like what’s the point they can only lift it a little bit uhm there’s no point uhm you get people like that do you know what I mean so I think getting the right technique is essential.  R: Getting it right is so important.  P2: Yeah, it’s the most important thing the technique.  R: That makes sense ok so the final question I have got is uhm so rounding everything up from this think aloud interview how do you think the accessercise application could be improved if anything for it’s use in improving physical activity levels so if you had to think about three key areas that we can take away from this interview for the developers to improve the app on what you have seen and what you known before what do you think they could be?  P2: Uhm, definitely the visual impairment part.  R: Ok.  P2: So, the contrast.  R: Ok.  P2: So, an area for development for the accessercise app is having the option to change colours on the app so that it can help someone that has a visual impairment using the app.  R: Ok.  P2: Uhm.  R: In terms of maybe the explore function so if we were to do one for every section so uhm the visual stuff is more for the settings but how about the explore section?  P2: Yeah, I just think when you press on a gym on the explore section it should have a bit more information about that gym uhm in terms of it’s accessibility so what is accessible uhm what equipment is accessible uhm that would be really important.  R: Right, ok.  P2: I think that accessibility rating section needs to be more detailed.  R: Ok, in terms of the social section do you think anything could be improved on that?  P2: Ugh, not really I just think there has to be an option on there for private messaging uhm probably.  R: Ok.  P2: So, if they have got a question uhm they can ask someone privately because I don’t think some people want to tell every body uhm you know what I mean uhm so for example if there’s someone with the same condition as me and they are asking me a question I don’t want everyone to know my answer you know what I mean?  R: Yeah, makes sense. So, you want to have the option on there to message privately if you can?  P2: Yeah, yeah.  R: Ok, and the last one is the actual workout section uhm so the actual exercises uhm do you think there is anything that could be improved maybe in the filter option?  P2: Uhm.  R: If you were to go onto filter actually uhm do you like how everything is laid out in like blocks uhm how they have little logos next to each goal?  P2: Yeah, it’s all clear.  R: Ok, so there’s not too much information there?  P2: I think they just need to improve on the colour but I assume that will change over time.  R: Ok, and here can you see how it’s four four two.  P2: Sounds like a football formation doesn’t it haha.  R: Haha.  P2: Four four two haha.  R: Haha, so you have four four two uhm if you were to scroll down and press outdoor training and you have like two two and then two again uhm but also here you have like three three two.  P2: Oh, yeah I understand.  R: Do you think maybe they could put it in alphabetical order?  P2: Ugh, yes alphabetical would work so I know what you’re saying yeah.  R: I guess if you’re looking for certain equipment if it’s alphabetical order it’s easier to find isn’t it.  P2: Yeah.  R: Compared to having it all in different orders like three three two.  P2: Yeah.  R: I’m also thinking that if you select home uhm then select the goal of improve strength and then chest it reduces all the options that are not related to that.  P2: Yeah, so sort of takes them out.  R: Yeah, so obviously if I’m doing flexibility I’m not going to need a bench press.  P2: Haha.  R: Haha, so whether it could reduce options based on what you press.  P2: So, having it a bit clearer.  R: Yeah.  P2: That would be good.  R: So, it provides you less options for when you press certain things.  P2: Yeah.  R: So, for flexibility then the equipment option you see will just be something like a yoga mat.  P2: Yeah, yeah.  R: It’s not going to be dumbbells or anything like that.  P2: No.  R: So, whether they could limit that down would be pretty good.  P2: Yeah.  R: Perfect, ok so that’s everything uhm so the final question I have got is whether there is anything else you would like to add or discuss before we finish the interview?  P2: Uhm, no not really uhm I just want to use it haha.  R: You want to get involved with it now haha.  P2: Yeah, I want to get started haha  R: That’s good uhm but you like the functions you have seen so far?  P2: Yeah, the functions are good but is there an opportunity to obviously in a couple of weeks time when I start using it more can I contact you to provide some feedback?  R: Yeah, of course yeah.  P2: Ok.  R: Yeah, if there’s anything you feel like adding please feel free to let me know.  P2: It’s quite difficult at the moment because I haven’t probably use it if you know what I mean.  R: Yeah, that’s perfectly fine.  P2: The first moment I started using this app I thought it was really good.  R: That’s really good.  P2: I do like the app.  R: That’s good to hear I’m happy you enjoy using it and I hopefully it is something that you can benefit from once you finish participating in this study.  P2: Yeah.  R: Perfect, thank you so much for your time.  P2: No worries. |
| --- |

## Transcript of interview undertaken with Participant 3

**Key code:**

R: Researcher

P3: Participant 3

| R: So, thank you Michelle for participating in this research so in the first phase as well uhm as well as the second phase uhm I appreciate your time and willingness to come to Loughborough and support the research study here uhm so first of all I will go through some interview questions uhm so the overview of who I am and so on uhm so the first one is who I am and what my role is within this research project so my name is James Haley and I am the lead ugh researcher within this project uhm so I will very much be undertaking all the data collection uhm but also analysing and writing up the paper ugh the aims, importance and purpose of undertaking this research project and the benefits uhm of participating is that we know during covid people with disabilities were not physically active as a result of the covid lockdown and the restrictions uhm and we found that with the development of technology and people now using more smartphone devices uhm using apps and so on at home could be a good way to improve physical activity but also reduce the barriers that they experience when it comes to taking part in exercise such as cost, transport and also travel ugh and the hopes of undertaking this research is that we can identify the strengths, limitations and also areas of development for the accessercise app so that the app developers can improve the app further so that more people in the community can get involved in this app and improve their lifestyle and quality of life.  P3: Ok.  R: So, the University that I attend is Loughborough uhm my position is that I’m a Doctoral Researcher and the faculty that I’m involved in is the Peter Harrison Centre for Disability Sport which is based within the School of Sport, Exercise and Health Sciences and the people that I’m working with on this project are the developers of the app so that is Sam Breary who is the director of accessercise and Ali Jawad who is the co-director of accessercise but I’m also being supervised by Doctors David Maidment and Daniel Rhind which are my main primary and secondary supervisors uhm I will also go through uhm the outline of all the ethical considerations in this study uhm so you have read the information sheet uhm you have signed the informed consent form which you are happy with uhm any information that you provide within this study will be confidential so any comments that you mention will be hidden with a pseudonym which is basically an alternative or fake name so no information will be linked to who you are uhm you are also provided the rights to withdraw from this study at anytime if you feel like you do not want to continue anymore so please feel free to let me know whether you would want to withdraw or not and you don’t want to continue anymore with the interview and before we start there are no right or wrong answers in this study uhm do you have any questions you would like to ask before we continue with the interview process?  P3: No.  R: Ok, perfect ok so the first questions I have got uhm are very much introductory questions so please may you provide the following information so your name, age, gender identity, ethnicity and county of residence?  P3: Yeah, my name is Michelle Rowlands uhm I’m forty-two and what else did you say?  R: Uhm, your gender identity?  P3: Oh, I’m female ugh female from birth.  R: And your ethnicity?  P3: Ugh, White British.  R: Ok, perfect and your county of residence?  P3: Ugh, Derbyshire.  R: Perfect, ok so to start could you please tell me how you got involved in using the accessercise application and why uhm so why you wanted to get involved in this sort of research?  P3: Uhm, yeah I believe it was an email from yourself uhm I wanted to get involved because I was once a powerlifter uhm extremely fit and then I had an injury uhm in my spine uhm I have actually had spinal surgery I think in twenty-twenty uhm so I’m still under a surgeon uhm and that was experimental surgery that I had at Nuffield health uhm so I’m under a surgeon for the next five years uhm from being mega fit to not being able to run uhm to also putting weight on is horrible uhm it’s an actual horrible feeling uhm I can’t do the things that I have done before my mood is more lower because I don’t look like how I used to look uhm my motivation has disappeared uhm because I’m always thinking I’m going to hurt myself.  R: Yeah, I understand.  P3: Uhm, and that is why I want to get involved in this app research really uhm to try and get me more confident in myself and understand that I can do things uhm because there are other people out there that are doing it uhm maybe they are worse than me you know uhm but they’re doing it so I should be able to do it as well.  R: Ok, so do you feel like you want to get involved in this app work because you want to improve your overall health and to get involved more?  P3: It’s to improve my health uhm and to get back into fitness because uhm I am currently nervous of the gym now because I think I have wraped myself up in cotton wall.  R: Ok.  P3: Uhm, and I am being told that I can’t do this uhm I can’t lift uhm I can’t run uhm so being told that by a professional uhm I have just wrapped myself up in cotton wall where I’m scared to do things so I’m quite nervous when I step into a gym uhm also because I think people are looking at me as well.  R: Ok, uhm so obviously this study involves using an app uhm have you used previous apps before?  P3: Yeah, yeah. I have used apps in the past.  R: Perfect, ok so what was your experience like using them before uhm was it helpful?  P3: Uhm, sometimes they are helpful uhm but I think the motivation is easy to just put your phone down and think oh I cannot be bothered now.  R: Yeah.  P3: Uhm, so it needs something uhm especially for myself I need something that is going to keep me doing it uhm I need to do this because I will get a bonus level or something like that.  R: Yeah.  P3: Or Google uhm when it gives you money uhm google play when you answer things.  R: Oh, yeah, I know uhm when you complete the surveys and they reward you with a bit of money.  P3: Yeah, something like that uhm so if you do this exercise, you will get some money.  R: Right, ok.  P3: Uhm, that will help me a lot uhm because I do that now when I get a warning uhm saying that I have a google questionnaire uhm I get some money.  R: Ok.  P3: So, for me that is what motivates me.  R: Right, ok.  P3: Uhm.  R: So, do you feel like you need some sort of motivation to use the app?  P3: Yeah.  R: Ok, so, would you say motivation is your biggest barrier when it comes to physical activity?  P3: Yeah, yeah. Motivation is my biggest problem.  R: Perfect, ok so you mentioned about obviously finding long term strategies to improve your motivation uhm is there anything in particular that you would like to see uhm so you mentioned about questionnaires but from an app perspective is there anything that you think they can add to the app to make that easier for you?  P3: Uhm, yeah more people like me I suppose could be added to the app.  R: Ok.  P3: Uhm, having people with the same disability so if you’re a wheelchair user uhm having a lot of wheelchair used stuff would be really good but I think for myself ugh I have a rare spinal condition so if someone else has that uhm I can follow and if they are doing it then maybe I can do it.  R: Right, ok.  P3: That kind of thing.  R: So, going onto that point so as you saw on the app it is very much filtered based on the impairment and it has got that social section where you can follow people with that impairment uhm do you like that function?  P3: Yeah, yeah I do I think tailoring the app to the disability is great.  R: Yeah, so you like the fact that you can follow people and there’s that relatedness?  P3: Yeah, the option to follow and have followers is really good to have.  R: Ok, uhm perfect ok so ugh you very much want to use the app to tackle some of the barriers in terms of motivation to get physically active?  P3: Yeah, I want to overcome physical activity barriers.  R: Ok, awesome uhm so this is a bit of a trick question but could you please tell me your experience of using the accessercise app and how long you have been a member?  P3: One day.  R: Haha, so not very long then.  P3: Uhm, no so I have looked through it uhm I have not used it yet because I downloaded it last night uhm but it seems fine uhm the colours of the app seem fine uhm the content seems understandable uhm yeah it just seems to be really easy to get around which is a really key thing really.  R: So, you find it quite easy to navigate through?  P3: Yeah, the app is really easy to uhm navigate through.  R: Did you find it quite easy to ugh find the accessercise app because I know from my experience uhm when it comes to physical activity that one of the issues is ugh a lack of advertisement for these sort of things uhm did you hear about the app before I mentioned it?  P3: Uhm I have never seen it ugh before uhm but when I put it into the google play store uhm I found it straight away when you told me what it was.  R: I think one of the issues is that these apps are great and we are obviously evaluating the accessercise app now but if it’s not advertised then no one is going to know about it.  P3: No.  R: So, I think it needs a bit more of a push for these things uhm through advertisement and they need to definitely be advertised better.  P3: Yeah.  R: The problem is that it comes down to funding you know uhm doing a TV advert it costs a lot of money uhm so whether it could actually be advertised through communities and so on would help.  P3: Uhm, yeah, I think word of mouth would be a big thing uhm but obviously uhm you have got your armed forces as well which have amputees and stuff like that so going towards ugh the veteran community which have been injured uhm Stanford Hall which is in Loughborough.  R: Right, ok.  P3: Ugh, they have all got uhm really bad injuries.  R: Right, ok so that would be a really good place to promote?  P3: Yeah, that would be a really good place to start.  R: Uhm, I think the app developers at the moment are going to different places around the country to advertise that.  P3: Yeah.  R: So, I think they are collaborating and very much working with people but sometimes I think it needs a bit more of a push just to get more people involved.  P3: Yeah.  R: I think that’s because if we found out that this app is really good uhm then how did we get it across to people so that more people can use it.  P3: Ugh, yeah. I think it’s going to be at first uhm more people using it and then uhm advertising it more.  R: Yeah, applying that technique.  P3: And I think that if I use this app and I lost some pounds uhm I would be mega chuffed with myself and I would automatically think that it’s that app that has done that and so then I would tell my friends uhm I would then put in on social media oh I have lost some weight great uhm I have been doing this and I think that’s.  R: Encouragement for other people?  P3: Yeah, and that’s how it gets out.  R: I guess yes you can be a role model for other people uhm so if it works for you uhm it will work for other people.  P3: Yeah.  R: So, I think what is good about these videos is that yes you select your impairment and then you see somebody with that impairment doing the exercise in the video.  P3: Yeah, the videos are helpful seeing someone else with the same impairment.  R: I definitely think that is helpful.  P3: I agree. I am a huge fan of the videos on accessercise.  R: Uhm, so some of these questions are very much about the accessercise app so obviously you haven’t used it for that long but do you currently use accessercise within your role and if so when and how uhm so if we flip this question uhm based on your previous answers uhm so if you were to use accessercise from today how often do you think you would use it?  P3: So, because I do long hours uhm as I do fourteen-hour night shifts uhm so I would probably use it instead of sitting on my ass haha reading.  R: Haha.  P3: I would probably go and use it then.  R: Haha, ok.  P3: It’s because it’s something there uhm I did ugh about seven days ago download a dance app which I haven’t used yet.  R: Right, ok.  P3: I paid for that forty-five pound.  R: Uhm, ok.  P3: So, uhm for this being free as well.  R: Yeah.  P3: It’s saving me money yeah a lot of money and it’s there.  R: I think what’s a good thing about this app is that uhm I think for the whole year it’s sixty-five pounds so you know people first download it uhm they then use it for a year uhm that sixty five pound is equivalent to three months in a gym.  P3: Yeah, the membership cost overall for accessercise is ugh really good.  R: These exercises you can actually do at home uhm so the long term it’s saving people a lot of money.  P3: Yeah, I agree, and I think uhm so for example you have got yoga included on the accessercise app uhm but I think it needs to have something that’s a bit different a bit uhm fun like a dance.  R: Right, ok.  P3: Because you can dance in a wheelchair uhm you can dance with one leg uhm you can be myself and dance uhm and that’s fun.  R: Ok, so you think not just focusing on common goals like losing weight or strength?  P3: I think just a bit uhm.  R: Something creative?  P3: Yeah, creative yeah.  R: Ok.  P3: I think because uhm yeah creative I like stuff that is fun because I find sometimes the gym so boring.  R: Yeah.  P3: Absolutely boring uhm doing that same exercise uhm I like the fact that the accessercise app gives you the option to be at home uhm and that’s the option I would choose because I don’t like going to the gym anymore because I’m scared that people look at me and stuff uhm so I like the fact that app is giving you the options to do physical activity at home uhm and outside.  R: Yeah, I think the issues with some exercise apps is that they are very much focused on the gym uhm and the problem with having a disability is that everything takes time uhm you need to get dressed which can take you a long time uhm you might have a carer come and help you uhm support you getting into the car and into the venue which can take like three or four hours uhm and then going to the gym doing everything uhm you’re talking about a whole day of things uhm so yeah if they could do these exercises at home with their carer uhm in their living room uhm it will save them a lot of money in terms of cost of petrol uhm and also their time uhm and they can just get on with their day so I think the focus of the app is to try and tackle barriers uhm but also try to encourage more people to do it because if we don’t develop these sort of apps then people will suffer uhm the NHS is going to under pressure with more health conditions to deal with uhm so whether we can actually do more at home would be better for these people you know.  P3: Yeah, yeah definitely!  R: I think because everyone likes their own space uhm their own home uhm so if they can do exercise uhm at home rather than a gym and getting changed in the gym uhm not have to go to the machines which is normally busy they can just do it at home would be really helpful.  P3: Yes, I really like how accessercise provide the option to exercise at home.  R: I really like the idea that they have the option to do physical activity at home.  P3: I couldn’t agree more, I like that too. The options of places to exercise is good.  R: Because I don’t know about your experience of using disability uhm physical activity apps but there is none that really exist uhm this is the first one so this is quite unique in it’s own way but I like the way that the developers have thought outside the box uhm so it’s not just going to the gym uhm you can do this at home uhm in terms of yoga and stuff like that uhm I think that is pretty good.  P3: Yeah, I think uhm doing exercise at home option on the accessercise app is really good uhm really good.  R: Yeah, uhm would you say if you were to do it during your role for your job you have the space to be able to do that uhm do you think you are in the correct place?  P3: Yeah, so at work I have uhm on my night shifts uhm a whole ship to myself so I can ugh run around uhm do whatever I want to do uhm exercise wise.  R: Ok.  P3: Uhm, I have noticed on the videos on the app that there are a lot of bands uhm so that means you will need a lot of bands to do those exercises uhm especially if you’re doing it at home so I now know that I will need to go and buy that uhm so maybe in the shop option on the accessercise app the developers could provide that equipment discounted or you know uhm I know that the different coloured bands mean different things uhm and stuff like that so.  R: So, do you think what you just mentioned uhm having a link to that equipment so users can buy it from the shop on accessercise?  P3: Yeah, even if that’s a link to amazon or something.  R: So, you think having like an amazon link on the shop for users to click and they can see the discounts?  P3: Yeah, so I’m on the gym bit here on the app but if I put the home option uhm.  R: So, I agree with you.  P3: I’m going through it now and I can see a girl in the video has a black band uhm resistance band uhm a blue resistance band uhm so now I’m going to think I need that.  R: Yeah.  P3: So.  R: Uhm, so whether the shop could provide that equipment uhm because I’m thinking what exactly can the shop provide you know uhm sometimes it’s nice to have a water bottle uhm or a t-shirt with ugh the accessercise logo on it so you feel a part of that group uhm a part of that identity uhm but also whether the shop could provide some of the equipment used within the videos uhm on the app in ugh the shop would be a good idea.  P3: Yes, linking equipment used in the video to the shop would be nice.  R: I think it’s great that you can do it at home but a very small amount of equipment you will need to buy.  P3: Yeah, the adapted equipment and option to workout at home will be nice.  R: Uhm, so whether there could be a link through the shop for that would be good,  P3: I think as well like uhm did I see something about nutrition on here as well?  R: Yeah, there’s nutrition yeah.  P3: There’s so much nutrition out there you don’t know what’s fake uhm what’s going to work for you uhm maybe some links as well uhm we all know that fresh chicken is good for you but we also know that some supermarkets pump it up full of water.  R: Yeah.  P3: So, it’s not actually that good for you.  R: Yeah.  P3: So, maybe having a link uhm that says yeah this is good stuff uhm even like a butcher I don’t know.  R: So, are you saying links to local butchers or are you saying link to local food?  P3: Yeah, even protein powders.  R: Ok.  P3: So, many protein powders are available but which ones are the best uhm I don’t know if it’s true but I never get anything from holland and barrett.  R: Right, ok.  P3: Because the ingredients are faulty uhm I would rather pay a lot more money and get really good stuff and I always have informed sports stuff.  R: Right, ok.  P3: That’s because it won’t come up in a drug test.  R: Right, ok.  P3: So, uhm things like that uhm but there is still so much stuff out there so if you go oh this company is really good because they have got scientific research behind it uhm this company is good uhm this company is good.  R: Yeah.  P3: Just to help you out.  R: So, do you think there is quite a lot of fake news uhm or fake information out there?  P3: Yeah.  R: Ok, that’s pretty good to hear your opinion on that uhm so what were your initial thoughts when you first started using the accessercise application uhm did anything stand out that you liked uhm so when you first logged on what caught your attention?  P3: Yeah, so when I first logged on I liked where it says what’s your disability.  R: Right, ok. So, you like how the app is very much impairment specific?  P3: Yeah, so mine is my disks uhm so even though you have got the spinal option uhm it’s quite bad spinal uhm I clicked the other option.  R: Ok.  P3: Uhm, so yeah maybe increasing all these uhm conditions uhm because disk degeneration and having no disks in your back uhm is common and it is a disability uhm so maybe having more common disability things on there.  R: Ok.  P3: Uhm, but also mental health uhm I find even really big things uhm this is why I said about the dance uhm the fun uhm because you are doing something fun and it makes you laugh uhm and you’re exercising as well.  R: Yeah, I agree with what you’re saying because I think at the moment uhm the developers are only focusing on physical disabilities but obviously mental health links in with your physical health so whether they could include you know uhm options for people with mental health conditions and so on.  P3: Yeah, including mental health onto the app could be a good development.  R: It could be something that they include.  P3: But I think as well uhm everybody that’s got a disability like uhm I mean if you’re born with a disability uhm that is what you’re used to but if you were fit and then one day you are not uhm it really does impact your mental health uhm it really really does.  R: Yeah.  P3: Uhm, and your confidence and I don’t care what anyone else says uhm it really does haha and it takes a lot to put them trainers on again and think you can do things.  R: Yeah, definitely it’s that motivation uhm you have lost that confidence to do it and so on.  P3: Yeah.  R: So, one of the features you like on the app is how it’s impairment filtered?  P3: Yeah, focusing om specific impairments on accessercise is really nice.  R: So, how you can select what impairment you have.  P3: Yeah, I like that uhm the app is impairment specific which is unique.  R: Ok, awesome uhm is there anything else that you really like about the accessercise app when you first saw it?  P3: Uhm, so yeah uhm I have yet to click onto the social group thing uhm I really like the explore section on the accessercise app.  R: Ok.  P3: Uhm, I like how you can find local gyms with the explore section uhm that’s a really useful feature uhm again I know it’s a lot of work for the app developers but putting these particular health shops or really good places where you can get your protein shakes uhm and stuff and say where they are uhm and the exact details.  R: Ok, so at the moment the app is very much focused on local gyms but do you think they could possibly include local sport clubs uhm and local sport events?  P3: Yeah, I think including sport clubs or events on the map would help.  R: Yeah.  P3: Yeah, uhm I think if the developers could add local sport clubs and event to the explore section that would be really good.  R: I think at the moment the app is only really focused on gyms but you know someone that doesn’t want to use a gym but wants to join a local club uhm where are the local clubs uhm whether that could be incorporated on that would be a good thing.  P3: Yeah, yeah that would be uhm really good uhm to have different clubs on it and for different ages uhm I think this is aimed at adults.  R: Yeah.  P3: Yeah, definitely adults uhm but yeah you know uhm you could even as a disabled individual uhm you can still box you can still go to a boxing club uhm you are still part of something uhm you can still use all your upper body.  R: I think one of the issues uhm which I mentioned earlier is down to a lack of advertisement uhm there’s also a lack of advertisement for what is available for people with disabilities so whether they can incorporate local opportunities onto the app and then help people understand what is available.  P3: So, I think that way this is where you could introduce like ambassadors.  R: Ok.  P3: Uhm, you could do that across different counties uhm so if like you was an ambassador for Loughborough then that gives you a sense of purpose as well uhm if you are disabled and you are an ambassador for this app uhm it gives you a sense of purpose that you want to help people as well so you probably can go off into your own little town uhm find all these clubs yourself and put them all on the app.  R: Yeah, that’s a good point.  P3: Uhm, because you are giving someone that role of responsibility and part of something and they feel like they are helping people.  R: I agree, that’s such a good point uhm and I think it’s good to have that but I think the only downfall of that is you don’t want the app to have too much fake information.  P3: Yeah.  R: Like you don’t want to include events that last for just one day uhm and they appear on the app for a long period of time because it then just becomes a bit confusing uhm so whether there could be a way that the events that they choose are actually valid and should be included onto the app.  P3: Yeah, I think that would be really good to have.  R: Yeah, makes sense. Uhm, does anything else stand out to you that you liked uhm so you liked the explore section?  P3: Uhm, yeah so when you see the exercises uhm you can just click a little button and like it uhm and add it to your favourites that’s great to have.  R: Ok.  P3: Yeah, I like that. I like the videos of all the different exercises uhm I like how it is clear and it is showing you what to do uhm and it is in colour.  R: Right, ok. So, what specifically about the videos do you like uhm do you like the fact that it’s someone with an impairment doing it?  P3: Uhm, yeah well yeah I suppose if someone was on there with like the same disk degeneration that I have uhm and they are able to do it that would make me think ok uhm because sometimes people like me can’t bend down to the floor uhm I can because I have had that operation.  R: Yeah.  P3: But if I can see someone there doing it I’m going to go oh look they have the same problem as me and they are doing planks.  R: Yeah, haha.  P3: That means I must be able to do it as well.  R: Ok, perfect.  P3: So, yeah, I can do it if I try.  R: Yeah, that makes sense that’s really good. Uhm, I don’t know if I showed you but there’s also an option that when you design your workouts you can then add it to the calendar so for example if you press calendar uhm so for example if you press the eleventh of May in two days uhm you can then add a workout.  P3: Oh, ok the option of adding workouts to the calendar seems brilliant.  R: If you wanted to do chest every Friday morning you can add the exact same workout every Friday morning uhm so it saves you time uhm having to plan.  P3: Yeah, I like that I will definitely be using the calendar function.  R3: That’s a good function to have.  P3: So, it helps you plan your day easily so if you wanted to do legs?  R: Yeah, because you can easily spend twenty minutes doing your whole weeks worth of workouts but then use those workouts everyday for the rest of the month uhm you can spend twenty minutes doing chest on a Tuesday uhm legs on a Wednesday uhm back on a Thursday uhm and design those workouts and design those workouts the way you want it and add them to everyday of the week for that month for example.  P3: Oh, alright ok yeah.  R: So, yeah, a little bit of planning will save you a lot of time in the future.  P3: Yeah, I like that.  R: Yeah, that’s pretty good uhm.  P3: Like ugh individual workout plans isn’t it.  R: Yeah.  P3: You can just look at the calendar and be like oh I have got to do that now.  R: Yeah, it saves you time because every Monday you do this uhm every Tuesday you do that uhm you don’t have to think too much uhm you just log onto the app uhm so today is Friday I need to do this and that uhm and it’s already laid out for you and you just get on with it which is pretty cool.  P3: Yeah.  R: Uhm, but yeah is there anything else that you want to mention that you liked when you first opened the app?  P3: Uhm, yeah I like uhm my workouts uhm that you can do and I like ugh the bit where you can go into the filters uhm I really like that uhm locations uhm choose a goal I really like that.  R: So, you like the filter option where you can select where you want to do it?  P3: Yeah, the option to filter and choose locations for workouts is fantastic.  R: Uhm, and obviously you can select your equipment and so on which is pretty good.  P3: Uhm, there is just one thing that is not on there uhm which is lose weight uhm so weight loss.  R: I think it comes under the section called toning.  P3: Oh is it?  R: I think what they are trying to do is to be careful on the terminology that they use.  P3: Oh, ok.  R: I think they want to avoid saying uhm losing weight because it makes people feel like oh I need to press this to lose weight.  P3: Right, ok.  R: So, I think with the word toning it seems like you are toning yourself up to be in better shape so I think they are trying to be ugh a bit sensitive on the information they use uhm and the words that they use.  P3: Ok.  R: So, for example things like mobility uhm they are providing ugh logos of someone who uses is a frame because someone that focuses on mobility may be someone that is elderly uhm so they are trying to focus on you know different ages but also making sure that the words that they use are appropriate uhm so they are not going to say like body building.  P3: Yeah.  R: Because that might give away the idea that you are just a meat head or whatever uhm but it is very much saying the word build strength which pretty much anyone can do.  P3: Yeah.  R: Because if you put body builder uhm someone that is eighty years old can’t be a body builder uhm but someone that is thirty probably can.  P3: Yeah, I understand.  R: So, at eighty years old you can still increase your strength uhm so the words that they use uhm they are trying to be inclusive for everyone.  P3: Ok.  R: Uhm, that’s perfect so you have mentioned a few uhm strengths of the app that you like so far uhm so the next question is do you have the required equipment to use accessercise in it’s full capacity so in other words uhm access to a mobile phone to use the application?  P3: Yep.  R: So, uhm do you have like a tablet at home that you can use?  P3: Yeah, I have got a tablet uhm the only thing that I think about with the question you asked me is uhm a lot of the exercises that I have looked at and picked uhm you need like rubber bands or as I call them resistance bands which I haven’t got which could be expensive uhm money I haven’t really got.  R: Right, ok.  P3: So.  R: I guess that is one thing that in the shop function maybe on the app uhm they could provide.  P3: Yeah, if the shop could link cheap alternative equipment that would be good.  R: Yeah.  P3: Yeah.  R: Because I think when it comes to doing any sort of exercise uhm some of them you will be able to do free of charge uhm but some of them like increasing strength obviously you need equipment that is quite heavy which is likely going to be something that costs so I think the best thing they can do in that situation is just to provide links for you to do the exercises at home  P3: I think they can provide them in the shop because like I said there’s so much stuff out there uhm there will be loads of resistance bands out there but they might snap because they are cheap uhm it’s having that recommendation uhm that these are good resistance bands uhm they are used in the NHS or they are used in the gym uhm you know that kind of thing so you know that you are buying quality stuff.  R: But I guess the equipment that you were to use at home is not too expensive like a rubber band will cost you max three or four pound if not five pound.  P3: Yeah, but some equipment here seems like it will be expensive.  R: Uhm, compared to going to the gym uhm with the racks of weight which cost hundreds of pounds uhm but actually some of the stuff you can do at home is a lot cheaper then buying the membership from the gym.  P3: Yeah, I agree accessercise will definitely be cheaper than gym membership.  R: So, I think what the app developers could uhm do better is linking equipment to the shops so it’s easier for people to access that uhm so whether that is like a catalogue uhm where you can just search uhm rubber bands and it comes up with a list of cheaper alternatives.  P3: Yeah, the shop should definitely provide a link to cheap alternative items.  R: Uhm and you just pay for it and it gets sent to your address or it could be sent to the address that you used when you registered your account or whatever.  P3: Yeah, that would.  R: For example, if you signed up for the membership it has obviously already got your card details uhm so it’s automatically on the system uhm with your card and you can just pay for the rubber bands.  P3: Yeah.  R: So, those could be things that save people a lot of time because what you don’t want to do is uhm you see that people need rubber bands uhm they have to go out there and search for it themselves on amazon or ebay but actually could the app developers provide a link or a catalogue.  P3: Yeah, I think that would be better if they could provide it through a link.  R: Whether that’s just an accessercise shop where it pops up with their logo uhm what equipment do you need and they can some how work with some people to provide that equipment uhm because yeah the rubber bands would be good uhm but what exactly is the size of the rubber bands uhm what’s the name of the rubber bands uhm do I need a certain colour like you know uhm what does it mean uhm what is a rubber band and so on uhm so providing those links would be pretty useful.  P3: Yep.  R: Uhm, so the next question I have got is are there any improvements that you believe could help improve the accessercise application uhm so obviously you have seen the features now.  P3: Yeah, only the ones that I have mentioned really ugh like linking equipment that you need for the exercises to the shop uhm especially if you want to do exercise at home uhm maybe having a bit of fun things on there.  R: Ok, uhm how about the layout of the app uhm so obviously you have all the bits at the bottom.  P3: Yeah, the layout of the app is good they have done a good job.  R: Do you think it’s quite easy to get around the app?  P3: Yeah, it is really easy to learn how to use this app because it’s made simple.  R: And in terms of maybe uhm the exercises available do you think uhm maybe categorising things is easier?  P3: Uhm, well I don’t know ugh for example I just used the filter option and only two things have come up.  R: Right, ok.  P3: But I think it’s because I’m on your account.  R: Yeah, I think it comes down to filtering and selecting the right stuff.  P3: Uhm, so I found that quite easy to do uhm all the filtering for workouts.  R: Uhm, but in terms of obviously the awards and you mentioned earlier about motivation ugh and finding ways to motivate yourself uhm what specifically would you want from a motivation side on the app?  P3: Me personally uhm what would motivate me is having like google points where you get ugh like money for completing things.  R: Right, ok.  P3: Uhm, because I’m on ugh like google opinion rewards which uhm they send me like a survey once a week but I will get a range from like five pence to twenty pence uhm and it just stays in like my little wallet and I can then go and buy uhm games and stuff like that so uhm things like that would be good uhm because I do think like sixty pounds for the membership on the accessercise app is quite expensive especially as loads of functions are not available yet uhm so I personally don’t think I would spend that so the maximum I would spend is around forty pound uhm but once you’re on the app if you can get like these little ten pence uhm four pence whatever rewards you can get would be good.  R: Uhm, so are you saying from doing a certain amount of exercise you will be rewarded an incentive?  P3: Yeah, so then when your membership comes around again you have got these little money tokens which you can use to make the membership price cheaper.  R: Oh, ok so like discounts?  P3: Yeah, discounts on the app would be helpful uhm yeah definitely.  R: Oh, ok.  P3: Yeah.  R: Uhm, how about like competitions with other users uhm would you like the idea of having leader boards and you know like a streak or something?  P3: Uhm, for me that wouldn’t bother me.  R: Oh, ok so even in terms of streaks so if you were notified that you have used the app for five days uhm you have to continue using the app or you will lose your streak do you think that might motivate you uhm so an example of that is if you have used the app every day for a year uhm so you are on three hundred and sixty five days.  P3: Oh, yeah that would help because that would annoy me if I lost my streak.  R: I think like the fact that you uhm do not want to lose that streak because you have worked so hard for it could be something that they add.  P3: Yeah, streaks are a good idea to maintain motivation.  R: Uhm, how about daily uhm challenges so you know uhm you do a thousand steps today you might be given a point on a leader board with other users and stuff like that uhm would that interest you?  P3: Ugh, yeah it would if it was easy and it was perhaps connected to my watch.  R: Oh, ok.  P3: So, it automatically updated everything uhm because I would not want to physically go oh yeah I have done two miles today uhm I would not want to type that all in.  R: So, you would not want to type that in uhm you would want the app to automatically do that?  P3: Yeah, so it can connect to my watch or my phone uhm connect to my steps on my phone.  R: Yeah.  P3: Uhm, that kind of thing.  R: Perfect, ok and from the social media section uhm would you want anything else to be added to that uhm so you have mentioned about you know uhm following people and liking and reacting to their posts.  P3: Uhm, where is that under?  R: It’s under the social section.  P3: Oh, social uhm and it’s there isn’t it in front of me uhm so yeah I kind of like that so if that girl there was the same as me I would uhm quite like that because I would like oh right she has just done that.  R: So, you would feel a bit connected to her?  P3: Yeah, yeah if someone on there was the same as me I would feel a bit of a bond because then there’s the ability to question uhm put things on so I could write did you uhm hurt after that exercise.  R: Yeah.  P3: And that would be nice uhm for my confidence.  R: So, you would feel a bit related to her?  P3: Yeah.  R: I guess you would feel related to her and so on.  P3: Exactly.  R: How about the group section uhm did you like how you can like join groups?  P3: Uhm, so finding new groups uhm it’s not working but uhm yeah.  R: As you saw, there was like cerebral palsy groups uhm spinal cord injury groups.  P3: Yeah, yeah so again uhm that would like motivate me because I would feel part of a group uhm so that’s really interesting because people can put on there like oh I have done this uhm I’m trying this drug out and stuff like that uhm I think that’s really nice.  R: Yeah.  P3: Maybe people will think oh that’s really good where did you get that from?  R: Haha.  P3: How much is it uhm you know things.  R: That makes sense.  P3: So, yeah having a group that is the same as you is really important.  R: Ok, and how about ways to develop that option further uhm would you like to develop your own groups for example?  P3: Uhm.  R: So, for example.  P3: Oh, this is where it gets hard uhm so yeah I would like to do that uhm I would like to put on there uhm people with disk degeneration uhm or spinal cord injury or whatever uhm but then you might put something slightly different and then someone else does the same and then you have got loads of groups and you are then constantly searching uhm that would be really annoying.  R: Right.  P3: That is the only dangerous part of uhm allowing everybody to open a group up.  R: Right, ok.  P3: It’s then going to have loads of groups.  R: So, whether you let the developers know uhm you want to set up a particular group and whether they agree with you uhm you know.  P3: Yeah, I think that would be good uhm.  R: So, you need their permission first.  P3: Yeah, so go through them first so perhaps emailing them saying you know I have this idea and whether they say that is something for the future uhm and maybe they will say yeah we can do that uhm I think it needs to go through them.  R: Yeah.  P3: Just so you stop the overwhelming.  R: Stop people going crazy haha.  P3: Yeah, like Facebook uhm there are tons of groups uhm so if I typed in on Facebook spinal injury groups uhm like twenty will come up and I am then like which one do I pick.  R: Yeah, there’s too much choice haha.  P3: Yeah, there’s too much choice yeah.  R: Within the groups would like them to just be community groups where you can talk about your impairment or would you like it to be focused on gym stuff?  P3: Uhm, where you talk about yourself uhm I think that’s uhm a good idea because when I uhm was going through my injury I was on loads of forums which I was asking people what they had done uhm what has helped you uhm absolutely loads of forums.  R: Right, ok.  P3: Uhm the spinal operation that I have had done uhm nobody has had I think there is like four people in the UK that has had it.  R: Right, ok.  P3: I would love to be able to find those people.  R: Is there no way you can find out who they are or uhm find a way to contact them?  P3: No because it’s like confidential.  R: Yeah, uhm that makes sense because I guess your experience is different to theirs and they might think you know uhm I don’t want to give too much information away uhm I may get in trouble.  P3: Yeah, that’s the thing uhm this is where uhm if somebody like me came on like a group that we were both just on uhm disk degeneration group or something like that and they say oh it’s getting worse and worse uhm I can then say oh I have had this operation uhm look for that uhm it gives people hope.  R: It gives them encouragement.  P3: Yeah.  R: I think that is sometimes what people need but obviously the world is changing and you have to be careful what you say.  P3: No, that’s it.  R: If we could that would be awesome.  P3: Yeah.  R: Uhm, the next question I have got is uhm have you used any other similar applications outside of accessercise if so what is better or worse than accessercise?  P3: Uhm, I have only used that dance app that I mentioned earlier.  R: Ok.  P3: Uhm, actually I haven’t really used that.  R: How about previous ones uhm have you used anything else before?  P3: Uhm, yeah I have used one which I believe is called rehab.  R: But have any physios or anyone else recommended any for you to use?  P3: Oh, yeah the NHS uhm particularly the doctors they constantly signpost you to the NHS feel good website.  R: Ok.  P3: Uhm, that’s a load of crap for me uhm I don’t like it.  R: Right, ok.  P3: I like being unique I like uhm doing things that no one else can have.  R: Right, ok haha.  P3: So.  R: You want to be different?  P3: Yeah, I want something that is for me uhm for my age as well so I’m forty plus so obviously my hormones are going to take effect on my exercises so on that app uhm this exercise app maybe they need to take consideration of women’s hormones.  R: Ok.  P3: And that we are going to find it a little bit harder if we are going through hormonal problems.  R: So, going back to where you design the workouts would you like the exercises to be graded in difficulty levels uhm so in terms of easy uhm moderate ugh difficult and then even harder?  P3: Yeah, I like how you can select your difficulty.  R: Ok, because I think at the moment uhm they do have that option where you can select uhm easy ugh moderate and so on.  P3: Yeah, they do.  R: Uhm, whether that could be laid out in a bit more of an easier way uhm so once you have done a workout uhm it then says uhm more difficult workout or harder workout so you are more challenged.  P3: Yeah.  R: I think what people don’t want to be doing is uhm using the app it’s really easy and then they don’t use it again because nothing is challenging for them so whether there could be an option for them to do more difficult exercises is important.  P3: Yes, I think they should have the option to do that more difficult exercises.  R: Yeah, but from this app that you have used and also the previous app is there anything that you find a bit different uhm do you find that maybe something that the previous app was better at compared to this app that maybe could be added onto this app?  P3: So, the two apps that I have had uhm so one was just for me.  R: Ok.  P3: Uhm, so personal to me which I love because it’s exactly the same as this app where it has got videos uhm it tells you what to do uhm it tells you how many reps and stuff like that and I know that is personal to me uhm and the one that I have downloaded recently is the dance app thinking that I’m going to use it a lot but I haven’t.  R: Haha, ok.  P3: Uhm, I like dancing uhm I like pratting around I like fun uhm I like laughing and I think dancing allows you to do that.  R: Right, ok.  P3: But obviously that is not injury specific so they don’t care that I am jumping around and hurting my back.  R: Yeah.  P3: So, I find that a bit difficult because I’m constantly going through the whole app thinking uhm what will suit me uhm what will suit me.  R: Yeah.  P3: Uhm, and there’s too much choice on that app uhm there’s just too much on there.  R: It’s a bit overwhelming?  P3: Yeah, and there’s too much colour uhm and it’s very much in your face uhm whereas this accessercise app uhm has a nice colour appearance.  R: So, in terms of colours on the app.  P3: I think the accessercise app is very basic in terms of the colours they have used but it’s good because you don’t want it all too much I don’t think you don’t want it too bright and in your face uhm.  R: So, do you think like ugh the size of the text uhm the visuals are ok?  P3: Yeah, yeah it’s brilliant and it’s very uhm it looks medical uhm it looks very medical yeah uhm so yeah I like it uhm it’s very simple uhm it’s very easy to navigate uhm and it’s very easy to search for the exercises that I want which is really good uhm because some other apps are really hard.  R: Yeah, I have seen some previous apps and yeah I know what you mean it’s quite overwhelming isn’t it.  P3: Yeah.  R: Uhm, and you think oh gosh I have got all of this going on it’s got that uhm it’s got that.  P3: Yeah, that’s it.  R: Accessercise very much has the four options.  P3: And I do like the bit that says legs uhm no equipment uhm arms you know.  R: Yeah, because I think it gives you an idea of what that workout is based on so whether that is legs uhm chest uhm you can quickly see that and be like ok today I’m working on legs.  P3: So, straight away if I type in overall body and running came up uhm I know that I can’t run.  R: Yeah.  P3: So, that would be like right great uhm they have got nothing for me.  R: Yeah, do you like how the app very much focuses on specific muscle components?  P3: Yeah, I do and to your injury as well so I like how you can filter the app based on your impairment uhm so choosing your disability.  R: Right, ok so we will now move onto the main bit so we are now going to move onto the main bit of this study and ask you to participate in a think aloud protocol while using the accessercise application uhm so the instructions for a think aloud protocol are straight forward uhm I would like you to say out loud whatever comes into your mind there are not right or wrong answers we will just run through a quick practice to get you used to the protocol so could I get you to say out loud everything that comes to your mind when you think about physical activity?  P3: How on earth.  R: Haha.  P3: How on earth uhm that’s what comes to mind.  R: But in terms of like easy, difficult?  P3: Uhm.  R: What words come to your mind when you think about exercise do you think?  P3: Uhm, so I would say fear.  R: Fear?  P3: Yeah.  R: How about pain?  P3: Yeah, fear uhm pain uhm what am I going to be like tomorrow.  R: How about enjoyment?  P3: There is no enjoyment there no.  R: Haha, so you find it quite challenging?  P3: Yeah.  R: Right, that’s quite interesting. Uhm, so I would like you to share your screen with me uhm and open up the accessercise application uhm so you know we went onto the app uhm I showed you around uhm what comes to your mind uhm so the first impressions when you used the app uhm would you say you are satisfied with what you have seen?  P3: Easy.  R: Ok.  P3: Yeah, very easy to use.  R: So, basic uhm not too difficult uhm the colours are ok.  P3: The colours on the app are fine uhm yeah the wording is fine uhm the size of the writing is fine.  R: Perfect.  P3: When you click on the filters ugh they are easy.  R: Ok.  P3: Again, there is not too much to choose from.  R: So, you think that the way they have laid out the filter section is good uhm there is not too much information?  P3: It’s alright yeah not too much information.  R: Ok.  P3: It’s good yeah a nice balance of information it’s not overwhelming.  R: Do you think for example the text ugh is ok uhm the colours of the text are ok?  P3: Uhm.  R: I think that it’s quite simple to use as well.  P3: Yeah, I think it’s all fine uhm the text.  R: What do you think about the logo next to each one uhm so you have got like uhm I don’t know what that is uhm it’s be fit with like a t-shirt uhm getting tone is obvious uhm the bikini uhm increasing strength is obviously uhm a medicine ball.  P3: Yeah, fine.  R: Do you think it’s good to have those little logos there?  P3: Uhm, so mobility I don’t know what that means uhm to me that only looks like it’s good for old people.  R: I think mobility is more about walking and moving so whether that is walking up and down uhm in your kitchen.  P3: Right, ok.  R: Uhm, yeah, I guess they could maybe define what they mean by that uhm so whether flexibility and mobility could be defined you know.  P3: Uhm, yeah because now I’m confused on what does be fit mean uhm I get what toned means uhm I get what be healthy means uhm but you’re going to be healthy in doing all of them.  R: Yeah.  P3: If you do it uhm yoga that’s simple ugh stretching that’s simple ugh cardio is simple ugh flexibility is around moving which is good.  R: Yeah, that’s a good point because yeah I think be fit uhm be healthy is the same as cardio isn’t it but being healthy is the same as doing yoga uhm and increasing strength because they are all increasing your heart rate.  P3: Yeah, these two uhm I think if you do all of this uhm you are doing that.  R: So, it would be good to see whether they could incorporate some of that together.  P3: Yeah, incorporate some of the goals together to reduce information.  R: Uhm, because I’m thinking do you really need to have be healthy uhm because isn’t being healthy uhm all of those goals.  P3: I think being healthy uhm is nutrition uhm food so not eating chocolates.  R: Yeah.  P3: So, maybe if you clicked that uhm I don’t know uhm I think being healthy I see it as good uhm nutrition.  R: Ok.  P3: Not fitness.  R: Yeah.  P3: Uhm, because if you do all of this uhm you are going to be healthy uhm and you will be fit.  R: Yeah.  P3: Yeah, I agree with you uhm I don’t know whether they need that option there uhm I’m not sure why.  R: But you like the location setting so how it’s got the gym uhm home.  P3: Yeah, the option to choose where to do your workouts is good.  R: Do you think there is anything else they could provide uhm for the location setting uhm so maybe something like at work?  P3: Uhm, I would say that would be at home anyway.  R: Ok.  P3: Yeah, I sometimes complete work at home.  R: So, the fact that at the moment there is just three uhm keeps the options simple doesn’t it?  P3: Yeah, because like if I was at work I would just click on the home button.  R: Ok, I guess work is done at home.  P3: Yeah, yeah that’s it.  R: Perfect, ok and if you were to scroll down ugh so obviously here you have got the muscle groups uhm do you like that or do you think they could add a bit more creativity with that?  P3: Uhm.  R: Could they potentially provide a logo of what the back looks like uhm because I think it’s good to have the location ugh the goals.  P3: Yeah, yeah, I think the muscle groups ugh I think maybe I mean uhm I know what latts are and I know what your core bit is and all of that but I don’t know if someone that hasn’t gone to the gym before would know what they are.  R: Right, ok uhm so I think.  P3: Uhm, maybe a picture with an arrow.  R: Ok, just to show them which muscle groups they are targeting?  P3: Yeah.  R: I think at the moment uhm the first bit looks really interesting and then it gets a bit boring with this bit because it’s like uhm legs ugh back ugh chest uhm could they not provide a bit more information there.  P3: Yeah, uhm.  R: We know what back is but how about shoulders uhm what does core mean uhm what does cardio mean you know?  P3: Yeah.  R: Could they eventually provide something there that could be useful I think is quite important.  P3: Yeah, maybe.  R: I think that’s important.  P3: So, with core as well uhm a lot of people think it’s just your tummy but it isn’t it’s your whole frame.  R: Whole frame?  P3: Yeah, frame isn’t it ugh your core.  R: Uhm, so when I think of core I think more of your abdominal area and stuff like that but I guess there’s more to it isn’t there.  P3: Uhm.  R: But also easy could they provide like uhm I don’t know percentages so one hundred percent is really hard uhm easy is like twenty five percent uhm just to encourage people because if you see different colours and a progress log.  P3: Yeah, so you see uhm when I see the easy option on here I wouldn’t even click easy uhm.  R: Why is that uhm is it because you find it too easy?  P3: Yeah.  R: Ok.  P3: Light to me uhm does it mean that there is light weight to it or?  R: I think it means that the intensity is quite light so it’s not hard.  P3: So, when intense to me is yeah really really hard.  R: So, maybe they could bring some of them together uhm so instead of having like five options.  P3: Yeah.  R: Could they potentially just do moderate uhm hard and intense or something like that?  P3: Yeah, easy, moderate, hard and intense uhm I just don’t really understand what the light means.  R: No.  P3: I just think that light is easy.  R: Yeah, that’s exactly what I thought so maybe instead of having five uhm having three so it’s easy to press.  P3: Yeah.  R: It’s less information to read which is important uhm how about exercise ugh requiring an assistance or a carer uhm what do you think could be added to that uhm that you would like to see?  P3: Uhm, so exercise that is requiring an assistant or carer would be I think they are kind of exercises like if you are going to do chest press uhm you need someone on there with you uhm spotting.  R: So, do you think there is anything that the gyms could to work with the app to be able to provide that support in the gym uhm so could they notified or something like that before the person comes so they are aware that a user may need assistant uhm because normally when I go to the gym I never normally see anyone working there.  P3: No, well this is it and the gym is not going to be paying someone to provide.  R: Support for someone?  P3: Support for one person.  R: Yeah, I know what you mean.  P3: Uhm, that is when you pay for your personal trainers and stuff so I think this is going to be common sense really uhm exercise requiring a carer.  R: So, whether the care plan of that individual could bring a carer with them.  P3: Yeah, so like people in supported living it would be that carer that decides anyway if that person can be left alone.  R: Yeah.  P3: Or do they need to stay.  R: Yeah, that makes sense.  P3: So, I don’t think that’s really required because it is all common sense.  R: Yeah, makes sense uhm it would be good to see what they provide for that you know uhm whether there’s anything interesting that is going to be added soon but if you were to press like outdoor training for example uhm just down the bottom.  P3: Uhm, this one?  R: Yeah, uhm so for example here you have like uhm three three three.  P3: Well, outdoor training would be no equipment uhm why would you have a hula hoop outdoor uhm why would you have a yoga block outdoors uhm why would you have dumbbells outdoors?  R: I think it means whether you’re outside working out with friends uhm so whether you’re doing a group activity outdoors with some friends and you are using a hula hoop.  P3: I get this uhm the prosthetic leg being outdoors because obviously you don’t want to be on an uneven ground uhm.  R: It’s quite confusing right?  P3: Yeah.  R: But do you like the way that they have laid it out so uhm it’s gone from like three three three four three one or do you think maybe they could put it in alphabetical order or something like that?  P3: Uhm, no I like that uhm I like that it’s alright ugh the layout is good.  R: Yeah.  P3: It seems all good to me the structure of everything.  R: Perfect, ok so you think the grey colour there is ok?  P3: See at first uhm when I did click onto it I thought all of these were highlighted anyway.  R: Right, ok.  P3: And then I realised that you press it and pick uhm but I initially thought they were all ready selected.  R: Right, ok so you got a bit confused with that?  P3: Yeah, I got confused.  R: I got confused with that at first as well haha.  P3: Yeah, and then I realised that you need to press them to select what you want.  R: Yeah, uhm ok so we will move of this section uhm you will now need to swipe down.  P3: Is that it yeah?  R: Yeah, and then you have got home exercises so obviously home based equipment uhm but you don’t think they could put these in alphabetical order or anything like that uhm you think it’s ok uhm because I find that when I read through that uhm I’m looking through trying to find certain idea uhm but whether they could do that in alphabetical order would be pretty good.  P3: Yeah, I suppose alphabetical order would be good uhm I mean home exercises are you really uhm going to have a TRX at home.  R: Yeah.  P3: Like where would you connect that.  R: So, whether some of that could be put into a different category you know.  P3: That is more for an outside one there.  R: Yeah, so maybe some of the categories are linked in the wrong places uhm so that is something that they could work on.  P3: Yeah, I guess they could just copy and paste it I think.  R: Yeah, it would be quite interesting to see uhm so if you were to go back onto the home page ugh so that’s the filter section uhm but yeah if you were to press on let’s say banded upper row uhm ok it’s frozen haha so if you were to go to exercises and do my workouts.  P3: It’s not working.  R: Uhm, and then press one of those ones and then press on the exercise at the top uhm sorry if you were just to scroll back and maybe go to my exercises at the top uhm so my exercises uhm and you were to press one.  P3: Just press one?  R: Yeah, uhm just press this one here ok uhm so for example here the descriptions do you think there is anymore that they could do with the descriptions here uhm do you think maybe they could lay it out ugh a bit better uhm or do you think it’s quite organised?  P3: Uhm, do they talk?  R: No, so some do not talk I believe which is the only down fall.  P3: Uhm, I think maybe sometimes ugh oh they are talking uhm I think if they talk would be good.  R: I think some videos they do have voice overs uhm and some don’t.  P3: Voice over yeah.  R: Yeah, voice overs would be a good thing to have.  P3: Yeah, that’s good having voice overs in the videos.  R: You like the videos?  P3: Yeah, any fitness app that includes videos is good.  R: Awesome.  P3: Yeah.  R: So, if we were just to go back onto the main bit uhm so you have got the videos uhm you have got the descriptions but do you think maybe they could lay it out in a bit of a creative way?  P3: Bullet points for the descriptions of each exercise could help improve the layout.  R: Yeah, I find the key benefits.  P3: I like the pictures of the muscle components being worked.  R: You like the photo of that?  P3: Yeah, the photo of the muscle component is fine.  R: Ok, and how about the useful tip at the bottom?  P3: Yeah, that’s good uhm the useful tip uhm so try heading out to the park so I now know that I can go outside and do that.  R: Right, ok. Perfect, ok so that’s all the exercises done so we will just finish up on the last sort of bits uhm so you have obviously got the exercises uhm how about notifications would you like to be notified regularly on when to do exercise or a vibration on your phone?  P3: Uhm, me personally no I don’t like notifications.  R: Haha, you don’t want to be pressured.  P3: It would annoy me.  R: Ok, so if we were just to go on explore uhm so the final section.  P3: Which one is that?  R: That’s the one. Uhm, so you have seen this function so if you were just to press one of them so obviously it gives you the name uhm it’s powered by Google it tells you the opening hours uhm their email address and so on but do you think there is anything that the gyms could do to make this function better on this uhm so whether that’s videos or photos of the gym uhm so whether there’s images of that gym before you use it so you can see what it’s like?  P3: Yeah, if I was a business owner and I owned that gym and I knew my gym was on an app I would want to provide as much information on there so I would put the emphasis on myself as the manager or the owner to get my product on that.  R: Right, ok uhm but how would you go around doing that uhm what would you as the owner do?  P3: Uhm, yeah so on this explore section on this app you’re showing me I would definitely include a bit on car parking and whether there’s accessible parking in the area uhm so I would put up pictures of that on this app.  R: It’s funny that you say that uhm because I have had a few people say that actually.  P3: Yeah.  R: They said whether on the map it could show you parking opportunities.  P3: Yeah, car parking spots.  R: Because in terms of accessibility you know uhm the gyms may be accessible but how is the parking outside.  P3: I normally turn things down if there is no parking.  R: Oh, really?  P3: Yeah.  R: Ok, so you think having an option on there that shows whether the parking outside that gym is accessible?  P3: Yeah, so maybe like paid carparking.  R: Yeah, that’s pretty good.  P3: Or free parking uhm for one hundred cars so you know.  R: You know what’s around you.  P3: Yeah, uhm and maybe a picture of the swimming pool if it has got one in there uhm a picture of the car park so you know where you are going.  R: Ok, I think that’s a valid point uhm how about like a free sixty rotation video of the gym?  P3: Uhm, yeah so if it’s powered by Google uhm if you are able to click the gym and it takes you into the Google Street view thing uhm yeah so you can have a look around and stuff would be good.  R: Ok, that’s awesome uhm and then you have obviously go the gyms email address so you can email them if you have any questions you have got uhm you have the phone number uhm how about the actual accessibility rating uhm so you can rate this gym uhm but what specifically would you want to be rating the gym on uhm what would you want users to see in terms of accessibility?  P3: Uhm.  R: Because when I think about accessibility I think of so many different things uhm I think of uhm.  P3: Uhm, so for me uhm I hate people watching me.  R: Ok.  P3: So, I would love to go swimming more but I know nearly every swimming pool has got ugh a gym up the top uhm and all the runners watch you.  R: Right, ok.  P3: That puts me off.  R: That puts you off?  P3: Yeah, so I won’t go.  R: Right, ok.  P3: So, uhm I would like to know that.  R: Right, ok so I guess that could be included within the videos uhm and the three sixty rotation clip?  P3: Yeah, within the videos uhm and yeah within the three sixty rotations.  R: But in terms of the actual rating system uhm with the starts uhm would you want that to be broken down into smaller chunks so you can see what is accessible?  P3: Uhm.  R: So, what I mean by that is uhm ok you have accessibility five stars but that gets broken down into equipment uhm facilities uhm staff you know parking uhm so you can see the rating for each of those ones.  P3: Yeah, yeah.  R: I think at the moment if it’s five stars I’m in a wheelchair uhm you have got an amputee my experience of accessibility is completely different to yours.  P3: Yeah.  R: So, could they make it clearer on that what they mean by accessibility I think is important.  P3: Yeah, yeah so like yeah having a ramp and lifts.  R: Yeah.  P3: Yeah, that would be important.  R: How about whether users can actually see the reviews of what people have said uhm because at the moment it’s quite hidden.  P3: Yep, reviews uhm it’s important that users can see each of the reviews that’s really important uhm.  R: Yeah, because I think at the moment you can see the information about the gym uhm but you can’t see exactly what makes that gym accessible uhm and what people have said about that gym uhm it’s sort of like they are hiding what the feedback is uhm so whether people could actually see what has been said uhm so for example when you buy a product you want to see a little bit about what people have said.  P3: Yeah, you want to see the reviews uhm I think it’s important to see the reviews.  R: Yeah, uhm so at the moment you see three in orange and then you see a muscle logo in white uhm do you think maybe having a key of what each of those mean uhm because I think this gym is a gym but it hasn’t been rated yet uhm and sometimes you may see the accessercise logo which means it has been rated so uhm this one hasn’t been rated.  P3: Yeah.  R: So, maybe having a key uhm so orange means two nearby uhm this one means ugh.  P3: Uhm, no because if I lived there I would just go around and be like oh there’s a gym there and then if it has got like review ten and accessibility then I would click more.  R: Ok.  P3: I would click on it and look for more information.  R: So, you would click on it?  P3: Yeah, to get more information.  R: Right, ok and do you like the fact that you can obviously zoom in and zoom out but also have the search bar where you can easily search.  P3: Yeah.  R: So, you don’t need to keep zooming in all the time.  P3: No, no yeah you can put where you live or where you are visiting so yeah I think that is really good.  R: Yeah and you mentioned about having sport clubs and you know sport opportunities.  P3: Yeah, I agree with sport clubs because not everyone is into the gym are they uhm.  R: So, you would like if they could provide that sort of service?  P3: Yeah, more options for different sports clubs and activities.  R: Yeah, I agree with you I think that would be good uhm perfect ok so we will just go onto the last bit which is the more option.  P3: Yeah.  R: I think it’s just the bottom right so you mentioned about the shops and having you know links to equipment uhm but is there anything that you like or dislike about the shop that you think maybe could be included probably?  P3: No, just the equipment like providing resistant bands uhm and then maybe like black resistance band uhm I think is the hardest isn’t it.  R: Yeah.  P3: And to put on there hard and then some information on why you would use that uhm why would you use hard sort of thing.  R: Ok, perfect and how about nutrition uhm you mentioned about meal plans and so on?  P3: Yeah, so nutrition I think uhm because if you are really serious about your fitness uhm your nutrition should be spot on as well so.  R: Do you think having the education videos on there you know uhm demonstrating to people what nutrition is?  P3: There’s so much stuff out there regarding nutrition I think it’s not a problem to get it uhm it’s what to buy so uhm like vitamins for instance uhm there are a lot of people that think they need to have vitamins uhm but some vitamins are dodgy uhm and they have no goodness in them.  R: Right, ok.  P3: Ugh, so maybe like including good quality nutritional products uhm so like that uhm company in Nottingham muscle something ugh they deliver fresh meat.  R: Right, ok.  P3: But it’s meant to not be pumped with water or anything.  R: So, it’s fresh and nutritious?  P3: Yeah, yeah it’s not a bad price either and they deliver it to your door.  R: Yeah, that’s pretty helpful.  P3: So, maybe someone could do some research into that uhm it’s a big company so uhm yeah they have good scientific meat available uhm and not from a dodgy company and stuff like that uhm it’s good I would like to put my name on that.  R: Right, ok.  P3: Uhm, so maybe stuff like that and your protein powders as well uhm again there are so many proteins out there.  R: So, you could link the nutrition into the shop as well so you can work together.  P3: Yeah.  R: I think that would be pretty good uhm so for example in the shop you could have a shop which has nutrition in there but also keeping the nutrition there where they can educate and help people uhm this is what a protein is uhm this is what a carbohydrate is uhm this is what your five a day looks like.  P3: Yeah.  R: But also having the shop sell the products that they are educating users on would be great.  P3: Yeah, but also why would you have protein powder uhm so maybe like a little snippet of protein powder is something that helps build your muscles or uhm because sometimes I take protein powder because I know that I certainly don’t get enough protein in a day uhm so even though it’s got sugar in it uhm I will have it because it has got that protein in there which will help.  R: Yeah, that makes sense.  P3: Uhm.  R: How about blogs and podcasts uhm what would you like to see from those functions?  P3: I never look really at blogs and podcasts uhm I’m not really into them uhm I’m not that interested.  R: Haha, ok.  P3: It’s not really my thing.  R: Ok, and then you have got account settings so obviously that is about membership and you know payments.  P3: So, yeah that’s all good uhm I think the membership is reasonably priced.  R: You like all that?  P3: Yeah.  R: You have also got manage subscriptions uhm but you like the colour here?  P3: I think with the subscription bit should have the option to pause uhm so if I know that my subscription is coming out next month on the first of June but I haven’t got any money then I would like the option to just pause it for a month.  R: Right, ok.  P3: Uhm.  R: So, you would like to pause your membership?  P3: Yeah uhm but keep everything there uhm keep all of it there uhm but pause it but give an option to cancel as well so again if I think right uhm I have lost my job I’m definitely not going to be able to afford three months of membership uhm when it comes out.  R: Yeah, so you would like the option to cancel.  P3: Yeah, pause and cancel.  R: Right, ok that’s pretty cool.  P3: Yeah.  R: That’s good to hear uhm so you have the option to continue for free uhm but yeah so you have got those options.  P3: Yeah.  R: But uhm whether they could order all of that better would be good uhm so why I have one three twelve then six in different places.  P3: Oh, right yeah.  R: It should be one three six twelve.  P3: Yeah, so maybe they could sort that out.  R: Yeah, so that’s probably something that they could work on you know uhm but I think the visuals is pretty good uhm it’s easy on your eyes.  P3: Yeah.  R: Easy to understand.  P3: Yeah, it just needs that option to cancel or pause it in case you have financial difficulties.  R: You have got change password which is fine uhm you can easily change your password.  P3: Yeah.  R: Uhm, you have got privacy settings where you can select what you have on your profile and so on.  P3: Yeah, the privacy settings are good to have.  R: Perfect, ok so we will move onto the final ugh over thoughts uhm so the first question that I have got is what are the potential positives if there are any of using accessercise for improving physical activity levels uhm so what do you think are the overall strengths of using this app?  P3: The accessercise app has good videos and pictures and it is giving you the option to do it at home and that is going to help people’s confidence to be able to do some exercise at home because not everyone wants to go to the gym uhm I like it that if you do go to the gym you can take your phone out as long as you have got Wi-Fi uhm maybe could there be an option to download uhm download the exercise plans.  R: Ok.  P3: So, if you are outside in a park that you can still do your exercises uhm you don’t need to use your data.  R: Right, ok.  P3: And stuff.  R: So, similar to music uhm you can download it.  P3: Yeah, yeah so maybe having that option.  R: Ok, uhm, and I guess that also reduces the barriers of travelling uhm you know ugh how about the social network do you think it’s good to have that?  P3: Yeah, I think ugh it’s got to try out social things uhm like I said before it’s nice to follow people that are like you.  R: Uhm, yeah so to have that connectedness.  P3: Yeah.  R: And there’s like a community which is quite nice and the community will only get bigger and bigger and more people will use it and more people will feel confident to go to the gym and so on.  P3: Yeah, that’s it and I think that’s a good thing really.  R: That makes sense ugh are there any other potential benefits do you think of using the app?  P3: Uhm, I think the pictures and the videos on the app is just good uhm and the option of being at home and outdoors uhm is really good.  R: Perfect, ok and uhm what are the potential negatives if there are any of using accessercise to improve physical activity levels?  P3: Uhm.  R: Do you think there are any negatives of using it uhm maybe becoming too reliable on using the app at home that you never want to leave your home uhm to do activity and so on?  P3: No, just that subscription thing uhm I have been in positions that I have not been able to afford a subscription and I have wanted it and I do not want to get rid of it but I have been able to like pause it.  R: Pause it?  P3: Pause it.  R: So, you think that function is something that should be included?  P3: Yeah, yeah because you don’t want someone to uhm and someone who hasn’t got much money at all uhm who is on disability benefits and stuff like that uhm which are really worried about money uhm that they may be able to buy it for a month ugh do a bit of exercise and then pause next month uhm but remember the exercises on their account.  R: Yeah.  P3: Maybe like that.  R: Yeah, that would be helpful ugh I do know an app that uhm they sometimes they offer scholarships uhm for people that can’t afford the full membership uhm they can then contact the app and get like a discount.  P3: Oh, yeah discounts yeah yeah.  R: Something like that.  P3: So, uhm discounted rate would be really really good uhm.  R: Because that could also show that you trust your customers and the customers trust you uhm you build that connection uhm you build that network.  P3: Yeah.  R: Ok so these people are really nice they have given me a discount to continue using their app I’m going to continue using it because I trust them.  P3: Yeah.  R: You support them and they support you.  P3: It would be really helpful and I think that is something that would help the NHS as well uhm because I know the NHS do a thing at the moment where you can get twelve weeks free uhm at the gym ugh the doctor will just refer you to the gym for twelve weeks uhm and you get like a personal trainer.  R: Yeah, I think that is what GPs are doing now they are trying to encourage exercise as a way of improving everyone’s health uhm so instead of giving them medication uhm they will recommend you go to the gym for a month and see how you feel.  P3: Yeah.  R: So, instead of giving someone antidepressants uhm here is your gym subscription for three go everyday and you are likely to feel better.  P3: Yeah, so with this app if you could offer uhm if you are on disability benefits ugh show us proof you can then be provided a discount on it and stuff but also probably in the future if that did work uhm is to start approaching doctor surgeries uhm the NHS and saying you know uhm we have got this app uhm if you have got any patients that uhm you can refer uhm you can be provided a code or something like that uhm sort of like an ambassador thing.  R: Yeah, yeah.  P3: So, the doctor can be like oh there’s this app uhm so if you use that but also use my code green one.  R: Yeah, haha.  P3: You can then get uhm twenty percent off because then that person is benefiting uhm the doctor is doing good uhm they then obviously have to endorse it because they are medical professionals uhm so that could be something included in the future maybe.  R: Yeah, so that’s something they could consider.  P3: Yeah.  R: Uhm, which follows onto the final question is how do you think the accessercise app could be improved if anything for it’s use in improving physical activity levels uhm so if you had to round everything up into three areas for development that they could use from what you have mentioned what do you think they could be?  P3: Add more disabilities uhm more common disabilities uhm I personally think mental health is a disability because that really can stop you.  R: Do you think mental health is a disability uhm is that what you mean?  P3: Yeah, I do uhm I really do uhm.  R: So, whether they could incorporate mental health conditions ugh learning disabilities but also physical disabilities?  P3: And physical.  R: I think at the moment it’s very much just focused on physical.  P3: Yeah, it’s mainly physical isn’t it  R: So, whether they could include someone with autism uhm what is recommended for someone with autism but I guess the only problem they have got at the moment is that there are hundreds of disabilities uhm how can they include everything.  P3: Yes, you can just put it in like the dance the creativity uhm I think children you can do uhm I know this is not aimed at children uhm I can’t think what sport it is uhm it’s like a creative flow where you are doing exercise through drama.  R: Right, ok.  P3: But running around and you’re then touching the floor and that kind of stuff but do it for adults like ugh autism uhm ADHD and down syndrome uhm all love creativity.  R: Right, ok.  P3: But even people like me love creativity uhm because it makes you smile uhm it gets you up but you don’t think you’re exercising so you have got those three categories there in one.  R: Uhm, you can incorporate all of them together can’t you?  P3: Yeah.  R: I agree I think the dancing thing is a good point uhm because dancing is still physical activity it’s still exercise.  P3: Yeah, if they could add something like dancing that would be good.  R: So, whether they could include some creative stuff uhm or get someone with let’s say dwarfism doing a few videos of how to do the salsa dance or whatever you know.  P3: Yeah, because there was someone on strictly come dancing who has got dwarfism.  R: Right, ok.  P3: And she did all of that.  R: So, they could be contacted to provide more information regarding that.  P3: Yeah, I think the accessercise app needs a bit more creativity in what is being provided uhm everyone loves creativity even old people uhm even young people it’s something for everybody.  R: Yeah, because at the moment it’s just focused on developing strength uhm getting toned which is very boring in a way.  P3: Yeah, the goals accessercise provides is good but needs more fun activities.  R: It needs some creativity which is good uhm perfect so the final question is there anything else that you would like to add or discuss before we finished the interview process?  P3: Uhm, nope.  R: Nope, so you are happy to finish haha?  P3: Yeah, yeah.  R: Yeah, perfect thank you so much for Michelle for taking part.  P3: Do I need to log out of the app.  R: Yeah, you can do. |
| --- |

## Transcript of interview undertaken with Participant 4

**Key code:**

R: Researcher

P4: Participant

| R: Awesome, thank you Ryan uhm participating in our trial and getting involved in this work that we are doing here at Loughborough University so uhm you have done the first phase which is great and you have now come back for the second phase which is awesome we have collected all the data and we are now undertaking a think-aloud interview so first of all I will go through the interview overview so I will explain who I am so my name is James Haley uhm and my role within this research project is that I am the lead researcher so what that means is that I’m working with a range of ugh different researchers uhm and I’m the one that is actually leading the study uhm collecting the data analysing it and also writing it up the paper.  P4: Ok.  R: The aims, importance and purpose of undertaking this research project and the benefit of participating is that we know that people uhm with disabilities uhm so for example physical and sensory are physically inactive uhm and as a result of covid this made it worse so therefore they were unable to participate in physical activity but we also know that there are a range of barriers to physical activity whether that is cost uhm transport uhm time to undertake regular physical activity so now with the development of technology and smartphone apps and smartphone technology we feel like the use of uhm mobile health applications may be an effective way at getting people with disabilities more physically active uhm the importance of doing this research is that we can understand the strengths weaknesses and also areas for improvement for the app and we can assist the app developers in improving accessercise further to be able to reach more people and also see whether it can improve various other health outcomes like quality of life uhm wellbeing and other physiological measures uhm the University that I attend is Loughborough University based in the East Midlands of England uhm my position is that I’m a Doctoral Researcher and I’m based within the Peter Harrison Centre for Disability Sport which is within the School of Sport, Exercise and Health Sciences and the other individuals working on this project is Sam Breary which is the director of accessercise uhm Ali Jawad which is the co-director of accessercise and also Doctors David Maidment and Daniel Rhind which are my supervisors uhm I will briefly go through all the ethical considerations in this study so you have read through the participant information sheet uhm you have signed the consent forms which is great so you are aware of what this study involves uhm in terms of confidentiality and anonymity is any information that you share in this interview will be protected uhm that means all your data will be password protected and will be removed at the end of the study also any uhm data you give within this interview will be hidden with a pseudonym which means that your name will be hidden with a fake name uhm also you have the right to withdraw from this study at any time if you feel like you don’t want to participate and also uhm there are no right or wrong answers in this study ugh please feel free to talk openly about the app and give your honest views on what we can do to improve the app further uhm is there any questions that you would like to ask before we continue with the interview process?  P4: No, no.  R: Perfect, awesome so the first introductory question that I have got is please may you provide the following information your name, age, gender identity, ethnicity and county of residence?  P4: My name is Ryan Smith, uhm male ugh thirty-nine years old uhm what was the next one?  R: Uhm, gender identity?  P4: Oh, male ugh.  R: Ethnicity?  P4: White British.  R: And your county of residence?  P4: Nottinghamshire.  R: Perfect, ok so to start could you please tell me how you first got involved in the accessercise application and why?  P4: Uhm, just to get involved in research and to be a participant and help improve physical activity for those with disabilities.  R: Ok, uhm and do you have any experience of using any physical activity apps in the past?  P4: Only one uhm I used to track my weight.  R: How did you find that ugh was it ok?  P4: Yeah, I just googled it uhm then downloaded it and yeah it seemed straight forward.  R: Perfect, ok and is there any other uhm overarching reasons why you want to get involved in a physical activity app and why you want to get involved in some physical activity research?  P4: Uhm, just to contribute I suppose.  R: Yeah, uhm so this is a bit of a trick question but could you please tell me your experience of using the accessercise app and how long you have been a member?  P4: Uhm, I can’t just yet haha.  R: So, yeah, I guess because you have only recently downloaded the app.  P4: Yeah.  R: You’re a new member aren’t you which is fine no problem uhm so this is accessercise specific questions so do you currently use the accessercise in your role uhm if so when and how and if you were to use it how often would you use it do you think?  P4: Well, from the brief thing that you showed me with the videos uhm which shows you how to do an exercise properly that is very good.  R: Ok, and if you were to use accessercise ugh in your life how often do you think you would use it?  P4: Probably a couple of times a week probably uhm because I have got swimming to do yeah.  R: Perfect, ok and do you think using a smartphone app like ugh physical activity app could be something that you benefit from?  P4: Oh, absolutely! Uhm, particularly with the videos that’s spot on uhm because that’s always something that has worried me if uhm I’m doing something wrong uhm and whether I’m causing myself harm.  R: So, you have mentioned about videos, so you like the videos on the app and how it shows you the demonstrations?  P4: Oh, absolutely! The videos on this app of the exercises are brilliant uhm so I have rowing equipment and when I watch videos on YouTube to make sure that I’m using them correctly uhm so somebody with a disability like your own and them showing you how to do it is amazing isn’t it.  R: That’s great, so we know that people with disabilities experience various barriers in terms of you know accessing gyms uhm money and transport do you think the fact that you can use app may help you to do more exercise at home compared to going to the gym?  P4: It would probably encourage me to stay at home which might be counterproductive but if it gives you the information to find a gym when you’re out and about maybe uhm and as we have just discussed about if we could incorporate perhaps disability parking requirements would be really good.  R: Yeah, that would be a good strength to include and I completely with you on that I think uhm because at the moment the app only focuses on local gyms so whether they could incorporate you know local clubs uhm local sporting activities uhm local events uhm but I think parking is really important.  P4: Yeah, because I mean yeah I mean I can swim a fair distance but walking wise it hurts so if the app can show you whether parking is accessible nearby is so important.  R: Because I think yeah if someone finds a gym accessible based on the information on the app they won’t know whether there’s accessible parking and what’s the point of having accessible on the app if the parking is too far away?  P4: Yeah, and parking is not straight forward uhm blue badge parking can be challenging at times uhm it’s very confusing.  R: Yeah.  P4: Yeah.  R: Uhm, I think ugh that could be something that the gym provides for each one so when you press on the gym it shows you how many ugh hours are open a day it shows you the phone number it shows you uhm email address uhm you can then contact them but whether they can tell you whether there’s accessible parking available would be good.  P4: Yeah.  R: Uhm, so that’s stuff that they can work on so you mentioned about this previous app that you used uhm is there anything that put you off using that and maybe why you don’t use it anymore?  P4: It’s just well too ugh I will bring it up now it’s just too over the top it’s designed for people who seriously want to do something uhm and I don’t uhm well effectively I’m unable to uhm the app is designed for someone who is able and I can’t reach their bench mark so it looks like I’m always under achieving.  R: Yeah.  P4: Yeah, yeah, I can’t reach what they want me to do because I can’t.  R: And do you find maybe the previous apps weren’t really focused on the disability itself?  P4: No, they are not uhm yeah so when I’m trying my best ugh my best is not good enough for it.  R: Haha.  P4: Haha.  R: But do you like the fact that maybe this app at the moment you are using uhm accessercise is tailored towards your disability?  P4: Yeah, if it appreciates that everyone has got their limitations then yeah you won’t feel like you are under achieving?  R: Yeah.  P4: Yeah.  R: So, you feel like having an app that focuses on your impairment is a good thing?  P4: Yeah, because the other one recommends that I should be losing a kilogram a week uhm but I can’t do that.  R: Haha, no.  P4: I can’t do that yeah.  R: But in terms of the social sections you saw people there with similar impairments?  P4: Yeah, that’s encouraging because you can find similar people in the same boat as you uhm that’s always helpful isn’t it.  R: Yeah, that’s good to hear uhm but do you think from the previous apps uhm so you mentioned it was overwhelming uhm do you think maybe the cost of using that app was expensive?  P4: Uhm, maybe but I can’t remember how much it cost uhm so the accessercise one is around sixty-five a year uhm I don’t know a lot of us get uhm benefits to help us with these sort of things uhm so ideally some of that money can go towards a fitness app like this.  R: Yeah, so do you think that ugh.  P4: That money is obviously there to help us.  R: Yeah, that makes sense uhm do you think the fact that the app uhm you know has different exercises uhm it has people with different impairments on there is maybe a motivating factor?  P4: Yeah, absolutely!  R: Yeah.  P4: Absolutely! I mean the videos on the app alone for me uhm seeing somebody else in the same boat as myself doing the exercise is really powerful and you have no excuse to not do it.  R: Yeah, that makes sense.  P4: You just have no excuse.  R: But do you think the previous apps that you have used uhm didn’t focus on the impairment and there weren’t’ really instructions on how to use them?  P4: No, it mainly goes on about your diet uhm and scanning barcodes in for food uhm but I like how accessercise provides some welcome instructions when you first use the app which is helpful to use the app more smoothly.  R: It becomes a bit overwhelming, doesn’t it?  P4: Well, yeah, I don’t know it’s just a bit too much.  R: Ok, so you mentioned earlier about uhm obviously like apps for people with disabilities and so on.  P4: Yeah.  R: Uhm, but in terms of exercise apps have you ever seen anything advertised for people with disabilities.  P4: No, never.  R: So, obviously that is an issue in terms of apps and stuff.  P4: No, never uhm which is strange because I just have not seen them advertised around.  R: No, I think that’s because these apps are mainly focused on the abled population and so on so if someone with a disability is supposed to be encouraged to use those apps ugh how do we know whether it’s actually going to support them?  P4: Well, you don’t feel supported because on some of these computer games you go to options uhm it let’s you put in a handicap setting uhm and just now thinking about it uhm the previous fitness apps that I have used didn’t even give you that so.  R: So, when you say handicap do you mean that this app you have used is disability specific?  P4: Well, yeah some computer games will have colour blindness uhm also whether you have got one arm because you can’t use the controls properly uhm I mean just thinking about it this other fitness app hasn’t got that uhm so no this accessercise fitness app is good isn’t it.  R: Yeah, that’s good uhm so my next question is uhm so I showed you the app uhm I have showed you the different functions but what were your first thoughts when you first started using the accessercise app uhm did anything stand out to you?  P4: Yeah, well the videos straight away uhm because for me I procrastinate a lot but if you have got a video of someone doing something that’s great.  R: Do you like the fact that the person in the video is someone with an actual disability that you have selected?  P4: Yeah.  R: Ok, and in terms of like uhm the social media section do you like that how you can interact with others?  P4: Uhm, maybe uhm it wouldn’t be the actual function uhm it would be based upon the people that you actually get in touch with I think because if it’s a group of people that ugh you’re all getting on with it’s good.  R: Yeah, that makes sense. How about uhm the actual exercises itself so the filtering section where you can select the goal?  P4: Yeah, that’s good uhm I like that a lot where you can select your goal from the big list of options because uhm so yeah doing it from home and being able to show others what you are doing is great because I am guilty of using that as an excuse oh I haven’t got anything and effectively that is why I have got a home gym now and I still make excuses.  R: So, do you think the fact that there’s adapted equipment options where you can do it at home is great?  P4: Yeah, the ability to customise and choose adapted equipment for your workouts is an amazing idea.  R: Uhm, ok.  P4: Yeah, it’s brilliant very brilliant ugh.  R: Do you like the fact that you can select not just building strength uhm or toning but there’s also variations of different goals on there?  P4: Yeah, yeah it’s good uhm there was one thing I was going to say but I have forgotten now something uhm you were showing me uhm but I am thinking to myself uhm will this app encourage you to isolate yourself if you get into this and you’re doing it from home uhm is there nothing to motivate you uhm so like why don’t you go and join a group uhm why don’t you get involved uhm because I think a lot of people that are disabled uhm put walls up.  R: Yeah, they put barriers up.  P4: Yeah, because sometimes you feel like you’re a burden on people having to accommodate you and sometimes you feel like you’re a pain.  R: I guess because you can do it at home uhm using your own phone it could be in one way useful.  P4: Yeah, yeah maybe.  R: But do you think anything else stands out to you?  P4: No, I don’t think so.  R: How about the actual uhm exercises itself uhm how you have over two-hundred and fifty?  P4: Yeah, I was browsing through them all now and yeah what equipment you can use uhm inside and outdoors uhm the app developers must have spent a lot of time thinking about this.  R: How about the colour?  P4: Well, yeah, my favourite colour is purple so you’re asking the wrong person haha.  R: Do you think ugh the white goes well?  P4: Yeah, the white and black is good uhm don’t make the same mistakes as some sites where they have the black background with the white writing.  R: Uhm, ok.  P4: Yeah, because I find it hard uhm because you don’t want to really engage with it.  R: Right, ok and in terms of obviously the content on the page uhm do you think maybe there is a bit too much information there uhm do you think maybe they could lay it out a bit better?  P4: Maybe I mean I like when I do the cooking uhm like recipes I like bullet points or step by step uhm I am the sort of person that likes step by step instructions uhm I’m put off by big blocks of text uhm but everyone is different aren’t they.  R: Yeah, I guess it depends on what sort of learner you are.  P4: Yeah, yeah.  R: Uhm, but if you go back onto the videos so if you watch a video for example uhm so obviously here it has got the written descriptions uhm it has got someone doing it uhm but do you think there could be a voiceover included?  P4: Maybe, uhm a voiceover would be handy wouldn’t it but uhm it’s not necessarily uhm maybe somebody telling you uhm for example what worries me when I’m doing an exercise is that I will get because I have got nerve damage so I will get random pains right and I always start worrying to myself is that uhm should I be feeling that pain uhm should that pain be so intense and how long should I be feeling that pain uhm am I doing myself good or bad uhm you know and other things that worry me slightly but I don’t know how you would really incorporate that in because even if you have got two people with nerve damage next to each other it’s not going to be the same is it.  R: Yeah, because pain is different I guess.  P4: Yeah, so that’s one of the things that worries me ugh yes so that’s probably why I’m a bit boring and I stick to ugh the same exercises over and over again uhm like swimming cycling and whatever.  R: Right, ok. So, if we were to get of this one uhm so for example if you go back onto here.  P4: This one?  R: Yeah, press that one uhm but we will just pause for now uhm so my next question is do you have the required equipment to use accessercise in it’s full capacity uhm in other words access to a mobile phone to use the application?  P4: Yeah, yeah.  R: Would you say your phone is reliable for you to use the app?  P4: Yeah, phone, tablet all good yeah.  R: If you were to go uhm so let’s say for example if you were to go to the gym would you bring your phone with you or would you bring a tablet?  P4: Oh yeah, I would bring my phone obviously because I’m using it everywhere I go yeah.  R: Do you think the thought of maybe using a phone at the gym may be off putting?  P4: No, no.  R: Ok so you do have the full capacity which is good uhm so based on obviously you seeing the app uhm you have seen you know the accessible section uhm you have seen the exercises uhm the shop and so on are there any improvements that you believe could help improve the accessercise application?  P4: No, I mean I think if you could incorporate parking uhm that’s the only thing I would see as ugh I think that would be for a lot of people uhm the whole mobility thing of being disabled is important and everything ugh to remove barriers ugh mental barriers and make things easier ugh because all humans have difficulties and it’s important to find ways to overcome them.  R: Yeah, but in terms of uhm selecting.  P4: No, no I find it brilliant I think it’s ugh the filter setting is very good uhm so I’m happy with it.  R: Ok, so for example if you were to press like gym uhm or home and let’s say you were to select like strength.  P4: Uhm, which one?  R: Increase strength at the top that’s it.  P4: Ok.  R: Do you think maybe they could do something where you are doing it at home uhm you are doing strength and you can then narrow down the equipment that you need for that specific goal?  P4: Uhm, maybe.  R: So, for example if I was to do uhm home ugh flexibility and I wanted to do yoga it would remove all the strength stuff so narrowing down the options.  P4: Yeah, maybe but could that be too difficult so for me I keep getting told that I need to build strength up in my back but for me as a not very sporty person I don’t really know which exercise to do.  R: Right, ok.  P4: So, could you filter the other way around so I need to strengthen my back up so uhm could they show me what exercises are good for my back.  R: Do you think it would be good to provide maybe some tutorials or instructions on selecting the right goals?  P4: Yeah, maybe something like that uhm because I wouldn’t know really which ones of those would be good for my back.  R: Yeah, so I guess having tutorials ugh having videos of the specific impairment would be helpful.  P4: Yeah, yeah because I’m meant to build strength in my back and my right leg so which one would I go for uhm I just don’t know.  R: That makes sense uhm do you like the images they have there?  P4: Yeah, the images they have used on the app is good yeah.  R: The mobility with the walking frame uhm but do you think having that image next to mobility is quite off putting?  P4: Uhm, it’s a bit but I suppose it’s a bit.  R: I guess someone would think mobility is for someone old.  P4: Yeah, but it’s a broad umbrella isn’t it.  R: Do you like the fact they have sort of split them up so you can see each one with the logo?  P4: It doesn’t matter what picture you go for uhm my dealings with people in real life you are never going to be able to get everybody on board.  R: Yeah, that makes sense.  P4: Uhm, you’re never going to get everybody on board.  R: So, that’s the exercises uhm in terms of improvements let’s think about ways that could motivate you to use the app long term?  P4: Well, this is a problem for me because uhm I wake up with back pain and that is my demon and I become very ugh demotivated uhm I don’t really know how to tell you uhm how to keep me interested uhm even now I have been on this diet since like February uhm I have made good progress uhm I have lost eighty kilograms and I feel better for it even now I could feel the benefits uhm and I know it’s doing me good uhm I have more energy and all the rest of it and I have lost weight uhm people are saying that I look better uhm clothes are fitting better but even now I’m bored of it already uhm I can feel myself bored of it so how do I keep going.  R: Do you think something like streaks so for example on your phone if you were to be notified everyday oh you have done another day uhm and you get to the point where you have done like one-hundred and fifty days straight.  P4: Uhm, I guess streaks could help with motivation to undertake exercise ugh.  R: And you don’t want to lose that streak.  P4: Uhm, maybe I don’t know uhm I know what you mean.  R: It’s because you don’t want to lose that streak.  P4: No, because one of my ugh earlier electric cars used to have ugh the more you used it the more environmentally you drove uhm every day a tree would grow on the dashboard to encourage you to carry on driving well uhm you would get more leaves on your tree uhm.  R: Yeah.  P4: Yeah.  R: Do you think that could help?  P4: Uhm, maybe because a lot of people back when that car first came out people were posting on social media uhm to see how many leaves on the tree you got and I suppose yeah uhm I don’t know I suppose.  R: I think it’s just trying to find ways to encourage you.  P4: Yeah, but eventually even then you get fed up of driving like a granny and you uhm sod your tree you just want to get somewhere don’t you.  R: Haha.  P4: Yeah.  R: That makes sense uhm do you think the idea uhm so you have got the social media section which is great but do you think there could be anything in terms of leader boards uhm competitions with other users could be included?  P4: Uhm, maybe as long as it’s healthy competition because that’s the thing isn’t it once again because you have got a group of people with all sorts of disabilities and ugh some people are going to feel that I can’t keep up with the pack and it’s a sad thing in life uhm because there’s always going to be someone last.  R: Yeah, uhm the thing with disability is everyone is different and everyone’s experience is different.  P4: Yeah, uhm I think it would be a great idea but it has to be done in the right way hasn’t it.  R: Yeah, because I think you don’t want to cause injury uhm you don’t want to rush people.  P4: And all I can say is that someone has always got to be last and you don’t want to disturb them from doing the exercise.  R: No, that makes sense. Uhm, I agree it’s trying to find ways that can get the person to do more uhm so for example you think about New Year resolutions you have a certain amount of people that really go for it.  P4: Yeah.  R: After a month people can’t be bothered.  P4: Yeah.  R: It’s because the idea of doing it for a bit is ok uhm but how can you encourage that long term behaviour for someone to really you know change.  P4: Yeah, well something that I really struggle with is uhm I eat out a lot uhm and it’s trying to stick to your diet uhm while you’re eating out.  R: I eat out a lot as well haha.  P4: Yeah, well.  R: It’s the convenience of eating out isn’t it.  P4: Yeah, I’m always out and about uhm I’m quite socially active and yeah ugh it’s like uhm how do you keep a diet going when you have gone to a restaurant uhm and you look at the menu and there’s so much good food.  R: Haha, yeah I have had the same situation uhm it’s the convenience uhm someone would rather workout at home than workout at a gym because if someone could have a home made gym they would rather do it at home because it’s cheaper.  P4: Yeah.  R: It’s also finding a way to get people involved.  P4: That’s why I got all this stuff at home uhm and I’m not body shy or anything but I just prefer doing the exercise on a treadmill or the rowing machine at home uhm because sometimes when I finish exercise uhm I’m in a state and I am quite bad and there has been a few times where I have gone swimming and when I am having a minute uhm the lifeguard comes over and asks me how I am and I am like yeah I’m fine you have been asking me that for years now I’m fine I’m literally just floating for a bit and I am literally having a minute and maybe it’s because of my disability and from the outside world I don’t look disabled so when I am struggling it does look a bit strange uhm I don’t know but I do find it quite annoying.  R: Yeah.  P4: Because yeah uhm particularly swimming baths where I have been going for over a decade now.  R: Yeah, especially because they know you so well it must get quite annoying.  P4: Yeah, exactly.  R: Haha.  P4: Yeah.  R: In terms of your bike uhm you mentioned that you enjoy using that which is great but what was one of the motivating factors to get you on that and actually to use it uhm was there anything in particular that would encourage you to do that?  P4: Uhm.  R: That maybe could be transferred on this accessercise fitness app?  P4: I don’t really know really.  R: I think you mentioned about doing it with friends.  P4: Well, yeah it’s nice to be well yeah doing things as a group uhm I like doing things with people so yeah I mean if you could meet people in the same situation as you or likeminded.  R: So, that goes to the group section uhm so you said about following people ugh finding groups for people that are similar to you.  P4: Yeah.  R: So, as you can see here there are different groups.  P4: Yeah, but the thing is you have to try find people with your disability uhm and living in the same situation as you so it’s ugh.  R: I guess with yours you experience a lot of nerve pain uhm you can go onto the spinal section.  P4: You think so?  R: I think nerve pain in the spine where you experience issues would definitely be more spinal so I think you sort of need to narrow it down into a group.  P4: Yeah.  R: If you were to be too specific it would be hard to find.  P4: Yeah, not quite an app orientated but over the years going to different groups uhm you will tend to find uhm some groups are very uhm it’s their community.  R: Their community?  P4: Yeah, and sometimes it’s a bit ugh difficult.  R: Sort of invasive?  P4: Yeah.  R: But do you think for example being a part of a group would be something that you would enjoy and appreciate?  P4: Oh, absolutely! Well, of course it would be uhm and you learn about working in a team and all the rest of it uhm exchanging information is great isn’t it as long as it’s done in a healthy way because that’s like everything isn’t it uhm as long as it’s done in a healthy way.  R: But in those groups is there anything in particular there that you would like to see happen or occur uhm would you like there just to be physical activity?  P4: Oh, no of course not I think uhm because with the other app uhm the normal fitness app I used it’s just too intense.  R: Yeah.  P4: It should be fitness and like state of mind and also having a bit of ugh real life banter because like with the chronic pain group I used to go to uhm most of the time spend there we didn’t talk about chronic pain uhm it was twelve to fifteen people who have got chronic pain talking and with like mental health that is brilliant because everything is chilled and on this accessercise app with everyone being disabled uhm we are all on the boat one way or another together aren’t we.  R: Yeah, it’s a difficult one because with the developers they are currently focused on developing the app for physical activity and exercise but whether they start incorporating parking uhm and groups for other stuff uhm it sort of takes away the focus of the app.  P4: Maybe.  R: I think it would be beneficial uhm but that would be quite interesting.  P4: Yeah.  R: But in terms of ugh having people follow you and you follow them uhm do you like that idea?  P4: Yeah, once again uhm as long as it’s done in a healthy way as the internet can be ugh dangerous.  R: If you were to go back onto the exercise one uhm that’s the one uhm and you have got this one here ugh so this is your profile.  P4: Yeah.  R: Is there anything that you like about this so the fact that you can add a photo ugh do you like these features?  P4: Well from my own experience I don’t have my photo on Facebook or anything like that uhm and that’s mainly because of my old job when I was taxi driving uhm I used to get people who I didn’t know adding me uhm and I know other people that have photos of themselves on there and things like that uhm I’m not a fan of that.  R: No, right ok. Uhm, but the fact there’s the option to do that and the option to have followers and achievements.  P4: Oh, no no that’s all fine yeah I don’t need a photo on my profile I think.  R: I think what they are trying to do is uhm make it you know ugh provide options for people to provide a bit about themselves uhm do you think that is something that could be developed uhm maybe have a description about themselves?  P4: Yeah, yeah you can do all sorts of things uhm you could add a brief description about yourself uhm this that and others yeah.  R: Yeah, but at the moment I think it’s just got training uhm I think training would be pretty good so if someone was interested in doing strength uhm you could put that so if you were to follow someone else uhm you can see they ugh are interested in that.  P4: Yeah, if you set the foundations to build a community, I think it would definitely thrive yeah.  R: In terms of achievements, is there anything in particular you think they could benefit from adding there?  P4: Well once again it’s got to be done in a thoughtful way hasn’t it uhm if you get it done to the standard uhm that you feel like you’re achieving something but not that people are struggling uhm you don’t want people to feel like they are being punished.  R: Uhm, what sort of things would you like to see for that uhm would you like to see daily ugh challenges uhm daily records?  P4: Ugh, I don’t know about daily uhm because for a lot of people daily would be ugh.  R: Too much?  P4: Yeah, for some people it would be far too much.  R: Maybe weekly?  P4: Yeah, maybe weekly or whatever uhm maybe uhm not so me but a lot of the young ladies that have osteoporosis they wouldn’t be able to exercise daily.  R: No, it’s quite a difficult one.  P4: Yeah.  R: But do you think from just seeing that ugh anything else could be added.  P4: No.  R: You don’t think there’s too much information?  P4: No, not at all I think it’s fine yeah I think it’s fine.  R: Perfect, ok awesome.  P4: Uhm, but in terms of the map though it would be good to have parking included uhm I don’t know how you would do it because thinking about it uhm not everyone drives do they so then you have to put in like disabled bus routes and things like that uhm so maybe that should be left for people to sort themselves out I don’t really know.  R: That makes sense.  P4: Yeah.  R: Right, we will move onto the next question so do you use any other similar applications outside of accessercise if so what is better or worse than accessercise?  P4: Uhm.  R: So, at the moment you don’t know?  P4: Not really.  R: That’s fine uhm right so we will move on so we are now going to move onto the main part of the study and ask you to participate in a think aloud protocol whilst using the accessercise application uhm the instructions for a think aloud protocol are straight forward I would like you to say out loud what ever comes into your mind there are no right or wrong answers so what ever you think about when you use the app let me know that’s perfect uhm we will just run through a quick practice to get you used to the protocol uhm could I ask you to say out loud everything that comes to your mind when you think about physical activity?  P4: Ok, what I think aloud uhm so straight away I think there are many negatives when it comes to physical activity uhm it hurts.  R: Right, ok.  P4: It’s painful uhm effort it’s just setting the equipment up uhm it is a tremendous ugh mental effort because I know it hurts uhm yeah my back ugh my leg ugh and recently my right arm it’s a huge effort uhm but you just have to try and push through uhm and also when you’re out on your mountain bike once you have got yourself up and going uhm I love it uhm it’s great I find it awesome uhm and I know that I mumble to myself that I don’t want to be here doing this but ugh yeah as soon you mention physical activity uhm in terms of walking I’m thinking jesus christ this is going to hurt.  R: But afterwards you feel slightly better?  P4: Yeah, of course particularly when I’m out on my bike and I have got my music on uhm with the sun coming out and I do know and I suspect a lot of other human beings we are our own worse enemy yeah.  R: Yeah, that makes sense ok uhm so we will go through your phone together and I will ask you different questions uhm so this is the main bit now so you have made an account uhm so you are using my account for this session but uhm what are your first impressions when you first saw this app?  P4: Well, it comes across pleasant straight away sort of thing uhm and compared to the other fitness apps uhm there are only four options at the bottom and it’s straight forward and clean and if you look at the other fitness apps uhm you have your home screen.  R: Yeah, the black doesn’t work well no.  P4: No.  R: But I think with the white and purple on the accessercise app it goes really well.  P4: Yeah, I mean yeah this is literally it’s good.  R: Do you think the options down the bottom with the social uhm exercises and also explore are good to have down there?  P4: Yeah, that’s fine.  R: Yeah.  P4: I have got small hands and phones are massive now but yeah you can’t do it any different can you.  R: Ok, I think what they are trying to do with the social section is to make it like twitter uhm with all the posts.  P4: Yeah, I think that’s a good thing uhm I think that would make a difference uhm because I don’t think people will come straight on uhm they will hit their head against the wall uhm they will be like jesus this is a fitness app uhm they will come on and see the social uhm and see people posting things I think that would be yeah it’s nice.  R: Do you think so for example here Liv has posted this but I don’t think this is Liv in the video do you think maybe what they are posting is ok uhm and whether they could video themselves completing the exercises?  P4: Well, it’s up to them though isn’t it.  R: Yeah.  P4: Once again, you’re getting into ugh a problem because you’ll never be able to keep people happy are you because they could be open to abuse.  R: Ok, so if we were to press on Liv quickly.  P4: On her profile?  R: Yeah, so here you can see some personal information uhm city uhm do you think that’s all ok you can see some Liv uhm you can unfollow her uhm does anything come to your mind when you think about that?  P4: No, but once again maybe a small section uhm where they could be a brief description about themselves if they want to about themselves and their situation because for some people with a disability uhm it breaks down the awkwardness of people tip toeing around your limitations.  R: Perfect, ok that’s fine uhm so if you were to press the three dots in the top corner uhm let’s see what that does.  P4: Ok.  R: Ok, so it’s got the privacy stuff uhm so block ugh report uhm that’s pretty good.  P4: Yeah.  R: Do you like the use of colours there?  P4: Yeah, yeah that’s fine uhm even with the perfect colours you will not make everybody happy are you.  R: No, haha.  P4: And even from my board game society uhm colours people are very strange haha you can’t keep everybody happy.  R: Haha, so if we were to go back uhm perfect ok uhm so you have got the groups that you liked which you mentioned about joining.  P4: Yeah, that may take a while to search through your groups to find one that you ugh like and matches up to yourself.  R: Do you think now that you have seen the group option and the fact that you can join little groups uhm do you think you will be more likely to go onto it?  P4: Yeah, yeah uhm when you have got a minute you will sit there and have a look around won’t you sort of thing.  R: But do you think it’s something that you will go on just to go onto use because of the group function or do you think you will go on it for other reasons?  P4: Maybe it depends what you find isn’t it uhm it depends on what people you build up wouldn’t it.  R: What sort of groups do they have on it at the moment?  P4: It’s just loading at the minute uhm it’s not happy.  R: No.  P4: Oh, I think it’s done now.  R: Ok, so we have got things like new to this so I guess that would be an introductory one.  P4: Yeah.  R: Uhm, so you have got spinal cord.  P4: Yeah, they are good ones to have uhm straight away.  R: Uhm, do you think they could maybe categorise these groups into ugh for example I think I’m new to this uhm I’m not sure really what that means.  P4: Uhm, maybe but I don’t think you would want to have too many sub groups uhm maybe because also those two groups there you could use this as a gateway to get somebody who isn’t thinking about uhm so they want to start looking after themselves better.  R: Yeah.  P4: But they are a long way off that yeah.  R: Right, ok.  P4: Getting people on here and talking about it uhm even if it takes them six months to start making some progress.  R: Yeah.  P4: Yeah.  R: What actually happens when you press on uhm that option?  P4: I don’t really know.  R: It’s got a description.  P4: Yeah, that’s good.  R: It tells you how many people joined uhm that’s also pretty cool how you can join a group uhm it’s got a bit of a description uhm do you like the fact that it’s got a photo to show you how many people have joined?  P4: Yeah, yeah.  R: It’s pretty simple to use.  P4: Yeah, that’s good that.  R: It’s got a support group in there.  P4: If you can get people into that uhm that’s the way to go uhm yeah building things up slowly.  R: Ok, perfect so if we were to go back uhm so go back to the home page uhm so we have done all of this and if you press the filter option do you like how uhm for example locations at the top and then you have goal next to it or do you think goal should be at the top and then the location?  P4: No, it seems alright doesn’t it.  R: I had a participant mention that if you go down to the bottom uhm this is all available and then these ones you have to untick whether these could still be open as well.  P4: I think at that point you will be scrolling for days wouldn’t you.  R: Yeah, uhm but I was wondering whether for example if you were to choose uhm home uhm and you were to build muscle whether the app could then remove the ones that are not related to your goal.  P4: Maybe.  R: So, outdoor training won’t be done at home so whether that could be then blocked out uhm how about the option that you can press to require a carer or an assistant uhm what do you think would be interesting in terms of that?  P4: Yeah, that’s ok uhm it’s not going to stop somebody doing it uhm I mean is this the idea of trying to encourage people uhm I mean I don’t know I’m luckily not in that situation so I can’t really tell really.  R: But do you think there is anything the gyms could do to work with the app developers to encourage?  P: Well, anything I suppose to make things easier uhm improving things like ease of access and all of that sort of stuff which for a lot of people is a big hurdle uhm a lot of things are difficult.  R: Ok, awesome. Uhm, so if you were to press on this option do you like how the fact uhm it has got the videos uhm it has got the title do you like how you can favourite here as well?  P: Yeah, yeah well what happens if you favourite something?  R: You can go to your favourites and all of the ones you favourited will be there.  P: Well, yeah that’s brilliant isn’t it uhm yeah once you have built up some favourites then that’s great.  R: That’s fine, so if we were to press on this one uhm so you have got videos uhm you have got the favourites which is great because you can then add that to your favourite list do you think here which we mentioned earlier about do you think that is too much text?  P: No.  R: No, uhm so you don’t think there’s too much there?  P: Uhm, I don’t think so no.  R: But here there’s bullet points uhm do you think they could include that all over?  P: Yeah, but up here is your description isn’t it uhm I don’t know uhm maybe but I am a step by step person uhm so I would prefer bullet points but I don’t think it’s enough to put me off and I am put off by text.  R: Haha, I’m the same sort of person.  P: Yeah.  R: Perfect, ok so we have got the description uhm the key benefits uhm target groups uhm do you think they could benefit from any additional information there or anything that you would like to see on there?  P: Not really.  R: Do you like this?  P: Yeah, yeah but maybe this is something that I’m a bit paranoid about but maybe having something small at the bottom that says uhm this might cause this to hurt or something like that uhm so maybe like a warning of what this exercise may or may not do.  R: Right, ok.  P: Yeah, because over the years now I just hate it when you do something and the next day you wake up in pain.  R: Pain?  P: Yeah, you are in a lot worse position than before you started but again what I have learnt is that you need to try to see if there’s any benefits.  R: Uhm, do you think on this app uhm they could provide options for graded tasks uhm or even more difficult exercises uhm because you may finish an exercise like this one uhm you may think of how could I make this one more challenging.  P: Well, yeah this is something that I have noticed with the e-bike uhm I think in the grand scheme of things I don’t think I’m actually getting that much exercise in because what I understand is that you need to be actually sweating to be doing something and I am not so I don’t know uhm you need to strike a balance with something that is easy and you’re not really doing anything but then again you are getting out and about and doing something to actually doing some fitness so you have got to ugh balance that out.  R: But I wonder whether on the actual exercise they could say something like this exercise is a bit more difficult.  P: Yeah, once you have done it a few times uhm you can then say add this to it.  R: Uhm, I’m thinking right because if we are looking at smart targets uhm we are looking at like you know goals and getting people more stronger uhm people would do the exercise and think ok I have done this one hundred times uhm how do I make it more challenging so whether they could actually provide the option.  P: Yeah, so add this to it or uhm remove that component uhm have you thought about doing this.  R: So, you really like the videos on the app?  P: Yeah, absolutely! The videos included are great.  R: Perfect, ok that’s great.  P: Uhm, I know it’s going to be resource intense doing the videos but for a lot of people that will be good.  R: Just one thing that I forgot to speak about so uhm you have the filter section here uhm so you go down to uhm let’s say outdoor training uhm do you prefer for example this to be a bit more organised so for example you have got two options here then three uhm then two two and then three.  P: Maybe and also uhm.  R: Do you think maybe something like alphabetical order could work?  P: Yeah, I do like when things are in order particularly alphabetical.  R: Yeah, because at the moment it’s like two three uhm two two.  P: Alphabetical order is the way to go isn’t it.  R: Yeah, just to make it that bit more easier.  P: Yeah.  R: Uhm, right so we will move onto the next section.  P: Is there the option to make the text bigger uhm or is that within the actual phone setting?  R: That might be more the phone setting.  P: I think it would be good to have the option to increase the text within the setting option on the app.  R: That could be something that maybe they could include on the app.  P: Yeah, for a lot of people that would be helpful.  R: It’s a good point uhm so that’s all done so we will go onto the final section which is explore so what stood out to you when you first saw this uhm was it something that you liked or disliked?  P: Yeah, once again.  R: So, as you can see it says three uhm are you wondering what this is at first?  P: Well, is there different logos for different things?  R: Yeah.  P: Oh, I see.  R: But do you prefer maybe uhm an understanding of what each one means.  P: Yeah.  R: A few people have said what does the different logos means.  P: Well, yeah a map needs a key so you know this app could benefit from including something like this.  R: I don’t think the app has that uhm so it could be developed further by including that.  P: No, it really should have a key.  R: So, maybe that is something they could add.  P: Yeah, a map needs a key or your little symbols are pointless uhm because it may mean something to you but it doesn’t mean anything to people using it for the first time.  R: If you were to press on one for example uhm the information there do you think there is anything that could be added to that?  P: No, no like I said.  R: What about photos or videos?  P: I mean photos they could follow something similar that the Nottingham city council do with the blue badge things uhm a photo is good because you know what you’re looking for uhm don’t you.  R: How about a three-sixty rotation of the gym?  P: Uhm, a three-sixty rotation of the gym and what’s included would be amazing.  R: Haha, uhm it’s all about finding ways to improve the app.  P: Maybe uhm I mean if you just open it to the community and let people to upload what they want uhm and as long as you uhm one way or another administrate it uhm because people tend to go over the top.  R: That makes sense.  P: So, the more that you add for yourself uhm you’re also adding more work.  R: So, for example if you were to press on this uhm how about the rating system uhm so accessibility rating uhm so the way that I see this is that someone may rate five out of five.  P: But once again everyone with a disability is different and have their own limitations so that is going to become ugh potentially a mini black hole because what may be accessible for me uhm may not be accessible for somebody else.  R: But are you also wondering what accessibility means uhm in terms of equipment or other things?  P: Yeah, exactly uhm it could mean anything from the car parking to the toilets.  R: So, on that conversation do you think there is anything that they could improve on?  P: Uhm, I suppose for the accessibility ratings you have to broaden it out to like three or four sub categories aren’t you but once again that is more work.  R: It may be more work but do you think it will be beneficial?  P: Yeah, yeah absolutely yeah.  R: Because when I think about accessibility and I think well this is someone with a spinal cord injury uhm what do they mean by accessibility.  P: Yeah, exactly so straight away for me going upstairs really hurts uhm it’s one of the activities that really is painful uhm the motion of going up and downstairs really hurts my back so sometimes I will look at places to see if it has got a lift because straight away the stairs are painful.  R: Do you think this is an option that you think you will end up using uhm do you think it is something that may help?  P: Yeah, yeah, I will try anything once.  R: It’s good that it’s powered by google.  P: Absolutely!  R: Do you think they could benefit from a link to their website?  P: I don’t think some people will really take that on board uhm but isn’t it already there?  R: No.  P: Yeah, you definitely need to include a link to the gym’s website yeah you need that.  R: So, you said it earlier about having parking logo on there uhm what is accessible uhm which is good but having a link on there would help because at the moment it’s just their email address and contact details.  P: Yeah, you need a link to their website because that might cover quite a lot of the questions that we have just been talking about.  R: Yeah, that makes sense how about if you search something do you like that function?  P: Yeah, yeah searches are always good.  R: In terms of actual reviews, would you like to see any reviews of the gym’s users on here?  P: Yeah, yeah but once again that has got to be administrated haven’t you because people can be over the top.  R: Because I guess if you press on gym you want to see a bit of information about people.  P: Yeah, yeah.  R: How about notifications would you like to be notified regularly?  P: Well, maybe notifications may be a good thing.  R: So, if you were to design a workout would you like to be notified of when to complete that?  P: Maybe but we live in a world uhm where you have all these apps uhm trying to uhm go through your attention is it going to become another notification that you ignore sort of thing uhm you need to try and find something that engages the person.  R: Yeah.  P: Once again I don’t know how to suggest that uhm.  R: I know what you mean.  P: I am guilty of it uhm my friend and his girlfriend are learning to speak Spanish and the app that they use uhm has this thing that you were talking about daily things.  R: Uhm is this Duolingo?  P: Something like that yeah yeah those two are interested in it but I don’t know how long it will stick for.  R: That’s the thing it’s trying to find ways that can motivate people to do it long term.  P: Yeah.  R: If we were to zoom out so you have got the more section now uhm so in terms of the shops is there anything in particular that you would like to see from the shop?  P: Uhm.  R: So, at the moment it’s still under development.  P: Uhm, I don’t really know uhm so when you showed me I didn’t really know what you would expect from a fitness shop I don’t know.  R: So, whether that would be people are signposted to equipment to buy based on the workouts they have created.  P: Maybe.  R: It could even be discounted equipment based on your streaks.  P: Equipment appropriate for your disability would be very handy I think maybe uhm yeah I think that would be very handy.  R: So, for example if you need a dumbbell for a certain exercise you could then be linked to a website.  P: I know it sounds ridiculous but my right hand I find it difficult to peel fruit uhm carrots and stuff and it took me ages when I was typing different things into the google search engine to find a machine that peels stuff for you.  R: Right, ok.  P: You would think that it would be so straight forward but it really wasn’t.  R: Yeah, I know.  P: Yeah.  R: So, whether they could link stuff to that would be quite helpful uhm because if I needed a certain type of equipment which I don’t really know where to get it from it would be good to locate it through the app.  P: Yeah, that’s it uhm maybe yeah that could be handy.  R: In terms of nutrition, you mentioned this earlier about what you would like from that?  P: Well, this is the first isn’t it so maybe just a guide showing you uhm because my own arrogance I did not know that certain things were so bad for you uhm so instead of showing recipes it would be good showing that this food product is high in sugar uhm high in fat uhm because people like me are quite arrogant to what is made out of what.  R: So, would you like an education resource?  P: Yeah, maybe yeah just to point out uhm don’t assume because this is something that I have learnt through my healthcare is that you go and see these specialists and that is their professionalism so they know the information and I don’t uhm I don’t so I didn’t know that tomato ketchup sauce or sauce in general uhm had loads of liquid sugar uhm I have only found that out over the last few months.  R: You wouldn’t think tomato ketchup has sugar in it would you?  P: Yeah, but again I bet you a lot of people didn’t know that because some people live in their own bubble.  R: I think having things like what is a protein uhm how much protein you need is valuable?  P: Yeah, uhm because what is a protein uhm another thing that annoys me is when people say the word cardio uhm like I have no idea what that means.  R: Yeah, I know what you mean.  P: Ugh, I just don’t know.  R: So, whether they could do videos explaining what different types of nutrition is would be good.  P: Yeah, uhm so you need to do more cardio and I’m like I don’t know what you’re talking about.  R: I think having videos of people talking about what they are.  P: People talk about carbs uhm I’m like I have no idea what you’re saying uhm I’m sorry but I’m clueless it’s not my area.  R: Yeah, people could benefit with being provided definitions and explanations of all these things.  P: Yeah, little basic stuff.  R: Yeah, that makes sense I think that’s a good one uhm when I think of nutrition you don’t want to be saying uhm take this uhm take that because people start thinking that I need to do this every day.  P: A lot of people feel stupid to ask though and I’m like I just don’t know what these terms mean.  R: Yeah, makes sense. So, that is something that could be worked on so educational videos.  P: Yeah.  R: Or even a mentor or something like that.  P: Uhm, I mean don’t go overboard with it but keep it straightforward.  R: Even like a minute video of someone describing that a protein is this uhm a carbohydrate is this.  P: Yeah, too much protein causes this uhm too much carbohydrate causes this uhm so trying to encourage people to understand that it’s all about balance.  R: Yeah, but also how much of the carbohydrates you need per day.  P: Maybe but it’s important to try and make sure that’s relevant you don’t want to give people ridiculous examples uhm because online I always see bananas yeah.  R: Haha, makes sense. Uhm, in terms of blogs is there anything in particular that you would like to see from blogs?  P: Not really uhm I’m not really a blog sort of person I do listen to a few podcasts uhm when I’m driving sort of thing but uhm but yeah blogs I’m not really ugh a blog sort of person uhm so you’re asking the wrong person haha uhm but I do like a good podcast because you get to listen about people’s progress.  R: Ok.  P: You can get onboard someone’s journey with them uhm can’t you really.  R: Uhm, so would you like to have users uhm involved in podcasts?  P: Yeah, maybe uhm you will always find people that are outgoing and extroverted who like taking people on their journey with them sort of thing.  R: Yeah, it will be good to see how you find the app and how you get on with it uhm and if you have any questions, please feel free to let me know uhm so that’s perfect so now we will go onto the last final bit uhm so you have got account settings.  P: Yeah.  R: So, do you like all of that sort of stuff so uhm impairment filters?  P: Yeah, it’s all straight forward isn’t it.  R: Exercise habits?  P: Yeah, yeah I think you will be alright with it uhm measurements.  R: Metric and imperial.  P: Yeah, I think you can’t go to wrong with that.  R: Yeah, you can’t go wrong but overall do you like the app?  P: Yeah, it’s fine uhm I prefer it with the black and white text.  R: You then have log out and delete which is pretty good.  P: Yeah.  R: Awesome, right so we will move onto the final few questions so to summarise everything uhm what are the potential positives if there are any of using accessercise app for improving physical activity levels.  P: What do you mean?  R: So, what do you think the actual benefits to users are of using this app uhm so is there anything?  P: Well, anything.  R: Is it quite easy to use uhm is it user friendly?  P: Yeah, to me it’s easy to use and it seems pretty simple uhm.  R: And it’s a good way to socialise and meet other people.  P: Yeah, and I think for a lot of uhm disabled people that want to try uhm and do better in terms of exercise the social side uhm that has got to be the thing first uhm because I think that will carry the weight more than the fitness.  R: But also I think you might have streaks uhm achievements that might motivate people, so I think there’s more positives on the app than negatives really uhm involved.  P: Yeah, it all comes down to finding a balance isn’t it.  R: That’s good so uhm the second question is uhm what are the potential negatives if there are any of using accessercise for improving physical activity levels?  P: That’s the negative uhm no matter how good the app is uhm there is always going to be someone that finishes last isn’t there.  R: So, do you think maybe encouraging people to use this app frequently could cause injury?  P: Well, maybe yeah, I mean just because you’re going to upset two percent of people uhm you have helped the other nighty eight percent haven’t you.  R: Yeah, exactly so there are strengths there are negatives uhm so my final question so if you could summarise in three or four points how do you think the accessercise app could be improved from what you have seen so far uhm if there are anything for it’s use in improving physical activity levels?  P: Well, just the ugh on the filtering thing uhm and I don’t know how you would do it uhm but if you are someone that doesn’t know what all of this means like cardio uhm get toned or whatever if they uhm if it’s the case of ugh scroll down uhm result and I clicked on that uhm search ugh strength and back or strength and leg and that showed you what you needed to do uhm so other way around filtering oppose to choosing that and the other thing was on the explore section for the map uhm providing a link to the gyms website could cover probably most things now you mentioned it.  R: Yeah.  P: They click on it uhm they click on the gym and then just a link to that gym’s website uhm because you will probably find disabled parking and all the other stuff on their own website.  R: Yeah, you mentioned about the reviews uhm and breaking down the reviews as well.  P: Yeah, yeah uhm once again I suspect that you will find most of that information on their website so if you have a link that way uhm it’s kicking the can down the road so you don’t have to do the work but you’re still benefiting.  R: That’s perfect and is there anything else uhm is there any final things?  P: No, I don’t think so. Oh, maybe a key for the map uhm but once again that’s really simple to do.  R: Yeah, and the videos you are happy with?  P: Yeah, the videos are the best feature on the app because you’re getting to see somebody in a similar situation.  R: And you like the fact that in the video is actually somebody with the same disability?  P: Yeah, absolutely!  R: Perfect, ok so if there anything else that you would like to add or discuss before we finish the interview?  P: No, no.  R: Perfect, ok but yeah, I appreciate your involvement in the study Ryan and yeah I look forward to see how you get on with the app and the findings from this study.  P: Yeah, it will be ok.  R: Thank you so much for your time.  P: Cheers. |
| --- |

## Transcript of interview undertaken with Participant 5

**Key code:**

R: Researcher

P5: Participant

| R: Uhm, so first of all I will go through an overview of who I am and the purpose of the research, so I’m James Haley and my role within this research is that I’m the lead researcher and I’m working in a team with Drs David Maidment and Daniel Rhind uhm we are also working with the developers of the Accessercise application and these individuals are called Ali Jawaad and Sam Breary uhm so the aim, importance and the purpose of undertaking this research is that during COVID-19 obviously a lot of people with disabilities were based at home and they could not leave so physical activity levels were being reduced and with the development and evolvement of technology uhm these app developers launched a new app to try and improve physical activity levels uhm and we basically want to see whether users that use the app over a period of three months improve various health outcomes for example fatigue, pain, self-reported physical activity, and so on.  P5: Ok.  R: Uhm, the University that I attend is Loughborough University uhm I’m a Doctoral Researcher and I’m based within the Peter Harrison Centre for Disability Sport which is within the School of Sport, Exercise and Health Sciences uhm I will now go through some ethical considerations so you have read through the information sheet, you have signed the consent forms which is great uhm and as I mentioned earlier any confidential information that you provide will be hidden and no one will get that apart from the research team and all of that data will be deleted at the end of the study uhm and if at any time during the study you want to withdraw you feel like you don’t want to take part please feel free to let me know I will be happy to stop the interview there and we will ugh delete any data ugh I would like you to know that there are no right or wrong answers in this study and do you have any questions that you would like to ask before we continue with the interview process.  P5: No, that’s fine.  R: Perfect, that sounds great. So, the first introductory question that I have got is please may you provide the following information for example your name, age, gender identity, ethnicity and count of residence.  P5: Ok, I am Phil Lucas uhm I am fifty four uhm sorry what was the third thing you said.  R: Uhm, gender identity.  P5: Male and I live in Derbyshire.  R: Perfect, and your ethnicity?  P5: I am white and British.  R: Perfect, awesome. Uhm, so the second question that I have got is to start could you please tell me how you got involved in the Accessercise application and why?  P5: Ugh, I received an email uhm which I was interested in uhm if I can help I certainly will try to uhm there are a lot of people like me so if everyone like me could do this that would be awesome ugh I just thought that it would be an extremely beneficial thing to do.  R: Great, thank you Phil. Have you got any experience of using uhm a fitness application in the past?  P5: Uhm, no not fitness applications uhm I have received similar stuff from physiotherapy.  R: Ok.  P5: Uhm, exercise plans but they are very much stick man drawings and you know if you do not know what you are talking about like me uhm I just try my best to follow what they are trying to get me to do.  R: Ok.  P5: So, over the years I have been provided with those but I have not really applied them to be completely honest with you.  R: Ok and in terms of Accessercise and the advertisement regarding the app uhm have you seen it advertised around before online for example?  P5: No, I haven’t.  R: Perfect, ok. And uhm what would you say is your biggest reason for using the app uhm do you want to improve your physical activity levels uhm but is there any particular reasons why you want to use it?  P5: I definitely do uhm you mentioned earlier with the COVID-19 lockdown uhm really knocked my fitness routine in adverted comma uhm because I just didn’t go out as much uhm I didn’t play sport uhm you know not through choice uhm we were not able to so I definitely need to do something uhm over and above from what I’m currently doing now.  R: Yeah, that makes sense perfect. Uhm, so obviously today is the first day that you’re using the app but could you please tell me your experience of using the Accessercise app and how long you have been a member?  P5: About fifteen minutes I think uhm today is my first time.  R: Haha awesome, so these questions are more focused on the Accessercise application itself so do you currently use Accessercise in your role if so when and how?  P5: Uhm, no I do not use it at the moment, but I will do in the next three months.  R: Perfect, ok. And do you think it’s something that you will use regularly uhm do you think it’s something that you will use once a day?  P5: Uhm, I think I will try to do it on Tuesday and Thursday mornings uhm something like that uhm something that is on a regular occurrence but I will need to make sure that I include this into a regular plan because otherwise if I do not keep a schedule I will not stick to the plan if that makes sense.  R: Yeah, that’s perfectly fine. I think what is quite good about the application is that when you design a workout you can then set a certain day that you want to do the workout and it can then notify you so you can set a timer like if you want to do muscular strength on a Tuesday at twelve the app will notify your phone and give you a little nudge so it’s like please do not forget to do your workout so it provides the encouragement and motivation to do that which is quite good.  P5: Yeah, that’s fine.  R: Uhm, so what were your thoughts when you first started using the Accessercise application so when I showed you the application did anything stand out to you that you liked or you didn’t like?  P5: Uhm, to be honest until I play with the app you know at home I won’t be able to form an opinion until I fiddle with the app haha uhm as I say I’m reasonably open to the ideas you know you’re providing so I will definitely find bits that I enjoy and will benefit me in the long term.  R: Perfect, ok. And in terms of the functions that I showed you like the accessibility option with google maps and how you can design a workout and it has the social side do you like those sides uhm is there anything that you would want to include or remove from that?  P5: No, basically what I want to achieve from this is something personal to me that doesn’t mean that I’m going to go to a gym because I never go to a gym so why would I start now uhm when I have gone to a gym in the past uhm it has been horrendous so by the time that I have adjusted all those different types of equipment to suit me it takes far too long.  R: Ok.  P5: So basically all I’m looking for is something that I can do for myself uhm to improve my personal exercise levels and hopefully that will inform the debate to encourage people similar to me on how to go about it.  R: Ok.  P5: Like I said earlier I’m not really interested in uhm groups or other things because we are all individuals.  R: Yeah of course.  P5: Uhm, there are probably five people with my particular condition in Europe so it’s a really small group haha but as I said I have been involved in disability groups and it’s such a wide thing even in Derbyshire uhm it’s such a wide thing I’m a bit sceptical about designing one app to cover absolutely everything.  R: Yeah, but going onto that point uhm it has the option where you can choose the impairment and the app sort of tailors exercises based on that impairment but do you like the idea how it’s got those individual impairments so obviously you can’t include every disability but they are including a range of them at the moment but do you like that function?  P5: Ugh, there will be some things where it is more applicable than other things uhm obviously if I can save time without going through the whole app that would be good uhm as I said I’m not an expert on this I’m not a PhD student uhm I don’t know what I need to do so if I can be lead in the correct direction uhm you know uhm that is good as I have said I have spoken to occupational therapists and physiotherapists uhm and they always talk to me uhm on a one to one basis and I completely understand what they are saying but when it comes to next week you know I don’t always remember so something that will remind me or as a reference would be good.  R: Yes, that sounds good. So, just going back to the COVID-19 situation, do you think the fact that COVID-19 sort of restricted individuals from undertaking physical activity and now with the development of apps and technology it’s sort of pushing everything in the right direction in a way?  P5: It is because while you were explaining to me earlier I was thinking well all the time I have had conversations with GPs or ugh specialists or physiotherapists or medical professionals uhm if they could show me on a tablet or you know what they are talking about that would make a lot more sense because yes I understand the things that apply to me but I don’t necessarily understand anything else haha.  R: Ok.  P5: So, you know it’s a learning thing for me as well.  R: Yeah, that makes sense.  P5: Uhm, so yeah.  R: That’s great, thank you. Uhm, so I showed you earlier how it’s got the gym, the home and the outside park section to do exercises uhm it’s got those options based on the equipment for that uhm would you say that you have the required equipment to use Accessercise in its full capacity for example access to a mobile phone to use the application?  P5: Ugh, I have got access to the technology uhm I haven’t got any uhm fitness equipment.  R: Ok.  P5: Uhm, I have properly bought some over the years and it’s just sat in the garage collecting dust whereas I said if I can find via the app things that interest me or I can do then I am perfectly willing to you know get involved.  R: Yeah, that makes sense. I think when I first started using the app I said the videos of people doing it uhm and it makes me think like ok going to the gym you have the equipment there but if you haven’t got the equipment at home then how can you do it but when I look through the app in a bit more depth I could see that there is equipment available that you can use from home like a table uhm even tins of beans ugh towels so they are really trying to adapt the app based on equipment at home so people can still do exercise at home even with the cheapest of equipment so it reduces that barrier to physical activity with cost and time and stuff like that so that’s quite helpful.  P5: Yep.  R: So, from showing you the app this morning uhm are there any improvements that you think could improve the Accessercise application further?  P5: Uhm, I don’t really think I’m in a position to say because I have not got much experience of the app uhm but as I said there are positives ugh perhaps if I give an example of the sort of thing that I have experienced where this may help it may help to inform the situation uhm I have got a condition which is quite unique so I got locked in with stroke and uhm other diseases haha ugh which is fine but they don’t really understand me and I don’t really understand them so as I said the individual disabilities you are targeting makes a lot more sense because if there are options available on the screen that don’t apply to me I can then scroll through them uhm you don’t have to sit haha listening for fifteen minutes uhm someone that is a stroke survivor you know it doesn’t really apply to me because I’m not a stroke survivor.  R: Yeah, that’s a true point.  P5: So, as I said I think for most people they really only pick out the bits that ugh are relevant to them uhm and are willing to undertake uhm they are not generally interested in anything else you know uhm that sort of puts me off should I say haha.  R: Yeah, obviously on the app at times it can be quite overwhelming if there is too much going on but on this app it’s quite light in terms of content uhm for example it tries categorising impairments with exercises uhm I think that is quite a good idea but when you saw the exercises with the videos and the description of how to do it uhm did you like that or do you think they can maybe improve a bit more on that?  P5: Like I said uhm can I use the phrase baby steps uhm I would probably start fairly slowly because I’m not very physically active so I don’t really need to jump into doing loads of cardiovascular exercises because I probably will end up having a stroke then haha so yes as I said if I can design uhm a schedule where I do I don’t know half an hour once a day to start with and then you know build it up uhm over time so as I said for this trial three months might be a short time scale for me but over time hopefully I will be able to get into it uhm so yeah that’s what I’m looking for.  R: That’s great to hear I’m happy to hear that. Uhm, in terms of seeing the exercises in person and seeing the descriptions uhm do you think there is too much information on there or do you think there is just about enough where it has got the description where it says this is what it is uhm this is the muscle group you’re working and then it has the tip?  P5: As I said uhm in the past disability covers a wide range of thing so you have to start with one hundred percent of information but as I said most disabled people I have spoken to uhm can pick the bits that are relevant to them so yes you need one hundred percent of information but individual users should be able to reduce it you know the percentage that is relevant to them you know wheelchair users are different from people with multiple sclerosis.  R: Yeah, that makes sense.  P5: So, like I said yeah I think you have to start with all the information but I think the advantage of personalised accounts is that you will be able to tailor it to you know.  R: Yeah, I think it really needs to be tailored because there will be too much going on which makes sense.  P5: Yeah.  R: Uhm, in terms of the videos uhm I don’t think I mentioned earlier but in the videos you watch the person doing the exercises is actually an individual with that specific impairment uhm so if someone has got a spinal cord injury uhm in a wheelchair they will be the one undertaking the exercises in the videos.  P5: Yeah, I think that’s important to have because uhm I have come across videos of exercises in the past and people can do that and they say it’s really easy but they don’t necessarily have the same condition that I have so uhm what is easy for one group of people uhm is not easy for another group uhm so as I said uhm even within my narrow field of medical condition there are differences so uhm people with Spinocerebellar Ataxia can do some things that people with spinal cord injuries cannot do uhm my impression like I said the more specific ugh exercise examples are the better than a general twenty year old totally fit person showing you how to do things that doesn’t always work well with disabled people.  R: So, you like the idea that the videos have an individual with that impairment making it relatable?  P5: Yeah, it’s going to be tricky as I said it’s going to be tricky to get exactly the match for the impairment but it’s more relevant to disabled people to see other disabled people doing exercises rather than trying to mentally adapt it you know to their focus if that makes sense.  R: Yes, that’s perfect I appreciate that uhm I understand what you mean uhm so last question for this section is that do you use any other similar applications outside of Accessercise and if so, what is better or worse than Accessercise?  P5: No, I don’t use any applications.  R: Today could be the day.  P5: Haha, yes, I think so.  R: Haha, perfect so now what we will do is move onto the main bit of the interview which is the think aloud protocol so I will just go through an overview of what that is so we are now going to move onto the main part of this study and ask you to participate in a think aloud protocol while you’re using the Accessercise application uhm the instructions for the think aloud protocol are straight forward I would like you to say out loud whatever comes into your mind ugh there are no right or wrong answers and we will now just run through a quick practice to get you used to the protocol uhm so can I ask you to say out loud everything that comes to your mind when you think about physical activity?  P5: Uhm, haha I’m not fully sure really.  R: So, like any keywords that come to your mind like whether you find physical activity easy or difficult?  P5: Uhm, yeah uncomfortable is the most relevant word uhm yeah.  R: Ok, great thank you. So, we will now move onto the main bit so if I uhm sit next to you and we can go through the app.  P5: Yeah, sure.  R: Perfect, so uhm I would just like to see you uhm open your screen again and go onto the app.  P5: Ok.  R: Awesome, so when you first come to the main screen what do you think are your first impressions when you see that do you like the colour of the app uhm does anything stand out to you?  P5: Yep, no it is fine uhm as I said it’s my first time using the app so I’m curious to what everything does uhm you have explained it to me but I need to have a look through it for myself.  R: Ok, so if you sort of go through what you would do if you were to go through the app if you were at home.  P5: Ok.  R: So, for example, you have come onto this screen.  P5: Yep, basically uhm I will figure out what I need to do uhm as I say uhm there is a filter option.  R: Uhm, so what I would probably start with if it’s your first time using the app is if you go to the main menu uhm and then design a workout uhm so you can go to the my workout option.  P5: Ok.  R: And then you can add a new workout.  P5: Ok.  R: So, when this screen pops up and you have the different options do you like how you have got these different options to choose what your goals are do you like the options that it has gym, home and outside?  P5: Uhm, yeah it’s fine yeah.  R: Perfect, ok.  P5: As I said for most people they will start at home I would have thought uhm so that may be more prominent than the other two options but uhm yeah.  R: I think for example if it only had the option to do exercise in the gym than obviously some people would want to do it at home so the fact that it’s got those options uhm it provides people the option to do it at home which is great.  P5: That’s true but people that want to go to the gym probably won’t use the app haha they will be going to the gym so as I have said I personally think that disabled people need to be encouraged to do things that they are comfortable with uhm which is in the privacy of their own home or within their small controlled group whereas yes a gym is uncontrolled haha.  R: Do you think people are more likely to use the app when they are at home in their own space compared to the gym?  P5: Yeah, I can’t personally imagine anybody that wants to go to the gym using an app.  R: So, you think it’s more focused for home users this app?  P5: Yeah.  R: That makes sense. So, for example if you were to create a workout uhm I would like to see what you do.  P5: Ok, so it’s got the option here about yoga so I will go for that option first.  R: So, I think you need to press the home button first.  P5: Ok, right. Sorry!  R: That’s alright uhm then you can give the workout a name but it’s not essential but you can say something like workout one is for Tuesday’s or something.  P5: Ok, so I will call this workout test 1 haha.  R: Haha.  P5: Ok, yep that’s right.  R: You need to now press the tick.  P5: Ok.  R: Perfect! And then press add exercises so you said you want to do home exercises and if you press the filter option it will come up with more selections.  P5: Ok, uhm.  R: You don’t want the session to be too difficult haha.  P5: Let’s start with light.  R: Ok.  P5: Oh, it’s come up with all sorts of things uhm perhaps I didn’t click it correctly.  R: I think what you have to do is so you have to very specific in the workout you’re doing.  P5: Ok.  R: So, do you like the fact that it’s got the option to uhm choose what muscle component you can work on?  P5: Uhm, yeah like I said I’m not an expert so I wouldn’t know what needs working on uhm like I said uhm for me personally it’s all about core muscles.  R: Ok.  P5: I think anything else is probably a bridge too far.  R: Do you think in terms of the podcasts and uhm maybe the weekly videos that they are looking to do they could include maybe ugh videos on how to train different muscle types and stuff like that or do you think it’s maybe a bit too much?  P5: So, uhm my personal opinion is that from a general users point of view they will start small so I wouldn’t necessarily understand everything you know from the start so like I said the goal is to get people interested on improving ugh you know ugh physical activity because I think most people like me will start from ugh a low level haha.  R: Ok.  P5: So, they gradually work their way up.  R: So, do you think the app over time should include a function that involves graded tasks which start easy and gets more difficult.  P5: Uhm.  R: So, the graded task sort of option.  P5: Uhm, like I said yeah I suppose you could build in a function like that for example you have been on yoga for three months now uhm you may want to go to moderate level yoga you know or come up with a suggestion uhm everything is done with algorithm these days so haha they could probably design one to encourage people to you know move up but I think it’s important to understand as well that most disabled people have got uhm a ceiling that they won’t go much over so yes encourage people but you know probably realistically people get to a level and they become entirely comfortable with that.  R: Yeah, that makes sense.  P5: But any improvement is better than no improvement so.  R: Yeah, I agree with your point. So, you have got the option for example if you wanted to do yoga for let’s say uhm muscle groups.  P5: Sorry, what do I do?  R: I think for yoga if you just leave that option unticked.  P5: Ok.  R: Yeah, and now it comes up with more options so let’s say you want it light and you’re doing it at home, and you want to do it for legs.  P5: Sure.  R: Ok, so it’s coming up with a range of different things that you can do.  P5: Yeah, these are not yoga exercises because it says dumbbells.  R: Yeah, no let me have a little look. Uhm, yoga at home let’s see what comes up.  P5: Ok.  R: I think I must have made an error on the filter option on the app. Uhm, I will need to look into this but let’s say for example uhm you want to build muscle at home and let’s say shoulder and let’s say easy let’s see what comes up without pressing the options down here.  P5: Ok.  R: So, you have chest press, and we can add this to the workout uhm and now you can schedule the workout to a specific date.  P5: Yep, ok.  R: I think it’s very much you just need to play around with all the filters because I’m not fully sure myself about yoga either but if we were to go off this and we were to go down to the social section so obviously you wouldn’t use this but what are your first impression when you use the social side?  P5: Uhm, well as I said yeah I’m not really interested uhm because like I said yeah I’m not really interested because there is a difference between being shown by somebody with a similar condition to you how to do things but I wouldn’t necessarily share personal information with anybody uhm I couldn’t imagine any scenario where I would be interested in telling a friend of mine I did ten dumbbell sets.  R: Yeah.  P5: They are not going to be interested and I think it would be rude of me to say I was doing it you know uhm friends of mine do the whole gym thing uhm I do not use the gym uhm but I do remote gyms.  R: Ok.  P5: Uhm, I do that and like I said I did thirty minutes today which is fine but anything more detailed than that would not be interesting to be honest.  R: But do you think for example during COVID-19 people with a range of physical disabilities would have benefited from the social element?  P5: Uhm, no it’s not really a social element uhm you see uhm it’s cool to have a social element but it’s only somebody sitting and looking at their tablet or laptop or whatever or phone uhm it’s not really social it’s just intrusive.  R: Yeah, I know what you’re saying.  P5: You know ugh people do become addicted to these sort of things you know and I don’t think that’s really a good thing so as I said they are personal aids you know enabling you to get information that otherwise you wouldn’t have but I don’t think there social haha I think they are actually anti social in my opinion haha.  R: Yeah, it makes sense.  P5: Yeah, so like I said that side of it doesn’t interest me at all.  R: Right, so the social side doesn’t interest you?  P5: No not really.  R: Ok, we have done uhm the social uhm we have done the exercises so what I will do is look later on about the yoga and whether I’m doing it correctly because I’m not an expert and I’m new to the app as well uhm but if we go to the Accessibility section so where the google maps function is.  P5: Ok.  R: For some reason when we press on the maps it takes us to Africa haha.  P5: Yeah haha.  R: For example, if you put like London for example.  P5: Ok, yeah.  R: So, obviously here is the all the local gyms.  P5: Ok.  R: So, for example if put a postcode in and you’re staying in a hotel somewhere and you wanted to use a gym uhm you can put the postcode in and it comes up with loads of local gyms uhm do you like the fact that it has that function like do you think there is anything that they can improve on do you think?  P5: Yeah, like I said I never go to gyms uhm I went to about three different gyms uhm and they were all not for me so I would never go to a gym that I didn’t know.  R: Ok.  P5: Uhm, you know yeah like I said if people want to go to the gym but you won’t get me to go to a gym no matter how many times you tell me to go haha.  R: Haha.  P5: Uhm, can I say that a team of wild horses wouldn’t drag me to a gym so it’s no benefit to me.  R: Ok, so for example if we were to press on one of the options so let’s see what comes up so if you press on find a location uhm and you press the little picture.  P5: Ok.  R: Do you think for example someone that wanted to use a gym in a local area have got much options there do you think do you think they could include more?  P5: Yeah, like I said it depends how reliable the information is because I have used the internet for quite a long time uhm since like nighty nighty four and the amount of rubbish that is on there uhm things that used to exist do not exist anymore so yes if it has up to date information yes it is useful but the worse thing for any user is that they click on something and they think oh I can go there and there is nothing there.  R: So, to add onto that point so you have mentioned about like you know the gym might look good and they go there and it’s not accessible is there anything that gyms can do personally to make it a little bit more easier for users to use?  P5: Yeah, as I said they are businesses like any other ugh and there are lots and lots of business types ugh information sources which quickly before uhm redundant because uhm you know high street shops for example they start off with a great uhm social media campaign but then they go out of business haha but it’s still on the internet uhm no one actually bothers to take things down.  R: Yeah, that makes sense.  P5: So for example I used to work for uhm a high street shop and we closed down four years ago and I still get phone calls on my personal phone.  R: Even though they have closed down?  P5: Yeah, even though it’s closed down uhm but they still contact me because at one stage somebody added the company to a database uhm you know and people look on the database and think oh I can sell them a service or whatever uhm but it’s redundant but because I didn’t put the information on the internet I have got no control over deleting it.  R: Yeah, because it’s out there on the internet now isn’t it.  P5: Yeah, as I said yes initially uhm sorry I sort of forgot what we were talking about haha.  R: Haha.  P5: Uhm, so initially yes the information is going to be useful but in six months time it will be less useful uhm in a years time it will be even less useful so like I said you can start off yes it looks good but you know uhm I have got a certain sceptical ugh because there are loads of rubbish on the internet but that’s partially why it’s so big haha there are loads of redundant information.  R: Do you think there is anything that gyms can do to work with the app to maybe provide the users more?  P5: They could.  R: Uhm, so they have the phone option uhm you have the email address of the gyms but could there possibly be a video of the gym itself?  P5: Uhm, yeah again initially yeah a gym is going to be quite receptive at giving advise but they won’t keep the information on there up to date so it will become irrelevant over a relatively short time scale so like I said the only thing from the app point of view is that it could look up databases to see what is closed down and they have then taken that information down you know but it’s a difficult task and no app does that you know.  R: It’s a lot of work for them I guess.  P5: It is so like I said yes there is optimism initially but you know over time they do lose their touch.  R: Yeah, they lose their touch.  P5: Yeah, the overall relevance.  R: That makes sense.  P5: So, as I said yes if I was looking for a gym yes I would use that app but from my experience of not necessarily apps but other databases that have shops and restaurants and hotels you know they quickly become full of things that no longer apply.  R: Yeah. Perfect, ok so that’s the accessibility section done.  P5: Ok.  R: So, if we press the x button in the top right uhm then we have got the more options section.  P5: Yep.  R: So, do you like the fact that it has got a shop, nutrition uhm a blog section?  P5: Yes, uhm as I said uhm I wouldn’t necessarily say I like the shop to try and sell me too much.  R: Ok.  P5: You know uhm ideas possibly uhm as you said uhm incentives maybe for doing things uhm nutrition is quite a big subject so unless you know what you’re looking for uhm I wouldn’t know uhm blogs I wouldn’t be interested uhm podcasts I wouldn’t necessarily watch uhm as I said unfortunately I am from a generation where I do things physically rather than via apps uhm and things like that so yes I wouldn’t necessarily look at them.  R: Do you think the fact that it has got achievements could be a good option for example providing users a streak for how many times they have used the app?  P5: Yeah, targets setting is never a bad thing uhm so like I said uhm realistic target setting you know I’m not going to suddenly turn into Arnold Schwarzenegger uhm I’m never going to get there even in a million years but if there was a way that it knows my weight uhm sorry if I know my weight but if over time you know it leads to weight loss that’s great that makes everybody feel better and also you can say to your GP oh look this app on my tablet says I have lost a stone over the year or whatever so from that point of view yeah it might be good uhm as I have said there are vast improvements to technology over the last thirty years uhm something such as medicine hasn’t even scratched the service to be honest.  R: Yeah, that makes sense.  P5: So, anything that encourages individuals to set better targets for themselves and then over time that can feed into medical professionals to hopefully prevent you know or at least help conditions that people already have you know because as I said most people don’t know what exercise is valuable so it would be good to be able to set targets towards a better place for everybody.  R: Yes, that’s a good point I agree with that. I think setting targets and things are always quite good and just the last little bit, so you have got the account settings.  P5: Yes.  R: So, if you press account settings uhm so if you press the account settings here it has the impairment filters where you can choose your impairment.  P5: Ok.  R: So, if for example for some reason it is on the multiple sclerosis section you can change it to whatever impairment you have uhm do you like the fact that it has got the exercise habits and you can select the measurement units do you like this section here?  P5: Yeah, as I said uhm providing it’s uhm secure right haha I’m happy to put information on here to get a better outcome so you know generally I don’t mind sharing information uhm that leads to something better uhm what I don’t like is you know inputting information and it then gets put into a database as I said I’m sceptical of this sort of thing but you know as long as I’m convinced that it’s secure information than yes I would definitely uhm to use the word again haha target a better way of achieving what I want to achieve so yeah.  R: Ok. Perfect, sounds great. So, uhm we will now move onto the overall thoughts so thank you for going through what you think about using the app now that’s great so this section is mainly about the positives or the negatives about using the app so what do you think are the potential positives if there are any of using Accessercise for improving physical activity levels?  P5: As I said for most disabled people in my experience we tend to do nothing unless someone pushes us with a sharp stick haha so the sort of uhm you know the advantage is that having technology let’s say it’s Tuesday morning and you really ought to be doing yoga or exercise of some kind whereas in the past you know that wouldn’t happen until somebody physically knocks on your door and said you know it’s Tuesday morning you should undertake more physical activity and this is what we do uhm so like I said yes from that point of view it’s a definite advantage uhm you know individual responsibility can’t be taken out of the equation so if people don’t want to do it uhm they won’t do it but yeah people like me who are aware that I should be doing more uhm yeah it’s definitely going to help.  R: Perfect, ok. And what do you think the potential negatives if there are any of using Accessercise for improving physical activity levels?  P5: As I said the negatives apply to any technological advance uhm across the board so there will be some people for whom I don’t think it will probably work out to be a good thing using the app but as I said it’s a free country we can all do these things uhm we can go to gyms or not go to gyms in the same way we can use the app or not use the app so like I said targeting use yeah definitely beneficial.  R: Do you think for example the cost of the membership to use the app might be a problem for some users?  P5: Ugh yeah I don’t think high costs will ever interest people in using things uhm like I said can we integrate the system of the app into things like care plans.  R: Oh, ok.  P5: Yeah.  R: I was a carer for seven years.  P5: Yeah, I used to work for social services so there was a big problem around personal care plans uhm as I said for most people my age it would be more difficult to use technology but that doesn’t mean to say we shouldn’t be doing it uhm we should be doing it because the cost of actually employing a personal trainer is quite high whereas an app can be adapted to the individual for a relatively cheap price so like I said cost of the app may be an issue uhm I wouldn’t necessarily subscribe to it ugh but if it was incorporated into a care plan then yeah.  R: Perfect, yeah. Obviously, it’s an important point to try and reduce the cost of everything uhm so if it’s possible then go for it.  P5: As I said most expenses is preventable haha it all comes down to society as a whole uhm we cause problems for ourselves haha especially when it comes to expenditure so if we can nudge people into a different behaviour pattern then that’s got to be a good and I don’t mean something like big brother or anything.  R: Good point though haha I know what you mean it’s ugh a tricky one because if you can include the membership into care plans to reduce the overall cost and stuff it’s always a benefit.  P5: Well like I said if there are some benefit which is relatively low cost I think we definitely need to take advantage of it.  R: I agree and that makes sense. Right, so last question I have got is uhm how do you think the Accessercise app could be improved if anything for it’s use in improving physical activity uhm so if you had to give three you know points on what you have seen so far is there anything that they can really focus on?  P5: Uhm, I don’t know enough about the individual or the unique attributes of this app to comment at the moment as I said hopefully when we do this again I would have used it and you know I can work out things that I would like to say or things I don’t really think are relevant.  R: Oh ok, awesome perfect and is there anything else that you would like to add or discuss?  P5: No, that’s fine thank you.  R: Perfect, thank you Phil I appreciate your participation and thanks for the interview.  P5: Ok. |
| --- |

## Transcript of interview undertaken with Participant 6

**Key code:**

R: Researcher

P6: Participant

| R: So, thank you Gary for taking part in this research study and getting involved in using the Accessercise app uhm so I’m James Haley and my role within this research project is that I’m the lead researcher so I will be undertaking both phases of the trial and also undertaking the think-aloud interviews in the usability uhm data collection uhm the aims, importance and purpose of undertaking this research is that uhm we would very much like to understand how good this novel smartphone application is at improving physical activity and various other health outcomes for people living with disabilities uhm because obviously with COVID uhm individuals with disabilities had to stay at home due to lockdown and stuff like that uhm there wasn’t much physical activity going on so with the improvements and the developments of technology we would then like to see ok how good is this new app at actually improving physical activity.  P: Sounds very useful at the moment.  R: Yes, I agree haha. So, the University that I attend is Loughborough uhm I’m a Doctoral Researcher undertaking PhD studies uhm I’m involved within the Peter Harrison Centre for Disability Sport within the School of Sport, Exercise and Health Sciences uhm and I’m being supervised by Drs David Maidment and Daniel Rhind uhm and the other individuals that will be working on this project is Ali Jawaad and Sam Breary which are the developers of the app uhm any information that is collected within this study will be confidential so for example names, dates of birth and anything else that you would mention that is personal will be confidential and hidden for this research study uhm you have read the information sheet and you are happy with the consent forms you have signed them which is great uhm if at any time throughout the study you want to withdraw uhm because you don’t want to participate and you want to stop then please let me know and I will be happy for you to stop uhm and there are no right or wrong answers in this study ugh do you have any questions that you would like to ask before we continue with the interview?  P: Nope, I’m happy to proceed.  R: Perfect, thank you ugh Gary. So, first of all we will go through some introductory questions so uhm the first question that I have got is please may you provide the following information so your name, age, gender identity, ethnicity and county of residence?  P: Gary Spratt, ugh did you say date of birth?  R: Age.  P: Ugh, fifty.  R: And your gender identity?  P: Male.  R: Ethnicity?  P: Ugh, white British male.  R: Perfect.  P: And uhm county of residence is Northampton.  R: Perfect, awesome. So, to start could you tell me how you first got involved in the Accessercise application and why?  P: There was an email sent out from the head coach at Northampton Saints called Jamie Higgins ugh about people who had ugh spinal injuries and other disabilities who may benefit from taking part in this study so uhm I contacted uhm to see whether I was an eligible candidate uhm if I met the criteria uhm for me it’s all about giving something back and things that can help uhm myself and others uhm willing to give it a go.  R: Perfect, thanks Gary. So, the third question for the introductory section is uhm please could you tell me your experience of using the Accessercise app and how long you have been a member? So, do you have any experience of using the app uhm have you heard of it before hand from anyone?  P: Never heard of it before uhm today is the first time uhm actually seeing it uhm and yeah it’s nice to see some of the videos that are already on there.  R: Perfect, ok. In terms of people from your ugh club that you are involved with now do you know if anyone is using the app or?  P: Uhm, I have not been there for two weeks because I had birthday celebrations and then I went away uhm for the six nations rugby but ugh I am going back on Monday uhm but it will be a case of uhm catching up to see if uhm others are using it.  R: Ok.  P: I did see a wheelpower ugh forum that they were advertising about the study on there as well so it was another forum not just in our club that have mentioned the study.  R: Perfect, ok. And just going into your general physical activity levels and what you do day to day uhm would you use an app to go to the gym uhm is it something that you would look to use or would you go to the gym and not really think about using it?  P: Uhm, I think with having my spinal cord injury from last year uhm weight has massively uhm creeped on so I’m looking for something one to give me motivation ugh to help and assist me because obviously going from ugh a T1/T2 uhm injury uhm is all about learning what I can do uhm as opposed to what I can’t do so looking for things that can help me burn weight and ugh improve ugh muscle tone as well.  R: Ok, awesome. So, if we go back to sort of during COVID time ugh did you use any apps during that phase or are apps something that you don’t really think about using and if so why is that the case?  P: Uhm, with COVID uhm I was classed as clinically vulnerable so I didn’t really see anyone for eighteen months uhm inside or outside apart from the window uhm waving uhm so I didn’t ugh use an app personally but my son uhm who is massively into fitness has used apps uhm tracking calorie counting uhm and reporting all of his fitness and things and for workouts that he has done to improve uhm his fitness prior to competitions.  R: Ah, ok so your son uses apps as well?  P: Yep.  R: Do you feel like your son could help you maybe use the app further?  P: Most definitely uhm he is yeah a wiz on apps and also on fitness as well uhm so you know I think he will definitely assist uhm as well and you know we have a couple other types of PT instructors within the club uhm who could again uhm assist to see what’s on the app and what things they are going to add uhm using their knowledge as well uhm of you know PT uhm and yeah we have a lot of youngsters who play uhm yeah who will be a bit more advanced when it comes to technology as well so I think you know there are lots of feedback that can be collated from me using the app in the club with other people assisting.  R: So, do you feel like people from your club would be wanting to use an app if it’s obviously effective at improving physical activity?  P: Most definitely! Uhm, you know we started of playing for fun uhm but as competition and becoming more successful it’s all about how can we improve uhm and you know physical fitness uhm will play a massive key in progressing the team uhm and getting through challenges uhm you know quicker, leaner, faster, stronger uhm in competitive sports it will make a massive difference uhm you can even look at say mainstream rugby how it was back in the eighty’s you know beer drinking and things like that and now they are like super athletes uhm it is the training, the coaching uhm the game is sort of still played the same way and the rules haven’t changed massively but it’s the players who are basically taking on new challenges for example if you look at the developments in science and technology and how they have moved forward and how they were is like chalk and cheese basically.  R: Ok, awesome so going back to the app obviously so there is this new app that has been developed called Accessercise but why do you think maybe people from your club haven’t used an app before uhm so do you think it’s something that they haven’t really known about?  P: I think it’s not because they don’t know about it but I think it’s because there are not many things out there for disability uhm it’s all for you know for able bodied there is nothing specific for uhm disabilities and certain disabilities uhm you mentioned spinal injuries uhm which is great for myself you know but if I was to go onto a normal app and I have a spinal injury there is not going to be many things uhm it is going to be generic for an able bodied person uhm it’s not going to be tailored to assist me in my specific injury so for me that is fantastic.  R: So, do you like the function on the app where it’s very much you select your impairment and then the app sort of creates exercises based on that impairment so it’s very individualised?  P: Yeah, because you know when you have a spinal cord injury your life changes massively uhm about what your limitations are now uhm they are different for everyone but having a more generic approach to a certain disability will be more beneficial because you can use the information and the videos to assist you in your recovery to get stronger, healthier uhm weight loss particularly in my uhm case so it is a good way to assist a spinal cord injury.  R: Yeah, I think what’s quite good about this app is that you can select your impairment and then it creates exercises based on that impairment whereas I think other apps maybe don’t have that function they just have generic exercises, and you think to yourself well maybe I can not do this maybe I can.  P: Yep.  R: So, I think what I like about the app from using is that it has that function where you know if you wanted to build muscular strength and you have a spinal cord injury then these exercises might be suitable and you press onto it and you can add specific exercises to your workout plan so it is very tailored to the individual because obviously every disability is different and every person is different so if we can get the app very much focused on the individual itself it can then you know be very beneficial to them and not just everyone and anyone you know.  P: Most definitely.  R: So, I think that’s quite important. Right, so we will now move onto Accessercise specific questions uhm so do you currently use Accessercise in your role and if so when and how?  P: It will be starting from today.  R: Ok.  P: Uhm, I shall have a look more into detail once I have set up and putting in my injuries and what exercise I can do and then tailor in the plans.  R: Perfect, so on the app it has got an option where you can select where you want to do the workouts so that can be in the home uhm in the gym ugh it could even be in the park uhm are you more likely to use it at home or in the gym or?  P: Uhm, well I have just moved into a gym close to work uhm but I think to start of with I will most likely do it at home.  R: Ok.  P: Uhm, because it is also tying in with my fatigue levels as well so uhm I think until you can sort of gain that confidence back and going out into a gym to do these things I think doing it at home will be my first approach before moving into a gym but I did like the explore function especially because it shows gyms that are accessible uhm which I think is a lovely ugh idea because you know I have just come back from Cardiff and some of these bars and things I couldn’t get in there well because there are steps and things you know so it’s a waste of my time going there because I can not get in there but it’s nice to see that there is an option that if I go to this gym it is recognised as being fully accessible so.  R: You like that function?  P: Yeah, I do.  R: Because I think what the app does is very much people can review like other types of gyms and if it’s accessible they provide ratings and the app sort of understands like ok this gym is accessible but this other gym isn’t uhm and you press on it and it provides information uhm on that gym itself but do you think there is anything in particular that you would like from that function to be added or do you think maybe removed or anything that you particularly want from that function?  P: Uhm, I think once you start going to the gym’s and with the review’s it’s what machines or things that you can or can’t do uhm it make be accessible however it may not be fully accessible.  R: Right, ok.  P: Uhm, so it could be uhm a review of the gym looking at you know uhm what machines can work and what support is available as well.  R: Ok.  P: Because sometimes it’s good to have that uhm good relationship with staff as well because if you were struggling or need a bit of help or something with a machine because depending on what type of spinal cord injury you have will impact on how you use the machine so it could be with the review that you know the staff are happy and kind of like you know move the keys down on the weights or move your chair out the way uhm so there are some additional help that could promote that gym more.  R: Ok.  P: Uhm.  R: I think on the app there is a function that says do you require additional assistance but I think at the moment when you press yes it doesn’t actually do anything which I think it’s something the developers are working on but for example if you were to press the yes button is there anything in particular that you would want the staff to do uhm would you like want them to be notified and for them to come over ugh or anything that you think the app could include for that?  P: Uhm, I think notifying uhm you know because some of these gyms are twenty-four hours you can easily hit a button and go in type of thing and yeah it would be nice because with the sign up to the gym it could have a function where it knows that there is a user around that may need additional assistance uhm on site so if there is someone at the gym they may be able to keep an eye on that person would be more beneficial or actually speaking to that person or what actual support that they need.  R: Ok.  P: A lot of people are stuck in their ways that they can do it by themselves and they don’t actually want help uhm as opposed to you know we do need help and it’s actually having that conversation uhm what support you actually need from that gym.  R: Because I’m thinking with the app if there was an option where you can press you know like I require assistance whether the app developers could link up to local gyms where for example uhm you pay a bit more on the membership then that money goes towards someone at the gym that knows that someone is coming into the gym with an impairment and so when you press the button then the member of staff instantly comes over.  P: Yeah.  R: Sort of like an assistant.  P: Yeah.  R: I think that would always be quite good but going back to what you said about the equipment and obviously pressing on the gym and you can see a list of all the information regarding the gym uhm would you like to see for example uhm a checklist of all the accessible equipment that is available there?  P: Yeah, or even like a three sixty view so you can actually see the gym and the layout as well.  R: Oh, ok.  P: Uhm, a lot of places are now doing like you know virtual tours uhm.  R: Oh, another participant said something similar the other day.  P: You know for example when you go to hotel or use similar accommodation you do uhm you know a virtual tour so you can actually see the layout and things uhm and then if you have questions you are sort of prepared or you could drop that gym an email saying that I have noticed a few issues uhm but will it still be fully accessible for my needs uhm if not then I can go to another gym on the list ugh so you know but I think a three sixty few will give you a better idea of what you’re going into beforehand.  R: Yeah, I think that’s a good idea I think obviously if you were to go to London for example and use a gym down there for a few days you would want to see how accessible it is rather than going there you know and finding out it’s not accessible and you have to return home because you can not use the equipment and stuff so I think that option is pretty good but in terms of obviously pressing on the gym seeing the other options uhm do you think there is anything else to include so you have got the contact details you have got the opening hours uhm you have got the rotation option that you want but is there anything in particular that you want from that accessible feature on there?  P: Uhm, I suppose a question box where you can ask the gym questions that would definitely help me.  R: So, like a live chat sort of thing?  P: Yeah.  R: Ok.  P: Or even like a standard q and a with the most asked questions with answers for that particular gym?  R: Yeah, that makes sense that’s a pretty good idea. Uhm, so what were your thoughts when you first started using the Accessercise application so when I showed you it did anything stand out that you liked from it.  P: Uhm, when it came through I liked the option to choose exercises and having the videos there uhm for someone that is new to using the app and new to a spinal injury uhm to actually see some exercises and seeing it in motion uhm so you are seeing what you are doing as opposed to you know a seated band uhm arms.  R: Yeah.  P: Yeah, so it comes down to how you are doing it and the goal of doing that so for me it’s actually seeing uhm the demonstration what it’s doing and how you are supposed to be doing it as opposed to a generic wording.  R: Do you like the fact that you can press on the exercise and it has the video and it shows you how to do it.  P: Yeah.  R: Because when I think about uhm the barriers to physical activity I think sometimes the lack of knowledge of undertaking physical activity and I think for some individuals they won’t know how to workout or go to the gym so I think the fact that there are individual exercises with those videos are quite useful uhm so it has got obviously the videos it has got the subtitles and stuff like that but do you think there is anything else that they can include or work on?  P: No, ugh you know for me uhm having used gyms previously but to see uhm you know from a generic point of view people that have not been to gyms ever and they have a spinal cord injury uhm to see you know how these exercises are going to help them and why they are doing it and what these are doing to help them achieve their goals.  R: Ok, perfect that makes sense. Uhm, do you have the required equipment to use Accessercise in it’s full capacity for example access to a mobile phone to use the application?  P: Yep! I also have a tablet as well so.  R: Ok.  P: They are all quite well and linking in with one another as I use apple products uhm I have a smartwatch that will start measuring and looking at uhm blood pressure and heart rates to obviously keep a tag on things uhm which may give me an overview of my cardio so yes.  R: I think what is quite good about Accessercise is that you do not really need to be connected WI-FI or the internet to use it so for example if you were to go to the gym and you ran out of data you can still use the app because you can use it offline so I think that function is quite useful.  P: Yeah.  R: Ugh, I like how it has that uhm last question for these specific questions related to Accessercise is do you use any other similar applications outside of Accessercise and if so what is better or worse than Accessercise so maybe from your previous experience or maybe using an app uhm was there anything that was different on that app compared to this app or maybe this app has better than the other app?  P: Uhm, I only used uhm like a fitness training app many years ago uhm so this is definitely a lot better in my opinion.  R: Ok, awesome. So, we are now going to move onto the main part of this study and ask you to participate in a think aloud protocol while you are using the Accessercise application so what I’m going to do is I’m going to see you going through the app and maybe I will ask you questions about what you think about the videos and what you think about you know the exercises provided uhm so the instructions for the think aloud protocol are straightforward I would like you to say out loud whatever comes into your mind uhm there are no right or wrong answers and we will just run through a quick practice question to get you used to the protocol ugh so could I ask you to say out loud everything that comes to your mind when you think about physical activity?  P: Uhm, equipment ugh timings uhm the uhm areas you are working on so will it be back, chest, arms uhm yep.  R: So, these things for example are things that you might find difficult or are these things you relate to when it comes to physical activity?  P: Things you know I would relate to when it comes to physical activity or exercise you know uhm I think timings is a massive thing and am I doing it right and in a correct way uhm to actually get the benefit of doing an exercise because your interpretation uhm of what you think compared to how you are going to see it being done correctly uhm because you could be putting thirty minutes of work in and be absolutely shattered with fatigue but when you actually see the videos like uhm was I even doing it right in the first time and you spent all that energy to not actually have any type of benefit whatever.  R: Yeah, that makes sense and going back to your point on equipment do you think there is a general equipment issue uhm because for example if I was going to play wheelchair rugby, I know there is issues with you know getting the wheelchairs to be able to use them but maybe is that an issue that you have experienced in the past?  P: Ugh, yeah getting chairs is difficult because they start around six and half thousand pounds uhm but a lot of clubs do have chairs which are provided by GBWR uhm a lot of people will self fund uhm and you know for the actual chair itself yeah it is uhm very expensive but things like resistance bands uhm wheelpower are giving them out to anyone in a wheelchair so when you have someone in a wheelchair so they can contact wheelpower and they will send them out and they will do different strengths and colours and ugh different yeah resistance bands uhm I think they have six different colours to assist people undertake physical activity.  R: Oh, ok. In terms of the self-funding have you had any experience of self-funding a wheelchair uhm is that quite difficult thing to do?  P: Yeah, you know because when you have a club uhm everyone is self-funding uhm then it is difficult to you know you have five players you know it’s kind of like best part of forty grand trying to find uhm that sort of money is difficult and they are all from the same club.  R: Right, ok.  P: And there is only so many ways that you can do raffles or you know race nights or whatever because everyone is in the same boat uhm so it is very difficult to get events up and running to get funding uhm so yeah some clubs have struggled but we have been quite fortunate because one person one year where a new player has come in and enjoyed it and fortunately my club have savings and things you know they can go and get the chairs uhm so I think yeah it can be difficult for a big club with many players to get that funding.  R: Do you think that is something that puts people off maybe using the app when they see for example doing exercises that involves using weights for example the app has the option where you can go to the gym and use equipment but if you don’t go to the gym and you are meant to work out at home do you think people will be put off that they need the equipment to use the app?  P: Uhm, you can do things around the house you know whether it will be a pair of tights or a tin of beans there are things that you can do as an alternative uhm it could be that you know an option could be an alternative if you don’t have you know a small dumbbell instead you can use a tin of beans you know there could be an alternative because not everyone has the funds especially because of the cost of living to go out there and buy these new types of equipment and things uhm but if there are items around you know the home that could be as an alternative that could be good.  R: Yeah, that could be a good alternative. I was thinking like the app is very good if you are at the gym but some of the videos might be focused on weightlifting and if you’re at home and you haven’t got the weights so an alternative maybe they could include on the app that you can use a tin of bins at home and stuff like that but there may be a health and safety issue.  P: Exactly.  R: Uhm, so that is something that they could sort of include uhm so if you haven’t got the equipment for this reason then maybe you may want to consider this item that you can find at home.  P: Yeah.  R: That could help and support so yeah. Perfect, so what we will do is uhm if you show me sort of what you have been looking at so uhm as you can see on your screen you have your exercises uhm you can also favourite uhm exercises so if you have an exercise or workout that you like you can then obviously favourite it.  P: Yeah.  R: Uhm, you have my workouts so you can ugh create a workout so if you press add a new workout uhm so what do you think about all these functions available, so it obviously has the option to choose the location that you want to do it.  P: Yeah, because again it is going to know that if you are not at the gym but you’re at home uhm it’s going to filter the exercises based on that so it won’t provide you all the big machines and things uhm it gives you the option to choose what you want to achieve so whether it will be you know cardio, strength uhm muscle ugh get toned ugh stretching, flexibility and it also has the timing function so how long because sometimes you may only have twenty minutes you know I suppose it will help you definitely set the timer for you and where you can actually do the exercises and you can even provide your workout it’s own name as well so you know what it is for you and for that reason it’s more personalised.  R: Yeah, that makes sense. So, if you could for example uhm create a workout as if you were doing a workout at home uhm so you are working out at home, you want to get toned and let’s see uhm what exercises you can select for that. Uhm, so on this app there are two hundred and fifty exercises available so it has got quite a range.  P: Ok.  R: But the best way to choose the correct exercises for you is through the filter and look through everything that way.  P: Ok.  R: Uhm, so for example this comes up with obviously doing it at home uhm cardio uhm whether you want to do arm, shoulders, chest and that sort of stuff uhm.  P: I like that it has the assistant and carer uhm option as well uhm that is good and obviously the intensity of the workout and how hard you want to make it for yourself.  R: So, I guess if you wanted to do like quite an intense workout then you can obviously select hard uhm I want to build muscle uhm and do it at home.  P: Yep!  R: Then it can help filter the exercises that you want to do.  P: It has the option on here for coffee table, which is interesting and cans of beans, towels and so on so there is these options.  R: I always think that when I first use this app so ok so a lot of the videos are tailored for people that are sat in the gym you know working out there but with home these people may not have the equipment so the fact that it has got coffee table there, pullup bar ugh what do you think of that do you like those options?  P: Yeah, I do like that because uhm most people will have a rucksack at home you know so simple things to uhm.  R: What is good is obviously the cost of equipment will be quite expensive so the fact that you know you can use a coffee table at home, and you are not spending any money to use it.  P: Nope.  R: So, it is possible to use equipment for the exercises on the application you know.  P: Yeah.  R: So, this is a pretty good filter option so for example if you were to use this app uhm and you couldn’t find an exercise then my biggest advice would be to go through the filter section so for example if I show you here you are at the gym, you want to build muscle, you want to focus on chest then this will help you filter into that exercise that you are looking for.  P: Yep! No, I like that.  R: Do you think there is anything else that they can possibly add into this section do you like the colour of the app?  P: I think the colour that they are using is too much of a light grey against a white background it probably may need to be a bit standee outty.  R: Right, ok.  P: Uhm, the purple is fine uhm I think the grey may need to be a little bit darker uhm, so it stands out a bit more from the screen.  R: Right, ok. But do you like the way that the app has laid out all the options on the screen or do you think it could be sort of more concise and like less information because I do know some people prefer information all on one page and not dragged off onto separate pages.  P: No, I suppose the only thing you could do is to make the filtered options alphabetical uhm because if you look at the outdoor training uhm all the items are within that one section so you wouldn’t have all the items up at once so outdoor training you have the open drop down boxes and everything you need is there uhm you want to make everything specific to where you want to work out and have the options filtered based on that uhm but yeah.  R: Do you like the option for choosing goal for example being fit, building muscle uhm do you like uhm the little uhm emoji’s that they have used next to these?  P: Yeah and getting toned as opposed to weight loss is a better terminology uhm we all know what it means but yeah again splitting it down to uhm the core sections that you are looking for especially from a wheelchair side uhm shoulders, back, lats, chest is really good uhm so yeah.  R: It also has the carer function.  P: Yeah, I do like the carer option that may be beneficial for some users uhm so.  R: I think what is pretty good with that one I think for example if you select that option ok, I think it provides options uhm maybe not but I think that’s because the app is still in the development phase, and they are continuously adding new components to the app.  P: Yeah.  R: Uhm, but I think on the app there is many things that they still want to include but they are still working on it.  P: Yep.  R: But I think it is good to have that option with the carer because when I think of for example someone with a spinal cord injury they go through the rehabilitation route where they are more likely to work with a physiotherapist or a family member so you know having that option and it filters exercises based on that is quite effective.  P: Yeah, and the safety point too as well.  R: Yeah.  P: There is no point trying to do something and you kind of like uhm get pinned down with something and you are sort of stuck there until someone comes over.  R: So, going back to the whole carer thing and what I mentioned about you know the option where you can get assistance from the gym so for example instead of maybe a care company spending money on a carer that will have to go in with the user do you think there is more that the gym could do to provide extra care for those individuals coming in?  P: You would like to think so uhm it’s a fantastic promotion opportunity uhm for them uhm you know and definitely a unique selling point uhm yeah definitely can you imagine you know all of our gyms offer this for all disabilities that would be amazing uhm and I think that would be yeah a massive selling point because we look at some of the gyms for example we have a gym in Northampton which is so much more expensive uhm and it doesn’t have half of the things that maybe a pure gym or a virgin would provide.  R: So, it’s more expensive and has less equipment?  P: Yeah, it’s more expensive and has less equipment uhm and it’s you know pure gym is around twenty pound a month uhm and the one in Northampton I mentioned is like thirty-eight pound a month starting.  R: Does that come with a swimming pool and a sauna?  P: No, it doesn’t provide that much haha.  R: Haha.  P: It very much has basic equipment.  R: Is it a private company though?  P: Yep, and it’s kind of like you know uhm when put the word disability in things just rocket up and get so much more expensive it’s unbelievable.  R: But do you think it’s a lot more expensive to use the gym with a disability do you think just because of the extra demand on the equipment and resources around that?  P: Yeah, anything to improve disability and disability sport uhm equipment yeah it’s very expensive for example adapted cars uhm a lot of people will have automatics uhm you are looking at advanced payments like an additional nine hundred pound because you want an automatic and it’s kind of you know a different disability does not need to have one uhm you can get like zero advanced payments and as soon as you need more disability equipment and things especially automatic then yeah it’s unbelievable.  R: An automatic car?  P: Yeah.  R: Yeah, I can imagine.  P: You are looking at around nine hundred pound added on because you need automatic hand control and so on uhm and it’s crazy.  R: I always feel like the government doesn’t realise the cost of these things and they should really be working harder to support individuals with disabilities.  P: You know you look at a wheelchair for example a rugby wheelchair costs around six and half thousand as a starting price uhm Casey Jamie’s son has just bought his new chair uhm and that cost eleven and a half grand.  R: Gosh!  P: And that’s just an average chair.  R: Is that just a wheelchair rugby chair or is that a general chair?  P: That’s his wheelchair rugby chair.  R: Gosh.  P: So, yeah he took out uhm a small loan to pay for that and the chair is his pride and joy.  R: Yeah.  P: Now you look at it and you think oh my gosh it has scratches and marks and everything you know uhm you could have a brand spanking you know second hand car for that cost.  R: Exactly.  P: And people are smashing into it left right and centre.  R: Yeah, but that’s his pride and joy though isn’t it.  P: Yeah, uhm and again you know the inner tube uhm the tyers is an additional cost uhm with the sport.  R: Yeah, it can easily become quite an expensive hobby.  P: Yeah, it’s uhm not the cheapest thing to do.  R: Yeah, gosh. Uhm, ok so if we go back onto the app and for example if you select uhm a workout we will look at the individual exercises itself.  P: Ok.  R: So, what’s your thoughts when you see the exercise video you are on now?  P: Yeah, very straight forward uhm it shows you ugh a demonstration on where the band needs to go and also the start and ending positions.  R: So, for example if there’s a few exercises that you wouldn’t know how to undertake uhm do you think this option is something that you would click on and watch before you do the exercise?  P: Yeah, I would do that before adding the exercise to the workout uhm because there is no point adding something into your workout because you know without the videos can you physically do the exercise yourself so uhm the videos show you what the exercises are going to look like and then you can tailor that towards what your limitations are.  R: Ok, that’s awesome. So, as you can see it says completed so I’m assuming that when you finish it will say completed and you will be able to see on your list that you have completed that workout.  P: Yeah.  R: But if you were to go for example back to the home menu.  P: Yep.  R: If you just press on one of the options for example assisted bandit and not the actual video uhm and you press show more uhm and it shows the key benefits of the exercise uhm do you like what you are seeing on the screen now?  P: Yeah, it’s showing you what muscle groups the exercise is working uhm on there.  R: Do you think there is anything else that they could potentially add do you think or what they have included on there so far is good?  P: It’s ok ugh so from what I am seeing on this page is stands out more compared to when we looked at the filter page uhm this definitely stands out a lot better against the lighter background.  R: Ok, so you think the colours that the app developers have used is something that they need to work on?  P: Yeah.  R: Ok.  P: Uhm, for the filter section page most definitely uhm because they really need to pop out to me uhm because the yellow and purple options that I’m seeing now really do stand out.  R: Oh ok, great thanks. And do you like the key benefit section uhm do you think maybe they have enough there or do you think they need more?  P: No, because you know what muscle group you are working based on the description uhm you have very much tailored it to what you are looking to achieve.  R: So, what are your thoughts for example when you first look at the exercise do you find there is too much information available that may distracting or do you like that it’s simple that it has the videos uhm it’s got the descriptions the benefits and so on?  P: No, I like it because you there are so many disabilities, and you need to focus on a range of things uhm so having the video uhm having the wordings coming up ugh the hearing and the visual from both the words and also seeing the exercises uhm it’s really typed out for you uhm as well because a lot of uhm things you will see like I have from the physiotherapist all I had was this is just words.  R: Ok, so not videos or anything like that?  P: Just words no videos ugh so to actually see a video actually putting them words into practice is definitely a benefit.  R: It also has a useful tip at the bottom which is quite nice it has a blue so the mixture of colours is quite nice but I know what you mean about the first page being quite bland isn’t it it’s not very colourful.  P: Yeah, but again as you are getting stronger uhm it is showing you how to make the exercises more difficult so you are getting more benefit from it.  R: Yeah, so for example going to that uhm more difficult section for example if I do the exercise and I have finished it uhm do you think there needs to be graded tasks on their so for example once I have completed this exercise then there’s more of a challenging exercise next?  P: Yeah, I think also probably uhm maybe a pre workout and a post workout option.  R: Ok.  P: Because uhm you know you should never go straight into a gym session without stretching and you should also have a cool down as well so.  R: Ok.  P: Maybe uhm some basic stretches could be incorporated so any kind of workout that you do it is going through some basic uhm stretches because if you do not stretch properly, you suddenly experience some pain.  R: Yeah, and your workout will not be good then. So, you are saying for example before you jump into the main workout uhm it should include a little pre workout bit where you can do some exercises uhm you can do some stretches and so on.  P: Yeah.  R: Ok, that sounds good.  P: Uhm, and obviously when you are cooling down so again you know uhm to avoid any doms or anything like that uhm for example if you have done a massive workout uhm and maybe go through levels uhm so for example you complete like light and you go to moderate and then to medium so again you are going through in stages uhm to build yourself up uhm because you know we always say to ourselves this is where we are here but really we are much lower.  R: Exactly, haha.  P: We find ourselves going straight into something and then the next day you can’t move haha uhm everything is hurting and killing you whereas you know what I should have been more realistic and started at a lower level and worked through different levels.  R: So, progressive levels of the exercise?  P: Yeah, it could be a way that again avoids or even reduces any types of injuries because you know your arms and shoulders you know are not going anywhere.  R: Haha exactly. Ok, so if you press the calander function let’s see what that does in the top left.  P: Yep.  R: If you press on a date for example you can then add the workout to that day.  P: Yep.  R: For example if your workout is all based about building uhm muscle which we worked through and if you wanted to do that next week then you can select that uhm but overall what do you think of this function do you like the colour uhm do you like the way that this section is laid out?  P: Yeah, because you can then target what you want to achieve uhm with the exercises and it’s giving you that kind of ugh option to basically say today I’m focusing on legs uhm tomorrow is back day the following day is chest and you can then build it into your routine so yeah.  R: Because my initial thoughts are when I saw that was like if you spend Monday doing legs uhm if you spend Tuesday doing chest uhm you can design the workouts based around what you really want to target and for every day of the week from next week you can then select that workout uhm so you can go to the gym open your phone and get on with the gym session.  P: Yeah.  R: Yeah, and it saves you time long term instead of thinking about you know I need to focus on this today ugh I need to do that but instead you just jump on the app uhm so for example Tuesday night we are doing this so it has got that time saving management option uhm which I think is pretty good.  P: Uhm, most definitely.  R: In terms of the colour of the app, do you think the colour could be a bit more interesting?  P: The thing with most apps on the iPhone are that the colour is never good is it uhm.  R: Yeah, that makes sense. Awesome, so you have that function. So, if you press uhm build strength option that you have done and then add the workout to the date you want such as the 9th of March and you can save it.  P: Yeah.  R: Oh, you can actually set a reminder for each of your workouts which is a really good option to have I think.  P: I agree, that calendar function is really good.  R: I’m assuming that on a certain day and a certain time they will notify you through the app to remind you to undertake physical activity and therefore it sort of encourages you to maintain a healthy routine.  P: Yeah, I can see that.  R: Uhm, so in terms of a social element and building confidence and stuff like that uhm and motivation uhm do you think that little nudge that the app provides is quite nice to have?  P: Yeah, it’s gentle yeah.  R: Yeah, it’s not too aggressive it’s very much like don’t forget your workout and I think the timing and the wording of the notification is quite important.  P: Yeah.  R: Perfect! So, you have done that option so if you press uhm go back uhm and go back again and then down the bottom of the app we have socials uhm so let’s see what the social section is like uhm so for example I am following someone called Liv.  P: Yeah.  R: So, for example if you can tell me what you think about when you go through the option uhm so if you press on her profile and see what comes up.  P: Yeah, it’s a fitness focused app uhm it’s not a dating site uhm so you don’t need to know the in and out’s of a person’s background if that makes sense.  R: Yeah.  P: Uhm, so that is fine and the achievement option that they have put there is nice for motivation.  R: So, in terms of achievements what sort of things would you want from the achievements section uhm for example if you used the Accessercise app to go to the gym uhm what sort of achievements would you like to see?  P: That’s made me think haha.  R: Haha, so on the iWatch you have random notifications like take a breath and stand up after twenty minutes and you are rewarded for completing those tasks uhm but sometimes the achievements can become quite boring and it loses it’s purposes over time so is there anything in particular that you would like to see such as leader boards.  P: Uhm, I think if we went back to the point that I made about levels uhm it would be good to see that you have gone through the different levels of exercises that you have completed uhm for example such as light or easy or you know moderate uhm you have completed those levels for an exercise and you know I think it’s sort of nice to see the people in the videos uhm they are actually really humans and they are actual copies of them doing the exercises.  R: Yeah, having people that fit that impairment doing the exercise is really important. So, in terms of that uhm achievement section would you like a leader board but if you wanted a leader board would you like a leader board with other users to have that competitive side or do you think that is too much do you think?  P: I think that may be a bit too much because you don’t want it to become a negative uhm because your impairment does not allow you to get to that level uhm or ever get to that level due to the type of spinal cord injury that you have because you know it has certain limitations compared to you know C1/C2 to a T8 uhm the bodily function is completely different uhm so it may be a bit demoralising uhm you know what ever you want to achieve however you are never going to get there and that is kind of like a negative.  R: Yeah, so in terms of the leader boards obviously is there anything else that you would like to see in the achievements like daily records or daily achievements or monthly?  P: Yeah, I think the idea of having a streak on how many days in a row you have exercised is helpful.  R: Oh, so like a winning streak?  P: Yeah, like a winning streak.  R: What I like about the streaks is because if you do for example a month straight uhm you don’t want to lose that streak.  P: No.  R: It’s because you feel guilty of losing the streak so that could help build some intrinsic motivation which is pretty good.  P: Yeah.  R: Uhm, I think that could be a helpful option having the streak.  P: Yeah, but I think the actual profile side yeah is not a dating app uhm you know you can see other people uhm does it have the function to ugh contact for advice uhm so you can actually contact that person.  R: At the moment, you can comment and like and follow them uhm and unfollow them and stuff like that uhm I think the only people you can contact is the gyms if they provide that option such as a mobile number or an email address.  P: Ok.  R: So, if you go to the accessibility option you can find a local gym uhm you can press on it and it comes up with the time that they are open.  P: Yeah.  R: It also has a link to their number uhm I’m not sure because of privacy issues I think you can not do too much on the app regarding other users because as you said it’s not like a dating app.  P: Yep.  R: But you can comment on their workouts uhm you can see their profile.  P: Yeah.  R: So, what’s good with this is that you have finished your workout uhm you have the option to post it onto your profile.  P: Ok.  R: Or you can just leave it uhm so if you have a few friends that you follow on there you may want to post it and say hey I did this today and your followers can react with an emoji and a love heart and so on.  P: Yeah.  R: And you sort of feel connected to that group.  P: Yep.  R: So, it has that option uhm but if you go onto Liv’s profile uhm do you like the fact the information it has got your gender your uhm age your city uhm do you think that is enough or it needs more?  P: Yeah, that’s all you need.  R: But, what I think is quite good here is that you can see her achievements as well so yeah that function is still to come and to be developed but I’m assuming it will be like Liv has done two weeks of muscle strength training she has done this and that so you can always see what other people are up to and you can always see who she follows and who followers her.  P: I suppose what you possibly could have there is if she has had certain things uhm whether it’s getting toned, uhm build muscle.  R: Yeah, yeah it has that function already.  P: Ok.  R: Yeah, so I think she has just decided not to have that on her profile.  P: Oh, ok.  R: But you can have the option to say whether you prefer working out at the gym, park or at home.  P: Ok.  R: Uhm, even your goals is to build strength and so on uhm so it’s very selective on what you want to appear on your profile.  P: Uhm, right ok.  R: So, it does have that option uhm so if you press the three dots at the top right let’s see what comes up from that uhm so it comes up with uhm block user so it has got those options there.  P: Yeah, like safeguarding issues.  R: Yeah, it’s got the safeguarding it’s got the privacy uhm so that’s pretty good. So, if we go back to the main page and see what else they have uhm so if you press more uhm and let’s see what comes up uhm so you have a shop option uhm which I think is still yet to come but if you could choose what specific things would you like to have in the shop function?  P: I suppose it would be.  R: I guess that links in with equipment uhm so if you want to purchase equipment.  P: Yeah, yeah if you want to get a discounted water bottle, uhm cheaper clothing.  R: Would you like to see Accessercise have their own branded merch in the shop function or would you like just general sporting equipment.  P: Uhm, I think you know the actual brand is good.  R: Ok, yeah that would be quite useful.  P: Yeah.  R: What they do at Loughborough which is pretty good for example students can volunteer and do coaching and all that sort of stuff uhm and if they do one hundred hours of coaching uhm they might get a free water bottle uhm if they do two hundred they may get a gift card.  P: Yeah.  R: So, they could have that incentive on here where you have done a month’s streak of exercise uhm you then get a discount where you get a free water bottle from the Accessercise team delivered to your address you know uhm they can contact you by using the email you have used when signing up to the app and they can then deliver you a water bottle or something.  P: Yeah, uhm it’s getting you very incorporated into the app and its benefits.  R: Yeah, that makes sense.  P: Uhm, the nutrition option would be of good help as well ugh it would also be nice to have again uhm real life stories on the blogs and podcasts uhm of actual people who share a story about their injury and their physical activity goals and how they maintain their motivation uhm what got them into doing this uhm any top tips uhm type of thing so again podcasts you know people can talk about their experiences of disability sport and providing user their stories.  R: Yeah, that’s pretty good yeah. Uhm, in terms of vlogs would you like to see any vlogs available so users doing videos of their exercises.  P: Yeah, again you know if you have experts and things uhm who you know can share best practice or different ways of doing things I think is always good uhm seeing that visual video is quite important I think because not everyone has done PT and used instructors and things like that you know uhm your interpretation of the instructions but actually seeing a video of it maybe have a greater impact because you can actually see how low they are going uhm how wide they are moving their arms uhm how high and yeah I think that would be great.  R: What I have also noticed about the app is that it has a notification bell on the home page I think if you press for example ugh let’s say exercises uhm it has the bell in the top right corner.  P: Yeah.  R: So, it notifies you uhm when you have done a work out uhm I’m assuming that when you follow people it will provide you notifications when a friend follows you for example if you follow someone and they have done a workout then it might notify you saying this person has done this and you can look at it and provide a comment and it has that social element.  P: Yeah.  R: Uhm, but in terms of vlogs I think what would be good is if the app developers can provide monthly vlogs or what has been included on the app so for example if they have included another twenty exercises or whether there is a new impairment included uhm because I know this year they are including a range of different impairments such as Parkinson’s and so on but I think at the moment they only have a few so it would be good to get those monthly updates through a newsletter or even a notification uhm something like that would be pretty good.  P: Oh, definitely!  R: If we were to go back uhm the final little bits uhm for example if you go to more so you have got the nutrition, blogs, podcasts uhm these are all yet to come but if you press account settings and as you can see the impairment filters uhm so if you press on that for example you can see what they have at the moment.  P: Yep.  R: So, for example spinal cord you know uhm paraplegia and so on it has got those options.  P: Yeah.  R: So, the app very much filters the exercises and the options based around your impairment so it has got all that at the moment and obviously over time more will be included then you have got exercise habits so you can select uhm how often you exercise uhm it has the option for someone to assist you but obviously that is still to be developed so it has got those options uhm what do you think about these options?  P: Yeah, uhm so having this page slightly darker really stands out more and is helpful for me compared to some of the other pages.  R: So, you think the colour is really important uhm that is stands out and looks appealing?  P: Yeah, and it’s not like it’s an aggressive kind of like red uhm that stands out but instead it’s more a light colour.  R: And then you have got measurement units which is metric and imperial so if you’re American or British so what the app developers really want to do is to get the app very much pushed out onto an international level.  P: Ok.  R: So, they want to get out of the UK uhm there is an option to manage subscriptions so uhm they have the premium subscription so I’ll make sure you’re all set up by the end of the day but if you wanted to downgrade to a free version then you have those options.  P: Ok.  R: Uhm, change password they have that option uhm you have privacy settings uhm so for example if they sent you emails you can tick the box that you are happy for them to contact you uhm for example I guess if you put your phone number into the option there you will then get a notifications uhm such as texts saying you know your workout is due this time and so on but I’m not too sure.  P: Yeah, you have control over your account.  R: Exactly! So, the last thing we have to go through is the accessibility section which shows you the map of accessible gyms uhm so if you zoom out of London for example or Northampton uhm so all the orange options that you see are basically gyms that are accessible so there are three there and if you really zoom in the options will then spread uhm so for example if you were to press on one.  P: On the number?  R: Yeah, or just the white option here uhm so it comes up with this uhm so it has a rating scale so obviously no one has rated that one yet.  P: Yep.  R: It has got the times that the gym is open uhm it has got uhm the location uhm some places will provide their phone number and some places will provide just their email so the gym we are looking at now prefer just an email so they have the option for users to email them directly uhm so it has the little mail box that you can press and email them as well uhm but is there anything in particular that you would like to be added into that section there or do you think just the opening days and hours is enough?  P: I suppose what would be good is if there was a q and a option for example there is a particular question that you wanted to ask uhm the gym.  R: The gym, ok. So, this gym has a phone number that you can phone them but in terms of like the gym itself and obviously we don’t know what Darlington fitness is like so uhm having a three hundred and sixty rotation of the gym itself would be good but in terms of anything else that they could include so you mentioned about a checklist?  P: Uhm, no I think you know having the three sixty rotation option so you can actually see is important and it actually gives you the chance to uhm ask any questions.  R: So, as you can see it has the option to search for a local gym so for example if you were in London uhm just a heads up that some of the gyms have ratings but some of them don’t.  P: Yeah.  R: Yeah, so this comes up with yeah a list of the gyms uhm but do you think a list is ok or do you think that there’s too much information on one page but I guess if you put in a postcode it might pop up instead.  P: Yeah, most likely. For example, if you put London in it will even come up with London Road so yeah that’s fine.  R: Perfect, ok.  P: The gym that I work at is on here so happy days haha.  R: So, it’s on there is it?  P: Yeah, uhm so I run two libraries for the council uhm so yeah it will good to include more on there.  R: But I think the three sixty rotation option of the gym itself is a pretty good option and how you can provide feedback and comment and also the option to ask questions which may seem to be effective long term.  P: Yeah.  R: You know you can ask them questions they can get back to you uhm like a live chat thing but I’m assuming that not every gym will have that live chat function but sort of like a q and a would be good where someone posts something before and they provide the answers and you can look through that uhm I think that would be quite good uhm to have but obviously at the moment they have not got that function.  P: No, but yeah, I’m looking forward to ugh using the app.  R: Perfect, so we will go onto uhm the last three questions so uhm the first one is what are the potential positives if there are any of using Accessercise for improving physical activity levels?  P: I think doing it correctly uhm and choosing the right exercises to reach your goal uhm and what you want to achieve uhm and I think if you are looking to use gyms and things using the app to see what gyms are accessible instead of time wasting uhm is a definite uhm positive but you know finding out more exciting things that will be happening in the future uhm in terms of the nutrition side uhm an additional support side from the vlogs and blogs uhm will be great uhm on there but I think the more streaks that you can do would be a good incentive from the app uhm like you said with the water bottles uhm but overall I’m very impressed with ugh obviously everything included so far.  R: Perfect, ok. Would you say there are any potential negatives if there are any of using the Accessercise app for improving physical activity levels?  P: No.  R: No, haha.  P: There is nothing kind of uhm negative at all uhm it’s all positive.  R: One thing I can think about could possibly be the cost of membership uhm for example it’s something like forty pound a year uhm do you think that could be a negative for some people?  P: No, not really if you spread the cost out across the year uhm you know some of these apps for instance I don’t know are like one nighty nine a month or something uhm it can be done through the app account and stuff like that.  R: So, it’s basically paid for without even realising really.  P: Yeah.  R: Ok, perfect. Uhm, last question for this one is how do you think the Accessercise app could be improved for it’s use in improving physical activity levels uhm so if you have to think about four overall uhm areas of improvement what would those four be.  P: Uhm, sorry I couldn’t hear what you were saying.  R: Haha, no worries. So, how do you think the Accessercise app could be improved if anything for it’s use in improving physical activity levels for example if you had to name three or four key areas which uhm like right this is what I have seen today this is what the app developers need to go away and work on.  P: I know this may sound a little bit cheesy but if you actually hit certain goals or milestones uhm you could have a visual thing uhm like illustrations or stars or something uhm sort of like when you use your apple watch and you have dome your rings for the day it lets you know you have actually achieved it because if you are training alone it’s quite nice to receive that praise and that little pat on the back sort of thing.  R: Perfect! Again, is there anything else that you think.  P: Uhm, no but I think the colours on the app they could definitely work on and it’s something that should be improved uhm so like the colours stand out and it’s attractive uhm but no I think you know there are a lot of positives and there is nothing really until you start using it more and more uhm to build your workouts and actually using the program uhm I think there will be more feedback later.  R: Awesome, ok thank you. Uhm, and final question is uhm is there anything else you would like to add or discuss regarding the app or anything else that we have done today.  P: Nope, I’m looking forward to using the app in the future and setting my goals.  R: Perfect, thanks Gary. Thank you for participating in the interview.  P: You’re welcome.  R: Cheers. |
| --- |

## Transcript of interview undertaken with Participant 7

**Key code:**

R: Researcher

P7: Participant

R: Awesome, Fin! So, thank you so much for participating in this study uhm for taking part in the first phase of the trial and also the second phase today uhm so we are very much looking at the usability of Accessercise which is a new smartphone app focused on improving physical activity for people living with disabilities uhm to start of the think aloud interview I will go through ugh an interview overview so I will explain who I am uhm so my name is James Haley and my role within this research project is that I’m the lead researcher so I’m very much interested on how much physical activity people regularly do ugh and how we can actually benefit them and improve physical activity through smartphone devices and smartphone apps uhm the aims, importance and purpose of undertaking this research projects and the benefits of participating is so the aims are very much we want to see whether using this smartphone ugh application can improve various outcomes so things like heart rate, blood pressure, physical activity, pain, fatigue and so on uhm we are looking at extending this trial into a bigger one where we can look at the impact of the app on things like sleep uhm but also things like behavioural physical activity uhm where you are using fitbit watches and so on uhm the importance of it is to see you know how many people adhere to the app uhm what we can do to improve the app uhm you know the benefits of the app so far and work with the developers to try and push the app further forward uhm the benefits of participating are that you will be able to access the app you will be able to see what is available but also contribute to developing the app further so that more people within the UK and also on an international platform can use the app and benefit from the app uhm so the overall purpose is to try and reduce the barriers to physical activity so things like transport uhm you know like negative attitudes, stigma and get as many people as physically active as possible within the disability community.

P: Ok.

R: So, the university that I attend is Loughborough uhm my position is that I’m a Doctoral Researcher uhm the faculty that I’m involved in is in the Peter Harrison Centre for Disability Sport within the School of Sport, Exercise and Health Sciences uhm and the other individuals that are working on the project is Doctors David Maidment and Daniel Rhind as well as Sam Breary and Ali Jawad which is the directors and co-directors of Accessercise uhm I will briefly go through the ethical considerations uhm so for example the consent form and information sheet uhm so you have read the information sheet uhm you have signed the consent form to show that you are happy with everything which is perfect uhm confidentiality and anonymity so any information that you mention today will be confidential so these will be covered through things like pseudonyms uhm any data that you provide will be deleted at the end of the study and you also have the right in this project to withdraw if you feel like you need to uhm before we start I just want to let you know that there are no right or wrong answers uhm do you have any questions that you would like to ask before we continue with the interview process.

P: I think I’m ok I don’t have any questions at the moment.

R: So, very much with this interview it is very much what you think of the app uhm so don’t hesitate to you know speak about what you first think of the app when you use it uhm so we will now go through the introductory questions uhm so the first question I have got is uhm please may you provide the following information so your name, age, gender identity, ethnicity and county of residence.

P: Oh gosh! Ugh, Finley Jakes, twenty-three, ugh male, ugh.

R: Ethnicity?

P: White British.

R: And your county of residence?

P: Ugh, Leicestershire.

R: Perfect! So, to start off the interview could you tell me how you first got involved in the Accessercise application and why you wanted to participate?

P: Uhm, so I’m a disabled ugh elite athlete myself so I know what it is like to try and get involved in physical activity uhm and I have worked with James previously with the Para Sport committee at Loughborough and he contacted me to get me involved.

R: Perfect, and based on your experiences is there any reason why you particularly wanted to get involved in a study like this uhm with a smartphone app and physical activity?

P: Uhm, I just think that physical activity for disabled people is definitely important and there is still a stigma around not being able to do certain types of exercises uhm but having benefited from doing training programs previously I know how much they will help so having something like an app which is really accessible like on a phone rather than having to find the S and C ugh instructor will definitely help to do stuff at home rather than having to go to a gym.

R: Perfect, ok and in terms of using smartphone apps for physical activity uhm have you got any experience of using them in the past whether that is at the gym or at home?

P: Yeah, I have got a smartphone app from my S and C program currently uhm it has got similar sort of functions uhm for example sets, reps, weights uhm and a long list of exercises but this is prescribed to me by a strength and conditioning coach.

R: Perfect, ok! So, your experiences of other colleagues and other ugh friends that you are close with uhm do you know if they have ever been involved in any smartphone apps for people with disabilities.

P: Uhm, no so the app I’m using is not disability focused uhm so from as far as I’m aware no.

R: Perfect, and in terms of accessing physical activity apps for people with disabilities do you think it is something that has been limited in the past uhm do you think it is something that hasn’t really been around or thought about?

P: Uhm, yeah it probably has not been focused on much uhm I don’t think anything exists that has been tailored specifically for ugh disabled individuals uhm.

R: Perfect, ok so this question is a bit of a trick question but could you please tell me your experience of using the Accessercise app and how long you have been a member?

P: Uhm, so I have just looked over the app over the last few days so I have put together some workouts and looked through the social side uhm yeah just explored the app.

R: Awesome, ok so have you found it quite easy to find the app uhm and download the app on the ugh Apple Store or Google Play Store?

P: Yeah! Yeah, there is no ugh yeah it was easy enough to find with the name yeah.

R: I think one of the issues when it comes to physical activity for people with disabilities is the lack of advertisement for it uhm so you know they say there is a lack of physical activity opportunities uhm and whether that is a result of the limited advertisement so the fact that you have managed to find that quite easy is a positive thing to start off with is really good.

P: Yeah, I agree with what you’re saying.

R: Uhm, so we will now go through the Accessercise specific questions so do you currently use Accessercise in your role and if so when and how or if so when do you think you are more likely to use it?

P: Uhm, I have currently not used it uhm in my role so I will just explore it more over the next couple days.

R: Perfect, ok and if you were to use it ugh how often do you think you will use it uhm do you think it is something that you will pick up every now and then?

P: Ugh, yeah so I have a strength and conditioning ugh session twice a week so with the exercises are on here will definitely be useful to have stuff added to it so I can schedule in what I am doing each week uhm and it will have the videos and explaining how to do the exercises uhm because a lot of the time you can forget how to do an exercise so the videos are really helpful I think for me especially uhm and I think it’s really important having the videos and descriptions of the exercise on the app.

R: Yeah, that makes sense uhm has anything in the past ever put you off using an app like this uhm or using physical activity apps based on your previous experiences uhm do you find they are quite difficult to use or they are quite tedious to use?

P: Uhm, I have found physical activity apps quite difficult to use sometimes uhm when they have put exercises up and there are not videos linked to each of them on how to do something uhm so for example if there is an exercise you can’t do then it becomes a problem.

R: Ok.

P: Uhm but what is good about the Accessercise app is that there are actual videos for each exercise.

R: So, you like the video function?

P: Yeah, I like the video function uhm yeah so you can actually see how movements are done which makes exercising easier because you can copy the techniques especially if you’re new to exercising.

R: Ok, and I guess you have to go to YouTube to try and work it out which makes it quite difficult.

P: Yeah.

R: Ok, so obviously you have gone through the app uhm you have had a little bit of a look around but what were your first thoughts when you first started using the Accessercise application uhm did anything stand out that you really liked uhm or maybe didn’t like?

P: Uhm, it’s quite easy to navigate uhm mainly with the four tabs down the bottom of the screen uhm and I found that it is quite nice to make your own workouts uhm and it’s pretty simple to be able to do that uhm well currently right now with my exercises.

R: Ok.

P: I have just got seven available based on I think the filter section uhm well I didn’t actually put a filter in to start with but there is only seven.

R: That have popped up?

P: That have popped up otherwise I would have to search for the exercises uhm so like you have said there is two hundred odd exercises uhm I can’t necessarily workout how I would find all those other than the ones that seemed to have been put on so when you said there are two hundred odd exercises I found that odd when I couldn’t find it.

R: I think the reason why you couldn’t find them because you haven’t got the full access yet.

P: Right.

R: So, with the full access you will be able to access them all but if you have any difficulties accessing any exercises it’s probably an issue with the filter option and working out exactly what you want to work out and those exercises will then filter based on what you want to work out uhm so you like the function where there is all the different components at the bottom.

P: Yes.

R: Is there anything that you would like to be pinned down there or maybe you think would benefit?

P: Yeah, so what I was going to say is uhm I follow this user here called Clare and the groups uhm I did the lower body amputee group.

R: Ok.

P: But there are other groups like just starting out uhm and things like that uhm and now there is no where to find where to do that so I had to click on profile uhm go all the way down to the followers uhm search and then add.

R: Right, ok.

P: I think there could be an easier place to add people uhm so if you have got the followers there could be an add button.

R: Right, ok.

P: So, instead of going into profile.

R: So, to make it a bit more easier to navigate through?

P: Yeah, I think the app could be made easier to navigate.

R: So, you think this app could benefit from like a navigation option?

P: Yeah, because now there is just Ali on there so if I wanted to follow somebody else I now cannot find anything other than going through a few different steps.

R: Yeah, which takes a bit of time!

P: Even if I had to type in it can be slow uhm but I presume that when there are more people on the app you will be able to find people uhm like the search thing on Instagram uhm where there are loads of accounts that you don’t follow but you can find them uhm because right now I would not be able to find anyone if I didn’t know there account name specifically.

R: Right, ok.

P: Does that make sense?

R: Yeah.

P: If I don’t know there name or know who I’m trying to find.

R: So, you sort of thing the Accessercise app could benefit from like a friend suggestion option?

P: Yeah, something like that. A find friend or user suggestions would be amazing.

R: Oh, ok so a friend suggestion option could be pretty good. So, that is definitely something that they could work on.

P: And obviously with the groups there is the list of groups uhm but to get back to the group option uhm I have to go back down there uhm so here is the lower body amputees group uhm which I have joined but then ugh I have to go back onto the profile and then view all.

R: Right, ok.

P: Then add uhm so let’s say I have lower body amputee group here and then if wanted to try serious about training.

R: Right, ok.

P: So, instead of it being maybe slightly easier on the group tab uhm I have to go profile, groups, view all and then add.

R: Ok, so yeah that takes a bit more time when there could be an easier method included.

P: I think that is something that could possibly be added uhm but I think yeah the explore finding the gym option is a really good function on the app as well.

R: So, you like the accessibility option where you find different gyms?

P: Yes, I really like that! The finding gym option is great it’s a great feature. So, just to clarify the Accessercise logo is that an accessible gym is it?

R: Yeah, so someone has reviewed that and given it feedback.

P: Right, ok. I like the star review system here on the map function.

R: Uhm, where some if you go on there you won’t be able to find a review yet.

P: So, is it someone on the app has reviewed it?

R: Yeah, so yeah it will be a user on the app.

P: Ok.

R: It won’t be able to tell you ugh the quantity of how many people have actually reviewed that.

P: A clear weakness of accessercise is that you can’t see users reviews on the map.

R: But that gym will be rated out of five stars uhm so at the moment it hasn’t been clarified on what they mean by accessibility because accessibility means different things for different people depending on their disability uhm so what people have mentioned in the past is whether the app developers could develop something where there could be a rating for each component of accessibility so whether that is the building uhm the equipment uhm the staff and so whether that could be a function that could be added because when I see five stars for accessibility uhm I’m think well what exactly do you mean by accessibility so that is something that could be developed but just going back to something that you said about the groups uhm is there anything that you liked about the group uhm anything that stood out that you would like to be added to that function?

P: Uhm, well I think it is good to just find loads of different ugh exercises so there’s David Hill that I know and yeah the videos are great so yeah I think it’s good on this app that it’s very much impairment specific.

R: So, you like the impairment specific function?

P: Yeah, impairment specific will help users uhm because it is very specific to what you will need rather than scrolling down through loads of visual impairments, physical disabilities uhm exercises that are not relatable.

R: Do you think that is also a limitation for other apps that they have not thought about yet uhm and how this app has gone beyond that and focused specifically on the impairment which is good because it narrows down what you want to focus on?

P: Yeah, possibly uhm I’m not aware I have not gone through any apps where there is an amputee specific exercise.

R: Perfect, ok awesome! Uhm, would you say you have the required equipment to use Accessercise in it’s full capacity for example access to a mobile phone to use the application?

P: Ugh, yeah myself uhm I have a relatively older phone and the app still works relatively well on it uhm yeah there is a couple of buffering moments that I have had looking at it over the last couple of days but just reloading the phone it comes back.

R: And do you have access to a tablet if the phone had any issues?

P: Ugh, yes I do! If it’s the same sort of thing on a tablet uhm yeah a tablet would definitely help especially with videos if you’re in the gym uhm.

R: Ok, and in terms of obviously using your phone and using the app uhm is this something you are more likely to use at the gym or at home?

P: Uhm, the app I will probably use at the gym more than I would use at home but I would definitely use it at home as well uhm especially for exercises that don’t require the dumbbells and all of the equipment ugh and again there are some exercises on there that you can tailor to not have that.

R: Yeah, I think what is quite nice is that a lot of people think you know to be physically active you have to go to the gym but the fact that they have managed to include adapted options on there for equipment and also doing it at home uhm because that will save a lot of people time uhm if you think about the equipment you need to buy to go to the gym uhm the membership is expensive so if you could use things like a water bottle uhm or a towel at home uhm and do some flexibility stuff on a matt uhm it’s easy to do you know which I think is quite important.

P: Yeah.

R: Yeah, it’s really good. Uhm, so based on using the app a bit you know seeing the different functions uhm do you think there is any improvements that you believe could help improve the Accessercise application uhm so you mentioned about the videos that you like uhm and the key benefits but from your experience of what you would like for an app is there anything specifically you would want to be included?

P: Uhm, I don’t think so because I have not been able to go through uhm but when you record uhm can you record workouts?

R: Yeah, so you can ugh put how many reps and sets you have done uhm it’s also got timers as well.

P: Yeah, so when you do uhm for example another workout in a couple weeks are you able to uhm because when I have used an app before there is a little button on the workout that you’re on you can see what you have done previously.

R: Yeah.

P: So, you can see what you have done in the previous weeks.

R: So, if you add it to the calander.

P: Yeah.

R: You can go back and see the workouts you have done and all of the results for that.

P: Ok.

R: Uhm, I think the current uhm standard of the app there is not too many functions available but with the premium you have got a lot more.

P: Ok, so with apps I always find it good to track progress.

R: Ok.

P: So, you can see what you have done in week one compared to week ten uhm and it’s easier to look at the build that you have done for it.

R: It has got awards and incentive and things like that on here.

P: Ok, if accessercise could provide you awards or discounts that would be good.

R: It has got where you can track uhm but specifically in terms of tracking performance would you like notifications, so you are notified uhm you know you have achieved this uhm sort of like the iWatch does?

P: Yeah, yeah that would be good yeah there’s a couple of uhm strength and conditioning specific awards and badges that I normally receive for doing cycling and stuff and like that yeah.

R: Perfect and in terms of things that can motivate you uhm you said about uhm you know the social media section in terms of getting notifications and stuff like that is that something that would work for you or something that you would like to see more of?

P: Yeah, I think uhm I think with the followers you could track uhm so stuff on other apps that I know there is certain points for doing a certain type of exercise uhm on New Year Day you may get more points so you can see there is like a leader board and you can see each other’s sort of levels of badges and awards.

R: Ok.

P: Uhm, and stuff like that uhm could be helpful.

R: Do you think having competition would maybe be something that would benefit you or maybe motivate you uhm because I know some people that might not appreciate that.

P: Uhm, not necessarily me specifically uhm because I don’t ugh do all the badges uhm I see them on there but I’m pretty low down the leader board because I don’t do many of the badges and stuff but uhm it may help some people but maybe if there’s an option to not have it uhm well you don’t even need to look at it uhm yeah.

R: So, when you say stuff like that do you mean like being notified that you have done five workouts this week so sort of like a streak?

P: I think the idea of streaks could help users engage in exercise.

R: Ok, but from actually using the app now and the colours uhm and the visual appearances uhm does anything stand out that you like uhm are the colours ok there?

P: Uhm, I think the colours uhm I like the colours because it is sort of minimalistic uhm sort or less the style that is easy to follow it’s not lots of bright colours uhm which could give you a headache uhm it’s sort of nice and easy design.

R: Perfect, ok! Is there anything else that you think could be developed further so uhm we have got the videos which are good uhm it’s got the descriptions uhm it’s got the favourite section uhm calendar.

P: Uhm, other than the thing I said about trying to find other people in the feeds and stuff uhm yeah it’s not too bad and I’m happy with it.

R: Perfect, ok uhm so you have mentioned about your experiences of using other apps uhm do you use any other similar applications outside of Accessercise and if so what is better or worse than Accessercise?

P: Ugh, ok am I allowed to name them or?

R: Yeah, sure.

P: So, I currently use training peaks and garmen connect ugh and my gym one is just training ugh the training one there uhm yeah they are both really tracking exercises and stuff where you can see charts and tracking progress from months and years previously uhm so you can track what you are doing week in and week out.

R: So, based on the apps that you have used is there anything that you think maybe could be added onto Accessercise that you may have not seen on the app already?

P: Uhm, so for the garmen one there is the challenges thing which are like the awards and badges uhm which I mentioned previously uhm if that one wants to load uhm but yeah you can check other peoples workouts that they have done uhm but I know there is a similar thing in the social ones uhm which is similar on the Accessercise social section.

R: Uhm, I think at the moment what the app you have showed me compared to the Accessercise app is that it hasn’t got all those functions that you have just showed me like the heart rate, steps uhm and all of that.

P: If accessercise could include blood pressure, weight options would be good.

R: I think that is definitely something that could be added because I think Accessercise has got everything that we need but it’s missing that uhm that list that could be added on down the bottom.

P: Yeah, so the training peaks app does have your analytics so it has got your heart rates uhm so it’s more for run, bike, swim sessions but it has got all the analytics for what your average heart rate was for the session uhm what your power was uhm on the bike and stuff like that.

R: Awesome, ok is there anything else from that app that you think could be added to this Accessercise app?

P: Uhm, so for that app a coach is able to put in exercises for me so uhm maybe if a coach is able to access your account uhm because if you’re not aware of what exercises to do on Accessercise uhm a coach or someone who is familiar with exercises uhm like a physio but then again I don’t think everyone has access to a coach or a physio uhm but if you do the coach or physio can then add in a workout.

R: Right.

P: So, when you go onto the exercises and you have got the add a new workout uhm maybe a coach could put in a list of workouts and then you could follow that.

R: Yeah, what I think would be good for that is whether like a physio uhm a caregiver, family or some sort of relative could add that information in but then again I think the issue with that is confidentiality and stuff like that but I think that sort of stuff is quite valuable.

P: Yeah, I agree.

R: So, you don’t think anything else from other apps could be implemented or?

P: Uhm, no I think no not that I can think of.

R: I think from this app compared to other apps uhm the colour on Accessercise is a bit more appealing compared to the other one.

P: The colours, text size and fonts on accessercise are visually appealing to me.

R: The other one is quite dark uhm so I think they have done quite well with the ugh the colours which I think is pretty good.

P: Yes, accessercise have good colours compared to other fitness apps I have seen.

R: Right, so we are now going to move onto the main think aloud protocol so I will just go through an overview for this uhm so we are now going to move onto the main part of the study and ask you to participate in a think aloud protocol while you use the Accessercise application uhm the instructions for a think aloud protocol are straight forward I would like you to say out loud what ever comes into your mind uhm there are no right or wrong answers uhm we will just run through a quick practice to get you used to the protocol.

P: Ok.

R: So, can I get you to say out loud everything that comes to your mind when you think about physical activity?

P: Right, ok uhm.

R: So, from what you have said so far do you like the app do you think it has got the good functions?

P: Yeah, I really like the accessercise app I think it will be really easy to follow when you are starting the session.

R: Ok, because I think a lot of people think that going to the gym is all about getting stronger you know.

P: I think with exercise for disabled people they generally lack the knowledge.

R: But actually on this app there is actually a range of different things uhm such as mobility, flexibility, toning uhm even things like strength training, weight loss uhm so it has got those options for that uhm so you can very much filter it down to what you want your goal to be so I think what is pretty good is uhm that you can follow other people uhm see what there goal is uhm and you can then connect if they are sharing the same goal uhm so if they have got an amputee and they are focusing on muscular strength uhm you may want to connect.

P: Yeah, colours, various goals, locations and impairment specific are all great.

R: Uhm, it hasn’t got any links to ugh like uhm Facebook or anything so you can’t follow people outside of the app so it removes that sort of privacy issue uhm where people are trying to follow you and stalk you all of those issues.

P: Yes, if you could message other users that would be a really good function.

R: So, it is very much focused on uhm this is the social section of the app uhm communication if you want to communication.

P: Yeah.

R: Uhm, so what I will get you to do is can you say everything that comes to your mind when you think about physical activity?

P: Oh, gosh uhm ok so yeah physical activity uhm is good for my motivation uhm so stuff with regards uhm my training because I’m training most of the time so being able to be physically active is good thing for me to uhm be able to go towards my goals and also uhm sort of being ugh a disabled uhm it’s quite a nice feeling being able to go for a run or go to the gym uhm the same gym as an able bodied athletes uhm yeah that go across uhm what else ugh the social side is good too uhm I really like that uhm so mixing between everyone so uhm as an amputee I do not see myself as strictly prohibited to an amputee group uhm I train with people in wheelchairs uhm people cerebral palsy uhm people that are visually impaired uhm so it’s good to get that breadth across everyone uhm and I find that is a really good thing to help breakdown barriers as well across the able bodied individuals.

R: Brilliant, ok! So, you mentioned about barriers so obviously there is a range of different barriers when it comes to physical activity for people with disabilities uhm do you think an app is something that could help fight against those barriers so in terms of things you know like the costs and the time of actually doing physical activity so an example of this is if you are in a wheelchair obviously you have to get up, washed, dressed which can take longer because you need a caregiver to support you with this uhm and then actually go to the venue to actually exercise uhm that could be a whole day thing.

P: Yeah.

R: Do you think the fact that you can do it online at home is a bit easier for people to get involved?

P: Yes, yeah, it’s definitely easier to get involved and yeah obviously easier for it not to be something locally as well it could go quite wider a field you could do sessions with people who are not near you uhm you could add workouts and do them together at home uhm yeah it would definitely be good for that.

R: I think obviously because you will be getting free access to the full version of this app uhm do you think people that first use the app will want full access uhm which I think is around forty pound per year and whether that price is reasonable?

P: Uhm, that’s a good question. I think accessercise is pretty for what it provides.

R: I think forty pound a year is ok.

P: Yeah, considering with what actual gyms yearly ugh membership prices are uhm let alone monthly uhm it will definitely be a cheaper way of working out what workouts you can do rather than hiring a strength and conditioning coach.

R: I think the problem with some people with a spinal cord injury is that they experience a lot of pain so doing exercises is not you know just all the benefits uhm it’s actually the pain of doing physical activity so the fact that it’s got a range of different goals on there could be really helpful uhm for example one of the functions is whether the exercises are light intensity, high intensity and so on.

P: Yes.

R: So, you can then filter that down depending on how hard you want the exercises.

P: Yeah.

R: Uhm, so I think that is a good function uhm you know whether doing physical activity through the app can actually alleviate additional health complications as a result of using it.

P: Yep, accessercise has all the right things to make the app great!

R: So, I think that is quite beneficial but if we were just to go onto the home page uhm so if you open up the app uhm we will go through it step by step and I just want to get your opinion on everything you see.

P: I would but the app has crashed uhm it doesn’t seem to be working right now but I will try and remember what was on there.

R: Ok, so obviously you have got your followers uhm so you have a few people on there.

P: Yeah, the option to uhm follow other users on here is helpful and supportive.

R: Uhm, so yeah you go on there uhm you see the following option uhm do you like the fact that you can follow people?

P: Yeah, no I think that is good uhm that is different from uhm what other training apps have got uhm it’s similar to others but uhm yeah it’s always good to ugh follow what other people are doing.

R: So, you think having the social section uhm a bit of competitiveness is quite good to have on an app?

P: Yeah, definitely! I really like the social section on accessercise uhm it’s useful.

R: Ok, in terms of groups is there anything in particular you think you would want or you would appreciate having included?

P: Well, yeah I appreciate having the ugh lower limb groups uhm so it’s easier to find something that is tailored to your impairment uhm and it’s obviously going to be good to see if you’re struggling with certain exercises it will be good to see what adapted options other people with similar impairments make so you can then make them yourselves.

R: Perfect! Ok, and in terms of uhm groups would you like other types of sport ugh clubs be added to that?

P: Yeah, other groups would be good uhm because currently it’s more based on the impairments and then what your goals are in terms of training uhm for the intensity or just getting used to it so having different sports to therefore focus on uhm so for example if you wanted to do swimming and having exercises that will help swimming specifically would definitely help and you can then have a long list of different groups.

R: I think that it’s good that it has the impairment filter section on there where you can find groups that are based on the impairment.

P: Yeah, the fact uhm accessercise is focused on disabilities is good.

R: Uhm, would you like to see things that are like local wheelchair rugby classes or similar activities?

P: Yeah, yeah so if you’re into your weightlifting having weight lifting specific exercises and groups or sport clubs would be good to include on the app.

R: Yeah, because I have had a few people mention that they really want to get involved with like wheelchair basketball but they can’t find the club uhm but whether that is something that could be added to ugh this uhm and taking it away from just a gym focused app.

P: Yeah, yeah.

R: Uhm, and just focus more generally on physical activity as a whole you know instead of gym focused would be really good.

P: Yeah, that would be good. So, somebody could advertise clubs that are available and you could add that to the explore bit as well.

R: Right, ok.

P: So, the Accessercise logo uhm you could have the Accessercise logo with different clubs.

R: I think at the moment it’s very much focused quite a lot on gyms and I know why they are doing it because they want to encourage some sort of exercise but to develop the app further they could add that in which would be great.

P: The exercises are great but having sport clubs would help users exercise more.

R: Uhm, but in terms of actually the groups uhm what information would you like to see included on there uhm would you want it to be like uhm once a week join this group for a class or something like that?

P: Ugh, maybe incorporating sport opportunities and events would be good.

R: If there were groups so for example if it was like amputee uhm what sort of specific groups would you want uhm would you want uhm a sort of like chat group where you just chat to people about your impairment?

P: Yeah, yeah you could ugh develop into another tab around having group chats uhm yeah that would definitely work so if somebody had a certain query which was specific to a certain impairment they could put that into ugh a group chat or something.

R: Perfect, ok so that’s all the group stuff done which I think is good uhm but in terms of following people do you like the fact that you can follow people uhm they can follow you uhm do you like how it’s sort of like social media in terms of Instagram where you have got a new follower?

P: Yeah, yeah that’s good but I think what would help that follower side of it is having the suggested friends option appear on the app rather than specifically typing in someone’s name uhm because if you don’t go to a gym and you’re just starting out and there’s not a long list of people that you could follow that is sort of tailored to you uhm it would be hard to find someone just based off.

R: So, you sort of want the app to suggest people that you may know in real life?

P: Yeah, the option of having suggested friends appear would be good.

R: Ok, that makes sense. So, if you just refresh the app and see if it works now.

P: Yeah, sorry. Uhm, I think I will need to turn my phone off.

R: Haha, no worries. Does it normally crash?

P: Ugh, no.

R: Yeah, it would be good to see how good this app is at you know improving various outcomes so the data you have provided me today is really helpful to see whether there were any changes or anything going on uhm because I think at the moment the app is only UK focused uhm so I think the app developers really want to try and get it out onto an international platform.

P: Yes.

R: So, with the accessibility option on the gym you can see all the various gyms within the UK.

P: Yeah.

R: But you can’t really see anything abroad. So, they are really trying to get more people involved so I think the actual uhm developers are going out to different places to see whether people would want to use the app.

P: Right, let’s try this app again.

R: But I think the things with an app uhm just general physical activity apps is that there are so many but there’s not many focused on disabilities.

P: Oh, here we go I’m back on the app.

R: That’s fine so in terms of uhm for example if you followed someone.

P: Yeah, so the only person I’m following at the moment is Ali.

R: But in terms of obviously the information you see on his profile uhm is there anything in particular that you would want to see on his profile that could help you so obviously you get things like age uhm male uhm you may get a profile photo.

P: Uhm, well the only thing I think that is up there right now is the workouts that they have put out so I don’t know uhm maybe if there was an option to put up uhm like the video of specific exercise or something uhm yeah because yeah I don’t know what else could be added that you could put up for a post.

R: Uhm, but I mean in terms of the profile.

P: Yeah.

R: So, for example Ali you may see a photo of him uhm you may see his age uhm his gender but is there anything else that you would want see on there in particular uhm so whether that’s their exercise goal they are working on?

P: Yeah, yeah, I think on the app in terms of workouts it had in a little colour what session that was uhm one was for increasing strength uhm another one was getting fit so I think there are little tabs on there for that.

R: Ok, perfect ok so if you were to press the profile section up here.

P: Yeah.

R: Ugh, so you have got your name.

P: Yeah.

R: Is the photo something you would include?

P: Ugh, yeah I think it’s good to include photos on my profile.

R: Perfect, ok and you have got the achievements and followers but what specifically within the achievements section would you want to be included uhm do you like that function when you see that?

P: Uhm, yeah so yeah from other apps the achievements are like it’s ugh a sort of growing tally so if you complete a certain amount of steps you’re provided with a reward uhm like twenty thousand steps a day uhm one hundred thousand steps a week uhm and so on uhm so stuff like doing a certain amount of sets in a week or a certain amount of reps.

R: So, you like the idea of having like a record of how much you have done per week?

P: Yeah, so the number of sets I have completed in general rather than a week.

R: Ok, so like a frequency table or something like that so you can monitor your performance?

P: I think accessercise is missing logistics data I want to review my progress.

R: Yeah, so like a percentage of how much you have completed overall. Ugh, they are all great recommendations which is good uhm but whether that keeps someone using the app long term is difficult uhm because we know long term behaviour change is quite challenging uhm but is there anything you think could be added uhm so I mentioned about a streak uhm where someone has used the app for like one hundred days and they don’t want to lose that streak so they are more likely to use it.

P: Yeah, streaks could always work they would definitely motivate me to exercise.

R: Do you think that is something the app developers could include?

P: Well, stuff like uhm you know when you have got the ugh what is it uhm one hundred minutes of physical activity per week.

R: Yeah.

P: Vigorous physical activity per week uhm if you have a total of that each week uhm and if you hit that for a week and then you have a streak of how many weeks you have hit across the year of physical activity, and you can see that you have been physically active every week all year so.

R: That’s a personal benefit so do you think maybe the developers could provide uhm rewards or gifts or like a discounted membership for users that use it regularly or a t-shirt?

P: Ugh, yeah, I think rewards like t-shirts and water bottles are good.

R: That would be pretty good. Uhm, and obviously you have got the following section where you can see who you follow uhm.

P: I don’t think it’s working right now.

R: But if you were to go back onto ugh this one.

P: Yeah.

R: If you scroll right down to the bottom uhm yeah and press this one you have got uhm training uhm personal information uhm but do you think anything else could be added to that uhm do you think it’s quite simplistic?

P: Uhm, I think maybe when you press profile uhm that could be part of the account settings as well.

R: Right, ok.

P: So, maybe this feature here could be move when you press on profile maybe.

R: Right, ok so having it all on one section would help?

P: Yeah.

R: Ok, do you think having like just your email address and maybe what you’re working on is enough?

P: You could maybe have other stuff like other socials uhm so if you really liked following someone and you wanted to find another one of their socials uhm maybe having the socials on there as well.

R: Ok, and in terms of maybe age and gender do you think that could be included?

P: Ugh, yeah it could be included. So, things like age and gender.

R: I think what would be really interesting is if you could follow somebody and press on their profile and what their workouts are.

P: Yeah, and maybe having a description on their profile so obviously knowing Ali is ugh a paralympic weightlifter uhm maybe having a little description on their profile of who they are uhm so based on the suggested friend recommendation that I mentioned uhm and if you’re really into upping your intensity maybe a suggested could show up some elite athletes and you could start to see what they’re doing.

R: Right, ok uhm I think you don’t want to press on someone’s profile and all you see is a little bit of information uhm you want to see some content uhm you know on Instagram when you follow someone and you see all their posts and so on.

P: Yeah.

R: Having something like that would be pretty good so we have done the personal section uhm we have done the ugh social uhm if you press exercises.

P: Yeah.

R: Do you like the fact that as soon as you press that uhm the list of exercises come up?

P: Yeah, it’s definitely very good and I think the filters are very good as well with the range of different options to choose from.

R: Do you like the fact that there are various types of goals there?

P: Yeah, yeah it will definitely help tailor because just having certain exercises like uhm this one here is going to be useless for trying to do some cardio stuff uhm yeah having the different tailored sort of sessions will be good.

R: Ok, and do you like the fact uhm so if you scroll to the top uhm the fact that there is different locations to exercise?

P: Yeah, yeah, I think the different locations will definitely help with regards to what equipment you can use.

R: In terms of the actual page itself uhm do you think the descriptors are quite easy to understand uhm you don’t think that it’s quite bulky?

P: Ugh, yeah, yeah it’s quite yeah simple as well uhm but like the mobility here for choosing a goal for me uhm is I think uhm is a bit more than just the frame because that indicates that goal is only for elderly individuals so I think maybe that logo could be slightly changed to something else uhm if that makes sense.

R: Yeah.

P: For example, if I wanted to do mobility and there’s the frame there I may think it’s for just the elderly population?

R: Yeah, I know what you’re trying to say. They need to focus on a better logo for that goal then.

P: Exactly. Some of the logos for each could be improved and made more clear.

R: In general, do you like the logos and what has been provided?

P: Yes, yes! In general, I think it is perfect and very creative.

R: So, you like the creative side. Uhm, I think if the logos weren’t there it will be quite bland and boring.

P: Yes. Some of the things on accessercise are quite bland and boring.

R: But do you like the fact you can select the different body component when designing a workout?

P: Yeah, yeah ugh yeah obviously to tailor what exactly you wanted to work on yeah lats and so on uhm yeah it’s good but also if you’re just starting out and you want them to be easier and then if you’re trying to build up you can then go onto intense I think.

R: I think if you first started using the app uhm you would think you know oh I can’t do too much I want to start with something easy uhm you have the option to do that uhm so that is pretty good.

P: Yeah.

R: Uhm, do you like the option uhm so for example if you had a spinal cord injury and you’re in a wheelchair and you wanted to go to the gym for the first time uhm it has the option to bring a carer?

P: Yeah, yeah that is definitely good as well to tick whether you need assistance.

R: Yeah, but what specifically would you want uhm for example if you had a spinal cord injury would you want from that function?

P: Uhm.

R: Would you want ugh it to be linked in with the gym uhm where someone from the gym comes over and supports you?

P: Well, yeah it would be good if you ugh could let the gym get a notification knowing they you require someone to support you in the gym uhm that would be really good.

R: I think the problem with that is that ugh there is an issue with coaches having limited knowledge of like ugh the benefits of physical activity but also how to support someone with a disability.

P: Well, that is definitely something that I have had where I have been with new strength and conditioning coaches but they are not entirely sure.

R: How to adapt it to your needs?

P: Yeah.

R: Uhm, so whether actual people in the gyms could be provided additional training on how to support an adapted athlete is a different story.

P: Yep.

R: Uhm, obviously you have got the option on the filters with the different equipment.

P: Yeah, with the prosthetic leg uhm specific exercises is also good.

R: Do you like the way that the option on the filter is laid out uhm with the different boxes uhm do you think they could do a better job?

P: Well, I think initially when I was going through here and I went down to the difficulty and saw all these tabs but then I didn’t open them.

R: Right, ok.

P: I just went here and thought that was it but now I have looked at that outdoor training and there’s a prosthetic leg tab.

R: So, you think the lay out of the actual task at the bottom could be laid out better?

P: I think these could be more expanded.

R: Expanded?

P: It would be good to visually see it better rather than just tapping on it.

R: Yeah, I think the only problem.

P: I think yeah because there’s such a long list uhm it’s probably why the developers have done it like that but it could be made much smaller.

R: Yeah, I think they are trying to avoid having too much information on the screen.

P: Yeah, but because this is equipment for outdoor training uhm home and gym uhm but then you have gym, outdoor there uhm maybe having those tabs like that so then you can open.

R: I think what would be pretty good if I was to use the app if you were to select gym uhm you select strength and then ones that are not related to those choices get blocked off.

P: Yeah, some of the equipment here is impossible for my goal so remove them.

R: So, for example the ones that do appear are important and light up or you know.

P: Yeah.

R: You can press on it uhm because you want to narrow information down uhm because so far at the moment there is quite a lot on there.

P: Yes.

R: And it’s easy to just press different buttons and get away with it.

P: Yeah.

R: So, I wonder if certain equipment could be adjusted or like uhm avoid you pressing on it based on those previous selections uhm but if you see for example how there are four here uhm four four two uhm and you scroll down again right to the bottom and just press on one uhm how you have got here you have got like two then three uhm then three and then two.

P: Yeah, the layout for the list of equipment needs developing as it’s messy.

R: Do you think there is anything they could do to maybe lay that out better uhm to make it a bit more appealing to the eyes?

P: It’s alright but it’s not all alphabetical if it was alphabetical that would be good.

R: Right, ok.

P: So, if you really wanted to find something uhm but I don’t mind having the options laid out like three then two then one and so on because obviously some words are longer than others.

R: Yeah.

P: I think that is fine but I think maybe if you’re trying to find something in particular uhm having alphabetical order it would be easier to find because just having weight disk uhm and having water bottle over there is confusing.

R: Yeah, so having alphabetical order would be really good uhm do you think the layout uhm in terms of the way the developers have done it uhm with the location on where you want to do the exercise uhm then the goal and then the equipment is good?

P: Yeah, so that is sort of what I was saying uhm these three because they relate to that option at the top uhm they could be removed uhm so the equipment for the different locations.

R: Right, ok.

P: That could then be moved to a part of the location.

R: Right, ok. And in terms of having an amputee uhm you have got here ugh the assistant and carer option here uhm is there anything that you would want to be added to this section uhm so based on your experience you know uhm.

P: Uhm, so I would say uhm no I think ugh no.

R: So, I think about things like a spinal cord injury being in a wheelchair ugh they have a lot of spasticity issues.

P: Yeah.

R: So, whether they can do things like flexibility uhm whether I don’t know there could be some alternatives or something uhm removed or added depending on the impairment uhm but I guess with an amputee it’s not too bad.

P: Uhm, I think for me depending on impairment starts going into things where you say you can’t do something uhm so I think having access to everything regardless of what impairment uhm you can then try what you want.

R: Yeah, that makes sense.

P: Uhm, I’m not sure if blocking off based on impairments is the right thing to do but if you want to do uhm mobility uhm blocking off some of the strength exercises uhm would be fine but yeah.

R: Yeah, that’s a good point. Yeah, it would be interesting to see how they develop that carer function uhm because at the moment you press it and nothing happens so whether they could link that into the actual uhm accessibility section on the next section is a different story uhm but I like how they have got the applied filter section in purple uhm which stands out but they have also got the option to clear all.

P: Yes.

R: So, if you were to press the wrong one you have got that and then you go from there so that’s pretty good so if you go off this bit.

P: Yep.

R: Uhm, and you were to press this option.

P: This one?

R: Yeah, that’s the one.

P: It’s not working?

R: Oh, maybe press the video?

P: No, it’s usually when I press it that it appers.

R: Right, ok.

P: This has only just happened.

R: Is there any other way you can go onto this?

P: Maybe it’s just a Wi-Fi problem.

R: Possibly haha.

P: Oh, we are back!

R: Right, so what are your first thoughts when you see this so the videos are at the top uhm you can see what type of exercise it is.

P: Yep. I love the videos the accessercise team have done a good job with that.

R: So, what’s your initial thoughts when you see that uhm do you like it?

P: Yeah, I think the videos are a really good function on this app and how you can see videos for al the different exercises uhm and also having the descriptions uhm I like the diagram of the muscle groups uhm as well.

R: I like how the uhm picture has someone with an amputee.

P: Yep, the photo here is some with an amputee which is nice.

R: So, it’s very much tailored to the individual.

P: Yeah, I like how this app focuses specifically on my disability.

R: It will be interesting to see how someone with a spinal cord injury will be whether there in a wheelchair or using ambulators such as walking sticks uhm but I think that is pretty cool.

P: Yeah, definitely!

R: Uhm, but with the videos is there anything else you would like to see included uhm so when you actually watch the videos.

P: Uhm, actually is there a voice over?

R: Uhm, yes there should be a voice over.

P: It doesn’t seem to be working.

R: Is it not working?

P: Possibly.

R: It may be because it’s muted?

P: No, it’s not that.

R: Maybe there isn’t a voice over I thought there was uhm maybe it depends on the exercises.

P: Ok, maybe if there’s ugh a voice over option as well uhm.

R: Do you like the fact that there’s actually audio descriptions on there?

P: Yes, yes that is obviously good. Audio descriptions for each video is great.

R: I think the fact that you have also got the key benefits uhm the descriptions is pretty cool.

P: Yeah, the key benefits and description for each exercise is good.

R: Do you like the way that it’s been laid out so far uhm so it has got the video at the top uhm then the description?

P: Uhm, I’m not sure having the show more option is always that useful uhm but instead maybe just having it all in one block.

R: Right, ok uhm I think the useful tip could be developed a bit uhm because at the moment it hasn’t really got anything beneficial to it.

P: Yeah, the useful tip box could include a bit more information.

R: So, it’s got here show more uhm or show less.

P: I just noticed that.

R: Do you like the factor that you can see the muscle components uhm and something that would help other users?

P: Yeah, that is really helpful I think that’s a good feature of the app choosing which muscle components to work especially if you’re a beginner in the physical activity world uhm but I think just usually when you’re on an app and you see that uhm and read the descriptions and then move on uhm but clicking the show more I think is just an extra step to get to the other stuff.

R: Right.

P: I think maybe having it all out like that would be a better option.

R: Do you think with the descriptions of how to do the exercises it could be bullet pointed uhm for example like step one uhm step two?

P: Yeah, that could make it cleaner uhm because it’s hard to see where it ends uhm between this line and that line uhm so bullet points for the descriptions of each exercise could really help uhm and definitely make it cleaner uhm because you have a bullet point up here but nothing here.

R: Yeah, and the little option here is the favourite.

P: Yeah, the favourite button is useful I will definitely be using that.

R: You can then add this exercise to your uhm favourite list and then you can add that to your workout.

P: Yeah, favouriting exercises and having a favourites list is always list.

R: So, it’s here which is pretty cool. Uhm, if you go back onto the exercise section uhm you can see here where it says like leg uhm.

P: Hard, leg, kettle bell.

R: So, that’s the exercises so if you were to press on it uhm you can see how difficult and hard it is and so on uhm so that’s that function uhm but do you think overall if you had to sum this section up uhm whether there is anything that could be improved?

P: Maybe just the formatting of everything needs to be improved quite a bit.

R: Do you think there is too much text included?

P: I don’t think that it’s too much text but I agree with the bullet point thing uhm tidy it up and then yeah uhm having it all on one page rather than maybe having the show more.

R: Perfect, ok and do you think anything else could be added to the page or do you think there is enough?

P: Uhm, let me think uhm whether anything else could be added ugh not that I can think of no uhm maybe an idea of a recommended amount of reps and sets of that exercise.

R: How many to recommend?

P: Yeah, recommended amount of reps and sets because if you’re new to it and you want to do kettle bell squats uhm you are then unsure on what to do and you have no clue uhm on how many reps and sets I should do uhm should I do three sets of ten.

R: Uhm, so like recommendations?

P: Should I do five sets of five.

R: Yeah, I agree that would be a good option to include uhm because you will know how to complete the exercise through the demonstrations and videos but you won’t know how much of it to complete uhm so providing you know the frequency, intensity and so on would be great.

P: Yeah, I think a section on recommended reps and sets per workout would help.

R: So, that’s good uhm so at the moment you cannot access the calendar function but do you like the idea of adding workouts to your calendar?

P: Yeah, the calendar is good to track uhm and go back to previous workouts uhm similar to other apps where you can track back to workouts that you have done.

R: Is this a function that you think you would use?

P: Yeah, the calendar function is great! I think especially planning ahead as well.

R: So, this notification option uhm so if you press that uhm you should be able to see the app updates uhm is there anything in particular that you would want to be notified on?

P: Well, yeah if there’s certain events like ugh the wheelchair basketball thing uhm that we were discussing just now uhm maybe if they’re looking for participants uhm they will be able to use the app?

R: So, you’re saying uhm opportunities to advertise other sport events on the app?

P: Yeah, opportunities that for participation uhm because if you’re looking to get into sport uhm it’s usually quite difficult to find if you are not already in a certain system.

R: That makes sense. Uhm, so you have got my workouts uhm my favourites uhm my exercise uhm my notifications uhm and calendar uhm we will now move onto the final section which is explore.

P: Yes. The favourites, notifications and designing your own workouts are great.

R: What were your initial thoughts when you first saw this?

P: I thought the explore section on here was really good uhm yeah uhm with finding all the gyms that are nearby uhm and are accessible uhm I didn’t initially see the accessercise logo but obviously already knowing which ones are accessible uhm as well and obviously zooming out and seeing how many there are.

R: Uhm, so your initial thoughts when you first saw it are that you like it?

P: Yeah, definitely! The explore function is so useful and I will be using it.

R: Ok, so if you were to zoom in quickly uhm and find an accessercise logo one uhm and you were to press on the actual logo itself uhm this is what comes up uhm so it’s powered by google uhm so it’s linked in with the maps.

P: Ok.

R: So, what are your initial thoughts when you see this uhm so you obviously see four stars?

P: Uhm, initially I’m not entirely sure what it means but I’m guessing someone on the app reviews the gym.

R: Yeah.

P: Is that right?

R: Yeah, so I think the purpose of the rating system is to make other users aware of how accessible the gym is so it’s to make it easier.

P: I think depending on who reviewed it uhm I’m not sure whether there is a way to put in a written review so uhm everybody knows what they mean by accessible uhm because they could mean there’s a lift uhm a lift available or it’s nice and spacious.

R: Ok.

P: But right now, I don’t know what that four star accessibility means.

R: Right, ok so you’re saying that an area of development is to have a written review?

P: Yeah, so maybe in a gap saying what was accessible about it uhm if that makes sense and sharing it with all users so they can see it instead of hiding it.

R: Do you think just having general reviews of that gym could be pretty good?

P: Yeah, yeah because it obviously gives you uhm an insight into whether the gyms are accessible uhm and if you’re selecting a gym around you and there’s three available uhm you may select the gym based on the reviews.

R: Ok, so if you were to press on the accessercise logo again.

P: Yes.

R: Do you think the information regarding the phone number and the opening hours is quite good?

P: Yeah, yeah definitely! The phone number and opening hours is good to have.

R: So, as you can see here it says uhm rate this gym uhm so you may rate it and someone else can see it.

P: Oh, I see yeah so that’s where they can review it and then leave a comment.

R: The only problem is uhm that you can leave a comment but no one actually sees the comment uhm so whether that comment could actually be added to that gym would be great.

P: That would be good if everyone can see the reviews written for each gym.

R: But I guess the only problem is that you don’t want to make the gym feel bad for not being accessible.

P: No.

R: You don’t want people to avoid using that gym and them losing business.

P: Yeah, but that might highlight the gaps.

R: The problems.

P: Yeah, the areas that need to be improved.

R: So, you have seen the gyms uhm you have seen the rating system uhm.

P: I think that only happens when someone has reviewed it right?

R: Yeah.

P: Ok, because I know Hollywell uhm that gym has a lift and it’s pretty accessible but right now it doesn’t have the logo.

R: Yeah, so these are obviously things that can be developed further in the future uhm so you have seen the opening hours uhm you have seen the review system uhm but do you think there is anything that could be added for that gym which could be easily viewed by the users?

P: Uhm, so maybe some images of the gym would help uhm so like a usual thing on google.

R: Ok.

P: So, like the usual thing on google where you press on something and you can see what it looks like uhm but I guess that is a personal preference.

R: So, do you sort of mean like a rotation of the gym?

P: Yeah, so just like ugh a couple of images uhm like some slides.

R: Ok, uhm is there anything else in terms of a link to the website or anything like that?

P: Uhm, maybe if there is like disability parking available that you can see through the app would be useful uhm so in terms of like the accessibility side of things.

R: Ok, so you want to have like a link that shows you whether the parking in this area is any good?

P: Yeah. An accessibility checklist for each gym would be amazing to include.

R: Ok, uhm.

P: I think that would be a pretty useful option to have on the app uhm to allow users to see whether the parking outside the gym is good or not.

R: Ok, so if you were to put your postcode in for example.

P: Yeah.

R: Are you from Eastbourne?

P: Yes.

R: It’s a nice area down there.

P: Haha, yeah.

R: Do you like the fact how the different gyms are listed?

P: I have put in Eastbourne but it has listed gyms within Loughborough.

R: Have you tried searching when the postcode is all together?

P: Uhm, let me think.

R: I think maybe because of your location settings and whether that has been ticked on.

P: Yes, you’re right.

R: But do you like for example the fact that it’s got a listed version with all the gyms rather than have them scattered across the map?

P: Yeah, yeah, a list of accessible gyms is useful to have.

R: Yeah, it’s easy to navigate through which is pretty good. Uhm, do you think in terms of what you have seen for this is there anything that could be developed for the map version?

P: Uhm, no not that I can think of.

R: No, so you are happy with that?

P: Yeah, the map function is arguably accessercises best feature.

R: Perfect, ok. Awesome, so we will move onto the final bit which is the more section.

P: Yep.

R: So, you can see the shop.

P: Yes.

R: But at the moment it’s under development uhm but is there anything in particular that you would like to have included on that so uhm any incentives or particular items to be added to that?

P: Uhm, maybe some stuff with some types of equipment uhm so if you don’t have a band uhm maybe stuff that will be helpful for the workouts.

R: Ok.

P: So, like an accessibility band.

R: Ok, so uhm whether the shop can actually link some equipment that you may need for your workouts to the shop?

P: Yeah, that would be extremely useful having links to equipment in the shop.

R: Ok, so not just water bottles uhm t-shirts or all that sort of stuff.

P: No, so something that would actually help the users yeah.

R: Ok, perfect! In terms of nutrition, is there anything in particular that you would like from a nutritional side?

P: Uhm, well yeah maybe some recipes uhm but also tips on what to have after specific sessions uhm so if you have had a strength session uhm maybe having tips on that uhm a more protein based meal or ugh if you have done a more cardio one uhm maybe having something that will help get energy levels back up ugh and stuff like that.

R: Ok, and in terms of meal plans uhm you wouldn’t find that quite interesting or anything?

P: Ugh.

R: I guess it depends on the person.

P: No, no I would find meal plans good but again it all depends on the person.

R: Yeah, it depends on the person. In terms of blogs uhm is there anything in particular that you would want to be added to that uhm or what are your thoughts about blogs?

P: Uhm, I have never followed a blog personally but obviously it’s something that people will look at and uhm will obviously uhm be good to follow someone’s journey around what training they are doing uhm be able to ugh yeah it would just be good to see what people are doing in a week and a month.

R: So, would you like that ugh to be delivered by the actual users or the app developers uhm would you like blogs to be posted uhm specifically by one of them groups?

P: I would initially think it would be the users for the blogs would be a good start.

R: Ok, so you would like people with an amputee giving a blog uhm about their experiences of using the app?

P: Yes, the users exercise experience and their disability for blogs would be good.

R: Ok, perfect! How about podcasts?

P: Yeah, I think podcasts would be a great idea uhm getting specific individuals to talk about the training uhm and the nutrition ugh what physical activity that they are doing uhm it would be good to have uhm obviously I’m sure there is some out there but disabled sport exercise podcasts so disabled athletes or people getting into it uhm and talking about their experiences uhm before they were disabled or since they become impaired and stuff like that ugh would definitely be good to have a breadth of different voices uhm would definitely be good.

R: Ok, perfect uhm do you think uhm because at the moment we don’t really know who the developers are uhm obviously I know who the developers are but actual users don’t know who they are.

P: Right.

R: So, do you think there is more that they could do from their side uhm doing regular uhm like monthly videos of them?

P: Well, yeah if there’s uhm well not necessarily monthly podcasts but maybe introduction video on the app uhm so stuff that we have gone through here today uhm stuff that I wouldn’t know without our discussion uhm maybe it would be good to have an introduction video on how to use the app and stuff like that.

R: Ok, so when you first log on you can see how ugh to register an account but do you think having an introduction video on how to use the app and how to navigate through could be helpful?

P: Yeah, an introduction video when you register would be amazing.

R: Ok, perfect so we will now just go onto the final little bits uhm so if you press more and scroll to the bottom uhm it says account settings.

P: Yeah.

R: If you press on that uhm do you like what is offered there?

P: Ugh.

R: So, you can select your impairment which is pretty cool.

P: Yeah, yeah that is obviously good and uhm it’s good that it’s tailored and ugh yeah the option to select your impairment is so useful.

R: In terms of exercise habits.

P: Yeah.

R: Uhm, at the moment you press something and it doesn’t really make a difference uhm is there anything in particular that you like to be added to that in terms of the actual habit section.

P: Uhm, well then maybe it tailors towards what sort of exercises you do uhm so if you’re new it will give some new introductory exercises to get you started uhm that’s back to the tailored exercise stuff that we were talking about earlier.

R: Perfect, ok!

P: Yeah.

R: Awesome.

P: If there is a way to get that linked to what exercises are in your suggested section or whatever would be a great idea.

R: Yeah. Perfect, ok so we will move onto the next one which is uhm measurement units uhm so that one is quite generic uhm metric and imperial depending on whether you’re American or not uhm managing subscription so uhm people that want to use the app in it’s full capacity uhm there is the option to do monthly uhm.

P: Yeah.

R: Uhm, I think yearly is sixty five pound.

P: Yeah, the membership cost is affordable.

R: Uhm, so it has got the option to uhm upgrade if you want to uhm which is pretty cool.

P: Yep, I think the cost of accessercise compared to the gym is so much better.

R: Uhm, so we will go back off that uhm so that is another thing to talk about is maybe navigating through is quite difficult on this section uhm but if we go onto the section that you were just on uhm which is the managing subscription section uhm do you think it was quite difficult to get off that.

P: Uhm, well I think compared to the others it’s the arrow up there I think just needs to be improved.

R: Right, ok.

P: I think that would be easier instead of having to fiddle around with this.

R: Perfect, ok and then you have got privacy settings.

P: Yep.

R: That’s quite generic so uhm account information and so on. Uhm, do you think that is all useful information there?

P: Yeah, that’s all fine yeah yeah.

R: Perfect, ok so that is done and then you have got the log out and delete section uhm so that is pretty much everything with the app.

P: Ok.

R: So, we will go through the last uhm three questions uhm so the first one I have got is uhm what are the potential positives if there are any of uhm using accessercise for improving physical activity levels uhm do you think it is something that is actually helpful?

P: I think it is definitely good for getting an idea of what exercise is to do uhm so for people that are completely new to it uhm and are not entirely sure what exercise looks like or uhm what exercise to do uhm having the massive list of exercises and then you can tailor it to what you want to do uhm it will definitely support users.

R: So, it’s improving the knowledge of what physical activity is uhm which is pretty good uhm is there anything else that you think is beneficial for people?

P: Uhm, I think the group option on the app is really helpful uhm and the social side to it uhm and seeing what other people with impairments are doing uhm sorry what did you say?

R: Uhm, do you think in terms of saving money in the long term the app is useful for that?

P: Yeah, I think that if you want to do your workouts at home and not having to go to the gym or finding a strength and conditioning coach uhm but seeing what exercises you can do through the app is great uhm with the videos I think are a big help as well.

R: Perfect, ok and what are the potential negatives if there are any of using the accessercise app for improving physical activity levels?

P: Uhm, like what I was saying with ugh being able to ugh keep participation going is definitely something that uhm because you can do one workout uhm you create a workout but then you don’t necessarily know how to progress it to something more difficult so you are starting out with the basic exercise but then I don’t know how you would then find the next exercise up once you start improving.

R: Right, so do you want more graded tasks uhm more difficult?

P: Yeah, I think more challenging exercises could help yeah.

R: Perfect, ok uhm is there any other negatives that you think could be mentioned?

P: Ugh, I think maybe just some of the ugh moving around the ugh social screen and being able to find people to follow will definitely be a good thing to have.

R: Ok, how about being ugh an older individual uhm maybe someone that is fifty of sixty that wants to use an app uhm they don’t really know how to use the app uhm do you think social media is something that may put them off?

P: Yeah, possibly yeah but then again because obviously there is different tabs uhm you don’t necessarily have to then look at that tab uhm you could just look at uhm the exercises.

R: Ok, and the last question which is quite important is how do you think the accessercise app could be improved if anything for it’s use in improving physical activity levels uhm so if you had to name three or four key areas uhm on what you have seen so far uhm that they can really use to improve it what uhm would they be?

P: Ugh, I think to encourage the consistency uhm to be able to keep people involved on the program ugh so they carry it on uhm I think being able to ugh progress uhm working out how to progress with the sets and reps or what intensity to do uhm and then being able to see the factors that make a gym accessible uhm specifically in terms of the accessibility ratings uhm would be really important future recommendations for the accessercise app.

R: So, are you saying what specifically makes the gym five out of five stars for accessibility?

P: Yeah, some more accessibility information on the map function would be good.

R: Perfect, ok and the last question is uhm is there anything else that you would like to add or discuss?

P: No, I think that is good.

R: Perfect, thank you so much Fin for participating uhm I appreciate all your help cheers.

## Transcript of interview undertaken with Participant 8

**Key code:**

R: Researcher

P8: Participant

R: So, thank you David for participating in our trial uhm first of all I will go through who I am, the purpose of this research and uhm what we would like to get out of the research study as a whole. So, I’m James Haley and my role within this research project is that I’m the lead investigator so I will be the person that undertakes the research, collects the data, analyses it and does the write up whereas the rest of the team will be supporting me with some of the analysis, providing feedback on the written content of the study and so on.

P: Ok

R: Uhm, the purpose of this research is to basically evaluate a novel smartphone application causes Accessercise uhm we would like to see whether uhm when the participants use the app over a period of time whether the app can support individuals to improve their health outcomes such as general health related quality of life, physical activity, fatigue, pain and so on. Uhm, I’m a researcher at Loughborough University uhm within the School of Sport, Exercise and Health Sciences and my position is that I’m a Doctoral Researcher and I’m specifically within the Peter Harrison Centre for Disability Sport which is based within the School of Sport, Exercise and Health Sciences. Ugh, the individuals that will be supporting the research and that will be working with me will be Sam Breary which is the director of Accessercise ugh Ali Jawaad who is the co-director of Accessercise, aswell as ugh Doctors David Maidment and Daniel Rhind which are my supervisors for my PhD ugh project. Uhm, if at any time you would like to stop ugh the interview then let me know and I would be happy to stop the interview.

P: Sure.

R: We discussed earlier about the information sheets, you have signed the consent forms which is great uhm any information about yourself will be confidential and no outside this research study will be able to access that information uhm and yeah so there are no right or wrong answers in the study and yeah do you have any questions before we start.

P: No, crack on.

R: Perfect! Uhm, so the first question I have got is ugh within the introductory questions which is please may you provide the following information regarding your name, your age, your gender identity, ethnicity and county of residence?

P: Yeah, uhm name is David Needham, age fifty two, uhm male, and ugh British.

R: And your county of residence?

P: County? Sorry I thought you said country.

R: Haha, no I mean county.

P: Leicestershire.

R: Leicestershire perfect! Uhm, so to start could you tell me how you first got involved in the Accessercise application and why?

P: In Accessercise application uhm so exercise in general?

R: No, Accessercise the application.

P: Oh, Accessercise uhm well you introduced me to it James.

R: Haha.

P: Uhm, I had heard of it uhm through sort of ugh probably I can not pick out the people but there are lots of people that I socialise with in the sporting arena uhm disability sporting arena uhm I am sure I have had people mention it to me before uhm so the name rings a bell as soon as you mentioned it but primarily you have introduced me to it.

R: Perfect, ok. In terms of ugh the advertisement for Accessercise has that been promoted online and have you seen it uhm around like uhm on Facebook and like general uhm social media.

P: No, not at all but it’s mainly come from you.

R: Perfect, ok. Awesome! Uhm, so could you please tell me your experience of using Accessercise so far?

P: I’m very much right at the very beginning uhm I have literally just started to uhm create my profile uhm I’m hoping over the next few days to actually start making full use of it.

R: Haha, ok. So, how long have you been a member for?

P: A member of?

R: Accessercise.

P: I think it was yesterday but ugh they uhm put the membership I think so ugh yeah.

R: Awesome! Perfect. So, now we are going to move onto Accessercise specific questions so uhm do you currently use Accessercise in your role and if so when and how?

P: Uhm, no it’s still early days uhm it is something I will incorporate into what I do uhm but I need to find my way around the app at the start.

R: Ok. So, compared to other apps and methods that you might have used in the past why do you think Accessercise might be beneficial and why do you think you would use it compared to maybe other apps?

P: Well I’m hoping it’s more specific to uhm the situation that I find myself in uhm most mainstream apps are for the mainstream people that are able bodied uhm hopefully uhm this will uhm be more specific to me.

R: Perfect, ok great. Uhm, so what were your thoughts when you first started using Accessercise application is there anything that stands out or you thought you know that the app developers could work on?

P: Uhm, just the bits that I have previously mentioned ugh the fact that ugh it’s incredibly hard to actually uhm pick out individual conditions and actually say you know this is specifically for that person that we mentioned before we spoke about the range of disabilities within cerebral palsy uhm the neuromuscular uhm conditions such as motor neuron disease could affect people in so many different ways ugh it would be useful ugh potentially uhm I do not know what difference it would make it if you were to modify the body and actually say I am affected here, I’m not affected here rather than actually approach it from a specific condition.

R: Perfect, ok.

P: And make a generalisation on the condition uhm if that makes sense.

R: So, do you feel like from previous apps it wasn’t very much tailored towards the impairment the user may have or do you think it was very generic?

P: So, certainly from other apps I have used and I have used a few I have just adapted on what is there in front of me to something that I can actually do uhm that is something I have just naturally done uhm hopefully that is something uhm I no longer need to do because it will be specific to the function that I have.

R: Ok. So, when you first logged onto the Accessercise app uhm was there anything specific that stood out to you which was quite different from apps? Like was some of the options.

P: Well, again I have only sort of uhm breezed through it uhm but ugh the one thing that was evident which you mentioned earlier was ugh the fact that the video have people in wheelchairs, people with the impairment that are actually doing the exercises, so I think that helps on many different levels ugh for people that probably aren’t uhm already ugh inclined so when they see that they may be attracted towards that direction uhm for people with disabilities it does help uhm certainly in my experience of being involved in disability sport uhm there have been several incidences where uhm there have been people that have already come across something that gets in your way uhm and they have found a way around it uhm and they will communicate that and it makes life a lot easier for everybody else coming in and finding that difficulty uhm the app hopefully will play that role ugh more broadly uhm and sort of uhm you won’t necessarily have to be in a sports team or a sporting environment to get that benefit uhm does that makes sense James.

R: Yeah, it does.

P: Yeah.

R: Yeah, that’s perfect.

P: I hope it does when you listen to it back.

R: Yeah, haha that’s fine. So, obviously the name of the app is called Accessercise so making sure it’s accessible for people with uhm impairments to get physically active based on what you’ve seen so far it does have the functions where you can locate local gyms down.

P: Yep, yep!

R: But do previous apps you’ve used have that function too. Do you think it’s something that Accessercise is good at because it has that function included compared to other apps?

P: Ugh, I have a friend that developed ugh a very similar sort of approach to accommodation and homing and things like that uhm two friends actually they developed this app independently uhm they have done a very good job of it but the uptake of what I understand was that it wasn’t fantastic.

R: Right, ok.

P: My hope would be that you guys find another way around.

R: Right, ok.

P: Uhm, the lack of uptake uhm I can not remember the name of the app but uhm one was very focused on the leisure industry, so it has included gyms but it is principally hotels and accommodations uhm and they have had to look elsewhere and I don’t want to put people off on what they are trying to do but they have had to look elsewhere and branch off and diversify a little bit from that rather than focusing on rating uhm and actual uhm individual sites and letting everybody else know they have had to go off and pull in other things as well to actually make that work.

R: Right, ok.

P: I could sit here for a very long time I will probably need to go away and have a word with him and find out exactly what it uhm has done differently uhm but my hope would be that it wouldn’t be far different from this app.

R: That makes sense, yeah.

P: Does that even make sense because I don’t even know exactly what I just said James.

R: Haha that’s fine don’t worry. Uhm, but with Accessercise obviously when you first go on you know you can choose your impairment you can choose your height and so on.

P: Yeah, but accessercise is limited at inputting data about you in.

R: Is there anything you first noticed when you started using Accessercise you feel you know it would be better if it had this or was there something you know the app developers could develop further.

P: Going back to my previous comment uhm and that is to approach dysfunctions.

R: Right.

P: I think focusing on various dysfunctions would be uhm very good uhm if that was something do able and then base the exercises around the results of what is being returned uhm I think you will get something that is far more useable if that makes sense.

R: Yeah, that makes sense. So, just following on from a comment you mentioned earlier about adherence to apps you know uhm so one of the barriers to physical activity for people with disabilities is the lack of knowledge of physical activity so that could be down to a lack of advertisement of the app uhm do you think from your experience of previous apps they were not advertised on the Apple Store or Google Playstore as they should be do you think there is more that the developers could do to push the app out further?

P: From a purely Accessible app perspective or just apps in general?

R: Just physical activity app for people with impairments.

P: Uhm, I have to admit I have only seen one of them advertised.

R: Right, ok.

P: Ugh, and I wonder whether that’s a financial thing uhm in that it costs money to advertise it and actually get it out there uhm it would be interesting to see uhm what methods uhm the developers use but you know ugh associating yourself with the correct organisations that uhm already support people that you are trying to uhm bring in to use the app would perhaps be the route to go down rather than general marketing.

R: Yeah, of course.

P: Uhm, just through apple app store and things like that.

R: Yeah, that makes sense.

P: But then what do I know uhm you know it’s ugh.

R: I think with these sort of apps they are so knew that there are always things to improve on but from my experience of uhm exercise apps for people with disabilities is that there are not many out there at the minute.

P: There is uhm and I’m trying hard not to make negative comments within the disability arena but there is a lot negativity and anxiety.

R: Ok.

P: Uhm, if the app could find a way of overcoming that would be great uhm for example I’m in a wheel chair I can’t go to a gym uhm that sort of negative attitude that I can not do this I can not do that uhm and so if the Accessercise app could find a way of bypassing the negative attitude that a lot of people in this situation have to sport and activity uhm I think that would be huge.

R: Do you think for example going back to uhm the social side which I showed you on the app do you think that might support people to get over that initial feeling of stigma in which they can’t do it.

P: I think the fact that the Accessercise app is showing videos of people already in that situation with that disability doing the exercise that’s bound to help.

R: Yeah, of course.

P: I hope I’m right but I think there might need to be a little bit more ingenuity there to get people in uhm and I’m speaking from my perspective and that’s because I’m the secretary of my local wheelchair rugby club and it can be so very difficult to convience people that you can see the benefit form having a go and transferring from the day chair into a sports chair and having a go you know that within five to ten minutes they will really be enjoying themselves it’s just trying to get them to make that step which is the difficult thing uhm and hopefully the Accessercise app can allow improvements in that uhm but so far I do not know the answer.

R: It’s also making sure that the environment and the location they feel comfortable with.

P: It might be exactly what is required and time will tell so fingers crossed and hopefully from there it will attract them to take a step out uhm go to the gym uhm and put things to one side because what you will find is that with the exception of one or two individuals they are only interested in themselves uhm but the general environment is actually a very friendly environment and a very positive place to be.

R: Exactly, yeah. It’s getting over that initial barrier.

P: Yeah, I hope.

R: So, this question slightly repeats what we spoke about earlier but from showing you the app uhm and going through it with you uhm are there any improvements that you believe could help improve the Accessercise application?

P: Other than what I have previously mentioned uhm but we have touched on groups but I think there is a group function in there that I have not properly explored but it would be interesting to explore that and comment again.

R: So, when you say group do you mean opportunities to exercise in groups?

P: Yes, absolutely! If you have got uhm a social group of some sort uhm where everyone is active to some degree uhm and the example that I would pick up is uhm and I’m not sure if you have seen the doggy aid.

R: No, I don’t think so.

P: So, that’s very much Motor Neuron Disease focused uhm and he does an annual thing where they encourage and to participate in various different exercises uhm but they can form there own groups uhm within that so you join pre-defined group but within there you can actually form your own sub groups uhm and that is more of a social thing so you can compete among your own peers so that does encourage people to exercise and there is no two ways about that uhm it pushes you and you actually want to compete uhm with friends and family.

R: Yeah, I agree with that.

P: I think group work would be a great function and you do not even need to be with them if there is a group function.

R: That is true yeah.

P: You can be on one end of the country with a different group trainer uhm for example you can say I’m going to go and do this uhm today but actually you have a league chart uhm or awards which shows that you have completed a certain amount of exercises, beaten my daily record and so on uhm within that period of time ugh and you can see things adding up then anyone in that social group can see that and become motivated to challenge that uhm and I’m going to go away and do more and it’s a way of incentivising each other without actually doing anything you know uhm I think that would be a good add if that was possible.

R: Because I think about like iPhones, iWatches and stuff uhm when you walk a thousand steps or something and it notifies you saying well done great you have completed your daily task uhm so it would be good to see something like that on this app you know uhm over time you get a trophy which you can then put on your profile where other people can see you know moving up and down the leader boards or something like that uhm or even doing like monthly challenges where if you participate.

P: Yeah, I’m not hiding from the idea at all but the idea of rewards and so on uhm could be demotivating for some people uhm for me I probably wouldn’t blink at it but the idea of having a table or a monthly competition uhm and trying to get to the top of that table would very much motivate me.

R: Yeah.

P: So, it’s all down to the person you are dealing with uhm so if you can incorporate a bit of competition I think that would be great.

R: Yeah, I agree with that. It’s sort of like when I think about like a Playstation or an Xbox uhm you can play the game through the campaign or single player or you can go online and play with other people so uhm if it’s got that function available where you have a bit of both worlds with the single and online functions would be good.

P: Absolutely! Yeah. An online function to exercise with others would be good.

R: Yeah, I agree with that. But going back to friends and family uhm do you think there is anything that the app could do to incorporate care givers you know uhm like family uhm so for example is there anyway the app can allow close relatives to keep an eye on the individual or more support that way if that makes sense?

P: Uhm, could you elaborate?

R: So, for example could a family member be able to log onto the app but not actually use the app but actually monitor one of your patients, friends, family members progress and provide them with some sort of support on there?

P: Yes, it’s certainly something that could work uhm I have got a friend that is a personal trainer uhm and he has an online set up and he does exactly that uhm but you mean more broadly uhm anyone can encourage anyone uhm so we have done the incentivising uhm now the sort of ugh the encouragement uhm yeah I think for certain characters that would work uhm for me it would very much there is a table, there is like a league table to get to the top but again that is me uhm that would be what would work for me uhm and to have people to pat me on the back would not be something that would uhm motivate me.

R: Ok.

P: But then again that’s me uhm I could see that working for a lot of other people that I know uhm in fact the vast majority uhm like I’m thinking of other people that you do naturally encourage when you are in the gym environment uhm that probably aren’t uhm as confident uhm and that sort of thing where you get the odd comment and a pat on the back and so on would mean the world to them uhm so that would work with most characters.

R: Uhm, just thinking back on you know with the gym so uhm the Accessercise app has the option where you can select the gym and uhm you may want to go to the gym with a friend but Is there anything that you would like to see from the app that can support the individual while at the gym? So we saw the function earlier where it says tick the box if you require assistance but is there anything more that the app could do in terms of supporting individuals in going to the gym uhm so like is there anything else you would like to see included on that?

P: Yes, but I guess there will need to be a lot of buying or support from the gym organisations on that behalf and there would need to be the incentive there for them to do it and that would be to get more membership through the door uhm I would imagine but I guess you could take this in the direction we discussed earlier where a uhm a member of staff is aware and even notified that a customer on site requires assistance and the staff know how to support them but what degree you could do that uhm I’m not sure but there is always the option to exactly open your mouth uhm particularly the gyms that I use and every now and then there are no staff on site uhm and when they are there uhm for instance when I want to use one of the bits of kit where the uhm weights uhm the free weights but you have to lift the weights on and it’s sort of out of reach or to actually pull the actual uhm weight down to a height that I can actually reach.

R: Yeah, I know what you mean.

P: Uhm, and hopefully the app can take away that problem but I really doubt that all gyms will be happy to provide that kind of uhm support I think uhm there expectations would be that if you want that kind of support uhm you would need to bring someone along uhm with you to assist you in that exercise uhm so the only thing that you could potentially do there is through the app is to encourage gyms to allow uhm a person to brought in to act as that function if the gym wouldn’t be prepared to do that themselves uhm but you know we could carry on talking about this forever.

R: Yeah. I have just had a thought in my head thinking uhm so on the app you obviously have this section where you can see what gyms are accessible and it’s linked through google maps but it would be interesting to see whether the app in the future could link up to the specific gyms uhm to sort of have a collaboration with the gym or even the staff within the gyms.

P: Certainly. I think if uhm accessercise could work with gyms would be good.

R: For example, I’m not sure uhm so let’s say you pay a little bit more money each money for membership which let’s be honest is quite a challenge sometimes for people but that function can allow someone in the gym to come over and support you when you press the button on the app or what the gym could do is to provide a number like a WhatsApp number where you can message and someone from the reception can come over and support you because for example if I needed support in the gym and it hasn’t got that function then I would just shy away from that exercise.

P: Yeah, and also shy away from the gym.

R: Exactly, yeah.

P: Yeah, that’s a good point.

R: Yeah, so it links up with google maps but it could link up with PureGym, it could link up with other companies like The Gym where they know this app is being used uhm and the people that use the app can get the support from the gym instead of a caregiver or a family member having to come along too to support that individual they can save a bit more of their own time.

P: Yeah, I know what you’re saying.

R: Yeah because it works both ways really uhm so a specific care company is paying a care giver to go and help that individual at the gym or could that money saved a little bit where the company is being paid a little bit more but it’s a part of there duty to go and support someone who presses a function on the app.

R: Absolutely! There is that side there is additional financial reward potentially from that perspective uhm there is also additional financial reward will attract more people uhm within the disability arena to get into that environment uhm but you wouldn’t particularly need to change membership fees too much as its cheap.

R: Uhm, I guess this method could help advertise the app so if they want to develop the app further and get it out there to a wider audience then you would want it to be linked and collaborated with the gym itself.

P: Yeah, exactly. It’s because there are so many independent gyms so it’s how you manage that uhm so the gym would have to buy into the app uhm not the other way round uhm it would have to be word of mouth uhm smart marketing I think to achieve that ugh if it’s possible then why not.

R: I couldn’t agree more. It would be very interesting to see uhm sorry this question follows on from a pervious question so the final question for this specific section is uhm do you use any other similar applications outside of Accessercise and if so what is better or worse than Accessercise?

P: I have used bog standard exercise apps uhm I wouldn’t say religiously I use them because I tend to do my own thing in the gym uhm I mean I have been going to the gym for a long while so I have a good idea in my head on what I’m going to do and what day or what week and so on uhm what I’m hoping the app does is to give me that little bit more encouragement and again provides the option to record exactly what I have done in the gym uhm it is too early for me to properly comment on that but it would be a question that I can possibly answer next time which we can have a chat about uhm but I don’t know if it has to be that different because even though there are disabilities uhm I think a big part of disability is finding the way to do exactly what able bodied individuals can do and finding ways to achieve what everyone else can achieve uhm and people with disabilities have always been at a disadvantage and I do not like that kind of language uhm it’s just not the way I think and I do not think it’s helpful uhm and I think that’s part of the problem that stops people uhm perhaps from trying to get involved in what you’re trying to push now uhm and they will think what’s the point I probably can not do that anyway uhm whereas there are a lot of people out there who think uhm this problem will not hold me back uhm I’m going to find a way of doing this uhm one way or another uhm but the app should try and focus on applying to both types of people.

R: It would be interesting to see that.

P: Yeah, yeah.

R: It would be interesting. Ugh, sorry last bit but when you enter into the Accessercise app uhm you start by putting your phone number or email address and it provides you the option to put your height, weight.

P: Yeah, I’m just looking at that now.

R: Do you think there is anything else that you would like to see specifically included onto the app? For example, BMI information, energy expenditure, water intake or anything like that included?

P: Yes it would be useful information uhm to have BMI and energy expenditure.

R: But do you think anything specific would benefit you that you would like to see included?

P: Haha uhm as an individual now uhm as a fifty two year old uhm not necessarily but that is not to say that I do not think it’s a good idea that the information you mentioned is there uhm so if you’re an athlete who is looking to improve and measure yourself uhm if that is something uhm you can frequently measure uhm over time then there’s no better incentive so yeah potentially I think for the wannabe athlete yes uhm for the social exerciser I think just the ability to interact and get involved with the people around them ugh that they are doing things with is possible uhm I do not know if the figures will interest them.

R: Yeah.

P: Again, I could be wrong I’m just saying that they should be in there as I think it’s relevant for a lot of people and not so relevant to others uhm but why not put it in there and leave it as an option.

R: Yeah, makes sense.

P: So, if someone wants to appear on their profile to give them a wider overview of where they have come from and their personal exercise goals then fantastic.

R: So, they have the option to include them.

P: Yeah, if someone is not really that bothered then they can tick a box and it’s not on there anymore but I think a lot of these apps particularly the most successful ones are flexible uhm as possible as they can be uhm to incorporate and be whatever uhm as many want it to be if that makes sense.

R: Yeah, that makes sense yeah. Uhm, so when I showed you the social function on the Accessercise app where you can react to friends workouts and even comment on them uhm is there anything else you would like to see developed further in terms of the social elements uhm so you can react to workouts providing emojis and love hearts but maybe if you don’t agree with someone’s you know they have not performed very well you can provide an unhappy or a sad face sort of like an emoji sort of thing or anything you think could be encouraged further.

P: It’s a tricky one to answer I mean if you were to push the group element of it where you know you are with people that would take things in the right way uhm the ability to have gym level banter could be uhm to an outside exerciser quite dreadful sometimes but sometimes it can incentivise the individuals within the groups that no one else can see what is being commented where you can provide pretty much anything as long as it doesn’t over step boundaries.

R: Yeah. So, you would like to see a bit of banter between the group members?

P: It works for me uhm if someone comes to me and says your performance was awful that would make me laugh and incentivise me but it would not do that to other people uhm it’s case of making sure that all those different levels of communication is available to you but it can only exist within groups that are acceptable uhm whether you can actually achieve that or not is a different story but you know it would be very interesting but I think you will motivate different types of people with different types of language ugh you know but for some people it’s about going well done, you did great and it really does mean the world to them you know uhm but for other people they will find it quite descending uhm but that’s where I’m coming from so if you can create your own groups and sub-groups on the app that might give you the ability to be flexible on what is and what’s not allowed.

R: So, when you say groups do you mean uhm putting users into groups where you are with other people with the same impairment or just groups of friends?

P: No, so groups of people that want to exercise or want to make uhm what they are doing partially public uhm you would want to make it fun, most people would be happy to say I have done this today uhm and make that public for everyone to see but you wouldn’t want uhm let me provide an example and I have to be very careful here but you know you wouldn’t want something on there that says you know that was shit because to the wrong person that would be demoralising.

R: Yeah, of course.

P: But to me that would be exactly what someone would say to me so it’s a case of probably catering for various populations that have lived their lives in the sporting environment and they know that even though it’s offensive language that gets used it’s motivation saying it to keep them a bit of a kick to encourage you but that could be misjudged sometimes by the wrong person in a general environment.

R: Ok.

P: So, is there a way of actually accommodating both approaches there uhm that is the only thought that I have got uhm and the honest answer is perhaps not because you have to be very careful if you have these app platforms uhm what sort of language is used because it only needs to fall on the lap of someone who doesn’t understand it all but it’s a tricky thing to do uhm so what is acceptable to me would not necessarily be acceptable to someone else.

R: Yeah, I agree. You have to be extra careful.

P: You have yeah but yeah you should come to my club sometime.

R: Haha.

P: You should see some of the language that gets used but for me that is a proper environment uhm but it’s done with respect and if there is someone in there that won’t necessarily understand the banter uhm people have got enough about them to not do it uhm and when that individual moves away then you can go back to that environment and it’s sort of the case that it is this but you have got to make sure that you cater for whoever is present.

R: So, you think group work on the app is important?

P: I think so yeah and if you could separate of into sub-groups then you can change your approach because if it’s just general and you put something up which goes back to what I just said uhm something that would incentivise me may demoralise someone else and if it was public it would be viewed badly and it wouldn’t have the impact that you’re aiming for because it would put people off and you don’t want that so I think sub groups is a good idea.

R: Ok, awesome. Thanks for that feedback. Uhm, right so we are now going to move onto the think-aloud protocol so this is the main part of the interview and basically I want you to participate in thinking aloud while you’re navigating through the Accessercise app on your phone.

P: Ok.

R: So, like earlier when I was showing you through the app I would like you to do that and basically tell me what you are thinking when you use the app.

P: You’re asking me to do two things at once haha.

R: Haha. So, basically the instructions for the think-aloud protocol are straight forward I would like you to say out loud what ever comes into your mind and there are no right or wrong answers we will run through a quick practice so you get used to using the protocol.

P: Ok.

R: So, can I ask you to say out loud everything that comes to your mind when you think about physical activity?

P: Uhm, hard work ugh exhausting uhm but at the same time ugh very satisfying, rewarding uhm it has huge benefits that impact not just on your physical health but your mental health as well uhm and it’s a sporting environment that I have always loved.

R: Ok, great! So, if you can go onto the app for me and show me uhm sort of what your screen looks like?

P: Ok, I’m just on the profile section now.

R: Haha, ok. Uhm, so what’s your first impression when you see the first page uhm so like when you see your name, there’s an option to include a photo uhm is there anything that stands out that you like from this screen that you are seeing?

P: It’s intuitive uhm the fact that anyone can go onto this and it pretty much follows the same sort of rules that an awful a lot of apps use so you could uhm navigate around that very easily uhm are there anything else to add uhm I think that’s a question to throw at me a little bit further down the line.

R: Ok.

P: Uhm, you don’t want to go over the top with all this information it want’s to be very basic.

R: So, you think the app is good when it’s basic and it’s not too complex?

P: You don’t want too much information ugh for example the if the pages were very long and detailed people will lose interest so the fact the Accessercise app has basic information and the pages are short that’s good.

R: Do you think the colours that they have used on the screen are suitable for example like a god match or is it too white?

P: It follows the pattern of uhm the logo and you will get different people’s opinions uhm but I think there is absolutely nothing wrong there.

R: Ok.

P: Uhm, the grey background maybe that might be a little difficult for some people uhm with visual issues.

R: But with for example uhm people with motor neuron disease having to use an app do you think this is something that you would get tired of using after a while like having to navigate around the app because you said earlier how you go to the gym and just get on with your workout and then come home uhm do you think using the app and actually over time it might get a bit boring to use or do you think it’s still motivating in the fact that you still have this option?

P: I think the recording element of it uhm is something that I will definitely make use of and that will continue over time uhm and referencing uhm if I wanted to introduce a new exercise into a routine uhm it would probably be a good reference to go onto the app rather than actually have to sit there and ponder and look at ugh different bits of kit uhm and then turn that into something that I can actually replicate on a piece of kit uhm you know at the gym that I’m at and you can add it in uhm I mean that’s again too early days but it would be nice to use the app a couple of times uhm create the exercises and hopefully it will allow you to do machine type exercises uhm I mean does this?

R: Yes, it does.

P: It does, yeah. I mean as long as it covers most of the mainstream uhm exercises and so on uhm I think that’s great uhm we are going to do this again aren’t we uhm at a later date and I should be able to give you a better answer.

R: Ok.

P: After I have had a good few weeks making use of it uhm the big thing for me is the recording element of your workouts uhm because a lot of the things that I incentivised myself are based on improvements that I can see.

R: Right, ok. So, you like the feature on the app where you can record your data and you view your data.

P: Yeah, you can view your data uhm is there a chart function?

R: Ugh, I have not fully looked into it yet but I know that you can track your uhm your progress you can see over time how many reps and sets you have done.

P: Yep.

R: Uhm, and there is a calendar function and stuff like that where you can look back on different days that you’ve gone through.

P: So, is there a function in there that can record how long it has taken you to do?

R: So, it has a timer function.

P: Yeah, it does have times?

R: Yeah, it does.

P: Yeah, you’ve shown me that.

R: Yeah, haha.

P: So, you can set the timers but can you record the time that you’ve uhm taken so like stop and start uhm but is there a stopwatch for example you can start an exercise?

R: I believe it has that function. I believe it has a timer and once that is done you can tick it off and move onto the next exercise.

P: Right, ok.

R: Uhm, it does have the sort of functions but what I would like you to do is to go back onto the Accessercise app and go onto the main page uhm I would like to see you navigate around uhm so obviously when you open up the app this is what will come so do you like the idea that you can find new friends on the app so for example if we press on find new friends.

P: Ok.

R: It comes up with ugh kate, so if you press on Kate’s profile.

P: Right, ok it’s come up with this.

R: It now says find more friends do you like the idea how you can follow people on there and how they can follow you back uhm do you like that social side to it?

P: It certainly would be great if there are other people that uhm I knew well uhm yeah I would do that on other apps uhm with bits and bobs on things we do so yes I would make use of the find a friends feature.

R: Ok.

P: Again, this would be an area where you potentially could have the sub-group function.

R: Ok.

P: So, you have a public section where you like and a smaller social group that would be useful.

R: Ok, so you have the option to follow people, so if you press on someone’s profile and it comes up with uhm the option to lock your account which is quite cool so if you don’t want to be so public about your profile and what you do it has the option to lock your account do you think that is a good option to have?

P: I think that is something that you would need to have on the app because if you were to have a person that is incredible private you would have to but I don’t think I would make most use of that myself unless there was people on there that uhm I had issues with uhm but that wouldn’t be too much of an issue.

R: Ok. So, if we go back so if we wait for this list to load or we can press kate again and it comes up with a list of more users uhm so obviously here it has the achievements uhm so what are you initially thinking when you see the achievements option so obviously at the moment this section is locked but what would you like to see in the achievements would you like to see daily record, weekly records.

P: Yeah, I want accessercise to provide daily, weekly and monitor challenges.

R: Achievements?

P: Uhm, yeah so like awards that you mentioned earlier which I think would work for a lot of people uhm if there was a summary provided at the end of what exactly has been done uhm this week uhm what exactly has been done today.

R: So, like a daily or a weekly log?

P: Yeah, so what they have done daily, weekly, monthly.

R: Ok.

P: If you could see that and again uhm that would be incentivising uhm or could be uhm incentivising uhm if you were someone that was competitive.

R: Ok.

P: So, yeah, I think that would be good to have daily, weekly and monthly goals.

R: So, linking in other achievements so let’s say linking it to the shop feature would you like it to link into the shop and be able to get rewards for doing a certain amount of exercise or discounts or anything like that?

P: I like the idea of the discount you mentioned earlier yeah if uhm that could be something that would be financially available to ugh users than yeah why not that is a really good thing but it really depends what they put in the shop but yeah if there is discounts on the app that would be great but it works both ways uhm you are more likely to get involved if someone thinks they are getting a good deal.

R: Yeah.

P: So, that could work both ways.

R: So, it has the achievements which is great so it has that encouragement and the followers but do you like the fact that you can see who followers your friends and so on?

P: Yeah, that again uhm it’s nice to follow people and they can follow you.

R: It all comes down to a privacy setting.

P: Yeah, absolutely I think that is something that is great if you are happy for people to see who you follow uhm from an individual perspective you would have to give them the option to say whether people can see who you follow or who is following you uhm it is not something that I would be sensitive to.

R: Right, ok.

P: I think I would be quite open for anybody to look uhm they can look but uhm but that might just be the development of the privacy function as you say uhm it might be something that they focus on more.

R: Yeah, that makes sense. Do you like the fact that ugh it provides your age, city, stuff like that?

P: Yeah, again there needs to be a more diverse function available where you can leave some type of information in and take some out uhm again I’m trying to look at it from a bigger perspective I don’t think it would be too much of an issue to many males but it might be a big issue to a lot of females.

R: Ok.

P: Uhm, you wouldn’t necessarily want people knowing what city you are in, what gym you are in and so on uhm it wouldn’t bother me but I think it may bother a lot of other people uhm so I think the ability to manipulate what is available uhm would be sensible.

R: Yeah, you only put on what you want to see which is pretty good. So, if we go back to the main screen and that’s all the friends stuff which is good but then you have the option in the middle for exercise so here you have got uhm my exercises, my favourites and my workouts so you can select something uhm so what do you think about this the option to have a video demonstrating the exercise and it has descriptions on it uhm do you like that?

P: Yep! That’s really useful.

R: Do you like this then?

P: The idea that it tells you where to align the equipment up on your body is pretty good because ugh obviously to get optimum sort of ugh muscle usage uhm you need to get it accurate.

R: So, in terms of the video and the description do you think the app developers have developed it well for different impairments would you say the fact that some individuals in the video have an impairment are slowly going through the exercise and telling you how to do it but also providing sub tittles for you know uhm.

P: It certainly covers a lot of bases there uhm.

R: Is there anything that you think they could do better with the videos?

P: Uhm, we have focused previously on uhm associating the impairments or the fact the impairment is visible uhm I’m looking at this video and I have no idea.

R: What impairment?

P: What impairment this demonstrator has.

R: Ok.

P: Ugh, it’s not a big deal for me but I think it would be a big problem from other people who look at the video and think well that may be an able bodied person and what relevance is that to me.

R: Yeah.

P: Well, it’s completely relevant but there first thought may be is that this is not for me.

R: Right, so do you think the fact that if they can not clearly see all of the instructors in the videos that may stop them from exercising?

P: Possibly, I think uhm I’m not sure how exactly you would manage that because you have such differing impairments uhm it would be nice to see in the video the person with their impairment every time so at least you can then relate to it.

R: Ok.

P: Uhm, the fact there’s a description of the exercise there and it is quite detailed is good.

R: So, do you like the fact that it provides diagrams of the muscle groups being worked?

P: Yep, the diagram of the muscle groups is fantastic.

R: So, your first impression when you see all that feedback there and the description of the exercise do you like it and how it’s provided?

P: Absolutely! You have got useful tips of the exercise and again in a box that is very short and concise that is good uhm the diagrams there for the muscle groups you are working.

R: It also has a filter section so you can filter it depending on what you’re looking for. So, I think what it does is based on your goals and uhm where you want to undertake physical activity it will then select the right exercises based on that information.

P: So, that’s a good example uhm this video here straight away oh it’s Will.

R: Do you know this person?

P: Yeah, haha I know him.

R: Oh, does he play for Leicester Tigers?

P: No, no he’s uhm at my local rugby club he’s a really good lad he’s one of the loveliest people you’ll come across.

R: So, going back to what you said earlier so you think this video is fine because it provides easy demonstrations?

P: Straight away you can see and relate to the demonstrators impairment but then you are dealing with such a broad spectrum of disability you can not apply a video for every single person in every situation uhm I appreciate that but it would be nice to be able to visualise as soon as the video starts and you can see that the person uhm has this disability whereas some videos above weight it may be because they are a double amputee but it would be nice to see that though easily.

R: Right, ok.

P: Uhm, I think a little bit more effort would make it more appealing to more people.

R: Ok. So, if we go onto the next one.

P: I thought this was what Will was involved in because when I spoke to the guys earlier, they said I don’t think so.

R: So, as you can see it has demonstrations, it has descriptions and I’m sure Will may motivate you to use this app now haha.

P: Haha, yeah I will give him a hard time about it when I see him next.

R: Haha.

P: Where he has come from is quite remarkable not a particularly uhm sporty person but he has done tremendously well uhm you will see him up here in Loughborough at some point.

R: Yeah.

P: Uhm, he was up here the other week actually.

R: Do you think he would be interested in taking part haha?

P: Uhm, he’s doing an awful lot he is based further down south he is obviously involved in this bur e’s doing a few other things as well uhm there is a watch monitoring device that has been developed specifically for uhm disabilities and he is involved in that which was developed by a University on the south coast.

R: Right, ok.

P: Hampshire, I think.

R: Sussex or something like that.

P: Yeah, maybe somewhere down there uhm but he really is a good lad uhm no he was up here for a disability triathlon day up here not too long ago.

R: Right, ok.

P: He gave it a blast and I think he’s struggling with his wheelchair racing events at the moment but he really enjoys it all as he has the right attitude because if he decides he’s going to do it uhm he will do it and be very successful and he’s young as well but I wouldn’t be surprised if you see him up here definitely.

R: So, if you were to use the Accessercise app tomorrow how would you go through uhm would you start looking at the exercises, would you start looking at you know changing the filters?

P: Initially what I would do is I would go through and I would pick the exercises out that I uhm I can use.

R: Ok, so you would build a workout plan?

P: I would build that and you know as close as I could uhm create it around the routines that I have already uhm and get used to that and from there I would do the recordings hopefully be able to visualise from it with the uhm app and what I get from it uhm start making improvements uhm and other time add bits in using the app because then I can search through uhm the exercises available and I will think like ok I can add that in I can do this routine quicker now I have an extra twenty minutes at the end uhm I can add this one in uhm and start from there so that is useful.

R: Do you like the notification options and how you can link your workouts to the calendar?

P: I have seen that and I still need to have a play with it but that is a good idea the calendar that it ugh it will give you a little bit of a kick ugh on this is what you have to do today and you need to get out of bed and do it now uhm that is definitely a good thing.

R: And there’s another option down there on the bottom I think?

P: Ok.

R: So, when you first see this option so the map what comes to mind when you first see this?

P: It’s just google maps isn’t it but you mean beyond that?

R: Yeah, like the function within the app that you’re seeing so for example the orange is the accessible locations do you think that is something you will use and even something that could benefit other individuals?

P: Uhm, initially I would take a look at it out of interest to see uhm how many accessible gyms there were or accessible venues that uhm offer the activity I’m looking to do ugh because I don’t do anywhere near as much travelling that I used to uhm certaintly as a younger person I used to travel around uhm so for example if you’ve had a days work and you want to use a gym and you’re down in London not familiar with the area you can easily look it up and find somewhere local very easily and I’m guessing if you click on it you can get contact details uhm which is good.

R: So, when then pops up what are you thinking uhm do you like the fact that it has the option to phone the gym, it has the option to see the opening hours of the gym, but do you think there is anything that they could include further?

P: I think the app is good because it actually has the rating of accessibility for each gym but it would be nice off the back of that you click on the gym and you can directly see people’s reviews.

R: Right, so linking it onto google feedback?

P: Yeah, so if anybody actually has written a review about the gym you can click it and see it.

R: So, for example do you like the fact that it provides the stars for how accessible the gym is so you can see here this one has three out of five.

P: Yeah, so straight away that gives you a summary and you can see what other people think about it.

R: Ok.

P: But if you have two of the same ratings it would be good to have the function where you click on it and you can say I’m a wheelchair user and I found this gym particularly good or uhm everything was great which you can easily make a decision on using that gym based on other people’s uhm reviews.

R: So, you would like to see the comments that users put in for each accessible gym?

P: Yeah, it would be nice to have a little review section which helps explain what is behind the start rating system and it’s visible to all users.

R: Ok.

P: Yeah, most people that go in and do these ratings there’s a star system and there’s a comment box and a lot of people fill the comment box in uhm sometimes a lot of things that people put in there are not useful but sometimes you see something that says uhm you know I came across this issue uhm but the staff were great they supported me and that uhm would encourage you to perhaps go there because you know other people have reviewed it well even if you struggle with something the people that work there would be quite happy to help so yeah it would be nice to see comments of the back of that.

R: Hmm, yeah.

P: Uhm, whether that’s possible I don’t know ugh. So, why does this actually have the full symbol sign?

R: I think it’s because there are four gyms within that area, so when you zoom in you can see more.

P: Oh right, so that looks like an individual spot.

R: I think the reason why it’s like that is because the gym has been classified as accessible for users.

P: Ok.

R: Whereas others are still being reviewed and checked.

P: Oh ok, I’m with you so that’s the difference.

R: The one with the Accessercise logo shows that this gym here is accessible.

P: So, I’m guessing someone has reviewed it and it’s definitely accessible.

R: Yeah, whereas the other ones they are gyms in the area but they are still to be reviewed.

P: Ok, yep.

R: So, would you like to see an option where they provide a phone number, and you can press on the phone logo and it goes straight through to phone the gym instead of copying and pasting the number from the app onto your phone?

P: Yeah, definitely! I think that would be quite easy to use and I think that’s something they could quite easily do uhm it depends on the actual phone but that seems pretty easy to do with the iPhone.

R: So, you would like that function?

P: Yeah, uhm that would be great and if it links to the gym website.

R: So, you would like a link to the website?

P: Yeah, and you can also take a look at the gym and you can see any online features that they have got uhm you can click on it that would be useful uhm and the only other thing that would be useful would be photographs of the gyms.

R: Photographs?

P: Yeah, photographs uhm if there is an option on there where the organisation of the gym can upload photographs of the environment and actually visualise the gym before you use it would be pretty good.

R: Yeah, I agree with that.

P: So, you can actually pick the bits of equipment out that you want to use.

R: So, you would like a birds eye view of the gym?

P: Yeah, definitely. Absolutely or one of these virtual walk arounds functions.

R: Ah, ok virtual walk around.

P: Yeah, a virtual walkaround of the gym would be great so you can zoom around and have a quick look of the gym and the equipment.

R: Yeah, that’s a really good point I agree with that.

P: Again, you don’t want to overcomplicate it.

R: Like a video demonstrating what’s included in the app.

P: Yeah, straight away uhm I do not think you have seen it but these virtual walk arounds are becoming very common with house sales and things like that.

R: Yeah, accommodation and stuff like that.

P: You think with something like that with that sort of technology would be able to expand into something like this quite easily.

R: Yeah.

P: It is just a particular type of camera and software that you just walk around and uhm that’s it job done uhm yeah.

R: So, you like that function?

P: Yeah, I do, I would use that if I was out and about in a place that I was not familiar with uhm, and it has got quite a few which is encouraging uhm I would imagine at the minute based on what you’ve said these are gyms that have been identified.

R: Yeah, they are general gyms.

P: So, over time they need to advertise themselves.

R: Yeah, so over time they will get the little icon uhm.

P: And that based purely on someone’s review then is it?

R: Yeah, I think uhm the app developers have been there and they have identified it to be accessible.

P: Ok, would they work with people or organisations that might have already done this?

R: I would like to think so uhm but I think the app is still in it’s really phases of testing so there are still things to work on you know.

P: I may already know somebody who has done something similar to what you guys are doing uhm not necessarily for fitness but more for hotels and leisure facilities.

R: Ok.

P: He may even collaborate with your team and give them an awful lot of information that they would otherwise have to go and pay themselves.

R: Ok.

P: This guy has basically done an app that is basically the same sort of app like yours but has a review section behind it.

R: Right, ok.

P: So, you can basically review anywhere and I’m sure your team could take advantage of working with someone like that if they wanted to.

R: Great! So, let’s move onto the last function in the bottom right uhm what do you think of these sort of things so you have like the shop, the nutrition section and do you think this is good and quite supportive?

P: Yep, again because it is a single page that’s perfect! You don’t want to be scrolling up and down so I think your team has done that well.

R: So, you’re saying having everything together on one page?

P: Yeah, I think having all the content on one page is great uhm and I don’t think they can improve on that uhm but I have to admit the app is a bit plain looking.

R: Ok, so do you think having a bit of colour would help?

P: Yeah, a bit of colour or some things highlighted in there but overall the information, the layout of the app is fantastic uhm the risk there is that if you add more things it would start making things drop of the page I think uhm and I don’t like that and I think a lot of people don’t like that uhm I’m sure there is a way of sort of navigating where every page you go to all the information is required on that page will be conveyed on that first screen that pops up.

R: Great, ok. Fantastic! In terms of the shop and uhm the nutrition options is there anything specific that you would like to be included so obviously these options are coming soon and there are things that the developers are focusing on but in terms of the shop is there anything that you would like to be included on that?

P: The nutrition would be interesting to seem uhm because if you eat well then you’re going to be more healthy which is really important but that is one thing that I do let myself down on uhm so having that would be good uhm if there’s a way to incentivise users to log onto the nutrition section would not be a bad thing uhm but it is something that people overlook except for perhaps the elite level athletes but I think in general it would benefit everybody and to improve their nutrition uhm you mentioned about incentivising uhm via the shop uhm so maybe you could do the same via the nutrition section uhm but I can imagine it’s something that they will charge for.

R: Possibly, yeah!

P: So, maybe uhm.

R: I think the nutrition is more about nutrition plans, healthy eating tips and stuff like that.

P: Ok, any form of educational videos on nutrition would help users.

R: Uhm, but possibly the full membership to the app will provide you some benefits on the nutrition option I’m not fully sure yet.

P: Uhm, but here I can see the blog option so is that individual blogs or uhm organisational blogs?

R: I think it’s related to blogs of actual users so users will provide blogs about you know their experience of using the app.

P: So, then ugh an awful lot of people uhm rather than actually writing the blog would perhaps prefer a video blog as I can imagine they would like to post a video because for some disabilities typing for a blog can be extremely difficult to do for a lot of us uhm but recording a video would be good.

R: So, in terms of blogs what sort of things would you like to be covered? So like people experiences of using the app or ways of navigating around the app better? Is there anything that springs to mind?

P: It could be anything I would say anything related to exercise for the big section.

R: Ok.

P: Uhm well if you’re opening it up to anybody then you’re probably going to get any type of content but I think probably sort of encouraging uhm the physical exercise uhm the benefits physically and mentally to exercise there could be a huge host of benefits but that could go in numerous directions uhm if that’s something that is open to everybody uhm it would be interesting to see podcast and where that goes uhm it’s ugh.

R: I think from my view of it uhm having the podcast and the blogs it’s nice for new users to hop onto that and see uhm ok one of my friends has been involved in this and it’s that encouragement that people are using this app therefore I can use this app too.

P: Yep, absolutely! Blogs and podcasts are really useful.

R: I think that is a good function.

P: I think from that the blog element or even the ability to post your own exercises would be nice.

R: Ok.

P: Like if you video your own uhm it might not be part of the main structure.

R: I think that’s a good point I think recording your own exercises and your own workouts and posting it on the app and getting feedback from others would be helpful for example showing the other users the technique you’re using that is definitely a good idea uhm including vlogs uhm you know on your own progress so other people can see it providing you feedback because then you feel more confident, motivated you know have that belief that you can undertaken physical activity uhm so I think the developers should consider something like this which links into the social element of the app.

P: Yeah. I think that would be very attractive to younger users that you are targeting uhm the older people like me may struggle to do that uhm so like watching my kids now uhm the kids in wheelchairs are no different uhm so they will probably be using this side to it uhm the social side more than others.

R: Perfect, so that’s pretty much everything. I just have a few final questions and some overall thoughts.

P: Ok.

R: So, what are the potential positives if there are any of using Accessercise for improving physical activity levels? So, let’s say you have never done physical activity before, and you have come across this Accessercise app what do you think the benefits of using this app are?

P: It’s spelt out for you isn’t it so it’s great even if you are very experienced or a novice uhm the fact that there are videos there uhm there’s a summary of each individual exercise down there and you can expand it out uhm I think moving forward the app could probably have a little bit more detail on the description of each individual exercise but more than just a single uhm point there uhm there may need to be options to modify the exercise so you can adjust your arms, feet, the seat and so on uhm so you can focus on different parts of the muscle.

R: Ah ok, so like graded tasks?

P: Yeah, graded tasks could be added into the app but that’s something that the app developers are looking to do further down the line as they develop the app uhm it really depends on who you are targeting uhm if you are targeting just the social user uhm or someone that just wants to get a bit healthier than it may help.

R: Ok.

P: If you’re looking uhm at going beyond that and focusing on someone that really does have athletic aspirations you may want to add a few more bits in there uhm if that makes sense.

R: Yeah, that makes sense I agree with what you’re saying. The second question is uhm what are the potential negatives if there are any of using Accessercise for improving physical activity levels?

P: Uhm.

R: So, I guess maybe if you are motivated to do physical activity and you use the app and that social side could it be a bit of a negative?

P: Uhm, there’s always that element uhm again that group idea uhm where you can create your own sort of social peer group uhm would perhaps get around some of the barriers and negativity to physical activity uhm the only thing that instantly jumps out at me that might be a negative is ugh within the gym environment particularly when it’s busy uhm if you have got someone there typing in all the exercises they have done on the app while they are using the equipment it would irritate people waiting to use it but then you get that in every environment.

R: Yeah.

P: Uhm, and I guess you would just have to be reasonable with the amount of time it is taking you ugh twenty seconds after an exercise to type in what you have done uhm then people can wait.

R: Exactly.

P: If ugh it is taking you a lot longer than that it may be better to jump off the equipment then ugh there may need to be a note on the app uhm that says if it’s going to take you a while to record what you have done then consider jumping back into your chair or moving away and doing it before you consider jumping onto your next exercise.

R: Yeah.

P: The app may need a sub section for gym rules uhm because lots of people don’t know uhm they go into gyms and they don’t have any idea that there is gym rules and even though it’s spelt out for them there they don’t read it so maybe if there was something in the app about gym rules for example the do’s and don’t of the environment may be useful even if it’s a video or part of the blog or vlog if they had that in uhm about you know best practice in those gym environments.

R: I think that would be good to have.

P: It would be a good thing to have uhm so you know how to behave uhm.

R: Even if it links onto the function where you can see all the accessible venues for example you press on the gym where it has all the contact details, opening hours but it has a bit about the rules of gym.

P: Yeah.

R: I think that would be quite useful uhm because a lot of people will go to the gym and not read it and they will continue with what they are doing.

P: They don’t they just skip everything and carry on ugh.

R: Exactly.

P: You know that bit because the amount of people that have just no idea they completely miss what they are supposed to do when they finish with equipment uhm and it does irritate the staff uhm and if there was something there you know for example if you are using a free weight station put the weights back when you finish uhm things like that you know uhm unless the next person has asked you to leave it uhm always put them away but that would spring to mind for people that have not been in that environment.

R: Exactly I agree! Uhm, so last question is, and I know you have answered this in similar ways previously but final thoughts haha.

P: Yep.

R: How do you think the Accessercise app could be improved if anything for it’s use in improving physical activity levels? So, if you had to think of three main areas for improvement.

P: All of the things we have just discussed.

R: Yeah, but from your experience of looking through the app is there anything that stands out for you that this needs to be included.

P: The peer sub-group element uhm so your comments do get published publicly but then uhm there is the ability to communicate uhm amongst your own peer groups I think would be very useful on the app uhm the ability to see the impairment in the videos would be helpful and it would attract more people more quickly.

R: Perfectly, ok.

P: Uhm, a third one haha.

R: Haha.

P: It’s a good job that I’m struggling uhm I did have a third one in my mind but uhm yeah, I think alongside blogs is to have vlogs as an option because I think that is a way that a lot of people are now choosing to communicate.

R: So, that is a function that could be used by the developers and the users?

P: Yeah, Vlogs would really help!

R: I think if I was to use the app I would like to receive regular vlogs from the developers telling us about what is new and what has been included so instead of notifying like notifications where you have to read it and even type things in they could provide a monthly update where they say this new impairment has been included uhm we have another one hundred accessible gyms across the UK uhm press this link if you want to read more so it has that vlog version where people can come in, join in and listen to it and then you know understand more.

P: I wouldn’t disagree with that I think blogs are a great function on Accessercise.

R: I prefer watching something than reading something.

P: Again, it depends on what kind of learner you are.

R: Yeah.

P: Uhm, but yeah I’m the same uhm I will pick something up a lot quicker when I can visually see it rather than any other way but it’s not because I have an issue with reading uhm it is something that I quite happily do is reading but I would pick it up quicker visually.

R: Perfect, well thank you for that. Is there anything else that you would like to add or discuss?

P: Not that I can think of but I’m sure I have bored you silly James.

R: But, uhm no all your uhm feedback on the app and the way you have used it has been helpful because it’s always nice to help the developers improve the app further because our aim is to get more people physically active but if that is everything than that’s it.

P: Yeah, from tomorrow I will set it up and start using it uhm yeah whenever we arrange future discussions, I would be happy to feedback more.

R: Perfect, we have finished the interview.

## Transcript of interview undertaken with Participant 9

**Key code:**

R: Researcher

P9: Participant

R: Awesome, thank you Phil for ugh participating in this research study so coming here for the first phase of the trial but also coming here today to take part in the follow up phase uhm I really appreciate your time and willingness to get involved in this research and I do hope that the app can support your fitness and exercise goals ugh so I will start of the interview going through an overview of who I am and what my role is within the research project so as you know I am James Haley and my role within the research project is that I am the lead researcher so I’m responsible for conducting the study ugh collecting the data ugh analysing and also writing up the study.

P9: Sounds good.

R: Ugh, the aims, importance and purpose of undertaking this research project and the benefit of participating is that the aims are to understand how good this fitness app is at aiming various health outcomes for people living with disabilities uhm and hopefully by doing this research we can understand the strengths ugh limitations but also the areas for development that the developers could use to improve the app to be able to reach a bigger and wider population ugh the benefits of participating is that you will be able to understand more about the app but also at the end of today you will be able to go away and use the app and hopefully it will support you in becoming more physically active and reduce the risk of other health complications that you could experience uhm what University do I attend so I attend Loughborough University and my position is that I’m a Doctoral Researcher ugh the faculty that I’m involved in is the Peter Harrison Centre for Disability Sport which is based within the School of Sport, Exercise and Health Sciences ugh the other individuals that are working on the project is Sam Breary which is the director of accessercise and Ali Jawad which is the co-director of accessercise but I’m also being supervised by Doctors David Maidment and Daniel Rhind which are my supervisors ugh I will quickly go through all the ethical considerations in this study so you have read the participant information sheet so you’re aware of what the study is about and you have signed the consent forms which is great uhm any information that you provide within this study will be confidential so no one will know who you are and where you are from or anything like that because the information you give will be protected by a pseudonym and your data will be removed once the study is completed.

P9: No worries.

R: You are also provided ugh the options to withdraw from the study ugh if you feel like you don’t want to take part anymore ugh and also there are no right or wrong answers in this study ugh do you have any questions you would like to ask before we continue with the interview process?

P9: No, you pretty much covered everything haha.

R: Perfect, haha. I think I need to take a drink.

P9: Haha.

R: Uhm, so the first introductory question I have got is please may you provide the following information so your name, age, gender identity, ethnicity and county of residence.

P9: Ugh, it’s Phil Gutteridge I’m forty-five uhm I’m male and I’m from Bedfordshire.

R: Perfect, ok and your ethnicity?

P9: Oh, White British.

R: White British perfect. So, to start off the interview could you please tell me how you first got involved in the accessercise application and why?

P9: Uhm, it was through my head coach at Northampton saints rugby uhm he passed your details on and I contacted you.

R: Perfect, ok and is there any specific reasons as to why you wanted to get involved in this physical activity research study?

P9: Uhm, being recently disabled it’s kind of any ugh sort of information I can get but also with my condition uhm help others uhm anything that helps develop and improve other people living with this condition uhm that’s the main purpose.

R: Perfect, ok and have you got any experience of using any apps in the past maybe before covid or something like that?

P9: Not exercise apps no.

R: No, ok so would you say your main focus of using an app is to try and improve your physical activity levels to try and become more healthier?

P9: Yeah, absolutely!

R: Ok, great! And in terms of going to the gym uhm do you have much experience of going to the gym or doing exercise at home?

P9: I have got loads of experience of using gyms uhm before my injury but not as much after it haha.

R: Haha, ok.

P9: So haha.

R: But in terms of uhm apps and obviously going to the gym and stuff like that uhm is an app a thing that you have wanted in the past to get involved in before?

P9: It’s not something that I have really thought about to be honest.

R: Right, ok.

P9: So, uhm yeah I have never really thought about it until the opportunity came up and I thought you know what that sounds interesting.

R: Perfect, ok awesome. Uhm, so as I mentioned earlier this is a bit of a trick question but could you please tell me your experience of using the accessercise app and how long you have been a member so for example I mentioned earlier uhm have you heard about this app before uhm is it something that you have seen advertised around?

P9: No, I have not seen it before uhm until Jamie mentioned it uhm so I’m completely new to it.

R: You’re fresh into it until today haha.

P9: Haha.

R: So, this is accessercise specific questions so do you currently use accessercise in your role uhm if so when and how uhm so obviously because you can’t answer this because you have recently found out about the app but if you were to use accessercise how often do you think you would use it and when would you use it?

P9: Uhm, I would use it quite often I would think.

R: Ok.

P9: I wouldn’t say every day because you never know with every day life and kids but I would plan at least to train three times a week.

R: Ok.

P9: Uhm, maybe more I don’t know until I use it.

R: Yeah.

P9: Haha, it might be one of those ones that takes over.

R: Takes over makes sense haha uhm I know with physical activity barriers ugh some people don’t want to go to the gym because of the cost and time of travelling but do you think now with the use of an app you are more likely to exercise at home or do you think you will still maintain?

P9: I do I would still like to train in the gym when I can but having the ability to train at home and actually having the knowledge at hand and uhm with the app itself I think would be huge.

R: Ok, awesome. So, going onto what you said about obviously uhm your experience of going to the gym and so on what were your thoughts when you first started using the accessercise application so when I first showed you it a minute ago uhm was there any first impressions you got of the app?

P9: No, I thought the app is quite good I like the fact that you can see people in the videos with the same ability demonstrating the exercises ugh I love the uhm option to plan your exercises around your own abilities and disabilities uhm and also the ugh social side of it as well.

R: Ok, so you like how the app is very much focused on your disability so you can select which one you have at the start?

P9: Yeah, I like how the app adjusts everything based on the impairment you select.

R: I think what is different about this app compared to other apps uhm is that other apps focus on your fitness goals.

P9: Yeah, but accessercise focuses on individual impairments which is amazing.

R: But don’t focus specifically ok you have got a spinal cord injury ugh you have got an amputee.

P9: Exactly.

R: Everyone with a disability is unique and they are different in their own ways so recommending this amount of exercise is going to be different depending on the person.

P9: Absolutely! A strength of accessercise is that it focuses on the disabilities.

R: So, I think that is quite good uhm how about the videos you saw do you like how each exercise provides a video?

P9: Yeah, I like the demonstrations in the videos uhm they are simple obviously it explains it with the text on the screen and uhm the fact again the people doing the exercises and uhm demonstrations are disabled as well with the same mobility or like I said disability.

R: Yeah, do you like how when you select your impairment it shapes the app based around that impairment?

P9: Yes, it shows you what you can do rather than throwing things at you that you can’t do uhm which is going to be useless.

R: Yeah.

P9: It’s more focused on what you can actually achieve.

R: Yeah, because I think if you’re experiencing a disability and then you see something that is not related to you doing exercise you’re going to think I can’t do that uhm so the fact that it’s very personalised and focused on what you want to get out of it but also your disability hopefully that can encourage more people.

P9: Yeah, the videos are personalised which is really encouraging to see.

R: How about the explore section do you like what you saw with that and how there is different options on there?

P9: Yeah, definitely uhm being able especially if you’re somewhere you don’t know uhm you can then find a gym that is going to be suitable for you uhm you can have a look check it out before you turn up and realise it’s not accessible and you can’t get in there.

R: Yeah.

P9: Oh, I can’t use that uhm and having ugh reviews for people that have used it as well and you know especially because parking is such a big thing you need to be near the gym so is there any parking that sort of thing uhm is always good to know.

R: Yeah, I mean in terms of the map it’s obviously got all the different gyms but would you like to see ugh I don’t know so like local sport clubs there uhm local wheelchair basketball uhm local wheelchair rugby clubs added to that list?

P9: Yeah, I think having sporting opportunities on the explore function would be a good idea uhm I was actually thinking that before uhm so if you could obviously playing wheelchair rugby I had to look online and try and find somewhere.

R: It’s hard to find isn’t it?

P9: Yeah, it’s really hard to find something but to have something like if you do train and you are using the app and then you can say actually do you know what I fancy having a look at on the app and find a local sports club uhm they do this and that uhm you know that would be a good idea.

R: So, I find that there are clubs out there but the advertisement and the promotion of them is limited.

P9: Yeah, they are hard to find.

R: Yeah, they are really hard to find.

P9: They are really hard to find.

R: Haha, I know.

P9: Uhm.

R: So, whether ugh I don’t know people could be ambassadors for local areas and then promote and maybe add clubs to the app that would be pretty good.

P9: Yeah, adding sporting opportunities to the explore function would be amazing.

R: But I guess the app developers don’t want to move to far away from exercise you know I think they just want to focus on gym and exercise at home uhm and encourage exercise through those environments and then if they start drifting off they might then start losing focus.

P9: Yeah, because a lot of people might think uhm that’s it uhm I can’t do this and I can’t do anything anymore but if you’re actually then find the app and you’re using it and you’re exercising you then become fitter uhm you become stronger and then something you know uhm might pop up on the screen saying this is a disability sport uhm because most people do think sports is for young people but I’m forty five ugh I just started playing wheelchair rugby uhm eighty months ago and I never thought at my age I would be playing a competitive sport and it’s you know crazy.

R: Do you enjoy it?

P9: I love it uhm it’s absolutely changed my life it really has uhm so yeah it just gives you ugh.

R: Was rugby something that you have always played in the past?

P9: I have always been a rugby fan.

R: Oh, ok.

P9: I played rugby in my younger days uhm but yeah I have coached kids rugby uhm when my daughter started playing and so I have been doing that for about six years and then it was obviously once I had my injury even coaching became really difficult but I just found ways of doing it uhm and I have a lot of other coaches that support me as well so uhm doing that sort of thing but then actually getting involved in sport as well uhm yeah it’s just been brilliant.

R: I guess it opens up more opportunities.

P9: Yeah, I went to Poland to play in a European competition uhm I was like in a massive arena and I’m thinking to myself how did this happen you know haha.

R: You never thought it was going to happen in the past but then the opportunities appear.

P9: Yeah, you just take each day and every tournament uhm as it comes uhm I really do appreciate it.

R: Do you think for example once you retire from playing the game do you think you will go into coaching more or do you think you will go into like officiating?

P9: I think definitely I will stay in the sport now for as long as ugh I can uhm whether it would be uhm as a coach or just as a volunteer at the club helping fix tyres or that sort of thing uhm I will just be all around it now.

R: That’s amazing to hear!

P9: And as most people who are into wheelchair rugby they will talk about wheelchair rugby till the cows come home haha.

R: Haha, I can imagine.

P9: Yeah.

R: Do you watch quite a lot of games there as well do you watch any matches?

P9: Yeah, uhm a lot of it is on YouTube uhm there are so many games on there but also uhm we had the European championships uhm so the paralympic version which was actually in Cardiff a few weeks ago.

R: Ok.

P9: And ugh the semi finals and the finals were all televised on channel four I think it was so they streamed the whole thing on their YouTube channel for channel four and then at the weekend they showed the live semi finals and final on channel four.

R: Ok.

P9: So, yeah it’s on prime time on the weekend you know haha.

R: It’s impressive for sure.

P9: So, it’s just snowballing at the minute uhm the sport is getting bigger and bigger.

R: I know Loughborough do wheelchair basketball uhm the lighting team.

P9: Yeah.

R: They do wheelchair tennis as well uhm I’m not too sure about wheelchair rugby.

P9: Not yet we.

R: I know obviously the game is still getting developed.

P9: We have been here last year uhm we came.

R: At Loughborough?

P9: Yeah, we came here and did a taster session uhm sort of thing.

R: Ok.

P9: That was organised by Tabitha uhm she is from the University but she has been working with Northampton saints.

R: Right, ok.

P9: Uhm, Tabitha Banks?

R: No, I don’t recognise her.

P9: She is from the University but she is working at the saints but she is back here now.

R: Right, ok. So, was she there for a placement?

P9: Yes, uhm so she invited us back again uhm she wanted us to come back again this year but there wasn’t much interest uhm we couldn’t get any commitment from people to turn up.

R: Right, ok.

P9: So, we decided to put it back a bit and do a bit at a later time.

R: Yeah, that makes sense.

P9: We will be back haha.

R: I’m sure haha but on the app what I was just thinking is how on the social section where you can join groups whether they can have that function on the maps where you can find groups in terms of finding like local clubs so having the gyms but having a subcategory of that where you can find local clubs would be pretty good.

P9: Yeah, that would be good if accessercise can provide sporting opportunities.

R: Because a lot of people you know will want to use this app uhm want to do exercise uhm want to be physically active but they also want to enjoy being part of a team if that makes sense?

P9: Yes, yes. People love exercises but I think the app could benefit with sports.

R: So, whether uhm because I know from my experience of searching for clubs uhm adapted clubs and so on it’s so hard.

P9: Yeah.

R: Uhm, so whether they can incorporate that information onto the app would be great uhm because I know there are things like wheel power and stuff like that which are good but they could be doing better.

P9: Wheelpower uhm you can get a certain amount of information but you want more localised things uhm nationwide.

R: Exactly!

P9: Yeah.

R: Yeah, because one club might be far away and you wouldn’t really know that uhm so local clubs would be pretty good uhm so that’s good.

P9: Yeah, if they could provide local sport opportunities on the app that’s great.

R: Another question that I have got is do you have the required equipment to use accessercise in it’s full capacity so in other words access to a mobile phone uhm to use the application?

P9: Yeah.

R: So, if you were to use it in the gym uhm would you use your phone uhm you wouldn’t use a tablet?

P9: I would use my phone when my phone works yeah haha.

R: Haha, I believe you can ugh use the app without internet.

P9: Yeah.

R: I think you can uhm but what some people have said in the past is whether you can download some of those exercises or workouts to your phone so for example if there were some issues with internet or something like that.

P9: Yeah.

R: So, that could be a good function uhm so sort of like downloading music without internet.

P9: Yeah.

R: Uhm, so you have no issues with accessing equipment which is good so from seeing the app uhm so obviously the different sections like the social and explore do you think there are any improvements that you believe could help improve the accessercise application from what you have seen?

P9: Uhm, I do think maybe just talking about the sports and accessible sports uhm with the groups maybe there could be a group of uhm different sports kind of thing but I suppose that’s something uhm I don’t know whether you can start a group doing that uhm.

R: So, it could be something that you advise uhm or suggest to the developers?

P9: Yeah, because if you have a group where you can speak to other uhm.

R: Members?

P9: Members that play sport and you want to look into that sport but can I play that sport uhm can I do it and then you have got like a social network really uhm already with people that say yeah I do that you know it’s all abilities uhm all ages kind of thing uhm you can find clubs here and there so that might be ugh useful.

R: How about uhm like the workouts for example some people said they wanted to know whether the exercises are getting more difficult so you know uhm you have all these workouts they show you how to do the workouts but whether there could be more progressive and more difficult versions of that exercise?

P9: Yeah, I suppose uhm as you develop yourself you really want to sort of push on and think can I do something uhm can I add to this kind of thing.

R: That makes sense how about like uhm personal achievements uhm personal goals uhm at the moment that hasn’t been included do you think they can improve on that uhm make some personal goals for people?

P9: Yeah, I do I think even if it’s just ugh a gold star haha we all love a gold star so just something to say oh yeah well done you have achieved this and whether other people look at it and think oh look at them they have done really good or you can say to yourself yeah I’m smashing it this month you know.

R: But do you think the idea of leader boards uhm and competitions might be a bit too much?

P9: Yeah, I think maybe with competitions you have to be careful you don’t want it pressured on you but if you want to go for that sort of thing then if there’s an option to do that but not necessarily as ugh you’re on a leader board whether you like it or not uhm whether you’re down here uhm but your smashing your own goals but you can kind of be put off if you’re not doing that well on a leader board type thing.

R: I guess some days you may not be able to use it and some days you can and you can then easily fall down the leader board and people will think you are not doing any work.

P9: You can go you know uhm you can have uhm just a small accident and you will then be out for weeks you know.

R: Yeah, you won’t be able to use it.

P9: Yeah.

R: So, do you think like personal uhm daily, weekly, monthly goals uhm or even yearly goals could be something that could motivate you?

P9: Yeah, definitely yeah personal goals are a really good thing to have.

R: Because I’m thinking about ways that people can maintain motivation to use the app uhm they might use it for a week so sort of like new year resolutions.

P9: Yeah.

R: People use their new year resolutions for like a month and then they stop doing it.

P9: Yeah.

R: So, how can we maintain that so I think things like streaks where you are notified every day well done you have used it five days you know you get those notifications and you don’t want to lose that ugh streak because you have put so much effort into this uhm I have like one hundred days I need to keep doing this.

P9: Yeah, I think having streaks could really help me undertake regular exercise.

R: So, whether the app could include something like that would be pretty useful uhm but how about the shop and the nutrition side do you think they could improve anything on that?

P9: Yeah, definitely to have uhm especially if you’re new and you do not have the equipment at home uhm and you’re seeing people using these things uhm you might not know what they are uhm or where to get them from so having like a link to go straight into the shop and find the equipment you need uhm it makes it easier to get involved and understand.

R: Yeah, makes sense uhm how about nutrition what specifically would you want to be added to that because nutrition is complicated because you could go down the specific impairment routes and then support nutrition in terms of your impairment or keep it generic uhm it’s a difficult one.

P9: Yeah, I think with nutrition because I find that ugh a lot of people see nutrition as like oh you have to go and eat salad and they don’t understand it uhm what about education uhm you must eat this you can’t eat that you know it’s just about educating people.

R: I had one participant that was telling me he had no idea what a carbohydrate was uhm they had no idea what a protein was uhm he just realised that recently that ketchup has sugar in it and stuff like that uhm so whether they could teach you that this is a protein uhm if you want to lose weight eat this food uhm you want to gain muscle eat this food.

P9: Yeah, so any form of educational videos for nutrition would help users.

R: So, just do short videos of maybe with like the developers or even users that have some experience talking about nutrition.

P9: And you could you know uhm so like you said if you want to do this uhm this is the type of foods or recipes including these types of food and try to avoid the high sugar ugh the high salt content stuff you know uhm maybe even like an explanation on how to read like the uhm.

R: Description?

P9: The description and ugh the calorific values on the colour chart food labels.

R: Right, ok.

P9: So, stuff like that uhm because most people look at them and they are like I don’t understand that.

R: You mentioned about receipes uhm do you think it’s a good idea to maybe include receipes on there uhm or does that become a bit complicated?

P9: I think that opens a bit of a mine field.

R: Right, ok.

P9: Personally, with recipes uhm it can do but I don’t know whether.

R: I think having things like your five a day this is the recommendations for your five a day.

P9: Yeah.

R: This is uhm protein uhm this is carbs this is some vegetables to have uhm this is what they are uhm this is when you need them could be a good idea.

P9: Yeah, and if like you know if your goal is this uhm or whether it’s bulking or you know just general fitness uhm you need to be having this amount of calories uhm this much protein uhm this much carbohydrates uhm so roughly outlining these amounts rather than trying to force people that they must do this ugh they can not eat this uhm you must eat this and you must stick to it you know because I always find that as soon as you put the word diet on there it puts the pressure on you.

R: Yeah.

P9: So.

R: I guess because people are like gosh I need to do something.

P9: Yeah, they start panicking thinking I can’t do this I can do that you know.

R: I think before people start thinking about nutrition and using it they need to understand what nutrition is.

P9: Yeah, exactly! This accessercise app could help users learn about nutrition.

R: So, I think they need to like start off with a few videos like this is a carbohydrate uhm doing stuff like that because I spoke to the developers before they said the last thing they want to do is recommend meal plans.

P9: Yep, educational videos for the nutrition section would be good to include.

R: Because they are like we don’t want some users to be like this food is really good I’m going to use this every single day and it’s like they become reliant on it.

P9: Yeah.

R: So, they can recommend good food but then it’s down to you to go and get the food from the supermarket.

P9: There are so many different things uhm especially on social media where people do all of these great uhm fun things with food uhm I mean I have done loads myself and they are brilliant.

R: Yeah.

P9: Yeah, you can find those really easy online.

R: Yeah.

P9: It goes back to the education side of things uhm you need to know what you’re looking at uhm because for example a lot of people won’t know what a meal prep is so some people might not be interested in meal prep but they can cook a meal like this and I can then do it for the whole family and then that’s benefiting everybody.

R: Yeah, I always think with like the cost of living and people working long hours these days the idea of buying meals and meal planning through the app is something that they might get a bit too carried away with.

P9: Yeah.

R: So, I think they want to avoid doing stuff like that uhm how about the podcasts and blogs would you like any developments to be added there?

P9: Yeah, I think podcasts are massive at the minute uhm there’s a podcast for pretty much everything but I listen to podcasts all the time uhm and I find that especially with disability there is not really podcasts for disabilities uhm there isn’t many really out there.

R: Yeah, so you have like uhm Joe Wicks which does loads of stuff regarding exercise but whether there could be something like that uhm I don’t know.

P9: Yeah, just sort of uhm you know with disability sports or uhm just general like fitness because uhm for example I was active every day I sort of stopped with the training a bit but I was still down at the rugby club coaching uhm running around the pitch.

R: Yeah.

P9: Just being active and I went to nothing and I just ballooned in weight.

R: Were you going through a rehabilitation phase?

P9: I did I went through but I didn’t get to the spinal unit until about a year after my second surgery.

R: Which one did you go to uhm was it the stoke mandeville?

P9: Yeah, stoke mandeville.

R: Ok.

P9: Yeah, uhm and I was only there for six weeks uhm but it’s kind of it’s that you don’t think you can do anything but until you see other people which have the same disability.

R: That’s why the videos on this app are useful.

P9: Yeah, because you start thinking I can do what they are doing uhm you kind of adapt and learn uhm so it’s sort of like using a wheelchair uhm I can walk very short distance and I can stand up but the more I do it the less I’m able to do it uhm and the more I do it the less.

R: I guess you have to do it in chunks then.

P9: Yeah, so I use the chair as my tool to be able to get around.

R: When you’re at home do you normally use your chair or do you try and walk around?

P9: If I have been really bad I will use the chair.

R: Do you have adapted equipment at home to be able to support you?

P9: Yeah, we have kind of put things in strategic places haha so I can do a lot of furniture walking but we have had the bathroom changes uhm so the bathroom has been converted uhm the only thing that I haven’t got is uhm a stair lift.

R: Yeah, but there’s so expensive aren’t they?

P9: Yeah, uhm.

R: I use to work as a carer so I used to deal with them a lot of the time.

P9: Yeah, but we have had the garden slope put in so the back of the house it’s all on one level with decking.

R: Right, ok.

P9: And then there’s a ramp that goes down onto another level so I can go straight down with my chair.

R: Right, ok.

P9: So, uhm when I use the chair at home it’s mainly in the garden.

R: Right, fair enough.

P9: Rather than in the house yeah.

R: Do you have hand rails and stuff like that uhm do they help?

P9: They do but most of the time I’m on my crutches.

R: Right, ok.

P9: So, if I have got my crutches I’m normally ok but if I’m not ok with my crutches I am normally in bed or in my chair haha so I’m not really sure.

R: So, does your condition classify more as a spinal cord injury?

P9: It is now yeah.

R: Ok.

P9: Uhm, they call it cauda equina spinal cord injury.

R: Right, ok.

P9: It has now been recognised as a spinal cord injury uhm I had two hernia discs uhm L3, L4 uhm L5.

R: Ok.

P9: They then damaged my cauda equina nerve uhm I have also got a bulging disc at S1 as well so mine wasn’t permanently uhm so my spinal cord was compressed but when I had the surgery not everything came back.

R: Right.

P9: Uhm, so I have lost the sensation in my leg uhm I can still move them but I just can’t feel things uhm so they are normally numb.

R: Yeah, because CES counts as a form of spinal cord injury.

P9: Yes, and also I have got uhm spinal stenosis from right at the top to the bottom.

R: Do you get quite a lot of neuropathic pain?

P9: Yeah, and I have got compressed nerves in my neck as well now.

R: Right, ok.

P9: So, yeah it’s just one of those things uhm you get arthritis in your spine and it then just impacts everything so I rely heavily on my upper body now.

R: Ok.

P9: I try and do as much as I can but everything is more upper body.

R: Yeah, but do you find that when you do a lot more stuff you feel a lot more pain and fatigue?

P9: Oh, yeah definitely!

R: So, you feel a lot more tired?

P9: The pain is more manageable now that I am doing exercise.

R: Ok.

P9: So.

R: Do you feel like exercise actually does support your health?

P9: Yeah, it really does so the stronger I am uhm the more pressure it takes off so for me if I just do nothing it’s worse for me because the pain gets worse.

R: It’s interesting how good exercise can be.

P9: Yeah, for your mental health as well as your physical health.

R: Yeah, there’s a topic now which they are very much classifiying exercise as medicine so when you go to a GP these days they avoid giving you like antidepressants uhm but instead they are like here is a workout plan uhm maybe go and try that uhm here’s a link to a personal trainer go and do that.

P9: Yeah.

R: I know with exercise you can actually reverse diabetes.

P9: Yeah.

R: It can also support other conditions.

P9: Just being involved with wheelchair rugby it’s uhm.

R: Also, with your mental wellbeing it’s great.

P9: Oh, yeah but also meeting other people you go in there and no one sees the chair.

R: Yeah.

P9: You are actually a bit odd if you are not in a chair haha uhm they call me a part timer at my club because I can get in and out so uhm they say oh here he is the part timer.

R: It’s good that you have managed to develop and join a community.

P9: Exactly uhm so for example my friend Michael he is a farmer uhm and he had an accident at work uhm he is only thirty-five but he’s been a farmer all his life.

R: Ok.

P9: Very like old school uhm we sit and have conversations like I will go up to his house now and pop in and we will sit and have a cup of tea and we have conversations about the weirdest of things like how is your bladder uhm how’s your bowels doing and things like that and he says that it’s weird that we sit uhm we have known each other like a year and we will sit and talk about these really personal things.

R: Yeah.

P9: I wouldn’t talk to my oldest friend or anybody about this and he says that it just comes naturally because we are all going through the same thing uhm we are all going through it together.

R: Yeah, even though you are all different in your own ways you are still experiencing the same things.

P9: Yeah.

R: You know things with them.

P9: That’s it and sometimes you can say like uhm have you tried this and he is like oh I have never thought about that.

R: I do have one participant and I think she had uhm a spinal cord injury and she said whether the groups that you join on the accessercise app could be groups where you can go in there and talk to people about your impairment.

P9: Yes, community groups on the app along with a chat function would help.

R: So, like someone has had this surgery uhm what was it like uhm I had this medication uhm what was it like uhm.

P9: Definitely! Groups where you can talk to similar people would be good.

R: So, that could be good uhm those community groups so having a phone number and contact details on the app uhm people can contact you and ask you a few questions uhm they have seen your workout and they can further which I think would be pretty good.

P9: Yeah, it would be nice to talk to other group members about our disability.

R: So, those are some great recommendations so the last question I have got for this one is do you use any other similar applications outside of accessercise if so what is better or worse than accessercise?

P9: Uhm, I haven’t really used any to be honest.

R: No, you haven’t really used any.

P9: No.

R: I was just thinking a minute ago so I have seen some apps like strava and so on where you can put in your calorie intake uhm you can put in your weight uhm this current app doesn’t do that uhm so is that something you would like to see where you know you can put in your weight and see your performance change over time because one thing I have noticed about this app is that they haven’t got ugh frequency tables to show you how active you were on this day.

P9: Yeah, even if you can just regularly input like maybe once a month rather than weekly because people get obsessed by it but your weight just for example even just your weight loss uhm and when you can see it you don’t necessarily see it every day but once you see it you think oh blimey that has come down you know uhm or even like if you’re doing cardio exercise uhm excuse me uhm doing some sort of cardio exercises where you can do ten minutes and then like a month or two months later you can do twenty.

R: I think what is good with the calendar is that for the different days of the week you can see all the different workouts uhm you can see your performance uhm you can see your records and goals.

P9: Yeah, I really like the calendar function on accessercise that’s great to have.

R: But it’s not laid out in a way that is visually appealing where you can see a graph so whether a graph is something that you would like to be included where you can see you results?

P9: Yeah, providing statistics and graphs would be good l to monitor performance.

R: So, this month I have logged onto the app one hundred times and so on uhm I have used it this amount uhm so it can provide you some useful statistics.

P9: You can find uhm so this week uhm this area has come down uhm why has this bit of performance come down and then you can think oh that date uhm something happened and so on.

R: Yeah, but also seeing yourself lifting heavier weights.

P9: Yeah, I would like accessercise to provide more feedback like graphs.

R: So, like uhm May I lift thirty and then June thirty-two but you can see the progression uhm and the difficulty increasing over the last few months.

P9: Yeah, monitoring progression through graphs and statistics would be good.

R: So, that’s something that could be interesting so now it’s very much going to be about the app so if you get your phone out so we are now going to move onto the main part of this study and ask you to participate in a think aloud protocol whilst you use the accessercise application uhm the instructions for a think aloud protocol are straightforward I would like you to say out loud whatever comes into your mind there are no right or wrong answers uhm we will just run through a quick practice to get you used to the protocol so could I ask you to say out loud everything that comes to your mind when you think about physical activity so hard ugh difficult.

P9: Yep, yep.

R: Is there any other words that come to your mind when you think about physical activity?

P9: Uhm.

R: Enjoyable maybe?

P9: Enjoyable yeah enjoyable uhm enjoyment ugh.

R: Painful sometimes?

P9: Yeah, that’s a big one haha.

R: Haha, painful?

P9: Yeah, if things are painful uhm or whether uhm you’re not enjoying something.

R: Would you say you have more of a positive view on physical activity or a bit more of a negative?

P9: Oh, positive!

R: Yeah.

P9: Yeah, definitely!

R: That’s good! That’s a good start. So, what I would like you to do is share your screen with me so we are going to go through each bit of the app step by step and you’re going to tell me what you think so this here is the social section so what are your first thoughts when you see this section uhm is there too much information ugh is the colour ok?

P9: No, it seems pretty clear uhm obviously with the people you are following or are following you and being able to see their progress uhm so if you’re following someone it’s probably going to be because they are the same ugh ability or disability as you.

R: Yeah.

P9: And to see their goals and their exercises uhm and their progression is great.

R: So, with the actual photos you see here it’s not actually Liv doing it uhm it’s the videos of those workouts and exercises that she has done so people don’t need to do videos of themselves doing it uhm it’s very much they are doing the workout and then they post it.

P9: Yeah, the videos are helpful to show how to do the exercise!

R: But do you think the colours are ok uhm so like the white?

P9: Yeah, the white background makes it stick out clearer.

R: It’s not too much for you?

P9: It’s not too much uhm because sometimes you get apps that are a bit of an overload with colour uhm especially when you’re old like me uhm so yeah it’s quite clear and the boxes are quite large with the actual name of the uhm exercise on there as well.

R: Yeah, so that’s one point that someone mentioned is that uhm at the moment the app hasn’t got an option to increase text size uhm do you think that is something that the app could benefit from where you have the option to increase the size and photos on the app?

P9: Text probably uhm I mean it’s fine for me but you might have someone which may struggle.

R: But I guess if you’re somebody that is a lot older and you don’t have a good sight.

P9: Yeah, having the option to change fonts, colours, text size would be good.

R: Uhm, that could be an option.

P9: It could take you a while to sort of read something.

R: I think if you use the app and you don’t get the initial like engagement with it uhm it may put you off so if you have got the option to zoom in and read stuff you may be like oh this is actually pretty good.

P9: Yeah, being able to increase text size and change fonts would be good.

R: But do you like at the bottom where it’s got the love hearts and comment box uhm do you think they could add anything there?

P9: Uhm.

R: So, you can actually react to her workouts here?

P9: Yeah, reacting and commenting to friends workout is good!

R: Do you think uhm liking and commenting is enough?

P9: Yeah, uhm the only other one is the option to be able to share it.

R: Going onto that point you just mentioned uhm a downfall of the app is that you can’t actually export the results from this app onto things like Facebook.

P9: Yeah, it’s a shame you can’t export your results out of the accessercise app.

R: So, whether they could incorporate that would be a great idea uhm because I know that a lot of people use Facebook uhm they may want to share their progress with their other friends on Facebook and they can say hey guys this is what I have done and they can then like that post on Facebook because uhm at the moment only people on the app can see it uhm so whether there is a reason for that uhm and maybe they just want to keep it on the app.

P9: Yeah, I mean you could have like uhm if you share it via Whatsapp that kind of thing so somebody personally and then it gives them a link to it but then it takes you to the app.

R: They do actually have that function.

P9: Oh, do they?

R: Yeah, so you can invite friends from your contact list but you just can’t share your results with contacts on WhatsApp and Facebook.

P9: Right.

R: So, that could be an option that they include uhm so it’s very basic uhm it’s not overwhelming and stuff like that but if you were to press on this photo here do you like the profile ugh section do you think it’s not too much information uhm it’s clear.

P9: Yeah, it’s yeah because yeah it’s obviously gender, age, uhm.

R: Would you like there to be anymore information included there uhm because some people could add fitness goals uhm they could add their city add their phone number.

P9: Yeah, uhm whether you could put what your disability is would be good so if you have got cerebral palsy or multiple sclerosis or anything like that.

R: Ok.

P9: And then you have got something in common with that person so you may be more likely to follow them.

R: Ok, uhm would you like the option to be able to actually contact the person so at the moment you know you can react uhm you can comment on their post but would you like the option to message or do you think that’s a bit too invasive maybe?

P9: Ugh.

R: Maybe it goes against privacy uhm I don’t know.

P9: Messaging uhm I suppose like Facebook you can accept or decline messages uhm so maybe you can add or remove that function within your own settings uhm.

R: Uhm, so having the option to message people is down to them whether they accept or not?

P9: Yeah.

R: Ok, that could be a good option.

P9: Yeah.

R: Because I could follow Liv uhm really like her workouts uhm like how she’s doing it but then I don’t have an option to message her.

P9: Yeah, if you could message others users that would be good to include.

R: So, if I want to learn more I can’t because I can’t message her so whether they have that function could be something good uhm do you like how the achievements and followers stand out there?

P9: Yeah, uhm obviously it’s still coming soon which is a weakness of the app.

R: But with achievements uhm you obviously said about personal goals and so on.

P9: Yeah, having personal goals and awards would help me become motivated.

R: That could be a good function to have uhm.

P9: Yeah, so when I started I was here and now I am here.

R: Right, ok.

P9: This is what is has done for me uhm that kind of thing.

R: That makes sense yeah uhm so now if we were to go back off the profile uhm and we go to the groups and find groups.

P9: Ok.

R: So, obviously you have all these groups uhm do you like the function here how you can join groups?

P9: Yeah, the group option on the app is great to network with other users.

R: So, if you were to press one uhm so press the group I’m new to this.

P9: I’m new to this.

R: Perfect.

P9: Typical, no internet connection haha.

R: Do you like the fact that it shows you that nighty six people are in this group?

P9: Yeah, nighty six.

R: Uhm, so obviously it has got the description uhm so if you press show more uhm do you like how they have laid this out or do you think they could include less information maybe uhm like bullet points I don’t know.

P9: No, I think that is pretty uhm it’s not too long it’s not too short.

R: How about the image that they have used at the top uhm it doesn’t really relate to the group uhm do you think maybe uhm a user or someone doing something related to the group could be added there?

P9: Uhm, yeah possible to be honest I didn’t really think about it when I looked at it haha.

R: It would be good to have something on there where you can see the person could be quite interesting uhm but obviously you have got descriptions which is quite good uhm you have leave group uhm you have also got the posts.

P9: Yes, the exercise description is good but could be numbered or bullet pointed.

R: Which is quite good so you like that function?

P9: Yeah, but the description of each exercise is one big block of text hard to read.

R: How do you think that function could be developed uhm do you think the opportunity to actually design your own groups could be something that could be added?

P9: Yeah, definitely designing new groups if you’re like I said before uhm starting to take part in a sport that you could you know because knowing how sports has helped me uhm I never thought I would be doing.

R: Ok.

P9: And there might be other people out there that you know wouldn’t realise until they actually come across that and think oh gosh maybe that is something that I could do.

R: Yeah, that makes sense ugh I think at the moment that is all they have in terms of the groups uhm but if we were to go back uhm do you like the fact that there is a notification bell at the top so you can be notified uhm so that could be something like uhm today is your strength workout don’t forget to do it or something like that.

P9: Yeah, notifications or nudges would really help me undertake more exercise.

R: Do you think maybe like a nudge uhm or a vibration on your phone could help or just a notification?

P9: Uhm, yeah probably because yeah so when you get the notification you get a little nudge uhm but obviously then people turn them off don’t they uhm or what ever they want to do with them.

R: Yeah, some people may get annoyed.

P9: Exactly.

R: So, that’s that section so if we go down to exercise uhm my advice is that if you ever get stuck with trying to find the correct exercises uhm just use the filter option at the top right because some exercises you may think are named this but because this is an adapted app it won’t always have the exercise you’re looking for under the name you think.

P9: Yeah, the filter option on the workouts is a really good feature they included.

R: Uhm, they are all there but they are under certain names but do you like how this is laid out here so at the top is your goal uhm your location and so on?

P9: Yeah, I think that is pretty good because you have got the location uhm so I’m at home so you will click home uhm and then the goal and then the exercises uhm the muscle groups and so on.

R: Do you like all that stuff?

P9: Yeah, I think the filtering option and designing your own workouts is pretty good uhm it flows pretty well.

R: What some people have said in the past is things like uhm be healthy and be fit uhm and also cardio options for the goal could all fit under one.

P9: Yeah, some of the goals on the workouts seem the same and could be reduced.

R: So, whether that could be reduced so maybe there are less options but be fit uhm be healthy.

P9: Yeah, because fit and healthy uhm and also cardio all seem to come under the same umbrella.

R: Yeah, so that’s maybe something the app developers could work on uhm but there’s obviously a reason why they are there.

P9: Yeah.

R: We don’t know about it but whether they could categorise them within one uhm so be healthy ugh fit and do cardio uhm because mobility and flexibility I can understand because if you’re like eighty uhm mobility might just be walking around the house uhm and that’s your goal of the day uhm yoga obviously uhm that’s understandable uhm using a yoga mat that you will have at home uhm flexibility and stretching and so on uhm so stretching you could do before you do gym work and you then go onto your next workout at the gym.

P9: Yes, categorising some of the goals together because they seem the same.

R: So, they are all relatable and it’s obviously ugh you have got the muscle groups.

P9: Yeah, the muscle group photo of the muscles you’re working is good.

R: What I think would be good is that if you were to select home uhm and then you were to select flexibility uhm all the other options go black or dark.

P9: Yes, so it just highlights the options that you can do.

R: Because you have got home equipment uhm gym equipment if I was to press flexibility I don’t need a dumbbell.

P9: No, some of the equipment that appears here is not related to my goal.

R: I don’t need you know uhm all this equipment so whether it could just go straight into it uhm so reducing the information could be pretty good but do you like the little symbols and logos that they have used?

P9: Yes, I do yep having logos for exercises is creative and visually appealing.

R: I think that is adds a bit more creativity which is good.

P9: I agree, the little logo they have used next to each goal is creative and helpful.

R: If you were to scroll down quickly uhm do you like how for example they have got so if you scroll up a little bit they have four four two uhm because I’m thinking whether these could be put in alphabetical order or something like that because some people have said like I’m dyslexic I have four four two uhm why can’t it be three three three or something like that.

P9: Yeah, uhm I would have never even thought about that but I would have just looked at that and saw like muscle groups uhm and the rest of the content all there.

R: Yeah.

P9: And I would have picked my preference out of what is there uhm I wouldn’t have even thought about how it was listed.

R: That makes sense uhm some people have said in the past uhm so difficulty uhm are these options ticked or are these ones ticked uhm so there’s a bit of confusion about which ones are ticked because if you press liked and then it goes off uhm so whether they could make it stand out on whether that is the one you have pressed.

P9: So, these are the ones that are highlighted.

R: Yeah.

P9: Ok.

R: Sometimes when you design a workout some are already clicked.

P9: Yeah, so then maybe you choose your difficulty.

R: Yeah.

P9: Uhm, so if you were doing yeah.

R: It makes sense uhm so you have got exercise uhm requiring an assistance or carer ugh what do you think could be developed for that uhm if you were going to a gym what sort of things would you want to be added to that?

P9: Uhm, I don’t know really uhm I suppose if it’s linked to let’s say the gym itself so who ever is on duty or working in the gym they will get a buzz that says someone is disabled is coming in uhm so whether there is a registration thing where you can say I’m in you know so sort of linking it in so the staff at the gym know you’re in there.

R: Yeah.

P9: And then if that comes up they know then know what it is.

R: I think the app could work more with the gyms so whether the link to the gym website could be on the maps uhm so whether the gym could be notified whether someone with a disability is coming to the gym.

P: Yeah, if someone at the gym is notified that you need help would be good.

R: They don’t need to know how to adapt the equipment for the person uhm the person should have a basic knowledge of what they are doing at the gym uhm so for example if I can’t lift a bit of the equipment above me then I just tick the notification and someone comes over uhm they then pick it up for me and then they go.

P9: Yeah, if someone in the gym is notified and can help me that would be good.

R: So, that could be an option because that’s what I’m expecting just a spotter.

P9: Yeah.

R: So, if you were to press out door training for example uhm so going back to what I said before about the four four two can you see here it’s two two two and then some three three and some two but do you think these could be alphabetical order for example because when I look at it uhm I’m looking for a certain piece of equipment it could be easier to find it if it was alphabetical.

P9: Yeah, I suppose that alphabetical order could help uhm yeah but if I’m looking at that and I’m thinking outdoor training.

R: Do you think they could categorise it in like letters so like a and b.

P9: Uhm.

R: Actually, that might make it a bit more complicated doesn’t it haha.

P9: It does yeah I think to me I can just look at them uhm I think all the information is there if you have it listed you have to start scrolling uhm where on here everything is on one page uhm and I can just read it and think ok I want that one uhm I want that one or you know.

R: Right, ok makes sense.

P9: So.

R: But you don’t think there’s too much information there in terms of equipment?

P9: No, I think in terms of information it’s ok because you want you know yeah I mean there is a lot there which is stuff that you will have at home like you could have dumbbells and kettlebells but more likely to have things like uhm a foam roller ugh a resistance bands.

R: Yeah, exactly.

P9: Uhm, I’m not going to use a skipping rope but uhm but yeah just those sort of things.

R: Yeah, makes sense.

P9: I mean out of all of those I have got uhm.

R: So, I guess depending on your impairment.

P9: Yeah, linking suitable equipment to your impairment would help.

R: Depending on what your goal is uhm depending on where you want to do it can really reduce uhm because that’s what I was saying about reducing content uhm because if you have a spinal cord injury you’re likely going to be in a wheelchair you’re not going to use certain equipment.

P9: Yeah, reducing information because you’re not going to use a hula hoop or ugh a skipping rope for their intended purposes anyway uhm.

R: So, whether information could actually be cut out based on what you have selected.

P9: Yeah, based on what you have selected certain information is removed.

R: That could be a good option uhm ok so perfect ugh you have all those options so we will now go off this section uhm so if you were to press on an exercise.

P9: Ok.

R: So, just going back to ugh what we mentioned when we first started the interview so the information down here do you think this could be laid out better uhm what comes to your mind when you look at that?

P9: Uhm.

R: Maybe bullet points or something?

P9: Yeah, I was going to say bullet points uhm would help present the app better.

R: Because if I read that I’m like gosh that is so much.

P9: You just have to sit and read it haha so the blocked text is a weakness.

R: Yeah.

P9: Bullet points for what the exercise is uhm what it does and then instructions on how to do it would help me a lot.

R: I also think what would be good is that at the end of each exercise it has got the benefits uhm it has got useful tips uhm it has got the picture of all the muscle components being worked ugh it’s got useful tip but whether there could be a bit about progression so this is the generic exercise uhm to make it harder pull the bands further to the right or something like that.

P9: Yeah, including a progressive exercise would be useful to have.

R: So, if you were to do these exercises over and over again uhm you are going to like them but you want it to be a bit more difficult over time.

P9: Yeah, you need more challenging versions of each exercise.

R: So, whether they can have uhm to make it harder do this.

P9: If you haven’t got thicker bands take some of the slack out so you have got more excess outside of your range compared to inside.

R: Yeah, so whether they could include that for every exercise uhm to make it harder do this because then it challenges people.

P9: Yeah, progressive exercises would help me.

R: Uhm, but you like the videos don’t you?

P9: Yeah, uhm. The videos on the app is really useful and helpful.

R: How about uhm so I mentioned about voice overs uhm so do you think anything could be added to the videos?

P9: Again, like you say about uhm increase difficulty by moving further away or thicker bands or you know.

R: Do you think maybe in the videos uhm at the end they could say to develop this exercise further do this?

P9: Yes, at the end of each video they could show you the progression.

R: Or add a useful tip at the end uhm or a useful box that says progression this so people see it and they are like oh ok I have done that uhm I want to make it harder.

P9: Yeah, so like this is too easy now you know uhm have something that shows the progression.

R: It’s got all the exercises you need but whether they could push people a bit further.

P9: Uhm progression uhm advice I suppose.

R: Yeah, progression advice uhm because they have got the useful tip uhm like inhale and exhale which is great but could they do a bit that says progression.

P9: Yeah, some videos don’t have voiceovers they need them on all videos.

R: Did you notice how the first video I showed you had no voice over?

P9: Yeah, voice overs are limited on accessercise they should have more.

R: So, whether they could maintain consistency on that?

P9: Yeah, maintain consistency on the voice overs for all the included videos.

R: Uhm, but you liked the favourite option uhm so the little tag?

P9: Yeah, the little tag for favourite is good ugh that’s easy.

R: Yeah.

P9: And then again it’s got the little icon there uhm I don’t know whether that does anything.

R: I don’t know what that means but that’s another point is could they provide a keys for what this information means?

P9: Maybe one when you need assistance uhm some you don’t need assistant.

R: Ok.

P9: Yeah.

R: So, if we were to back uhm and go onto the last one which is explore uhm so sometimes it randomly puts you in Africa so you might just have to zoom out and go to London.

P9: Oh, I’m in the ocean haha.

R: Haha, it does that depending on the phone.

P9: Yeah.

R: But what I mentioned about the key is that you saw that little symbol earlier uhm I was thinking what does that mean uhm because it’s confusing on what the difference is between this one and if we were to scroll down again uhm maybe zoom out again haha uhm so maybe like a key to describe what these mean uhm so if you were to press on one uhm so as you can see this gym hasn’t been rated yet.

P9: Yeah, on the map and explore function could benefit with a key function.

R: But is there any recommendations for the explore section uhm I think you about the videos and the images?

P9: Yeah, images of the gym would be good so you can see the gym before you come uhm also parking information uhm accessible information uhm so is there a lift that kind of thing.

R: Ok.

P9: Uhm, if there is anything split over the two levels.

R: Perfect, ok.

P9: Obviously you have some gyms that have cardio upstairs and weights down and vice versa.

R: So, can you see how this logo is different to the other ones?

P9: Yes!

R: For me I still don’t know what that means so whether they could provide a key uhm actually I think this means that it has been ugh rated as accessible uhm this one has start whereas the other ones don’t.

P9: So, are these just gyms?

R: Yeah, these are gyms but they haven’t been rated so there are no stars there so as you can see this information here is so limited.

P9: Yeah, accessercise is limited on reviews and should be sharing users reviews.

R: So, you would like a link uhm you would like some videos uhm parking?

P9: Yeah, parking uhm accessibility just the normal information ugh so everything you need as uhm someone with a disability whether you’re in a wheelchair or on crutches or using a frame or you know.

R: Perfect, ok so let’s go onto the final little bit so ugh the more section down here we will just talk through this so the shop you mentioned about obviously providing a link to equipment that has been used?

P9: Yes, if the shop can link websites for equipment that would be great.

R: Uhm, what about providing people with discounts if they work out more?

P9: Yeah, maybe like incentives could help so uhm the more you use the app and the more you achieve you know you can get a discount in the shop or special offers or you know if there’s one month there’s a special offer you get a discount on resistance bands if you hit a certain target or something like that I don’t know.

R: Ok, and nutrition obviously you said about educational videos and other useful resources which is great uhm blogs and podcasts you liked.

P9: Yes, nutrition, blogs, shop and podcast are all amazing features on the app.

R: Uhm, and account settings so the impairment filters you said you liked how specific the app is with regards to the disability?

P9: Yes, a benefit of accessercise is the impairment filter.

R: That’s great uhm so exercise habits so how often you exercise.

P9: Yep, but when I complete the exercise habits it doesn’t do anything.

R: Measurement units you like?

P9: Yeah, measurement units is good but they could add more to it.

R: Manage subscription and so on.

P9: Yeah, manage subscription is a good function that the app has.

R: So, that’s the whole of the accessercise app.

P9: Yeah.

R: Which is great ugh so we will now go onto the overall thoughts so these are the last questions uhm so the first one I have got is what are the potential positives if there are any of using accessercise for improving physical activity levels uhm do you think in terms of ugh tackling barriers uhm saving time uhm saving money?

P9: Yeah, I think it just cuts out a lot of hassle of trying to find things that are suitable you know uhm to have it all in one places rather than scrolling through and thinking no I can’t do that uhm I can’t do that and so on.

R: Yeah, and it’s also filtered based on your impairment.

P9: It is so I can just go through the filter and then just say oh right I can do this uhm and within a few minutes you’re good to go whereas oppose to sitting there you know scrolling through lots of information that does not really have any relevance to you.

R: I agree. Uhm, I had one participant he weighed not much at all and his height was really small so like two foot or something and he said that when he used previous apps uhm he put all his information about his height and weight and it said this is incorrect uhm so it didn’t really filter correctly based on what he was inputting but the accessercise app it’s not about your height and weight uhm they don’t really care about that uhm they care about your specific disability.

P9: Yeah, accessercise is great because it focuses specifically on your disability.

R: Uhm, so when you select that the exercises are based on what you can do.

P9: Yeah, so they know that if you put in a certain condition they are going to know that you are not tall and you’re not heavy haha.

R: Haha, exactly so it filters everything around which is good uhm I think what is also good is the cost of the membership uhm which is not too bad I think it’s about fifty pound a year but if you compare that to how much a gym is a year it saves so much.

P9: That’s how much my gym is a month haha so accessercise is very cheap.

R: Haha, yeah! So, it saves a lot of money ugh it saves the cost of travelling uhm the petrol but also the time to go to the gym uhm because it’s an app you can just do it at home uhm but also at the park.

P9: Yeah, accessercise is cost effective as you can do it at home and the park.

R: Uhm, what are the potential negatives if there are any of using accessercise for improving physical activity levels?

P9: Uhm.

R: Do you think people may become a bit unsociable uhm maybe not leave their house to exercise and meet people out and about uhm but you have got the group section?

P9: Yeah, I think the opposite actually I think it will encourage people I can’t think of any negatives about it really uhm if it improves your physical and mental wellbeing it’s going to encourage you to go out uhm and do more and it’s going to help you to get to that point where you can you know go out and be more independent.

R: Yeah.

P9: A lot of people uhm that will change uhm because they are not independent uhm but you know maybe it can be.

R: Yeah, it could try to encourage some behaviour change.

P9: Yeah

R: So, that’s pretty cool. Uhm, the last question I have got is if you had to sum up in three points how do you think the accessercise app could be improved if anything for it’s use in improving physical activity levels?

P9: Uhm.

R: If you had to name three or even four haha.

P9: Uhm.

R: So, if you think about each component on the app uhm so you have got the social section.

P9: Uhm, the social section maybe a little bit of improvement with like I said uhm with connectivity to sporting opportunities not just gym and exercise it could actually go onto sport as well.

R: So, like local opportunities?

P9: Yeah, if they could include local sports on the map would be awesome!

R: Ok.

P9: Yeah, sort that sort of thing.

R: Ok.

P9: Yeah, haha because not everyone likes gyms sometimes they want sport.

R: And you have got the exercise uhm workout section.

P9: The designing your own workouts is excellent uhm because it’s based on your capability and disability uhm that’s perfect uhm that’s probably one of the biggest things I think.

R: It’s specific to your own impairment.

P9: Yeah, it’s specific to yeah so accessercise focus on your disability well.

R: But do you think anything could be developed on that section?

P9: Uhm.

R: So, you have got the home, gym ugh outside but maybe narrowing down and reducing some of that information?

P9: Uhm, I think they are the fundamental three things aren’t they uhm you’re going to be at home uhm you’re going to be in a public space or within a gym.

R: Yeah.

P9: So, I’m not really sure if there is anything that you can add to that.

R: No, ok.

P9: Uhm, and just having those four options at the bottom makes it a bit easier than having more information.

R: Yeah, that makes sense and the last bit uhm is the explore section with the maps?

P9: Yeah, so maybe including like a key of what is what and what means what so having certain added features like parking availability and what facilities the gym has for people so like uhm changing facilities ugh toilets uhm.

R: So, like videos and images of the gym before you visit?

P9: Yeah, even if it’s like little bullet points of what it has got so uhm it has got accessible toilets uhm it has got accessible showers uhm it has got a lift ugh so show the users what they have at that gym you know.

R: Ok, perfect and the last question I have got is uhm is there anything else that you would like to add or discuss about the app before we finish.

P9: I think we have pretty much covered everything haha.

R: Perfect, thank you Phil I do appreciate your time and willingness to get involved.

P9: No worries.

R: Thank you so much.

P9: Thank you.

## Transcript of interview undertaken with Participant 10

**Key code:**

R: Researcher

P10: Participant

R: Perfect, thank you Thomas so yeah thank you for participating in my study uhm what I will do now is I will give you an overview of the interview, who I am uhm you know what my role is within the research project, the aims uhm importance and the reason for why this research is being undertaken so first of all I am James Haley and my role within the research is that I’m the lead researcher uhm the aims, importance and purpose of undertaking this research project and the benefits of participating is that we know that during COVID-19 people with physical disabilities uhm didn’t undertake much physical activity because of the pandemic uhm lock down and so on so therefore their physical activity levels dropped and we know with the advancement and development of technology we feel like smartphone apps and fitness apps used through phones may be a good intervention to improve physical activity in this population uhm and hopefully with the development and evaluation of this app it can reach more people to be able to improve their physical activity as well as a range of different outcomes like quality of life uhm psychosocial wellbeing and some physiological measures uhm so the University that I attend is Loughborough uhm I am currently based within the Peter Harrison Centre for Disability Sport within the School of Sport, Exercise and Health Sciences and I am being supervised by Doctors David Maidment and Daniel Rhind and we are also collaborating with the app developers uhm which is Sam Breary and Ali Jawad uhm so you have read through the information sheet earlier for both studies and you have also signed the consent forms which is great so you understand about what the ugh studies entail and what we will be doing today uhm any confidential information that you mention for example your name, your age, your gender and where you live and so on will be confidential and they will be removed from the study or hidden with black boxes when I transcribe the interview uhm you will also receive a pseudonyms so your name will be replaced with a fake name so no one will know who your real name is and any other information you mention won’t be uhm linked back to you.

P: Ok.

R: If at any time you want to withdraw from the study and you don’t want to do the study anymore, please feel free to tell me and we will stop the interview there uhm and we will remove your data that’s not a problem but please feel free to let me know if you would like to do that uhm also within this study there are no right or wrong answers and do you have any questions that you would like to ask before we continue with the interview process.

P: No, I’m happy to go ahead.

R: You’re happy to proceed.

P: Yeah.

R: Perfect! So, the first section for the interview is very much introductory questions so please may you provide the following information for example your name, age, gender identity, ethnicity, and county of residence?

P: My name is Thomas Mills I am thirty-one years old uhm I’m a male uhm white British and I live in greater Manchester.

R: Perfect, that is a very good memory haha I don’t think I could remember all of that.

P: Haha.

R: Uhm, so to start could you tell me how you first got involved in the Accessercise application and why?

P: Uhm, so I work for an NDSO which is called dwarf sport association and I was made aware of the app and it is always something that I’m looking to do is to get fitter and healthier and the opportunity to explore an app specifically designed for individuals with a disability was one not to be missed from my side of things.

R: Perfect, ok and during COVID-19 did you experience any difficulties with physical activity and exercise did you experience any more barriers that you wouldn’t normally experience?

P: Yeah, and I think uhm personally over COVID-19 my exercise levels dropped considerably and the motivation to get up and go and do something was difficult based on the situation that we all found ourselves in and it has been hard to get that mojo and motivation back following the pandemic and even though the kind of world has opened up and is normal again and we are able to get back to physical activity it’s hard to get that drive that I used to have.

R: Perfect, ok and have you used uhm any fitness apps in the past that may have helped you improve physical activity?

P: Uhm, I have recently started uhm using an app uhm and I am only in the very early stages of it and that’s called body coach which provides uhm weekly workouts for uhm ultimately beginner stages at the moment which is going ok I’m enjoying it at the moment but I’m also looking to explore the Accessercise app as well.

R: Perfect, ok so going back to uhm what you mentioned about uhm you know about Accessercise app uhm was that advertised as a part of the work that you do now or was it something that you have just seen online?

P: Yeah, so uhm I actually think the developers Ali Jawad uhm initially got in touched with the Dwarf Sport Association themselves so uhm I had heard about it through there and he let us know when dwarfism was being launched into the app uhm and this is obviously a great opportunity for me to explore the app.

R: Perfect, ok and in terms of apps uhm because you said you have some experience of uhm using apps in the past uhm do you think it is something that you will find quite easy to use uhm do you think it is something that you will use regularly as a result?

P: Yeah, uhm just due to my current life situation and with children uhm the time to get up and go to the gym at the moment is non-existent with two younger children so finding something that fits around my daily life and family commitments that I can fit in thirty minutes either in the morning or evening or on my lunch hour uhm because I do work from home uhm that would be beneficial to me so an app is something that I would be looking to take part in more physical activity and exercise during the day.

R: Perfect, that really does sound great! Uhm, so could you please tell me your experience of using the Accessercise app and how long you have been a member?

P: Uhm, so I have only recently uhm signed up and logged over the past week I have had a little look around the app uhm so yeah within the past week.

R: Perfect, ok awesome so now this is Accessercise specific questions so do you currently use Accessercise in your role and if so when and how?

P: Up to now I haven’t been using the Accessercise app but it is something that I’m going to look into uhm and seeing whether or not it’s something that works for me and my lifestyle and whether or not I find the app beneficial both physically and mentally because I think exercise for me personally is not all to do with physical goals it’s also to do with making me feel good and uhm being in the best frame of mind and I find exercising does that uhm the endorphins it releases help me and benefit me being a better person uhm yeah.

R: Perfect, ok and if you were to use the Accessercise app uhm what sort of goal would you go for uhm would you go for like building strength uhm would you go for uhm home based exercises, gym based exercises?

P: I think uhm at this stage because I’m new to my kind of fitness journey it would be to lose a bit of weight and to tone up uhm depending on how my personal journey went uhm yeah it would be good to build muscle and be stronger but uhm I think initially it’s mainly to do with the kind of health benefits and yeah to tone up and keep me as fit and healthy as possible.

R: Awesome, sounds great. Uhm, so what were your thoughts when you first started using the Accessercise application this week uhm so what were your first impressions uhm did you like the functionality of the app uhm did you like what was included?

P: Yeah, I thought it was great uhm that there are different impairments and it’s impairment specific so the exercises that you are performing are performed by somebody with your disability so you see somebody like yourself uhm and I think representation is key for me uhm and it’s great to see somebody on there with the same disability as myself uhm I forgot your question.

R: Uhm, what were your thoughts when you first started using the Accessercise application so for example did anything stand out so you have mentioned on how the app is very much focused on the impairment and it’s filtered based on that but is there anything that you thought wow this is really useful uhm this is going to help me on my own journey in physical activity?

P: I think in terms of like the muscle groups so some of the different areas where you can target different muscle groups that you want to work on uhm and specifically when you can kind of put your goal and it kind of tailors it so the tailored element I think is really useful so if you’re not into your sport and fitness and you have a specific goal in mind it does allow you to do that so you don’t have to anything because they have put the ground work in for you and the questions are very easy to answer.

R: Right, ok so you mentioned about how you like how the app is tailored based on the impairment uhm but do you think there is anything else that they could include to maybe make that bit more tailored towards the person?

P: Uhm, that’s a good question.

R: So, for example the app has got the videos of someone with like uhm a spinal cord injury uhm and you have the options to choose your impairment but is there anything in particular that you would like to be included?

P: I think specifically for dwarfism it would be nice to have a male and a female so obviously I’m male uhm perhaps that could be something that is looked into developing it further uhm I can’t think of anything else.

R: So, was that one of the functions that you like compared to other apps for example uhm previous apps doesn’t really focus on the impairments uhm but they focus on general exercises.

P: Yeah, definitely I think it pushes you that extra harder uhm for example if there is an exercise on there that you are struggling to do or you can’t do there is somebody there with the same disability as you who is doing it so you can work towards that through different means so you know it is achievable because somebody similar to you is doing it uhm you might not be at that fitness level or fitness stage of your life then but with the right training you can get there and work on that muscle group to perform that press up or uhm full lunge uhm you might not be there yet but you can because you have seen someone life yourself doing it so the motivation is there and that you can achieve it.

R: I really like that function uhm you know how it’s got someone there with the impairment that you have selected.

P: Yeah, having someone with you in the videos is amazing!

R: You very much feel like you know they are relatable to you.

P: Definitely! I think feeling related to someone in the video is good.

R: The fact that you see similar people to you doing the exercises you then feel like you can do it whereas if it’s someone that is more abled and does not have an impairment you feel like well ok they can do this I can’t do this so the fact that you can select your impairment uhm you can select the exercises you want to do but it also has videos of someone with a similar impairment doing that I think is really important.

P: Yeah, having videos of someone with the same disability is good.

R: Like you said for motivation and so on uhm so for example when you first start using the app you saw uhm the exercises uhm you saw the explore section uhm and more options uhm but do you like the fact that the app has that explore section do you like uhm the fact that you can search for other local facilities uhm do you like how it has got that accessibility section?

P: Yeah, so in terms of the explore section I’m not sure if it would be a section that I personally would look into using just because uhm like I mentioned in terms of going out and going to a gym uhm at the moment with having ugh a two year old and an eight year old it’s very difficult for me to find the time but I do see the benefits of it and I can see the wider community finding it useful if they wanted to find a gym environment that they wanted to know whether it’s accessible or not then yeah I think it is good but I think it could be uhm developed a bit further in terms of what makes it accessible because for example for someone with dwarfism the accessibility of something may be different to somebody with another disability or impairment so there are key things for people with dwarfism that they might look into so obviously some of the machines that people use with dwarfism could be difficult to reach down or whatever it might be I think uhm a chat function there maybe or an opportunity to ask questions further and get them answered within a week or whatever because yeah it may be accessible for a wheelchair user but that accessibility element might not be the same for somebody with dwarfism so maybe a criteria that is set for uhm what does accessibility mean within that gym are they talking about accessibility in terms of transport uhm and getting there or is it talking about accessibility in terms of getting into the building or is it talking about accessibility in terms of completing your workout almost?

R: So, really distinguishing what it means when they say accessibility.

P: Yeah, so I think traditionally accessibility for people is getting to and in the location and whether it has the right facilities there but I think it is much more than that on a wider scale.

R: That makes sense yeah I feel like on the uhm explore section you have got the gym uhm you have got the phone numbers uhm email addresses but it is quite limited on what makes that specific gym accessible.

P: Yeah, they need to include more accessibility content on the map feature.

R: So, I think if they have some sort of you know like forum or q and a that says this gym is accessible for these reasons then users will read that and think ok I understand why this gym has for or five stars for accessibility.

P: Yeah, including a Q and A for accessibility for each gym would be nice.

R: Because you see that the gym has five stars but what specifically makes it five stars.

P: Yeah, or even the opportunity to almost like write a review on the gym uhm I don’t know if each review was a similar review but people with different impairments could do it themselves and review it so you can see like ok someone with my impairment has gone to that gym uhm they have found it accessible so therefore I should find it as well there shouldn’t be any reason for any differences in finding it accessible.

R: I think that is quite easily done isn’t it as well because the explore section on the app is linked through Google Maps so with the google reviews it could easily be added onto the app to be able to provide that function for users.

P: Yeah, definitely! If users can see reviews for each gym they will be satisfied.

R: That’s really important because that can show users ok these are the fifty reviews comments and they can understand a bit about that.

P: Yeah, reviews are great so all users are aware of the gyms facilities.

R: So, I also think the gyms need to uhm have the ability to be able to filter what is important from those comments and what is not uhm because if there are fifty comments you want to read the twenty that are very important but the other thirty are not really relatable so I think if the gyms can collaborate with the app or the app developers so they can be like ok this point is important for users uhm lets have this on the app uhm if it’s someone commenting about something that is not really related to accessibility than remove that.

P: Yeah.

R: So, having that ability to go onto the gym and see what is accessible but also reasons as to why it’s accessible I think is important.

P: Yeah, and also having the ability to filter whether somebody with your impairment has been to that gym.

R: Oh ok, so sort of filter it based on the impairment?

P: I think yeah ugh the end goal could be that because uhm I think if I knew somebody who went to a gym local to me who had dwarfism it might uhm and let’s say there are four local gyms near me but I know one person with dwarfism that goes to that gym then I would personally choose that one over the others.

R: Ok.

P: Rather than uhm because I know somebody with my condition who has been to that gym, and I guess if they put a little comment or a little review than they have kind of done the work there and yeah I mean I don’t go to the gym now but in the past when I was at University it kind of took me two or three gyms to attend and do the free trials before I found the one I felt comfortable in uhm and I benefited from more so yeah it had more for me personally whether it was the machines uhm the classes ugh the swimming pool but yeah I think it would be good.

R: Yeah, I think so far it has the options you know you can phone them you can contact them but it’s never quick enough.

P: Yes, a phone number and opening hours are good but it’s missing information.

R: So, I think if someone can read a previous person’s comments or reviews it gives them a bit more of an insight on why that specific gym has two or three stars because when you click on the gym it might have three stars and you’re thinking oh so why has someone reviewed this as three stars so by giving an explanation as to why there are three stars or four stars it can really help users to feel comfortable before they make the effort to go to the gym.

P: Yeah, and I know that if you go on like trip advisor or bookings dot com when you’re booking a hotel there is always kind of like a main review but it does come up with like six or seven things underneath which you can star so whether that is uhm I don’t know location, accessibility uhm again cleanliness and tidiness is not really important but that does come up in a hotel or hostel uhm facilities and ease of use.

R: I always think categorising under things for example like accessibility, equipment because for example someone might provide four or five stars all based on the equipment uhm but whether they could filter the reviews into sub themes under the reviews could then help to quickly locate uhm ok I want to know how accessible this building is uhm I press accessibility ugh you can see the comments and you can see what people think about the accessibility of getting to the gym uhm also equipment uhm and all that sort of stuff uhm what is the accessibility in terms of like bathrooms and changing rooms.

P: Exactly! Accessercise should break reviews down into smaller categories.

R: So, instead of maybe having like fifty comments where you have to read through each single one.

P: Actually I think it would be easier for the individual with the impairment uhm filling it in because I think they would be more willing to do something that was just uhm I don’t know ten sub headings with a star uhm and a little comment box then write a review on their entire experience.

R: Uhm, what are your thoughts for example uhm on a three sixty rotation of a gym for example when you press the gym you want uhm you can see like ugh a rotation of the gym uhm and it’s like an empty gym with no one there uhm but you can see what the gym looks like before you use the gym uhm do you like the idea of that?

P: Yeah, I think that could be useful. A three sixty rotation is always a good idea.

R: Or even like a video attached to the gym so when you press the gym uhm you have the ratings, you have got the phone numbers uhm and the opening hours but there might be like a little video that you press and you can see like a three sixty rotation of what the gym looks like uhm because for example if I find a gym on this app it provides me the name uhm it gives me the reviews but I don’t really know what it looks like.

P: Yeah, ever user wants to see what the gym is like so a rotation would help.

R: Uhm, so whether they can link uhm the website to the gym there uhm so you can press on the website and you can see some photos just to give the user a bit more of an insight before they actually make the effort to go to the building.

P: Yeah, no I think that is a good point uhm personally for me I don’t think that would make too much of a difference seeing the gym uhm but I guess in the past I have gone on websites and clicked the photos so yeah I’m retracting that statement so yeah pictures and things are something that I often look at before I go somewhere and that does sway my decision.

R: So, you think with like a q and a uhm and a live chat on the gyms is valuable?

P: Yeah, live chats, a Q and A or forum would be really useful.

R: Ok, perfect! And in terms of when you first use the app uhm you saw the social function uhm did anything stand out that you liked about that or maybe you didn’t like from that function?

P: I think everybody likes a bit of social media and interaction with uhm different communities and I think for the disability community it is ugh a good feature of that uhm you can kind of put on the workout uhm you have done and I think it would give you that bit more motivation to keep coming back uhm if people are interacting with you and commenting and supporting you on your journey and you are doing the same to them uhm I think it will drive users to the app and keep them there for longer if that community element is there.

R: So, do you think having that social side and will help motivate users to use the app and giving them that drive to maintain physical activity levels?

P: I think yeah, I think that does help uhm a social section on any app is helpful.

R: Ok, perfect sounds great! Uhm, do you have the required equipment to use accessercise in it’s full capacity for example access to a mobile phone to use the application?

P: Yeah!

R: Perfect, so you have got all of that. Uhm, so from using the app so far uhm so you have used it for around a week or maybe a bit longer but are there any improvements that you believe could help improve the accessercise application uhm so is there anything that you really want on the app that isn’t on there at the moment that you might want to suggest to the developers?

P: Potentially like uhm an introductory video uhm perhaps so for example if you were to download the app or even prior to downloading the app uhm an introductory video to show you how to move around the app and what is available on the app because I think I managed to find my way around the app and do all the filtering uhm workouts and saving them and what have you but I think almost like a tutorial for people to get the most out of the app.

R: Right ok.

P: That would be beneficial because I think there are things that people could potentially miss or not understand that are there.

R: Ok.

P: So, that could easily be resolved with uhm a short video uhm and it doesn’t even need to be that long uhm it could be like a two or three minute video uhm this is the accessercise app uhm this is what it is here for uhm this is what you can do and get out of it because you have got to search and look for it and play around yourself whereas if there was somebody telling you.

R: It might be a bit easier?

P: Yeah or even guiding you uhm I wouldn’t say guiding you but saying did you know that you can put the workout in your calendar.

R: Yeah, I know what you mean.

P: The questions that it asks are very uhm easy to be answered and once you have got past that stage and it comes up with the videos you can then kind of left to your own devices whereas if you were somebody who wanted to do uhm I don’t know like leg day uhm arm day uhm this day you could completely set up a full routine and some people might not realise that functionality exists within the app so almost just like a basic introductory video to the app would be helpful.

R: Perfect, ok! So, I’m just going to tailor this question to the different components of the app so for example the shop, the nutrition uhm podcasts uhm the blogs uhm is there anything that you think they could improve on when it comes to them or something that they might want to consider when delivering those sections uhm so for example like nutrition and the shop is there anything you think in particular that you would appreciate as a user having there?

P: Uhm, maybe in terms of the shop it doesn’t necessarily need to be advise if there was as I go on this fitness journey myself kind of thing when I need to get uhm because I would be doing it from home rather than in a gym environment uhm maybe links to dumbbells and kettle bells that they would recommend.

R: Ok.

P: And where I could get this equipment from that you see the people in the video using.

R: Oh, so sort of like a link to the equipment that is used by the demonstrators in the video?

P: Yeah, so it doesn’t necessarily need to be accessercise selling that stuff but if you need a medicine ball uhm go to decathlon or whatever it might be uhm sort of like a quick and easy link and I know it’s so easy to google things but people can be lazy and if the link is there and ready you are more likely to do it instead of doing your workout uhm and thinking oh next time I need a medicine ball ok I’m going to go on google and find one uhm but if it is very much like have you got the equipment for this exercise if not click here to buy it uhm I think that would really help in terms of that and then nutrition I think it all comes down to including general advice uhm.

R: So, when you say general advice do you mean like sort of what foods to eat for your five a day or what is good for carbohydrates uhm what is good for protein?

P: Yeah or maybe kind of uhm like recipes to healthy eating uhm I think for me personally it’s more healthier snack options that you can like make at home rather than I mean like for example me and my wife we do the weekly shopping we know what we are eating and we do have a varied and healthy diet in terms of that but I think for me individually it’s the snacking that I find is my downfall.

R: Don’t worry it’s the same for me haha.

P: Yeah, healthier snacking options in the evening or something uhm.

R: Would you like uhm I know maybe it’s not an option but do you think maybe uhm nutrition plans or healthier eating tips depending on the impairment or do you think that is too focused or unachievable?

P: Uhm, I think potentially that could be an option maybe but it wouldn’t be something that I would personally tap into only because of the children and again it sort of sounds like I’m using this as an excuse but family life we all eat the same meal every evening whereas if I was eating something the money side of things and the cost of living at the moment wouldn’t be something that I would go down but if it was somebody living a different lifestyle to me who wanted to go that extra bit further than potentially it could be useful.

R: So, you like the idea of maybe breakfast, lunch and dinner recipe plans?

P: Yeah, or just kind of like uhm almost healthier meal options or recipes.

R: Ok.

P: Yeah.

R: Yeah, that makes sense perfect. And in terms of podcasts and blogs uhm do you think there is anything that you think they could improve on and look to roll out or?

P: I think in terms of blogs and podcasts it’s targeting the audience and giving those using the apps the opportunity to write a blog that features them in it so it’s not necessarily uhm so almost like it’s user lead to some degree that if you have been using the app for a month uhm and at the end of the month you send out a mass email to everybody and if they wanted to they could share their journey so it allows everybody the opportunity to feature in the blog.

R: But do you like the idea of the blogs?

P: Yeah, definitely! I think hearing about other people’s fitness journeys and what they achieved is a huge motivational side of things uhm and also finding out how other people do it uhm when they do it uhm do they do it in the morning or the evening or what they are doing uhm are they doing the same as I am doing uhm are they doing a full body workout uhm are they targeting different areas on different days uhm just to see how other people are doing it and how they are going about achieving their goals and whether or not you are doing it the same as them but getting the same results or you could perhaps tailor your way to their ways to see if the next month you do what they have done and your results are higher.

R: Right, ok that makes sense! So uhm going back to the point about motivation so obviously new years resolutions people do a new resolution and obviously sometimes after thirty days they don’t fall it up so that long term behaviour change is not always implemented uhm but do you think anything in particular you would like to see to keep you motivated to use the app uhm is there anything that the app developers could really do to keep you held onto the app for a longer period of time?

P: I think it would be perhaps useful to add extra exercises or varying the exercises so for example if I have seen all the content on the first day of getting the app and then there is nothing new for me to do I don’t think that would motivate me uhm perhaps the opportunity to see live workouts potentially or uhm online classes that you could follow so instead of exercise by exercise and I know you can create the workout for yourself but if there was more uhm I don’t know ugh like a cardio blast for thirty minutes or ugh a hit workout for thirty minutes uhm you don’t necessarily pick the exercise that you want but somebody with your impairment is doing something for thirty minutes and you follow that so it kind of ugh develops in that way because I think that once you have found your exercises and you stick to them there is nothing that would keep me there because there might come a time that I have done it and I have been shown how to do it by the video tutorials but what is the benefit to me for staying on the app because I can easily write down on a piece of paper the ten exercises that I’m doing on a Monday uhm the ten exercises uhm the ten exercises on a Tuesday I don’t actually need to see somebody on the app doing it so once I feel comfortable enough doing it and there is no variation or change uhm due to the cost and things you might kind of see a drop off because they have almost got to where they have got unless for me personally at the moment it isn’t about getting stronger necessarily it’s just about being fitter and toned and healthier so if I’m not kind of upping the weight or upping that then there’s no kind of reason to come back uhm.

R: Do you think there is anything so earlier you said about motivation and you know over a period of time your uhm enjoyment for the app might decline but do you think there is anything the app developers could add to the app for example like streaks or discounts or leader boards or competitions that might really uhm for example you know like uhm on Apple watches uhm and fit bits they have like ugh do a thousand steps today and you have your achievement for the day.

P: Yeah, a lot of that so streaks, daily, challenges, discounts would help me a lot.

R: Do you think there is anything that they could do in terms of the social section that might help with that?

P: Yeah, I think maybe uhm a community there who is kind of talking and sharing and potentially some sort of leader board that you could work up to might motivate people uhm personally for me a leader board and things wouldn’t be something that would motivate me as an individual but I can’t speak for others uhm and I do know others that would be interested to get from the top ten to the top five and what have you but for me personally I don’t think that would be a draw to me a leader board.

R: Do you think like maybe any nudges uhm and notifications to encourage you to do physical activity could be a good thing so for example like if you want to work out on a Monday morning uhm and do a chest workout and your phone sort of vibrates and reminds you to complete your workout uhm do you think that sort of nudge in the first place might help?

P: Yeah, definitely and I think reminders and things are always a good thing and I think the thing with the app is I guess there’s no feedback there almost once you have done the workout you have done it and the only feedback which is amazing which you get after it is what you are feeling yourself and there’s no one telling you oh great well done uhm come back again tomorrow and we will do it again.

R: Ok, so you would like some detail feedback or some meaningful feedback that might encourage you?

P: Yeah, I think if accessercise could provide more feedback would be great.

R: I know what you mean.

P: Yeah, like even if it was just ugh I don’t know how to explain but yeah almost like ugh a well done for completing today’s workout you showed up uhm you did it ugh you have exercised uhm you are one step closer to your goal today kind of thing uhm for me it works because it makes me want to come back and do it.

R: Do you think anything in terms of the social side that you have seen so far uhm so you can follow people uhm you can react to their uhm workouts but do you think there is anything that they can work on in terms of that uhm do you think they could have more groups uhm or groups tailored towards an impairment uhm because I think at the moment the social side is good but I think it’s still in it’s early days uhm could they do more could they add some functions to make it a bit more interesting?

P: Uhm, are you able to share things on the social at the moment?

R: Yeah, so what happens is so you do the workout and you can post it to your profile uhm.

P: So, you doing the workout?

R: Uhm, your workout for example.

P: So, the videos that you have recorded?

R: So, the videos I think are already pre-recorded.

P: Yep.

R: But I think what would be good is whether you can actually do the workouts yourself uhm maybe record it and other people can see how your technique and stuff like that and that may make more of an effort to record it and post it.

P: Yes, it would be good if you could record and post your own video on social.

R: So, at the moment what you do is you complete a workout and it says like you know well done ugh share, don’t share or cancel or something like that so you have the option to share your workout to your profile so other users can react and they can like your workout or you don’t have to share so it depends on that privacy setting and what you want uhm but in terms of what you see so far from viewing the app do you think there is anything that they can work on for that?

P: Uhm, maybe the option to kind of uhm I don’t know like post a picture of you with your water bottle for it to be more personal because to me all you’re doing there is sharing the pre-recorded videos of other users uhm basically the content of the app onto another platform within the app whereas if there was more I don’t want to say Instagram but even like a picture of where you have done the workout so like your home uhm so like the weights on the floor next to your water bottle or taking a selfie at the park.

R: So, it’s more tailored and more uhm individualised?

P: Yeah.

R: I know what you mean and I think with the videos I think uhm you do the workouts but it’s not actually you doing it uhm you’re just following someone that has already done it because it’s pre-recorded it’s not meaningful.

P: Yeah, like I almost wouldn’t call it a social area uhm it’s almost like an area where people can go to look for uhm oh this is a workout that they have done uhm they can then go and copy that workout.

R: Do you like that function where you are able to copy uhm for example if someone did a workout that you are really interested in uhm the option to share a workout between each other?

P: Yeah, that’s almost what I see the social element as it’s more of a ugh this is the workout I have done uhm do you want to do my workout so I think perhaps social is the wrong word for that bit of the app uhm it’s almost like yeah this is the workout that I have done today uhm here it is if you want to go and do it and the more that share the more variations of the different workouts that they have done.

R: Uhm, would you like uhm sort of like a news feed where people can share uhm sort of like Twitter where you retweet and like and other people can see what you have retweeted so like uhm you have got the social where you can react and comment and so on but would you like it to be a bit more like Twitter where you can see like other people’s workouts and share what they have done with other people that follow you or is that a bit too much?

P: I think yeah in terms of the whole sharing and whatever that’s not necessarily what I would go onto a fitness app for but I would go on it to scroll and sort of look to see what others have done or not I could copy what they have done so obviously this one here says shoulder stability for wheelchair users uhm that would be useful if um.

R: You’re a wheelchair user?

P: Yeah, if that was something that I wanted to do uhm so yeah I think the sharing element of your workouts and the opportunity to put in a title and how you rated that workout yourself and how you felt afterwards would be good.

R: That makes sense perfect. Awesome, so we will go onto the last question so do you use any other similar application outside of accessercise uhm if so what is better or worse than accessercise uhm so you mentioned you use another app.

P: Yeah.

R: So, do you think it’s maybe uhm it has similar features to accessercise or is there anything that stands out from that app compared to this app uhm that maybe accessercise can use from their app?

P: Yeah, so I’m using the body coach app at the moment uhm I started in January using it and finding it really quite useful and I’m going back to it to do it so my motivation has of yet hasn’t dipped and I think the thing with that is that the variation of it so it’s kind of for the first month I did ultimate beginner and at four weeks I checked in and now I am on beginner for the next four weeks so it’s very much a variation of uhm body workouts uhm but at the moment there are no weights or anything involved it’s kind of like jogging on the spot uhm press ups uhm lunges uhm I’m trying to think of what else.

R: Do you think there is anything from that app that you think maybe might uhm benefit the accessercise app that you have seen so far that you think ok I like this on this app but maybe this hasn’t been included on this app?

P: Uhm, I think uhm there is a lot of talking by Joe Wicks in it.

R: So, sort of like the commentary feature?

P: So, even though you are not with him in the room uhm so the sort of way that he has pitched it uhm is working for me personally at the moment and there is always things that I can’t do due to my disability but he says don’t worry if you can’t do this uhm do something that you have done before and because there are five workouts with twenty five things or whatever it might be uhm so if there’s something I can’t do uhm it doesn’t need to be something that I have done in that workout uhm I can do something from the workout that I have done before uhm I can replace so for me I find doing a burpee really difficult to get up and down but instead I will replace it with a star jump for example uhm and then kind of like at the end of it uhm when you have watched the video it will say well done and uhm you are doing great.

R: So, sort of like positive reinforcement?

P: Yeah and you do get sort of like small badges and things which is great and is provided all on the app so it says uhm at first you have completed your first workout then it’s you have completed five workouts and then the numbers do increase so I think it was one, five, ten.

R: Uhm, twenty-five?

P: Twenty uhm one, five, ten, twenty uhm thirty but now it’s fifty.

R: So, it’s a bit of a jump haha.

P: Yeah.

R: But again I think that’s quite nice because it means like ok I need twenty before I reach my next goal uhm let’s put in the work.

P: Yeah, so obviously at the beginning they were shorter because you are at the early stages of it so you need that but then yeah it goes fifty to one hundred or whatever it might be.

R: So, I agree with what you’re saying I think the star system and those little achievements are important and I feel like at the moment accessercise hasn’t got that and it’s definitely something that they could benefit from where they have those little perks where you know I have done five workouts congratulations uhm a bit of praise uhm a bit of motivation.

P: Yeah, for me there doesn’t need to be money off something uhm it’s just somebody telling you thank you for sticking with the workout uhm you are doing great uhm come back tomorrow for your next.

R: Exactly, so you want to keep building the motivation over a period of time.

P: Even like small things like I can’t wait to see you at the next week out or small bits of praise and then yeah I think drip feeding more content in as well like whether that is uhm more kind of like the thirty minute videos uhm that Joe does or whatever where you are doing something following somebody and they are talking at you.

R: Yeah, I think that’s important uhm you have those videos where someone is directing there attention at you.

P: Yeah, exercise videos can be really helpful in demonstrating the exercise.

R: You have got those streaks you have got those achievements uhm and sometimes those small things are the most important things.

P: Most definitely! Any form of streak or challenge will really help me.

R: If you use an app and it hasn’t got them then what is your reason to use the app uhm because there is no enjoyment or excitement but if you have something that pops up on your screen like for example you wake up in the morning and it says today we are focusing on arms uhm keep going you are nearly hitting your goal.

P: Yeah, I think every app should have some challenges and awards.

R: You think ok I’m going to work on this today.

P: Yeah, it’s almost like somebody is there telling you rather than creating your own thing and doing it like I have said once you have created those workouts and done it for a couple of months.

R: You can then easily get bored of the app because there is nothing keeping you motivated.

P: Yeah, if it’s the same video that you are constantly watching to do your lunge or do your what ever it may be uhm it would get very dated and repetitive almost.

R: Yeah, it’s like the same thing over and over again you start to feel like I’m opening this app uhm I’m not going to enjoy this app uhm I’m just doing it to be helpful whereas if they can get you to be healthy but also have some excitement on the app uhm it can actually improve your willingness to do that which I find very valuable.

P: Yeah.

R: Right, so now what we are going to do is go through the actual think aloud protocol so we are going to move onto the main part of the study and ask you to participate in a think aloud protocol while you use the accessercise application uhm the instructions for a think aloud protocol are straightforward uhm I would like you to say out loud whatever comes into your mind uhm there are no right or wrong answers uhm we will just run through a quick practice to get you used to the protocol so could I ask you to say out loud everything that comes to your mind when you think about physical activity uhm so whether it’s hard or easy in terms of like the activities or equipment uhm so anything related to physical activity?

P: Moving the body, raising your heart rate, being active uhm getting fitter.

R: Perfect, awesome! Uhm, so what I would like you to do is to open the accessercise app.

P: Yeah.

R: Uhm, so obviously you have logged in but what are your first impressions when you see the app uhm do you like the colours uhm do you like uhm what you see on the screen uhm do you think there is too much information on the screen there?

P: No, I think the layout of the app is easy to work your away around it uhm the colour scheme is fine for me uhm.

R: And what do you think of the four options down the bottom uhm do you like how that is sort of out the way how it’s down the bottom.

P: Yeah, no I do like it uhm the layout and it very much feels like Instagram.

R: Haha do you think that is a good or bad thing?

P: I think it is a good thing that people know about it and uhm they are used to it so you are not having to uhm yeah I think it’s a good thing.

R: Good thing ok. Uhm, so for example we are on the social section so for example if you press Liv’s name and go onto her profile uhm what do you think about this when you see this uhm anything come to your mind when you see this?

P: Again, Instagram comes to mind there uhm.

R: Do you like the fact that there’s a bit of information uhm not too much information about the person uhm it’s got their age, their gender, uhm maybe sometimes it has their goal of what they want to achieve from the app.

P: Yep, no I think yeah it has everything you need on a profile page.

R: Do you think there is anything else that they could include in that section uhm maybe to make it a bit more interesting?

P: Ugh, maybe allowing to include their impairment on the profile would be good.

R: Oh ok so you think maybe having their impairment there so other users can understand about them?

P: Yeah, because you may want to connect more with those with your impairment not saying you would but I do think for example uhm what I was talking about earlier in terms of uhm the social side to it with the workout that was shared uhm if you would then follow uhm more people with your impairment then your feed would come up with more workouts that you could personally do yourself so yes I do think that would be useful.

R: So, I mentioned it earlier about how you uhm can post your workouts but at the moment you can’t export your workouts out of the app onto things like Facebook and stuff like that, but do you think maybe things like that would benefit some users uhm how they might be able to export it out onto things like Twitter uhm and post it on like their Twitter uhm or do you think there is like no benefit of that.

P: I think the only benefit would be to make more people aware of the app itself uhm but for the individual I don’t know uhm I guess there is some sort of benefit if people are kind of like interacting with it uhm obviously the dopamine hit you get from the like uhm and people commenting uhm if you are starting your fitness journey and people are seeing you doing it uhm they could motivate you uhm so yes and no.

R: No, ok. Uhm, so obviously you went onto Liv’s profile and you can see a bit of information about them uhm so for example if you go onto Liv again.

P: Oh, sorry!

R: There is a functional that says achievements.

P: Yeah, achievements is good to have to encourage users to exercise.

R: Is there anything in particular that you would like to see from the achievements would you like to see how many uhm I don’t know how many awards she’s been given uhm is there anything you would like to see improved from that function uhm I think it’s still yet to be developed.

P: Yeah, I guess it’s down to how long they have been using the app and how many workouts they have completed I guess.

R: Uhm, what I think would be quite interesting there is uhm like daily uhm weekly or monthly awards.

P: Yeah, daily, weekly or monthly goals is what accessercise could benefit from.

R: So, your monthly reward might be uhm do a strength training workout uhm flexibility workout and a toning workout and if you have done all three of them then that’s your monthly goal.

P: Yeah.

R: Uhm, your weekly goal might be uhm one flexibility session and twenty minutes of cardio.

P: Yeah.

R: And then your daily goal might be like a thousand steps or something so you can actually see what their goals are and whether they have achieved them goals.

P: Yeah.

R: Uhm, it would be interesting to be able to provide them feedback on that so if they haven’t achieved their goal then maybe you can provide a comment and say you know uhm this could be improved uhm maybe you want to consider this and stuff like that uhm do you like the fact that you can see their followers uhm in terms of privacy do you like it uhm it’s not really like a dating app uhm you know you can see what they like uhm who they are or whatever.

P: Uhm.

R: It’s quite simple, isn’t it?

P: Yeah, I don’t see the benefit to seeing who they are following.

R: Ok.

P: And it’s important to remember that it’s a fitness app uhm not an Instagram or a dating app or whatever it might be but then I guess going back to uhm and I keep going back to the same comment about people that may have a similar goal with you in mind uhm you may want to see who they are following.

R: Ok.

P: Uhm, so you can link with them to see the workouts that they are sharing for example.

R: Yeah, I agree.

P: So, yeah, it’s hit and miss I guess there is a privacy element to it.

R: I think the idea of having like groups for example if you were to join I don’t know like wheelchair basketball uhm group you can see who else is linked to that group uhm who they follow and maybe you can see what they do or something like that.

P: Yeah.

R: I think it has it’s relevance but I think if it wasn’t impairment or goal filtered then there would be no reason to have it but I can see the benefit of having it because you might follow someone that does amazing workouts uhm then you are mainly following them for motivation so sort of like YouTube where you subscribe to someone because you like their content uhm and there channel uhm you see what they are doing on a regular basis and it’s the dopamine effect where you get excited to tune into what they are doing.

P: Yeah.

R: And I guess if you have those groups where every Monday you come together where you can see people’s workout uhm and it has that incentive to get up and do something uhm so the social side I can understand it’s quite useful uhm in terms of exercises so do you like the fact that there are a range of different exercises when you filter uhm do you like how you have your favourites uhm my exercises uhm my workouts?

P: Yeah, definitely, I think. My workouts, my favourites are all good.

R: There is not too much on the screen?

P: No, I think it’s good that you can favourite the exercises once you have kind of found something that you enjoy uhm doing and performing uhm and it makes you feel good.

R: Do you like the way that they have listed the titles uhm there is a video link next to the video?

P: Yep, no I think uhm and obviously there is the equipment you need and what area of the body it is focusing on I think.

R: Uhm, do you think ugh maybe just by looking at the cover of the videos uhm there is anything they can do to improve the videos?

P: No, because I think people just click on the video to find out more uhm I don’t think uhm I think they call it like a cover image I don’t think that is necessarily needed unless uhm no I don’t think that matters.

R: So, you like the different exercises that are there and for example it’s even got the little flag thing so you can press it and it favourites the exercise.

P: Yeah, I think that is really useful that it has that feature to favourite exercises.

R: You can also like unfavourite so for example what is quite good is that if you have a favourite exercise you can add it to your favourites and then to your workout and so on uhm so for example this section that you are on now.

P: Sorry!

R: Do you like the layout like how here it doesn’t have too much information uhm does anything come to mind when you see this?

P: Uhm, I’m trying to think uhm there is a lot of wording to explain the exercises but then again I guess we can’t really avoid that but whether or not it could be split into paragraphs maybe uhm but that’s my initial thought because underneath the title of uhm so I’m on barbell bent over row it’s telling me it works on the back arms and lats and that it’s a moderate intensity exercise.

R: Do you think maybe they could do like bullet points?

P: Yeah, bullet points for each exercise is helpful to break information down.

R: So, do you think at the moment there is just too much information uhm a bit wordy there?

P: Yeah, there is too much information per exercise with the big block of text.

R: Because is this about what type of exercise?

P: Barbell row.

R: Ok, so it’s got sort of like step by step instructions which I think is always useful uhm so you have got the videos uhm you have got the ugh how to do it step by step but I think they could possibly lay it out like step one uhm step two uhm step three.

P: Yeah, and even the bit there which says this exercise works the lats uhm back and bicep uhm that should link in with that whereas that says back arms and lats.

R: Yeah, it needs a bit more flow.

P: Yeah, that is not necessarily needed because it’s already said up there.

R: Yeah, I know what you mean and do you like the key benefits do you think maybe one benefit is enough or they should add more uhm maybe two or three?

P: Uhm, obviously the more benefits to an exercise the better but again I guess it depends on the benefit to that exercise is.

R: Do you like the diagram here?

P: Yep, personally it’s the first time I have seen this diagram at the bottom uhm I like how it looks like someone with dwarfism so it’s not an average height person uhm.

R: So, they have very much tailored it to the impairment?

P: Yeah, so to me is a big tick that the app focuses on the impairment.

R: I would be interested to see when you press amputee whether the exercises for that has a diagram of someone with an amputee.

P: Yeah.

R: I think that would be really interesting to see uhm but I think that is really an important feature of the app uhm that it is tailored to what the person wants uhm and what their impairment is uhm because we know people with a range of different physical disabilities are different from each other so it has to be focused on the individual and the guidelines and recommendations that you provide for physical activity needs to be focused on that individual.

P: Yeah, a key benefit of this accessercise app is that it focuses on the impairment.

R: Uhm, so I do think that is one good feature of the app uhm do you think there is anything else that could be improved on this section uhm so at the moment have the videos uhm you have got the description uhm the key benefits and the muscle components.

P: Uhm, yeah I really like the videos, exercise descriptions, and key benefits.

R: So, it’s got the videos of someone relatable doing the exercise.

P: Yep, ugh maybe that bit there could be condensed down and you can press read more if you wanted to uhm if it has already explained it uhm so what the video is saying is what is already said down there in the description uhm so you want to be able to get to this bit here quicker uhm I would have the exercise name and that bit and then maybe a read more if you wanted to expand it so then it can go to key benefits quicker.

R: Right, ok makes sense. I guess you want to have the information but you don’t always want uhm actually what is this here uhm but do you think seeing that there the videos and the description uhm it’s a good feature to have?

P: Yeah, the videos and exercise descriptions are good features to have.

R: Ok, perfect!

P: Yeah, like it’s there where it says show more uhm to me the picture of the person with the muscle groups should be higher up almost.

R: Ok, but do you think the order for example is ok the fact that it has got the video uhm the description uhm the benefits and then this or do you think maybe they could start with the photo at the top and then the audio description or?

P: Yeah, even like key benefits is a big one uhm then again.

R: I think the order could be quite important because if you see like a photo of ugh the body or a key benefit at the start you may want to read more.

P: Yeah.

R: Whereas if you have a big chunk of text at the beginning you might think oh gosh.

P: Yeah, people might not even go down to the bottom which yeah could potentially be ordered slightly different.

R: Perfect, ok! So that’s all the exercises uhm you have seen what the uhm exercises are all about uhm you have seen the audio descriptions but if you were to press explore uhm do you like the fact that you can search ugh for the post codes and so on?

P: Yep, I think it is a good function to be able to search for gyms using postcodes.

R: Uhm, because you mentioned earlier on that you may not use this function but obviously accessercise is all about accessibility and they are trying to make it as accessible as possible uhm do you think there is anything they could possibly improve on there?

P: No, to be honest I think it has everything on this element and when you scroll out you can see all the features.

R: For example, if you were to press on one uhm let’s say this uhm for example this one has four stars uhm and it’s powered by google but at the moment there is nothing really there so for example if you were to go to this gym you could rate it and give it a four out of five but from just seeing this do you think there is anything more that the gym could do or the app developers could really develop in that section uhm because to me it looks a bit bland.

P: Yeah, I think perhaps having more criteria for the accessibility side of things or a picture of the gym even the gym entrance.

R: Did you say a picture?

P: Yeah, images or a video for each is helpful to give us an insight into the gym.

R: Ok. I think what would be quite interesting as you said uhm a read more section where it’s got accessibility here uhm it’s got equipment and underneath that it has a rating uhm or they could do the rating based on those sub themes so they have accessibility four stars uhm they have got equipment five stars uhm because I guess when you go to the gym and you think about accessibility you’re also thinking not just equipment uhm how accessible is the car park uhm how far is the car park to the gym.

P: Yeah, and whether there is any parking at all.

R: So, I think it’s great having four stars but they need to break that down further.

P: Yeah, I think my only comment on the mapping itself is what do the different logos mean so uhm obviously there is one with a bar bell on uhm and then one with the accessercise logo.

R: Uhm, so if you press on the bar bell one uhm I think the bar bell ones are the ones that are currently under review or being judged for accessibility but the ones with the logo have actually ugh been reviewed uhm for example this one has zero stars uhm and if you press on another one.

P: Yeah, so I think the ones that have stars have been reviewed and have the accessercise logo.

R: So, this gym here has five stars so it would be very interesting to understand why this gym has five stars you know.

P: Yeah, they need to provide more information on why the gym is accessible.

R: So, here it has the opening hours uhm it’s got the times they are opening and closing uhm they have the phone number but I feel like they could definitely do a lot more you know.

P: Yep, what I have noticed is that a lot of the ones that have been rated are four to five stars and there’s no way that every single gym is four to five stars for people with a disability.

R: Yeah, it would be interesting to know why and yeah, I think comments is important because everyone could say it’s five stars but when you actually turn up it’s just a two out of five stars but a reason to explain why it’s a five star is really important.

P: Yeah, I think the reviews are good but they are lacking information.

R: I feel at the moment it lacks that information.

P: I agree. I’m wondering what exactly makes the gym accessible I’m confused.

R: Perfect, ok so if we move onto the next section.

P: Yep.

R: So, we have done exercises uhm and if you go back to exercises actually uhm and press my workouts uhm top right uhm and what we can do now is uhm if you press calendar uhm if you choose a date uhm you can then add the workout to the calendar and as I said before if on Tuesday’s you want to do ugh legs uhm Wednesday you want to do chest you can add it to your calendar so it saves that time uhm so if you want to have a workout plan in place for every day of the week it then saves your time so you can spend uhm I don’t know like thirty minutes designing your workouts and that will save you hours over the next few months just by adding these workouts to your uhm calendar which I think is quite a good function uhm so do you think this calendar section is quite good?

P: Yeah, I think it’s useful and you are more likely to do physical activity and complete the workout if you have kind of spent time on a Sunday doing it for the next two weeks or whatever that you know so it would be good to have some sort of function where you can link uhm so I am an iPhone user but it does remind you that today is Monday so please do your workout uhm so is there a time element on here?

R: Yeah, so when you do your workouts there is uhm a timer so you can time your workouts but there is also a notification option so ugh they send you emails uhm they send you notifications through the app.

P: Oh ok. Notifications are always a good idea.

R: So, maybe something like a nudge or a vibration on your phone may help.

P: Yeah, I agree.

R: So, do you think there is anything that the calendar could be useful for uhm in terms of collaboration with other people or companies uhm because I guess what I would find is like yeah you have calendar for gmail uhm you have calendar for work uhm a calendar for accessercise uhm it becomes a bit.

P: Yeah, if there was a way the calendar could be centralised uhm I mean I guess one thing about the calendar that could be put on by accessercise is some of the different days uhm for example like there is national fitness day uhm there is uhm international day for people with disabilities and cerebral palsy and dwarfism awareness month and very much linking it into that later down the line.

R: Uhm, so for example if it was cerebral palsy day uhm having videos of you know like physical activity opportunities or local events going on for that impairment.

P: Yeah, some information regarding sport opportunities or event would be good.

R: So, it sort of celebrate the impairment uhm I agree with that uhm I know what you mean. Right, so we have done explore uhm we have done the exercises uhm now if you go to uhm add a new workout uhm what thoughts come to your mind when you see this sort of stuff uhm so you can uhm design the workouts uhm for example do you like the location option.

P: Yeah, no I think it’s good that you can either do it at home, gym or outside uhm the goals.

R: For example, I like the way that it says toning and not losing weight uhm so they are being appropriate in the terminology if that makes sense.

P: Yeah, the terminology they have used for each goal is good.

R: But also I like how you have stretching, mobility, flexibility because this app is not just focused on children or young people but in fact it’s focused on a range of ages uhm so even if you’re like eighty or nighty you might not want to go running but you might want to do some stretching at home so it has a timer so you can select how long you do the workouts and so on uhm and you can add your exercises uhm and you have the filter section uhm so for example if you add some random exercises and then scroll up and choose your goal uhm so select get toned uhm home uhm select timer it will provide you with a list of appropriate exercises then select them and save the workout.

P: Looks nice. The app has some cool functions like designing your own workout.

R: Do you like the fact that it’s got the repetitions and sets?

P: Yeah, I’ll go back to what I was saying at the beginning uhm a tutorial of how to navigate through the app is really important.

R: Yeah, and also for some users they won’t even know what repetitions and sets are.

P: Yeah, an introduction video to the app would be great!

R: Not everyone will know about that terminology but I agree uhm we know what repetitions and sets are but uhm we know what intensity and frequency and stuff means but not everyone that uses uhm an app will know.

P: Oh, so it’s a bit of praise.

R: Yeah, it’s got that. Uhm, but what could they add to this section so uhm you design your first workout uhm what could be added uhm because it’s quite robotic isn’t it uhm just congratulations.

P: Yeah, they need to add more feedback when finishing workouts.

R: It would be nice if they could personalise the congratulations uhm message based on your name uhm so like congratulations Tom.

P: Yeah, they should personalise the congratulations message with my name.

R: So, it uses your name which you insert when you first sign up to the app and it uses that.

P: Yeah, and it’s a little bit confusing how it’s using uhm an emoji from WhatsApp as well to me.

R: For example, going to the final question on this section uhm so if you were to use the app how would you use it would you go straight to exercises uhm would you go onto socials uhm so if tomorrow you had to use it uhm and you log on and you land straight on the home page what would you first be checking out uhm would you first be designing a workout uhm would you go to account settings uhm would you change your filters and choose your impairment?

P: Yeah, I would probably make sure that all my account settings were correct and my exercise habits and things and then I think I would start by playing around with the workouts and creating workouts for different goals yeah.

R: So, would you have different goals uhm so for example muscular strength, cardio?

P: Yeah, yeah to see what it came up with.

R: Awesome, so you have pretty much seen everything about the app uhm and my biggest advice is that if you wanted to build strength and you feel like you can’t find the exercises uhm always use the filter function.

P: Yeah, I like the filtering option when designing workouts it’s helpful.

R: That will help based on what you’ve selected uhm it will help you to design the workouts uhm there are over two hundred and fifty exercises so even though sometimes you may have an exercise which you know of by heart, and you can’t find it uhm on this app it may be an adapted exercise with a different name.

P: Yeah.

R: So, for example you may want to do dumbbell press uhm I don’t know some sort of exercise but on this app it won’t always be named that so when you filter it uhm it will provide you with alternative exercises.

P: Yeah, that’s what I like about accessercise it’s adapted to your disability.

R: We have to remember that the app is not always focused on gym exercises uhm it also focuses on home exercises so they have alternative adapted exercises available uhm but yeah perfect so we will go onto the final three questions.

P: Ok.

R: So, based on what you’ve seen on the app uhm you have used it uhm you have used the functions uhm you have had a go uhm what would you say are the potential benefits of using the app uhm if there are any of using accessercise for improving physical activity levels?

P: Uhm, I think the benefits are to kind of get people up and active and seeing other people with their own impairments doing it uhm I think the longevity of people using it would be longer because they are seeing somebody like themselves I think yeah it’s good.

R: Do you think with COVID and like the fact that these exercise apps that are being developed can help people even when they stay at home for example if you have Parkinson’s or something uhm you might not feel comfortable going outside.

P: Yeah, definitely uhm I do think it has got a good target audience of those who are comfortable going out and to the gym who want to work out from home or a park or in the garden.

R: Perfect, awesome! So, this is a bit of a trick question but what are the potential negatives if there are any of using accessercise for improving physical activity levels?

P: Uhm.

R: So, maybe any of the social media functions uhm may be a problem?

P: Yeah, uhm.

R: I don’t really feel like there are any negatives uhm I mean like obviously there are negatives but I feel like these apps actually provide so many benefits to help improve physical activity for people with disabilities.

P: Yeah, I don’t feel like there are any negative reasons for using the app uhm I think there could be some things that could be added to the app to improve functionality and usability that would help the app appeal to a wider disabled audience.

R: Perfect, ok and the last question of the interview is uhm how do you think the accessercise app could be improved if anything in its use for improving physical activity levels uhm so obviously you have looked through the app uhm and you have mentioned a few areas for development and stuff like that but if you had to name for example three key areas that the app could be developed further what would they be?

P: Uhm, I think it is ensuring that regular content for each impairment is added and changed almost uhm.

R: So, like regularly updating the material?

P: Yeah, so as soon as you get it and use it you have all these exercises and like I said once you have created your workouts and done them there is actually no need to keep coming back and repeating uhm watching the same video over and over again uhm I think feedback is important for people to get uhm even if that is just a simple message at the end uhm saying something like congratulations come back tomorrow and do this uhm to just kind of reinforcing the users that they are doing a good job uhm they are turning up and doing the tasks uhm is a way that the app could be improved.

R: How about in terms of like the social media, shop and stuff like that uhm that stands out that needs to be developed?

P: Yeah, I think yeah obviously when the blog and podcasts things get developed that would be a good way to draw people to see other people’s journeys and what they have achieved.

R: Perfect! Awesome and the final question is uhm is there anything else that you would like to add or discuss?

P: Not that I can think of James thank you.

R: Perfect, thank you so much Tom for the interview I appreciate all your feedback on the app and I’m happy that you have gained a bit more of an insight into the app which is great.

P: Brilliant.

R: No, thank you!

## Transcript of interview undertaken with Participant 11

**Key code:**

R: Researcher

P11: Participant

R: So, thank you Stephen for participating in this usability study so I will start of the interview with giving you an overview of who I am uhm what my role is within the research project uhm the aims, importance and purpose of undertaking this research project uhm so during COVID-19 we noticed that people with disabilities were not physically active due to lockdown restrictions and therefore this increased the risk of things like diabetes, heart disease and so on so uhm we found that during the COVID-19 pandemic uhm the use of smartphone uhm and mobile health applications were an effective way at increasing physical activity and reducing those risks uhm to their health uhm the University that I attend is Loughborough University and I’m a Doctoral Researcher uhm and I am based within the School of Sport, Exercise and Health Sciences and I will be working on this project uhm with Sam Breary uhm which is the director of Accessercise, Ali Jawad which is the co-director of Accessercise and also Doctors David Maidment and Daniel Rhind uhm I will briefly go through uhm the ethical considerations for this study so you have read through the information sheet and you have signed the consent form so you know about the study uhm any information that you give me which is sensitive will be confidential so when I transcribe the interview uhm you will be given a pseudonym so a random name so no one will ever know who you are and nothing will be linked back to you uhm if at anytime you want to withdraw from the study please feel free to let me know uhm you do have the right to withdraw if you do not want to participate anymore uhm but just please feel free to let me know uhm and within this interview there are no right or wrong answers uhm and do you have any questions that you would like to ask before we continue with the interview process?

P11: No.

R: Perfect, ok so the first question I have got is uhm please may you provide the following information so your name, age, gender identity, ethnicity and county of residence?

P11: Uhm, Stephen Gibson uhm fifty-eight years of age uhm what was the next question?

R: Uhm, gender identity?

P11: Male.

R: Perfect! And your ethnicity?

P11: White Irish.

R: Perfect! Ok, and your county of residence?

P11: Sorry, what did you say?

R: Your county of residence?

P11: Oh, Buckinghamshire.

R: Awesome! Uhm, so to start could you please tell me how you first got involved with the Accessercise application and why?

P11: Uhm, I saw a note about it on the SIA website.

R: Ok.

P: Uhm, the Spinal Injury Association uhm and yeah I thought I would reach out and make contact uhm it seemed like a good one to start with uhm in particular I was interested on how universities do these studies uhm because I’m interested in contributing and take it from there and an app is a modern way of doing things.

R: Perfect, ok! So, this question uhm I don’t think you will be able to provide too much information uhm but could you please tell me your experience of using Accessercise and how long you have been a member?

P: About thirty minutes haha.

R: Haha, so not very long then?

P: Nope haha.

R: That’s fine, so uhm we will now go into the main Accessercise specific questions so do you currently use Accessercise in your role and if so when and how uhm so for example uhm if you were to use it when would you use it and why?

P: So, I don’t currently use it uhm because it’s the first day of me having it uhm I think it’s something that if it works for me I will try and imped it into my life so uhm a bit like you know uhm you will look at your diary to find out what meetings you have to go to uhm I will have it there as part of that uhm telling me that I need to get up and do these stretches before I set away in my day that sort of thing uhm so I’m going to try and imped it into my life.

R: So, do you think it’s something that when you go to the gym you will like use when you are there to monitor your activity levels?

P: Yeah, it would be good to have everything in one place because you know for me I will get the hydro therapists provide me stuff uhm the NHS neurorehabilitation guy offer me stuff and uhm my one to one at the gym uhm and bringing it all together plus the stuff that I do at uhm.

R: Right.

P: Uhm, it would be good to bring it all together into one place and have access to videos and you know make sure you’re doing it right.

R: Perfect, ok. Uhm, so obviously showing you the app and giving you the insight about what is currently on the app uhm what were your initial thoughts when you first started using the application uhm did anything stand out that you liked or you didn’t like?

P: Uhm, apart from sort of looking at it uhm it’s quite hard to tell but I think that will come more as I begin to play with it uhm because you will be able to identify strengths uhm limitations uhm things that work for me uhm and also things that don’t work for me.

R: Ok, so in terms of designing your workouts uhm so I showed you the videos uhm the key descriptions of each exercise uhm did you like that function uhm did you like the option to watch videos of each exercise?

P: Yeah, it all looks good especially each video as it’s tailored towards the disability but also the description of each exercise which helps if you’re unsure.

R: Perfect, ok! Uhm, so obviously uhm you haven’t got too much experience of using the app but do you think from anything that I have showed you so far could be improved or anything that you would like to see from your own perspective that you would like to be included on the app?

P: Uhm.

R: So, obviously there are so many different types of apps out there but there are not many specifically for disabilities but is there anything that you would really want from your own view that could be included?

P: Uhm, probably the option to customise the app to what I do uhm one thing that I have learnt about spinal cord injuries uhm is that we are all unique uhm the way that it affects us uhm how badly it affects us uhm and the impact it all has on our bodies uhm so because of that sort of environment of uniqueness I think it is quite key for spinal injury people to be able to modify the app uhm tweek it uhm be able to add things in that they use in their every day life uhm that will help them to do it.

R: Ok.

P: Uhm, you know there might be things on there but it might not be under the term that they use or whatever.

R: So, going back to what you said about everyone with a spinal cord injury being unique and different uhm I agree ugh do you like how this Accessercise app is very much focused on specific disabilities and how you can filter the workouts based on that.

P: Yeah, that’s a good start uhm like I said it’s really getting into it uhm whether you have got motor neuron disease uhm cerebral palsy uhm spinal cord injuries uhm lost a leg or arm or whatever uhm it’s really you know uhm all those individual things uhm it’s finding what is individual to you and searching down into the details of it and how we can use it to build something that is specific to what you really want.

R: So, you like that function?

P: Yes, any app that focuses specifically on my disability is so valuable.

R: Perfect, ok. Uhm, so do you use any other similar applications outside of Accessercise and if so what is better or worse than Accessercise?

P: Uhm, so I have a physio app.

R: Oh, ok.

P: It is popular with the uhm physiotherapists uhm hydrotherapists ugh the neurorehabilitation people uhm it seems to be a pocket leader with them all.

R: So, would a physiotherapist provide you uhm with uhm exercises to do?

P: Yeah.

R: And how do you find that app uhm is there many differences between that and Accessercise?

P: So, that is one that they provide you exercises.

R: Ok.

P: Have you used the app called physio?

R: No, I have never used it before.

P: Uhm, so they give you a set of exercises.

R: Ok.

P: So, in some ways it’s similar because it comes along and it will have what it is uhm what it’s supposed to do and then there will be a little video.

R: Oh, ok.

P: It will show you how to do the exercise and you can then mark it as complete for the day.

R: It actually looks pretty good.

P: Yeah, so it has that sort of thing.

R: Yeah, it’s pretty good.

P: However it’s not very customisable by me for me and I do not load in loads of data about me.

R: Right, ok.

P: They basically say right uhm in your one to one with your physiotherapist uhm these are the exercises they recommend and then the exercises are added to the app so you have that reference point.

R: But do you think from what your experiencing of using that app uhm there is anything that could be brought along to Accessercise that could maybe benefit that app further?

P: Uhm, yes.

R: I like how you mentioned about ticking off exercises for the day.

P: So, what would be logical is to have uhm you know your physiotherapist or hydrotherapist uhm so your sort of healthcare professionals be able to input stuff into Accessercise.

R: Right.

P: To say like uhm right this is what I want you to do uhm these exercises.

R: Right, ok.

P: Now whether it pulls from what is already in there or it adds in something specific uhm from your medical professional.

R: Right, uhm so you are saying uhm having a physiotherapist support you on the app and providing you with regular exercises?

P: Yeah.

R: Right, ok. That sounds like a good idea! So, in terms of like uhm the function uhm where you can tick whether you have a carer and maybe like a carer there uhm do you expect anything more from that uhm is there anything you would like from that setting uhm for example could there be videos for the trainer on how to support the individual do exercises at the gym.

P: That would seem like a logical thing to do.

R: Yeah. So, for example like where they stand uhm so like if you’re doing a set of exercises uhm obviously you will know how to do it.

P: Ugh and how it relates to your objective would be important and all that sort of stuff.

R: So, like videos for the trainers?

P: Yeah.

R: Yeah, that sounds pretty good. Uhm, right so we are now going to move onto the main protocol so what I’m going to get you to do is log onto the app again.

P: On my phone?

R: Yeah, so uhm the purpose of ugh the think aloud protocol is uhm yeah so the instructions for the think aloud protocol are straight forward uhm I would like you to say out loud whatever comes to your mind so there are no right or wrong answers uhm we will quickly run through a quick practice to get you used to the protocol so could I get you to say out loud everything that comes to your mind when you think about physical activity?

P: Sorry I got distracted.

R: That’s alright.

P: So, what am I using this for in terms of answering these questions?

R: Oh, so I’m just giving you an overview so keep the screen like that for now uhm and I will go through it again uhm so the instructions for the think aloud protocol are straight forward so for this interview I would like you to say out loud whatever comes to your mind uhm so there are no right or wrong answers here for so for example I’m just going to quickly go through uhm a quick practice run so uhm can I get you to say out loud everything that comes to your mind when you think about physical activity uhm so what key words come to your mind?

P: Uhm, so repetition uhm strength ugh stamina ugh tone uhm fitness.

R: Perfect, ok.

P: And movement.

R: Awesome, ok so from your screen uhm you have obviously logged onto the app which is great uhm so obviously what you see on your scree now are the first things you see uhm but what are your first impressions when you see this uhm do you like the way that uhm all the exercises and workouts are laid out one after each other uhm do you like the colour of the app?

P: Yeah, the colour is good uhm it’s not offensive uhm it doesn’t bother me uhm because it’s clear uhm I can see what’s representing uhm it’s responsive with the videos.

R: So, in particular about the videos uhm what specifically do you like about the videos that stand out for you?

P: Uhm, the videos are good because you can see how the person is using the machine uhm with their arms in this case uhm so you are trying to basically repeat the action.

R: Ok.

P: I think that’s important so that they can achieve their objective.

R: And do you like the audio descriptions?

P: Yes, the audio descriptions for each video are quite important.

R: Ok, and in terms of the person doing the exercises do you like how it is very much based on the persons impairment so uhm for example if you were to choose let’s say an amputee as your impairment uhm you will then see someone in the videos with an amputee doing the exercises uhm do you like how the app tries to relate to the individual?

P: Yeah, I think that is the next step on this sort of fitness apps uhm in terms of uhm being able to present them with exercises that are appropriate for the challenge uhm I think that’s a key part.

R: Right, ok.

P: And then the more that can be refined to whatever their objective is uhm you know an amputee has got a leg missing uhm is it based around building up the strength in the stump uhm in order to work with the prothesis uhm another example is spinal cord injury uhm how does it focus on getting the ugh where the signals interfere to the muscles to learn how to work again.

R: Yeah, makes sense.

P: And focusing on that and those sort of things and the more detail you can go into with the more specific options uhm I think is really the art of this uhm because I have seen many apps and they all sort of start off with clunky bits you know.

R: Haha, yeah, I know what you’re saying.

P: Uhm, getting them to develop and build a bigger database over time really.

R: Yeah, that makes it helpful. Uhm, in terms of the audio descriptions uhm not the audio descriptions but the actual content on the exercise uhm so do you think the written text at the bottom uhm do you think is uhm a bit too much information uhm what comes to your mind when you see that?

P: Uhm, I think the description of the exercises is quite good uhm because you can also see what parts of the muscles the exercises are targeting.

R: So, you like the key muscle component section?

P: Yeah, I do like that uhm this section has clear instructions on how to do the exercises which is very helpful as it all matches the content in the video uhm so you know where to put your feet or something uhm you can actually see that which really brings everything together.

R: Yeah, do you think they could possibly bullet point all the written information they have about each exercise itself uhm do you think they could make it less clunky in terms of uhm bulky text?

P: Uhm, it’s hard to say really.

R: Ok.

P: A lot of it comes with actually using the app and having a go uhm so the actually answers I will give you in two- or three-month’s time will be completely different to now.

R: Yeah, that makes sense yeah. Uhm, if you scroll to the bottom uhm what about the useful tip bit uhm do you like the useful tip?

P: I think it’s good that they have got the breathing tip.

R: The breathing tip?

P: Yeah, I’m always forgetting uhm to breath uhm when you’re moving a weight uhm so for example when to breath in and when to breath out.

R: So, you like the fact that they have added that option on to remind people?

P: Yeah, the key benefit option that accessercise have considered including.

R: I think what I like is that they have put the tip in blue so it sort of stand outs.

P: Yeah, the choice of colour here for the key benefit box is good.

R: Uhm, yeah I think that’s quite nice and the muscle component bit is pretty good uhm so if we go back on the app uhm so we have done the videos uhm we have done the description uhm how about the social section in the bottom left corner uhm so if you press the social uhm what comes to your mind when you think about the social section and how you can follow people on this uhm and you can see their workouts?

P: Uhm, what do you mean by following?

R: So, here you can see that you follow liv.

P: Oh, ok. It’s sort of bringing the social media twist into the app really uhm and how you can upload a picture and thumbs up uhm and heart and whatever you want to do haha.

R: So, in terms of the social section uhm not everyone is going to use it but do you think it’s a good feature to have on the app?

P: Uhm, I can see how it can have some benefits uhm I expect that it won’t be for everybody uhm these sort of things don’t always excite people and some people you know extroverted which theoretically should be into it uhm where introverts maybe not haha.

R: Haha, it depends on the people I guess.

P: Yeah, uhm but I like how it has got that function because uhm sometimes people like a bit of interaction with others and so on.

R: Yeah.

P: Uhm I think it’s good to help people and remind them that they are not alone in this uhm and potentially you know uhm how can people build a little network of people on this where they share similar characteristics to you and interests uhm sort of like community groups that you get these days.

R: Yeah, makes sense.

P: So, yeah, those sorts of things.

R: Do you like how you can react to others workouts and uhm you can leave a comment uhm do you like that sort of function?

P: Yeah, uhm I’m presuming that sort of function is managed uhm because I can imagine people fall out and say stupid things uhm so I wonder whether there’s a certain way of controlling that uhm.

R: I assume that it’s got some sort of function to control for that uhm and monitors each page uhm so any offensive content is automatically removed.

P: You would think so because that would be quite important.

R: So, uhm if you press the find group option uhm so in terms of groups uhm would you like the idea of maybe doing exercises in groups or having videos of exercises completed in groups uhm for example yoga uhm what would be a good idea is following a yoga group but also having videos of how to do yoga in a group.

P: I think it’s good uhm and people can either create or join groups that are relevant to them uhm what they are trying to achieve and their particular challenge uhm and it’s down to you know how they use them and how they let people join uhm you know how they set the rules uhm what they are about sort of thing uhm it’s the same sort of thing you know that people over the last ten years of all spent time learning about uhm in terms of social media uhm because it has a good side but also a dark side you know haha uhm like everything in life really uhm and it’s important to know how to manage these things.

R: Yeah, I agree. Uhm, in terms of groups would you like to follow individuals with a spinal cord injury uhm would you like it to be really broad uhm in terms of fitness groups or people with a similar interest?

P: Uhm, I think with this sort of thing it’s sort of like why am I here oh because I have a spinal cord injury uhm if I didn’t have a spinal cord injury I wouldn’t be using such apps uhm like a lot of things in life uhm so it’s sort of driven by that and I’m using it because I sort of want to improve myself in someway from where I am today.

R: Yeah.

P: Uhm, so any groups that you join or things that you get involved with have to support that uhm and it has to recognise uhm you know because as well as having this thing which is quite dominating in life I have to deal with others things as well uhm you know things around the house uhm house jobs because my wife has gone to work uhm and I’m out there doing as much as I can.

R: Of course.

P: Uhm, so yeah there we are.

R: I think it’s cool to have those options uhm because I think when it comes to physical activity uhm especially during COVID uhm you don’t always want to do exercise uhm you want to be a social support network uhm so having the option to do some forms of exercise in groups is great uhm especially during COVID because it was quite a lonely experience uhm it’s good to have.

P: Yeah, right.

R: Uhm, I’m not exactly too sure what groups they focus on when it comes to the Accessercise app but uhm it’s definitely something that they can build on.

P: Yeah, I’ll have to join some uhm you know and stick my toe in the water and see what it’s like.

R: Haha, I think what is good with the groups is whether that can link to the podcasts uhm and the blogs uhm for example so if you join a spinal cord injury group uhm could they provide then regular blogs for people with spinal cord injuries on how to do certain exercises uhm and working with like minded individuals uhm so I think that is quite good but uhm in terms of the notification option uhm so the little bell on the top right corner uhm would you like to be notified on when to do exercise?

P: So, that could help if I was notified when to do exercise so a little nudge.

R: So, like a little nudge so for example uhm like an alarm on when to complete your daily workout.

P: It can sort of be helpful uhm or it can quickly become very annoying.

R: Haha, yeah.

P: Uhm, so having one of these sort of apps yes uhm I think you should have notification options uhm because some people live very organised lives uhm and they love it uhm whereas others don’t enjoy that sort of thing as much uhm but you will probably need a bit of it to provide some structure to your exercise routine when it comes to dealing with these sort of things but you know it may not be their favourite part of it.

R: Do you think for example uhm that comes down to motivation uhm so the notifications could help people become more motivated to do physical activity uhm do you think the idea of streaks is quite a good idea?

P: Streaks?

R: Yeah, so for example if you use the app ugh for three or four days in a row it will say well done you have used the app for three or four days in a row so you are constantly maintaining a scoring system uhm so after thirty days uhm it will say well done you have used the app for thirty days uhm if you don’t use the app tomorrow you will lose that streak and that streak can then lead to awards and so on?

P: Ugh, yeah I think having a long-term streak on the app would support me a lot.

R: So, for example I think if you were to use the app every day for a whole year uhm you are going to want to use the app the next day because you don’t want to lose that streak uhm so it’s that continuous effort you have put into the app.

P: Yeah, it’s a bit uhm you know if you use it every day for a year uhm your body is going to want to do this stuff everyday uhm so yeah I think streaks do generate some sort of positive feedback uhm and I think that recognition of using the app a lot is a good thing uhm for people who have done something uhm who have achieved something uhm maintained something uhm whether that’s gained muscle uhm lost weight uhm kept their blood pressure down uhm walked more steps uhm it's all a part of it uhm you know uhm yeah so if you want to build one of these apps you have to consider these things.

R: What was your thoughts on maybe some competition on the app uhm so for example you have that social section in the bottom left uhm where you can follow people but do you like the idea of maybe uhm having leader boards or uhm I don’t know like some sort of competition there.

P: Uhm, to be honest I don’t really get excited for that sort of stuff.

R: Ok.

P: However I can imagine other people you know loving it haha.

R: Yeah, makes sense.

P: Uhm, so that is very much a personal thing uhm you know.

R: Uhm, in terms of uhm actually if you press the little person in the top left uhm do you like the fact ugh so this is your profile uhm so for example you can add a photo uhm people can see your achievements uhm they can see who you are following uhm what comes to mind when you see this page uhm is there anything that you like or dislike about this?

P: Uhm, it seems like a normal sort of thing from social media uhm you know you have a picture of yourself uhm you can have some words in there about yourself uhm who you are and what you are trying to do uhm your achievements uhm will I go and put loads of milestones in there with dates you know uhm and tick them off uhm probably I’m not sure.

R: Yeah, makes sense. Do you think there is anything else that is good?

P: Followers, yeah you know uhm in terms of social media the people I follow are normally people that I know outside uhm offline you know.

R: Ok.

P: Uhm, there are a very few amount of friends that I have met online.

R: Right, ok.

P: Uhm, so.

R: I think for this for example if you were to join a group at the gym and people wanted to use this app uhm and the app became more familiar with people then you would want to follow people that you go to a class with on a Friday uhm just to check up on them uhm they can check up on you.

P: Uhm, so it would be more about building a spinal cord injury network of friends uhm I suppose that would be interesting especially for those with a traumatic injury uhm.

R: Uhm, so yeah having those groups uhm having those communities is important uhm but do you think there is anything more that they could add to ugh the profile page for example uhm goals uhm it would be good to see what specific goals the individual has uhm or even maybe what impairment you have uhm so if we were to follow Liv for example uhm it would be good to see uhm you know what specific impairment does she have uhm if she wants to provide that information.

P: It’s important that they are given the option to provide how ever much information they would like uhm like I said some people will be all out there uhm and they will go for it uhm and others may not like doing that.

R: Yeah, they may find it too much.

P: Uhm, they may even struggle to put their real name on there haha.

R: Haha, yeah that’s true.

P: They may even put a picture of their cat haha.

R: Haha, so if we go back on the app for a second uhm so if you press the back option uhm we will now go to the explore section which is the third option on the app uhm so what was your initial thought when you saw the explore section?

P: It’s taken me to the Atlantic ocean.

R: If you scroll up and go to the UK.

P: Yeah, haha that’s kind of annoying.

R: So, what’s your initial thoughts when you see this explore feature and the fact that it’s linked in with google maps and it’s got those accessible gym options uhm and you can see all about the gyms uhm does anything stand out to you that you like?

P: Well, I think if the app had rehabilitation options available would be good uhm but the explore section on Accessercise is good to see what is available around you uhm how accessible the buildings are uhm and going back to my point it would be good if the app can also include rehabilitation facilities uhm NHS options and not just gyms.

R: Ok, so in terms of this uhm so this is specifically looking at gyms uhm but do you think having like particular sport clubs, venues uhm would be good if that was added?

P: Yeah, if accessercise can include rehab centres, sport opportunities would help.

R: Uhm, and not just all about gyms?

P: Gyms, swimming pools, rehabilitation centre options on Accessercise would be a really important feature to include.

R: Ok.

P: I use a swimming pool in Cranfield uhm just to go swimming in uhm at the hartwell facility uhm and that is a hot pool uhm like thirty degrees which is great to get the muscles going uhm now the reason that it is thirty degrees is to teach babies and toddlers how to swim.

R: Right, ok.

P: Yeah, bringing them into the water uhm that is why it’s so warm.

R: Haha, yeah.

P: Uhm, and then they do a few sessions for people like me who turn up and need to get in the warm water pool.

R: Yeah.

P: Uhm, so those sorts of facilities and having them available would be good.

R: So, you would like uhm not just gyms but a range of different physical activity facilities?

P: Yeah, so like hot swimming pools, uhm sport clubs uhm hydro therapy centres, uhm.

R: And not always just gyms?

P: Yeah, because it’s hard to find certain facilities, so if this app can include that as well as gym that would be great.

R: Yeah, it would be good if they can add more and not just gyms uhm because not everyone wants to go to the gym uhm some people just want to join a local sports club uhm that is an issue uhm because if someone wants to join a uhm wheelchair basketball team uhm it’s impossible to find.

P: So, as you can see on my phone that pink place is the neurorehabilitation centre.

R: Right, ok but it would be good to have an option there where you press on it and more information comes up.

P: Uhm, it would be because a few people that will be using this app will be going there.

R: It would be good to have that and not always focus too much on gyms.

P: Yeah, uhm having a full range of ugh facilities to support people who are uhm under a form of rehabilitation uhm related to you know uhm what we are going through uhm and you know there’s different groups so for example uhm there are people like me uhm I have had an injury uhm I have had it dealt with uhm I won’t get worse uhm because of that injury the question is down to uhm how much better can I get through doing all this stuff uhm people with cerebral palsy uhm motor neuron disease they get worse uhm that’s the prognosis uhm there is nothing you can do about that uhm the question is how can they stretch it out uhm and maintain as much capability for as long as they possibly can before it finally goes away uhm and it’s a different approach uhm but they will all be involved in these sort of centres and things.

R: Yeah, I agree with you uhm I think if I was to use the app I would like to see uhm where the local wheelchair tennis clubs are uhm where are the local wheelchair rugby clubs.

P: Yes, the map function needs to include more things and not always about gym.

R: And you have the option to contact them and you can say like hello uhm do you have any availability and it is really tackling the issue of limited advertisement for disability sport.

P: Yeah, I know what you’re saying uhm so where do you go to get your wheelchair serviced uhm repaired or whatever uhm in the SIA groups you can see it happen all the time where people say oh this has gone wrong with my chair uhm and that sort of stuff and the support is limited.

R: Yeah.

P: It very much all comes down to word of mouth you know uhm actually these days it’s more of a digital word of mouth.

R: Haha, do you like the fact that you can search for facilities by putting in your post code and having those options come up uhm in a listed format?

P: Yeah, I do like that option uhm that is pretty good uhm yeah it’s really good uhm from my own perspective.

R: Yeah, it’s good ugh the listed option uhm and it’s also got the map version uhm so the explore section on the app is that something that you really like.

P: Yeah, the explore section is really helpful and something I would definitely use.

R: So, uhm for example if you were to zoom out and were to find a gym uhm let’s actually look at the gyms itself uhm so if you keep zooming out and you press on this one here uhm is there anything that stands out to you that you like here uhm is there anything that could be improved uhm or any strengths for this feature?

P: Uhm, a bit more information about the gym possibly uhm even possibly a video clip showing you what the facilities are like uhm the machines would be a good thing to have uhm so for example does the facility have a swimming pool.

R: So, sort of like a three sixty rotation of the gym?

P: Yep, the idea of a three sixty rotation of each gym would be cool to have.

R: Yeah, that would be pretty good.

P: Even like a picture of the entrance so you can gain an insight of what the gym is like before you use it.

R: Yeah, I think that is good because if you spend a good amount of time going to that facility uhm and you can’t see what it looks like uhm that won’t be very helpful.

P: I agree.

R: So, yeah have a rotation uhm but in terms of the block of information there uhm so as you can see the opening hours uhm the phone number of the gym uhm is that something you like?

P: Yeah, no that is all the basic stuff you need like uhm the address, opening hours uhm it’s got the phone number so you can ring them up.

R: In terms of the rating system uhm would you like to see reviews there uhm would you like anything to stand out uhm maybe a messaging service to the gym uhm is there anything that the gym can do to support that option on the app further?

P: Uhm, yeah the idea of having a link for each gym would be a good feature to have uhm so you’re directed to the gyms website uhm most of the information here like the phone number, opening hours they will already have on the website uhm and I think the web page link would be the best option to include uhm you can click and you’re directed to the gyms web page and you can easily find all this information.

R: Ok.

P: Uhm, yeah probably the link to the website for the gym would be helpful to have on the app uhm for me the big thing missing is the web page for the gym.

R: Ok, so you think having the actual link to the gym would be good.

P: Yeah.

R: Ok.

P: Uhm, because they will already have all the information you can see here on the website anyway uhm and it will be easier just to have a link that users can access.

R: Yeah, that’s a good point.

P: Haha.

R: But in terms of the rating system with the stars.

P: Yeah.

R: Is there anything that you would like to see more from that side?

P: Uhm, I would like to see an accessibility review uhm also how the rating was worked out uhm you know what are the attributes to give the gym a five star rating uhm as opposed to a four or a three or a two or a one uhm what does it mean in a real world.

R: Ok.

P: Somebody like me will go there and think it’s great but for a wheelchair user may think it’s terrible.

R: Haha, yeah. So, maybe the option of breaking that review system down would be a good option to have.

P: Yeah. Maybe that is something to say uhm the person giving the review uhm had these limitations uhm found it very useful uhm because you won’t be going along there necessarily and would have been trained on how to assess a place as a wheelchair user or anything like that.

R: Yeah, I know what you mean.

P: One person gives five gold stars to a restaurant because he’s best mates with the owner haha uhm and another person goes and finds it awful.

R: Haha, I agree with what you’re saying. So, it’s got that function which is great uhm but yeah I think just including more and understanding what the two mean uhm.

P: Yeah, like right here as you can see is my gym.

R: Right, ok.

P: And that’s Nuffield uhm but there’s no reviews there.

R: Yeah, that’s something they could add uhm more stuff that they could work on uhm you know there’s different ways that they could improve it.

P: Yeah, it’s definitely under the review phase I guess.

R: Uhm, so if you go back onto the app.

P: However, I’m assuming that they would need to get permission from the gyms to put it on.

R: Yeah, that’s the problem but I’m sure that there is something they can do to make that happen.

P: Haha, that would be good uhm right what is next.

R: So, the last one is uhm so if you press the more option in the bottom right.

P: Ok.

R: Yeah, that is it. Uhm, in terms of the shop, nutrition, blogs, podcasts uhm do you like those functions uhm what stands out for you from that uhm so obviously they are coming soon uhm but is there anything in particular that you would like to see from those options?

P: Uhm, it would be good to have some fitness items on the app like buying a t-shirt, but I probably wouldn’t buy it haha.

R: Haha.

P: Uhm, you know doing nutritional plans uhm doing their own branded you know.

R: T-shirts, bottles, hats and so on?

P: Yeah, weights and all of that carry on I don’t know uhm.

R: Uhm, in terms of nutrition is there anything in particular that you would like to see from that?

P: Uhm, so the shop I think uhm every one of these apps has a shop option uhm they normally have something to tell you which is branded in their own way uhm nutrition I think it should be there because of what it’s all about uhm but again what is best for me uhm what is best for an amputee uhm a person with motor neuron disease is completely different.

R: So, do you think have nutrition options based on the users impairment for example a spinal cord injury?

P: Well, yeah because you are mainly focusing on impairments on this app uhm that is the reason people have it not because they ugh want it as a new social media site for example having a bunch of people with a similar injury uhm.

R: Yeah, makes sense.

P: You know this is more of a tool rather than a social media option uhm but it has brought that option into it.

R: Yeah.

P: So, yeah it has to have something in there that is related to what I am and where I am at.

R: Uhm, yeah, I understand. In terms of blogs, vlogs, podcasts do you like that?

P: Uhm, yeah you know uhm would I use them uhm probably not because I cannot imagine myself doing it uhm you know it depends on who is there talking about spinal cord injuries uhm for me to get interested in it uhm you know do they have an interesting voice uhm are they interesting uhm will they actually make me want to listen to them.

R: Yeah, I understand.

P: Or read what they put on there uhm you know some people will be well into it uhm and then others will you know uhm run a mile haha uhm and podcasts are exactly the same you know.

R: Yeah, there is so much more that they can do.

P: You know whether it becomes justifiable for them in terms of people creating content and ensuring people watch it and listen to it uhm you know only tell will tell about that.

R: Yeah, I know what you’re saying.

P: Yeah.

R: So, we will go onto the overall thoughts so the last few questions uhm so from seeing the app and using it and providing some background information regarding it uhm.

P: Uhm, just to butt in and saying something regarding that uhm so would you have people like the spinal injury association working with the app?

R: I believe yeah they are included so like amputee UK, dwarfism UK have been contacted by the developers.

P: Yeah, so I think you really need to ugh because I think what you are trying to do is as well as having all the ugh the very fitness and health focus stuff uhm this sort of stuff is the social media side and you really need to bring in the whole community uhm so it’s the swimming pools uhm it’s the ugh other neurorehabilitation centres uhm it’s the spinal cord association uhm and so on you know the environment and the support groups for the relevant things you are looking for.

R: Yeah, that’s a good point uhm that’s a good idea.

P: Uhm.

R: Yeah, I agree with you because I feel like the co-end users and the people that are developing the apps uhm the people that are getting involved in the apps so the physios uhm should always have an opinion and base the app of their experiences and flaws uhm so yeah I agree with you on that.

P: Yeah.

R: Uhm, so one of the last questions uhm I have got is uhm what are potentials positives if there are any of using Accessercise for improving physical activity levels?

P: Uhm.

R: Are there any positives that you think uhm?

P: Uhm, by bringing everything into one space like the app has done uhm uhm it’s like the Facebook of the exercise world uhm you know it makes life a lot easier.

R: So, if you had to name three strengths of using the Accessercise app uhm so from all the different functions you have seen uhm what three things really stood out to you that you really liked?

P: Uhm, so all the things that we looked at uhm so this social media area for a better term uhm as it doesn’t exist for a start is hard uhm to say uhm but in principle yes uhm I can see what they are poking at and yeah it could be a really good thing uhm but you have to really get into it uhm to see what it’s like uhm.

R: Ok, and a final strength?

P: Uhm, I think allowing people to interact with it uhm and you know tailoring things for each individual and their impairment and challenges uhm that’s it really.

R: Ok, perfect! Ok, uhm so let’s move onto the final question uhm so what are the potential negatives if there are any of using Accessercise for improving physical activity levels?

P: Uhm, I would say uhm the workout section and stuff I don’t really see a negative side to that uhm you know I showed you physio app uhm it’s all doing similar things uhm in terms of the social media side of things uhm the groups and that sort of thing uhm there is the usual sort of crap uhm you know on social media where people are slagging each other off and things uhm and lying and all sort of stuff you know uhm that has to be managed and you know monitored and you know people kicked off the app for misbehaving and that sort of thing.

R: Right, ok. So, the social media section and monitoring bad behaviour.

P: Yeah.

R: Ok, so last question uhm is if you had to give uhm three areas for improvement uhm how do you think the Accessercise app could be improved if anything for it’s use on improving physical activity levels uhm so you have seen the app uhm so far uhm you have seen what it’s functions are uhm is there anything that you think really should be added to it to make it the best that it possibly can be?

P: Uhm, I don’t know it gives you an electric shock every time that you struggle to do things on time haha.

R: Haha.

P: Uhm.

R: How about like streaks, competitions uhm you know uhm.

P: I find it hard to get into competitions because I’m not that kind of person uhm you know I’m here to do what I need to do for me uhm you know I can compete against my own milestones uhm I’m not going to be a weight lifting champion uhm so I can see for some people that you know uhm it would motivate them and all that sort of stuff uhm they would love the sound of the crowd.

R: Uhm, in terms of the colour uhm the visuals uhm anything you think they could improve on that?

P: Uhm.

R: Even the language that they are using?

P: It’s all clear and easy to pick this app up and use it even if you’re a beginner

R: Ok, haha. Is there anything else that you would like to add or discuss before we finish?

P: No, I think that’s it really uhm we have covered quite a lot haha uhm some good things for you to chew on.

R: Awesome, thank you so much Stephen for taking part.

P: Oh, the other thing I wanted to add is uhm how would you stop people trying to use it as a platform to sell things to people uhm which may or may not be authorised?

R: Uhm, it’s a true point but I think in terms of the social media section it’s not too much that you can really do uhm how do I explain uhm you can follow users and comment on their workouts but someone I’m sure will be regulating that section on the app to see and avoid anyone trying to sell anything and violating the terms and conditions of Accessercise.

P: You know somebody has recommended somebody does something which is not appropriate for them uhm.

R: Uhm, yeah that’s true yeah.

P: Yeah.

R: Yeah, it’s something to think about I guess but I’m sure the app developers have thought about this and you would like to think so haha.

P: Yeah, it would be good to see if they think about that.

R: It’s an interesting point but yeah thank you so much for taking part in this interview I appreciate that.

## Transcript of interview undertaken with Participant 12

**Key code:**

R: Researcher

P12: Participant 12

R: Awesome, thank you Adam for willing to take part in this study and coming here today to ugh get involved in our accessercise research study so what I will do first is I will go through an overview of the interview process so I will go through who I am so my name is James Haley and my role within this research is that I’m the lead researcher so I’m responsible for collecting the data, analysing the data and also writing the study up uhm the aims, importance and purpose of undertaking this research project and the benefits of participating is that we are looking to see whether this app can improve various health outcomes such as pain, fatigue uhm physical activity ugh quality of life and so on but also as a part of this interview we would like to see what the strengths, weaknesses and also areas for development for the app so the developers can improve the app further ugh the benefits of participating is that you can support us identifying any gaps uhm of the app so that the developers can improve it but also you can see things like your own pain, fatigue, physical activity levels and whether they have improved as a result of participating in this research uhm the university that I attend is Loughborough University in the East Midlands of England uhm my position is that I’m a Doctoral Researcher based within the Peter Harrison Centre for Disability Sport which is within the faculty of Sport, Exercise and Health Sciences ugh the other individuals that are working on this project is Sam Breary which is the director of accessercise ugh Ali Jawad which is the co-director of accessercise uhm but also Doctors David Maidment and Daniel Rhind which are my primary and secondary supervisors uhm I will briefly go through all the ethical considerations in this study so you have read through the information sheets uhm you have signed the consent forms which is great uhm any information that you provide will be confidential so the written work you will be provided a pseudonym so in other words is a fake name so no one will identify you as the person in the interview uhm and you also have the rights to withdraw from this study whether you feel like you need to uhm and lastly there are no right or wrong answers in this study do you have any questions that you would like to ask before we continue with the interview process?

P12: Nope.

R: Perfect, thank you so the first question I have got is uhm please may you provide the following information so your name, age, gender identity, ethnicity and county of residence?

P12: So, Adam Dickinson I’m forty two uhm male.

R: And your ethnicity?

P12: Ethnicity uhm White British.

R: And your county of residence?

P12: It’s Leicestershire.

R: Perfect, ok so Adam could you please tell me how you ugh first got involved in the accessercise application and why?

P12: Uhm, the truth is because I’m helping you out uhm yeah but I’m interested to see what the app is like as well.

R: Perfect, ok.

P12: Accessercise seems to be useful.

R: And do you have any experience of using any previous physical activity apps?

P12: I have used uhm couch to five k before.

R: Ok, and what was your experience like using that?

P12: It started off well and then it dipped after a while.

R: Is that because of your motivation?

P12: Yeah.

R: Right, ok so you feel like one of the issues with using apps might be motivation?

P12: Yes.

R: Perfect, ok uhm in terms of accessercise is it something that you have seen advertised around uhm I know obviously you have seen it because of my study?

P12: I have seen it on Twitter quite a bit.

R: Right, ok uhm have you seen it anywhere else advertised?

P12: No, just Ali tweeting about it.

R: Perfect, ok and why is a physical activity app something that you want to get involved in uhm is it something that you think will help you in terms of reducing barriers to physical activity?

P12: Yeah, I think in terms of being able to workout where accessible gyms are is a really good function on this app.

R: Right, ok so you like the explore function?

P12: Yeah, yeah uhm because it’s difficult to find a gym that is accessible so it’s useful for that and also just to give you that push into doing exercise.

R: Perfect, ok based on your experience of using couch to five k what would you say are some of the differences between this app and the one that you have used before?

P12: I think couch to five k gave you more sort of like feedback I suppose yeah and telling you when you need to do it.

R: Ok.

P12: Uhm, that’s because you set like a reminder and it basically told you what time you need to do it uhm.

R: Right, ok so you mentioned about that and that’s a point that I have identified about the app uhm that there’s no real time feedback uhm would you like there to be a function where you can see like some statistics and some graphs about how you have developed?

P12: Yeah, I think you need that to show that you’re actually achieving something.

R: Right, ok yeah uhm do you like how on the app you don’t need to put in your height and weight because I know someone with let’s say dwarfism because of their height and weight the app might find it incorrect.

P12: Yeah.

R: Do you like how it’s very much focused on the disability?

P12: Yeah, I like how the accessercise app is tailored for different disabilities and stuff yeah.

R: Ok, that’s perfect! Awesome, uhm so obviously this is a trick question but could you please tell me your experience of using the accessercise app and how long you have been a member?

P12: I have been using it for the last day.

R: Perfect, ok.

P12: I’m just going through it and seeing what it’s about.

R: Perfect, ok and so you mentioned about the explore function but is there any functions you thought stood out to you that you liked uhm so maybe like the videos or the content of each exercise?

P12: The videos are really useful uhm it’s difficult to know how to exercises when you’re in a wheelchair so it’s good to have a video showing you how to do it.

R: Perfect, ok.

P12: In the past I have had just like pictures from physiotherapists which doesn’t really tell you how to do it so the videos are a really good function.

R: Do you find that sometimes uhm obviously being a wheelchair user the videos the physio’s provide you are images of a more abled individual instead of someone in a wheelchair?

P12: They tend to just be someone sat in a chair yeah.

R: Right, ok.

P12: Like uhm a school chair sort of thing.

R: Right, ok and do you find from your experience that might be a bit of a barrier?

P12: Yeah, because you sort of want to see yourself in the videos yeah.

R: Ok, so you like that the app is tailored towards the disability but it also has videos which you find useful?

P12: Yeah, the fact the videos are tailored to individual disabilities is really good.

R: Perfect, ok that’s great uhm it’s nice to hear that and do you think if you were to use the accessercise app it would be something that you would use at home or do you think you will use it at the gym?

P12: I think probably more at home.

R: Ok.

P12: Getting exercise done at home uhm.

R: Is there a specific reason behind that?

P12: It’s because gyms are really expensive.

R: Right, ok so you find that a barrier?

P12: Yeah and if there is stuff that I can do at home I would rather do it at home.

R: Yeah, I think what is good about this app is that I think it costs like fifty pound a year so if you think about it uhm gym memberships are now like twenty to thirty a month.

P12: Yeah, the cost of accessercise is relatively cheap and affordable to use.

R: So, that’s like two months’ worth of membership to have full access to the accessercise application so I think the app will help tackle physical activity barriers which could be a useful way especially with the development of technology and you know with apps it could be a useful way to get people encouraged and also motivated to do some sort of physical activity.

P12: Yeah, the app is cheap and a lot more affordable than gym membership.

R: We are not expecting them to do loads but we want them to do some sort of physical activity which is good uhm so these questions are very much accessercise specific questions so another trick question but do you currently use accessercise in your role if so when and how?

P12: Uhm, I have only been using it for the last day just to take part in this study.

R: Ok, so if you were to use the app uhm do you think it is something that you will use on a daily basis?

P12: It will probably be a couple of times a week.

R: Ok, and you mentioned about motivation based on your experience of previous apps uhm do you think the use of the notification bell section could be a useful nudge?

P12: Yeah, I think you need like nudges to remind you to do stuff yeah.

R: But I even feel with notifications and nudges uhm sometimes they are not useful.

P12: Oh, yeah you can easily ignore them but at least it’s there reminding you.

R: Do you think the idea of a streak could be a good thing uhm so after like ten days you see ugh you have done eleven days don’t give up now?

P12: Yeah, streaks could help motivate users to be more active mean like the only other thing I can relate to is when I quit smoking and the smoking sensation app will count the hours that you have quit and then it will tell you in like a years time that you haven’t had anything for like a year so that is really good.

R: Oh, that sounds great!

P12: I think streaks gives you that motivation to keep going yeah.

R: Yeah, when I think about motivation I think you know notifications are good but they are so easily dismissed.

P12: Yeah, yeah streaks are good but some users may ignore it.

R: But I think if you see something that says you have done this for so long uhm you don’t want to break that streak because you feel guilty and a bit worthless so I think if they had a streak I think it would be a good way to get people involved.

P12: Yeah, streaks could be a good incentive to motivate users to exercise.

R: I think about things like New Year resolutions uhm people do it for like a month and then there is no like incentive long term.

P12: Yeah.

R: But I think if something popped up on your screen and it says you haven’t smoked for two years you are then not going to be tempted to go and smoke.

P12: No.

R: Because you have put two years worth of work in uhm you don’t want to ruin your performance so that is quite interesting uhm the next question I have got is what were your thoughts when you first started using the accessercise application so your initial thoughts yesterday when you downloaded it uhm what stood out to you uhm what did you like what didn’t you like?

P12: Uhm, I mean it’s fairly easy to use ugh I really liked the explore function I spent ages on that yesterday looking at different places that are accessible.

R: Yeah, I think the only thing with the explore function is because it’s a new app there’s obviously not too many users.

P12: Yeah, one problem on the app is that not many gyms have been reviewed yet.

R: There’s a lot that haven’t been uhm measured as accessible yet.

P12: Yeah, that’s a problem but it will take time to review all these gyms.

R: But that comes down to the users ugh reviewing that so as you can see there you have got a white and purple logo with accessercise uhm that has been ugh reviewed.

P12: Yep.

R: But it’s the ones with the weights uhm they are accessible gyms but they haven’t been reviewed.

P12: Yeah.

R: So, there’s like four there that haven’t been reviewed yet oh actually this one has.

P12: I mean I always ugh so there’s a usability guide for gyms and like you have to meet a certain threshold before you’re given an accreditation.

R: Right, ok.

P12: So, I go to that website to look uhm but the accessercise app is much easier where you can do it through the explore map function.

R: Do you find that for example uhm the first time you saw that like the different colours of the logos uhm you weren’t sure what was what?

P12: Yeah, the map function is unclear on what some of the features mean.

R: So, do you think maybe the use of a key?

P12: Yeah, a key would be useful.

R: Yeah, because when I first saw it I was like what does this mean?

P12: Yeah, you think automatically these are all accessible gyms.

R: But you get one that it different like this one uhm so whether they could include something like that would be pretty useful.

P12: Yeah, a key function included on the accessercise map would be useful.

R: So, you like the explore function but how about like the options with nutrition uhm the shop uhm did you like those functions so if you go to I think more uhm obviously they are still yet to be developed.

P12: Yeah, the shop, nutrition, podcasts and blogs are all really useful features.

R: But do you like how the app developers have included those functions?

P12: I think the idea of nutrition is really really good uhm to help you build a diet plan.

R: But in terms of going forward would you like there to be education videos uhm would you like there to be meal plans uhm receipes?

P12: I think uhm meal plans and recipes is useful.

R: Ok.

P12: Yep, uhm definitely for like nutrition uhm so you can plan out a weekly uhm menu kind of thing.

R: Ok, but what do you think about like educational videos because I have had a few people say like I don’t know what a protein is uhm I don’t know what a carbohydrate is uhm so maybe there could be some videos that say this is a carbohydrate or even having like the food wheel uhm is it called the food wheel?

P12: Yeah, yeah nutritional educational videos could really help users.

R: Having that on uhm could be a good option to have uhm but also how about podcasts and blogs do you think it would be good to have maybe users explaining their stories?

P12: I think blogs are really useful ugh podcasts it depends on what your taste is in podcasts ugh I listen to comedy podcasts uhm I wouldn’t listen to ugh sport podcasts.

R: Sport podcasts?

P12: No.

R: What do you think the reason is for that?

P12: I just like to switch so like comedy helps me to switch off.

R: Ok.

P12: I enjoy it more.

R: Ok.

P12: So, I think that wouldn’t really interest me but I think it’s good for other people.

R: Do you think the reason for that is because sometimes sport podcasts might be considered a bit dull?

P12: Yeah, so when I listen to these rugby guys they are funny so it’s interesting to listen to.

R: Yeah.

P12: But yeah.

R: Perfect, ok uhm ok that is awesome but in terms of the shop.

P12: Yep.

R: What would you want to be included there?

P12: I would like the shop to be able to link to places where you are able to get the equipment that are shown in the videos.

R: Perfect, ok.

P12: Uhm.

R: How about like discounts if you were to do a certain amount of exercise so if we go back to the streaks uhm so like you have done ten days here is a five percent discount uhm here is a ten percent discount on resistance bands.

P12: Yeah, I think like it’s good to have discounts anyway if you’re a member of something it’s always useful to ugh get discounts on certain shops.

R: Yeah.

P12: So, I get that through like my disability skiing membership uhm you get discounts for certain shops.

R: Ok.

P12: Just because you’re a member so I think rather than a streak thing it’s just good as an incentive to have discounts uhm anyway.

R: Do you think uhm having the accessercise logo clothing uhm and water bottles is a good idea so you feel part of the accessercise community?

P12: Yeah, I mean if they are decent quality yeah.

R: Perfect, ok awesome uhm so do you have the required equipment to use accessercise in it’s full capacity in other words access to a mobile phone to use the application?

P12: Yeah.

R: So, if you were to use the app uhm are you more likely to use it on a tablet or your phone?

P12: I would use a tablet.

R: Perfect, ok.

P12: Yeah.

R: That is great so you have access to a tablet which is good uhm the next question that I have got is based on your experience of using the app since yesterday are there any improvements that you believe could help improve the accessercise application so does anything stand out to you that you thought you know this is good but I think this could be better?

P12: Uhm, gosh.

R: Is it easy to navigate around?

P12: I think it’s fairly easy to navigate around yeah.

R: Do you think the colours are quite good?

P12: The colours on the app is a bit bland.

R: A bit bland?

P12: Yeah, the developers have used some bland colours on the app.

R: So, do you think maybe they should mix like two colours together?

P12: I think the colours they use could be a bit more exciting.

R: Right, ok.

P12: Uhm.

R: So, an area for development for the app is that they could make it a bit more exciting in terms of the colour?

P12: Yeah, and I think when you go into like that uhm this is not easy to follow in terms of the instructions that they give you.

R: So, the instructions for each exercise uhm you find it to have a bit too much information?

P12: I think it needs to be broken up because there’s so much information here.

R: Right, ok.

P12: Because you have like key benefits so you have uhm bullet points but the rest of the exercise should be bullet pointed like it should be step by step.

R: Yeah.

P12: Rather than just one big block of text.

R: Perfect, ok and do you like the idea of having key benefits down there?

P12: Yeah, the key benefits section is pretty useful to have on the app.

R: Yeah.

P12: And I like the photos of where you are actually working uhm so what muscle groups you are working.

R: That’s good to hear and you then have the useful tip at the bottom so that could be like inhale uhm exhale after each exercise.

P12: Yep.

R: Ugh, as well.

P12: Yeah.

R: So, do you think maybe the colour could be improved there?

P12: I think yeah it looks just a bit like ugh vanilla yeah.

R: Do you like the option that you can favourite as well?

P12: Yeah, the option to favourite exercises will definitely come in handy.

R: Perfect, ok and what were your thoughts about the calendar function do you think that is something that you might use?

P12: I think the calendar is a really useful function to have so you can plan things in properly.

R: Yeah.

P12: But then you want the notifications come off that so.

R: So, you would like to be notified when you’re supposed to do an exercise?

P12: Yeah, notifying users on when to exercise and any updates is good to have.

R: Perfect, ok that’s quite interesting but do you think the calendar there is quite laid out nicely?

P12: Yeah, I think the calendar is a really good function uhm if the calendar can sync with your personal calendar that would be really useful.

R: I have had a few people say that yeah which is quite interesting.

P12: Yes, I think syncing the calander from accessercise with others can be good.

R: Uhm, so you would like everything to be synced into one calander uhm so outlook is linked into accessercise?

P12: Yeah, syncing the accessercise calendar with my own calendar would help.

R: What are your thoughts about so the social section uhm so obviously you can post on there and people can see your workouts and so on uhm would you like that data to be exported out of the app onto things like Facebook and Twitter so people can see your progress?

P12: I think uhm for some people that’s a good option but I wouldn’t do that.

R: Right, ok.

P12: I don’t really use social media a lot so.

R: Yeah, me too.

P12: I think it’s a useful function to include on it yeah but personally it’s not something I would be using.

R: Perfect, ok and do you like the option how you can follow and unfollow people?

P12: Yeah, it’s a great way to build a little community.

R: But in terms of the social section do you think they are spot on with that do you think what they have done is not too much?

P12: No, I think it’s the way forward isn’t it uhm to have this sort of social side of it as well uhm but yeah it seems fine.

R: Perfect.

P12: I think as it develops you know it will get better.

R: So, as you can see in the top right there’s find groups.

P12: Yep.

R: What would you like specifically to be added there would you like there to be other opportunities for things or does it always have to be around sport and exercise uhm could it be other things?

P12: I think if it’s to do with the app it’s pretty useful just to keep it within the exercises and the sport side of things.

R: Yeah how about uhm so obviously you know on the explore section it’s all about gyms.

P12: Yeah.

R: One of the issues is a lack of advertisement and opportunities to undertake regular physical activity so for example wheelchair basketball you know it may not be well advertised but could they provide opportunities for those clubs on the explore section?

P12: I think they could probably link a hell of a lot into the explore function.

R: Right, ok.

P12: So, like the different sports that are available.

R: Right, ok.

P12: Within a county you know from accessible swimming sessions through to basketball, rugby.

R: So, they could then link in like wheelpower?

P12: Yeah, that would be really useful because like some swimming centres will only have a certain slot for disabled swimming uhm so you will want to know when that slot is available so uhm I think that would be really useful function to have.

R: So, at the moment it’s obviously all focused on gyms but you would like to see a bit more done there?

P12: Yeah, if you want to get people exercising you have to give them options.

R: Right, ok.

P12: Like to me the gym is the most boring form of exercise so I prefer to go and play a sport.

R: I wonder whether they could split the explore function up into two sections so they have gym section and sport section.

P12: Yeah, sporting opportunities as well as exercises on the explore function.

R: So, you can see like ok in Loughborough there is accessible wheelchair tennis uhm wheelchair rugby wheelchair basketball and they can easily find that uhm find the information and then contact the club.

P12: I think it would be useful to have like different keys so if it’s a wheelchair tennis club uhm it has a tennis symbol.

R: Like a racquet?

P12: Yeah, a racquet uhm if it’s a basketball club it has a basketball symbol.

R: Yeah.

P12: So, you can go on the one map but see what the different sports are.

R: Yeah, and you can then zoom in uhm they could also do a listed option so when you put your post code in it comes up with a list of the clubs.

P12: Yeah.

R: So, these are things that could be linked into wheelpower uhm dwarfism UK and so on.

P12: Yeah.

R: It helps to ensure that everything is linked into one page I think would be really good to have uhm so obviously you have got like the list there.

P12: Yeah, uhm that would be really good to be able to put your postcode in and have a list and the different symbols for the different sports come up.

R: Yeah, because then you can be like ok today I want to do this.

P12: Yes.

R: But also do you think they could have uhm a distance ugh like an approximation thing so like maximum fifty miles?

P12: Yes, you want to be able to limit it to a certain distance yeah.

R: You could put that up and it comes up with nearby but it might come up with hundreds of options and you are like oh I really like this club but it’s in Birmingham oh it’s in Durham uhm so whether they could have that could be really good.

P12: Yeah, you should be able to choose how far you want to travel for something.

R: Yeah, and how about uhm so you mentioned about the key section so having keys where you can see what the orange means uhm what the white means.

P12: Yeah, having a key for what all these features on the map is really important.

R: That would be pretty good ugh but do you think anything else could be added to that or do you think there is like enough there?

P12: I think you could have numbers so graded on the accessibility.

R: Ok.

P12: So, if it’s not got the accessercise logo which you know is an accessible gym it’s graded.

R: Right, ok.

P12: So, you know from like reviews what grading it is.

R: Right, ok.

P12: If I go to that and I give that a one and then someone else gives it a one then you will be able to see that it’s got an average of a one rating kind of thing.

R: So, just going onto this so you have pressed onto this now so you can see the stars uhm so it hasn’t been rated there.

P12: Yeah.

R: Do you like what you’re seeing on that page uhm do you think there is some relevant information there?

P12: I think it could do with a lot more information in terms of whether it’s wheelchair accessible uhm how high is the equipment ugh what sort of equipment have they got uhm what is the layout of the gym like is it good for visual impairments.

R: Right, ok so you would recommend like an easy to see demographic uhm I don’t know uhm like some pictures there uhm with tick tick tick?

P12: Yeah, having a tick list of all the accessible resources for each gym.

R: Uhm, because you want to make it easy so people can read it and be like ok I understand this.

P12: Yeah, I mean another thing you can do is on the explore section is to have a filter so you can put in wheelchair user.

R: Ok.

P12: You know whatever your disability is and it will only show up things that are suitable for you.

R: Yeah, that makes sense uhm so if you were to press on the link again.

P12: Ok.

R: Yeah, that one there uhm so that’s four stars uhm do you think they could possibly add any images there?

P12: Yeah, so the reviews don’t tell you why it’s four stars so you want to know why.

R: Why ok.

P12: Yeah, so what makes it accessible.

R: Perfect, ok so going forward what would you like to understand?

P12: I think specifically it would be good to know about access into the building uhm does it have a lift uhm is it wheelchair accessible uhm what is the layout like in terms of can you actual move around the gym and have space to work.

R: Ok.

P12: A lot of weight stations are so close together so as a wheelchair user you constantly feel like you’re in the way.

R: Right, ok.

P12: So, does it have space for you to actually work?

R: Right, ok.

P12: I know from people with visual impairments that gyms are cluttered so they want to know whether there’s a clear walkway around.

R: Uhm, so obviously at the moment it has got the opening hours uhm it has the phone number.

P12: Yeah, the information about the opening hours and contact details is good.

R: Would you like to see less busier hours uhm would you like to see whether staff is trained in adapted physical activity?

P12: It would be useful to know is there a staff member that is trained to deal with someone in a wheelchair.

R: Ok, uhm so you have got your checklist with accessible equipment uhm accessible this and that but you would like to see accessible staff?

P12: Yeah, so like trained staff.

R: I have seen before on gym websites you can actually see when the most busiest hours are.

P12: Yeah, like it would definitely be interesting if they could have like a quiet session.

R: Ok.

P12: And like advertise that on here.

R: Ok, and how about like a rotation of the gym so if you were to look at the gym you obviously wouldn’t know what it looks like so do you think having some videos?

P12: Yeah, like a 360 rotation of the gym would be really good yeah.

R: Yeah, ok uhm but also in terms of parking do you think anything could be added?

P12: Yeah, you want to know uhm if it has got disabled parking bays up front.

R: Ok.

P12: Having information regarding disabled parking is definitely useful.

R: Uhm, accessible toilets?

P12: Yeah, so has it got disabled parking uhm has it got accessible toilet uhm has it got a shower uhm that kind of information would be really really useful.

R: How about an actual link to the gyms website?

P12: Yeah, I don’t understand why it doesn’t have a link to the gym already.

R: Yeah, because some people said that all the information that was recommended could just be on the website.

P12: Yeah, a link to the gyms website will be so convenient and quicker.

R: So, whether they link that through the website.

P12: Yeah, I don’t understand why it doesn’t have a link that you can just click onto that gym’s website.

R: Yeah.

P12: That’s interesting yeah.

R: How about reviews so obviously that’s four stars.

P12: Yep.

R: Let’s say six people reviewed that.

P12: Yeah.

R: You don’t really know what they have said.

P12: Yeah, you can’t see the reviews so you don’t know why.

R: So, do you think maybe having the option to see the reviews would be good?

P12: Yeah, being able to see a detailed review would be helpful.

R: Ok, uhm but is there anything else there ugh do you like ugh is there too much information there?

P12: No, I think it needs more information yeah.

R: I think at the moment it’s got some useful information there uhm it’s four stars which is great but for example I may have an amputee uhm you have got dwarfism how can I tell whether that is accessible for me uhm because the person that has reviewed that uhm so you may get five people that reviewed that with a spinal cord injury but is it accessible for someone with an amputee?

P12: Yeah.

R: So, it would be interesting to know this is this uhm this is this uhm it’s accessible with four stars for equipment uhm three stars for the venue uhm so if I read that I’m like oh these are the reasons why they have given these ratings.

P12: Yeah, if you click on it and it’s got reviewed by somebody in a wheelchair you go ok what is their experience like and that would make it a lot more informative then just a star system.

R: Perfect, ok that’s awesome so if we were to move onto the next question so do you use any other similar applications outside of accessercise if so what is better or worse than accessercise?

P12: Uhm.

R: So, you mentioned about ugh the couch to five k.

P12: Couch to five k I used uhm that was a really useful one for telling you when you need to do it uhm so building it up slowly uhm you can have a choice of people talking you through it so that was a really useful function.

R: Right, ok that’s quite interesting so you mentioned about this app that it’s lacking the logistics ugh the statistics and stuff like that so would you like to have that section on there?

P12: Yeah, I mean it was always good on couch to five k to see how fast you have done it so you are basically trying to beat your time every time.

R: Yeah.

P12: So, it would be interesting like if you could have like ok today I did ten pull ups and you go right next time I’m going to do eleven and that kind of helps you to see if you’re progressing properly.

R: Yeah, that makes sense perfect ok so we are now going to move onto the main part of this study and ask you to participate in a think aloud protocol whilst using the accessercise application the instructions for a think aloud protocol are straight forward I would like you to say out loud whatever comes into your mind there are no right or wrong answers we will just run through a quick practice to get you used to the protocol so could I ask you to say out loud everything that comes to your mind when you think about physical activity?

P12: When I think about physical activity uhm fun but tiring.

R: Tiring ok how about painful?

P12: Uhm, usually painful afterwards.

R: Would you say overwhelming maybe?

P12: Not overwhelming uhm I enjoy it in the moment but then I suffer when I get home.

R: Right, ok but like a few days after exercise do you feel the benefits of doing it uhm do you feel more happy and have a better mood?

P12: Yeah, I mean like definitely for me playing sport makes me happy uhm the chance to socialise with people makes you feel much better.

R: Perfect, ok so what we are going to do now like we have done before we are going to go through each section ugh you’re just going to talk out loud what you think about the app and so on.

P12: Yeah.

R: The first question I have got is uhm what are your first impressions so you said you liked the videos uhm you like the explore uhm just to recap what other things did you say you liked?

P12: Uhm, I think the videos are really good and a useful way to show you how to do something because most of the time you don’t know how to do the exercise.

R: But do you like how in the videos it has got someone with the condition?

P12: Yeah, I think it’s really good that the app is individualised to certain conditions.

R: How about the voice overs do you like the use of the voice overs and how some videos have it included but some don’t?

P12: I think a voice over is a really useful tool so I think all the videos should include that uhm to talk you through how to do the exercise.

R: Yeah, do you like how it’s got the written description on each video?

P12: Yeah, written description for each video is great especially if you’re deaf.

R: So, it’s got like you know do this uhm so if you do have hearing difficulties you can see this is the video uhm it’s also got a description of that.

P12: Yeah, written descriptions for each video is a good function to have.

R: Uhm, the social section you liked didn’t you?

P12: The social section I think is really really good so it’s not something that I would personally use but I think in today’s age it’s something that is needed and for some people this is the way of socialising outside of the house.

R: Ok.

P12: So it’s useful to be able to build that group.

R: Yeah.

P12: And you can start building that motivation to exercise.

R: Yeah, that makes sense. Perfect, ok so I would like you now to use the application as you would normally use it please continue to speak your thoughts as you continue through the app please show me all the features you use and please feel free to describe your actions, thoughts and feelings as you do so uhm so have a look at the instructions that accessercise have provided uhm talk me through how you use these when navigating through the application.

P12: Ok.

R: So, if we start with the social section so if you were to go through it as if you were normally using it so obviously it has got your uhm profile.

P12: Yep.

R: Do you think you would add a photo?

P12: Uhm.

R: Do you think it is something you would add do you like the option of being able to add a photo there?

P12: I think adding a photo is a good idea.

R: Perfect, ok and the achievements uhm what would you like to see from the achievements section?

P12: I think you want to see like medals of you going through different steps.

R: So, like progression?

P12: Yeah, progression yeah.

R: So, sort of like Duolingo?

P12: Yeah.

R: So, you get points depending on how much you have completed?

P12: Yeah, I mean making an app a game makes it more useable so if you are building your points as you are going you are probably going to want to use it more it gives you that motivation to do it.

R: Do you think so personal achievements are great do you think if we start adding competition with other people it becomes a bit too much?

P12: I think you can have that option of competing against other people so if you want to go in a league with somebody but I don’t think that should be something that you are put in it should be something that is an option for you to do uhm so I have got these five friends and we are going to do it together and then you are seeing how you are beating those people.

R: Ok.

P12: Yeah.

R: That makes sense so you would like daily tasks uhm challenges?

P12: Yeah, I like the idea of having daily challenges on the app it motivates you.

R: And then you are provided a discount in the shop based on how much you have done?

P12: Yeah, so if you achieve your daily goal you then get a discount in the shop.

R: I think the only downfall with competitions is that if you have leader boards and stuff someone might have a really bad day which takes them out of the sport for two weeks and they then fall down the leader board and people think oh they are not that good but actually it’s their condition not their ability.

P12: Yeah, so I think that leader board is among you and friends they know you but if it’s you and a thousand people and you are down at the bottom it’s hard to get that motivation to keep going.

R: So, do you think the community groups which you can follow on the social hub uhm having a bit of competition there would be good uhm but I guess you don’t really know them?

P12: No, but you build a social circle don’t you on that app.

R: Ok.

P12: And then it’s up to you whether you decide to do that bit or not.

R: So, having the option would be a good idea?

P12: Yeah, a bit of competition on the app can be a good feature to have.

R: Ok, so if we were to go back uhm down the bottom do you think it’s good that they have provided the option to propose about yourself uhm so your training uhm so that could be build strength uhm is there anything else that you think could be added there or do you think that’s enough?

P12: No, I think in terms of you want it to relate to the app so just being able to say this is why I’m using the app is quite a good idea but I don’t want to go onto a fitness app and have someone’s profile where it’s their life story.

R: Yeah, yeah.

P12: I just want to know why they are using the app.

R: Do you like how you can see all the groups that you’re a part off?

P12: Yeah, seeing a list of groups you’re a part of is useful.

R: So, you can see at the bottom I’m new to this so you can see that I’m a part of this guy uhm you can also leave and you know join again and so on.

P12: Yeah.

R: So, that’s good so uhm obviously it has got your name uhm it has got your email address so if you wanted to put your email address and phone number there people can then contact you and be like oh hey I saw you doing this workout is it ok if you teach me how to do this and so on uhm so it’s trying to build that connection which is pretty good so if you were to go back uhm so if you were to press on the video how about this uhm do you like the description uhm do you like the videos uhm you mentioned about the favourites uhm is the colour ok?

P12: Yeah, I mean yeah I think it’s a bit of a ugh bland app at the moment.

R: Right, ok.

P12: It could do with being a bit more jazzed up.

R: Ok.

P12: I think the videos are a really good idea uhm I like the fact that uhm some of them but not all of them have a voice over they also have it written down in text so if you can’t hear it you can see what you need to be doing.

R: Yeah.

P12: The instructions for each exercise need to be numbered or bullet pointed uhm or ordered in stages or something because it’s just one block of text uhm it needs to be like right you start doing this and then you do this and then you do that uhm and that helps you do it correctly.

R: Right, ok. So, you obviously like the photos of ugh the muscle components being worked?

P12: Yeah, the muscle component section is good uhm being able to see what areas you are working uhm it would be kind of useful if you went like right I want to work this this and this area and as you are doing it uhm it ticks off that you have done this one today.

R: Right, ok.

P12: So, you know it kind of tells you that you have completed it through a checklist or something.

R: Ok, that makes sense perfect and is there anything else you think could be added there uhm how about like a progression uhm you know this exercise is great how about at the bottom to make this harder do this?

P12: Yeah.

R: It’s got a useful tip which is good uhm but how about a progression box?

P12: Yeah, I think like in terms of you start with this weight and you up the weight by this much to progress through or you start with this stage it becomes harder uhm that would be quite useful.

R: Ok, because you can put it like challenge yourself.

P12: Yeah, yeah you need a more challenging version of each exercise.

R: So, people can be like oh today I want a challenge I want to do this this and that uhm which is pretty good uhm awesome so if we were to go back uhm and we were to go down to exercises and go to the filter option do you like what you see there do you like the logos of each one do you like how it’s sort of clustered into home ugh gym and outside?

P12: Yeah, that’s really helpful having gym, home and outside uhm choosing goal the only thing I would want to fit into choose goal is some kind of social option?

R: A social option?

P12: Yeah, like a social element.

R: So, like dancing or something like that?

P12: Well, yeah some people want to get involved in sport to socialise uhm so it’s their opportunity to socialise so it would be good to have that as an option as well outside of you know.

R: Yeah, some people say you know sport should be fun like dancing uhm you know that sort of stuff so they should be given the option to have that.

P12: Yeah, all of these options here are related to gym uhm but you want something that adds that social element or fun side to it or that chance to participate in a team sports kind of thing.

R: Yeah, they could add a bit of sport there and not always focus on the gym.

P12: Yeah.

R: Do you like the logos that they have used next to each other so you have got like muscle uhm you have got like you know a frame for mobility?

P12: Yeah, that’s all pretty relatable.

R: Perfect, ok and you can obviously select your uhm muscle components that you want to work uhm but can you see here how it’s got like four four two here.

P12: Yeah, I really like the muscle component photos on this app.

R: Do you think the layout and structure could be better uhm do you think maybe they could do like three three three or something like that?

P12: I think overall yeah it needs a bit of formatting.

R: Consistency?

P12: Yeah, I think the accessercise app could be more aesthetically pleasing.

R: And do you think they should put these options in alphabetical order?

P12: Personally, I would have it in alphabetical order but that’s just the way that I would like to do things.

R: Right, ok.

P12: And to be honest it’s not very logical anyway.

R: Yeah.

P12: Like you would want your hand and wrist next to your arms and maybe your legs rather than core, lats and chest than hand and wrist.

R: Yeah, it’s not very clear is it.

P12: It’s not very logical.

R: And you have obviously got the difficulties?

P12: Yeah, the difficulty option is really good.

R: Yeah, but some people say what is the difference between easy and light could that not just be easy?

P12: Yeah, it depends uhm I know what these mean because I have read research around them so I understand what it means uhm it’s useful but yeah it’s not very understandable for someone that doesn’t understand that.

R: Yeah, so do you think maybe just keep it basic and have easy uhm moderate and then difficult?

P12: Yeah, less options for difficulty it’s better to have easy, moderate or hard.

R: So, three options uhm so instead of having five uhm you have easy moderate and difficult.

P12: Yeah, easy, moderate and difficult would make sense.

R: Yeah, uhm how about the option to have a carer uhm what would you want specifically to be used there?

P12: I wouldn’t go to a gym with a carer uhm I would want to know if there is an option of someone helping me get equipment down.

R: If you were there?

P12: Yeah, if I was there so does that gym have that uhm that help.

R: Ok.

P12: That would be useful.

R: If you were to press home exercises for me for example.

P12: Yeah.

R: Can you see how these are a bit inconsistent so how they have got two two three four four five?

P12: Yeah.

R: And maybe they could put these in alphabetical order because if I’m looking for a certain type of equipment uhm if it’s alphabetical order I can easily locate it.

P12: Yeah, having options in alphabetical order you can find it quicker.

R: Yeah, instead of looking up and down.

P12: Yeah, keeping the options in alphabetical order will make it easier for users.

R: So, that needs to be laid out a bit better I think.

P12: Yeah, the layout of some features on the accessercise app could be improved.

R: Uhm, but yeah I think there is a lot they can do.

P12: I think they could just have columns of two.

R: Yeah, but then do you think they have done that to reduce how much scrolling you are doing?

P12: Uhm, possibly but I don’t think it makes it any easier.

R: No.

P12: Like if it was in alphabetical order you go right I know that I need a medicine ball uhm that’s an m.

R: How about uhm so like you discussed earlier how about if you select based on your location uhm your goal it can then narrow equipment down?

P12: Yeah, some options could be removed if they are not related to your goal.

R: So, if I chose home, flexibility uhm I need a yoga mat but I don’t need a medicine ball.

P12: Yep, so remove any information that does not relate to my goal or workout.

R: So, whether that information could be reduced uhm do you agree with that?

P12: Yeah, but I think the only issue with that is that you are adding filters to filters uhm but I suppose yeah that would be useful.

R: You’re obviously not going to have a treadmill in the park are you?

P12: No.

R: Because obviously by doing that it will make it easier for people.

P12: Yeah, but it would have to have the formulae in the background without you going like gym and then you have to go right apply filters uhm and then it does it uhm so filters back kind of thing.

R: That makes sense so you like the favourite option don’t you?

P12: Yeah, I think the favourites is good.

R: Perfect, and you can obviously search.

P12: If you favourite something is it going to show up in the list of your favourite exercises?

R: Yeah, so you have got workouts and then you can add your favourite exercsies to your workout.

P12: Yep.

R: So, we have done explore uhm so we will go to the last one which is more.

P12: Yep.

R: So, you can see shop, nutrition uhm blog podcasts uhm you have mentioned about this uhm incentives and so on.

P12: Yeah.

R: If you were to go to account settings and you go to impairment filter do you like this option how you can select uhm so obviously at the moment it hasn’t got every disability on there.

P12: Yeah, the app has done a great job ensuring it’s impairment specific.

R: But they are focusing on including more.

P12: Yeah.

R: Uhm, but obviously you can select your impairment and you can save it and then it shapes the app around that selection.

P12: Uhm, so what happens if you have two disabilities uhm does it save both of them?

R: Yeah, it should do uhm that would be pretty interesting.

P12: Yeah, single uhm spinal cord injury uhm as paraplegic yeah because I have got more than one disability uhm it’s good that you have the option to select more than one because most of the time you have to pick one or the other.

R: Yeah, that makes sense. Uhm, do you like how it’s filtered based on that?

P12: Yeah.

R: Ok.

P12: I think selecting your impairment on the app helps you to see yourself in the app uhm so if you select dwarfism and all of the videos are people with dwarfism you can see yourself in the app then it makes you feel a lot more motivated and likely to use it.

R: So, did you feel more motivated when you see someone similar to you doing the exercises?

P12: Yeah, being able to see yourself in something makes you feel more motivated to get on with it.

R: Ok, that’s good so if we go back uhm we have here exercise habits.

P12: Yeah.

R: I don’t really know what that does to be honest even if you select you are very active uhm I don’t know if that shapes anything uhm would you like anything to be added to that or explained in a bit more detail?

P12: I would like to know why you need to select that uhm what is that adding?

R: Yeah.

P12: And the option when you can select someone to assist you when exercising uhm how does that help you by clicking that.

R: Yeah, makes sense.

P12: Like you want it to actually lead to something.

R: That makes sense so if we were to go back and obviously you have got measurement units do you like how it’s got that?

P12: Yeah, I think it’s a really good idea to be able to monitor your weight if you’re doing stuff.

R: Yeah, the only downfall is that I don’t think you can actually add weight into the app so I don’t really know why they have got that because you can’t enter your height or weight.

P12: Ok.

R: So, whether that’s something they are looking to include.

P12: I think it would be really useful to monitor your weight and maybe even have like blood pressure in there.

R: Ok.

P12: So, it becomes like a health app on top of the exercise.

R: Right, ok that makes sense uhm so let’s go back uhm you have the option to change that do you like that it’s quite simple and easy to do.

P12: Yeah, that’s good uhm it’s the same as any app or website.

R: And then you have got like privacy settings uhm do you like how you can select whether it’s private uhm you can put your phone number on and so on?

P12: Yeah, I wouldn’t feel comfortable having all my information on something so it’s good that you can make it private or say you don’t want certain things included on your page.

R: Yeah, I agree.

P12: Yeah, selecting what you have on your profile is important.

R: Sometimes with personal information it might be a bit too much uhm so people you don’t know might start trying to use your information and so on.

P12: Yeah, accessercise has done a great job at protecting your privacy.

R: Perfect, ok so we will now go onto the overall thoughts uhm so the first question I have got is what are the potential positives if there are any of using accessercise for improving physical activity levels?

P12: Like I said I think it’s a really good idea that you can see yourself in the videos uhm to me that is a really unique part of this accessercise app.

R: Do you like how the app is focused on the impairment?

P12: Yeah, it individualises it for you but it’s not like right you are going to do some weights and we are going to show you someone that is able bodied.

R: Right.

P12: Doing it and you have to kind of workout how you are going to adapt it.

R: Right.

P12: You can see yourself doing it from the off kind of thing.

R: Perfect and is there any other potential benefits uhm so like the community group?

P12: The community group I think would be uhm amazing for some people uhm for me it’s not a big draw but I do think if you are stuck at home by yourself it is a way of socialising with people.

R: Yeah.

P12: So it would be useful to see how the app develops like in terms of socialisation.

R: Ok, and the explore section you like that don’t you?

P12: The explore section I think is very good but I think it could incorporate a lot more uhm I would like it to incorporate sports so it’s not always about gyms and I would like to know why some things are accessible so I think that is a big area that needs to be improved upon uhm it’s about understanding why that gym is accessible uhm what does it mean.

R: Right, ok. Uhm, the second question I have got is what are the potential negatives if there are any of using accessercise for improving physical activity levels?

P12: Uhm, I think the only negative is the same with any app uhm is that you can ignore it.

R: Right, ok ignore it.

P12: It is a really good idea and it will probably give you the motivation for the first bit but with apps they can send you notifications but you can easily end up silencing notifications and just ignore it so.

R: Right, ok.

P12: Yeah.

R: Perfect, ok and the last question I have got is how do you think the accessercise application could be improved if anything for it’s use in improving physical activity levels uhm so if you had to name three key areas that the app really needs to focus on what would those three be?

P12: I think the layout so in terms of adding bullet points or numbered guides for each exercise rather than just a block of text uhm and I then think providing more information on the accessibility rating but also information on whether that gym is wheelchair accessible or is it just accessible for somebody with a prosthetic leg.

R: Ok.

P12: So, you want to know as a wheelchair user am I going to be able to go and use that gym.

R: Ok.

P12: That kind of thing uhm you want that sort of detail uhm and maybe some quiet hours included if they do quiet hours that kind of thing.

R: Perfect, ok and how about designing your own workouts uhm do you like the locations uhm the selection of equipment?

P12: I think.

R: So, when you filter.

P12: Yeah, like in terms of it doesn’t have things in like alphabetical order uhm so it just means that you’re scrolling through trying to find things a bit more uhm it would be interesting if it was in alphabetical order because that would make it a lot simple to find what you need.

R: Which is what we like uhm we like simplisticy.

P12: Yeah, you want to make an app as simple as possible to use uhm at the moment the layout is a bit dull and it involves a lot of scrolling.

R: Haha, great ok so the final question I have is do you have anything else that you would like to add or discuss before we finish the interview process?

P12: Uhm, no.

R: Perfect, well thank you so much for participating I appreciate your involvement in our work.

P12: You are welcome.

R: Thank you.
