# Supplementary material for: Assessing the usability of Accessercise to increase physical activity in adults with physical disabilities: A qualitative think-aloud study
Source: PLoS One. 2025 Apr 1;20(4):e0321109. doi: 10.1371/journal.pone.0321109 (PMC11960980; doi:10.1371/journal.pone.0321109)
Supplement: S3 Table — (DOCX) [file pone.0321109.s003.docx]

**SUPPLEMENTARY MATERIALS**

Contents

[**Table S3.** 2](#_Toc192049360)

[Content analysis map 2](#_Toc192049361)

# **Table S3.**

## Content analysis map

**Raw data** **Higher order theme** **Second order theme**

“I think blogs are a great function on Accessercise.” (P08)

“Nutrition, blogs, shop and podcast are all amazing features on the app.” (P09)

“The blog as well as the podcasts and nutrition if they had those options come out soon that would be amazing.” (P01)

“I never look really at blogs and podcasts I’m not really into them I’m not that interested.” (P03)

“Blogs I wouldn’t be interested [in] podcasts I wouldn’t necessarily watch.” (P05)

“I like the fact that you can make a group especially using that make friends function.” (P01)

“The group option on the app is really helpful and the social side to it and seeing what other people with impairments are doing.” (P07)

**
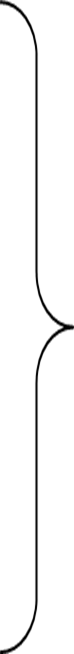
**

Entertainment

**
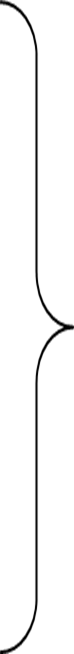
**

“I really like that you can choose where you complete your workouts I don’t think many apps have that option at all.” (P01)

“I think that’s a good feature of the app customising which muscle components to work especially if you’re a beginner.” (P07)

“I like the fact that you can choose a goal and the goal isn’t just like increase muscle [or] lose weight.” (P01)

“The ability to customise and choose adapted equipment for your workouts is an amazing idea.” (P04)

“The filter option on the workouts is a really good feature.” (P09)

“I really like the calendar setting on the app [and] adding your workouts.” (P02)

Customisation

**
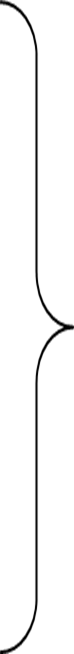
**

“I think Accessercise is missing logistics data I want to review my progress.” (P07)

“Accessercise is limited at inputting data about you.” (P08)

“I think the thing with the app is I guess there’s no feedback there.” (P10)

“Monitoring progression through graphs and statistics would be good.” (P09)

“I noticed here that you can invite friends which is really useful.” (P02)

“I like the fact that you can share your goals or ideas it’s bit like, you know, like the Strava fitness app.” (P01)

“Notifying users on when to exercise and any updates is good to have.” (P12)

“Notifications or nudges would really help me undertake more exercise.” (P09)

Interactivity

-0-0-

**
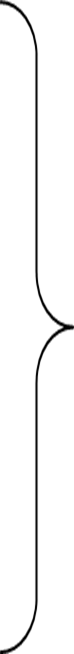
**

Target group

“The terminology they have used for each goal is good.” (P10)

“Getting toned as opposed to weight loss is a better terminology.” (P06)

“I like the fact that the app is adapted for people with disabilities if that makes sense?” (P01)

“A strength of Accessercise is that it focuses on the disabilities.” (P09)

“I like how you can find local gyms with the explore section, that’s a really useful feature.” (P03)

“I really liked the explore function. I spent ages on that yesterday looking at different places that are accessible.” (P12)

“Yeah, the map function is arguably Accessercise’s best feature.” (P07)

“I like that it has the assistant and carer option as well, that is good.” (P06)

“I like the fact that it says require assistance or a carer. I think that would be a great option to have.” (P01)

**Engagement**

Entertainment

Customisation

Interactivity

Target group

**
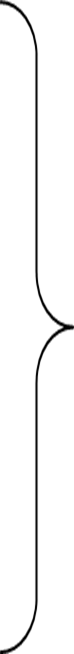
**

“I guess obviously just the stuff that says coming soon would be nice to have.” (P01)

“I think it’s pretty good. I think it just needs to have the rest of the stuff come out and then it would be good.” (P01)

“Obviously it’s still coming soon which is a weakness of the app.” (P09)

“I just used the filter option and only two things have come up.” (P03)

“Finding new groups, it’s not working.” (P03)

“I have put in Eastbourne but it has listed gyms within Loughborough.” (P07)

“When I complete the exercise habits it doesn’t do anything.” (P09)

**
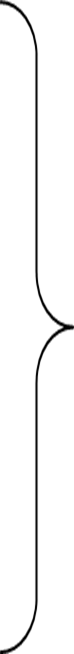
**

Performance

“Just having those four options at the bottom makes it a bit easier than having more information.” (P09)

“It’s all clear and easy to pick this app up and use it even if you’re a beginner.” (P11)

“It is really easy to learn how to use this app because it’s made simple.” (P03)

“I do like that this section has clear instructions on how to do the exercises which is very helpful.” (P11)

“I like how Accessercise provides some welcome instructions when you first use the app which is helpful to use the app more smoothly.” (P04)

“I also really like the videos because they show you how to actually do the exercise because some people don’t know how to do it properly.” (P02)

“I like the video function, so you can actually see how movements are done making exercising easier as you can copy the techniques especially if you’re new to exercising.” (P07)

Ease of use

**Functionality**

Performance

Ease of use

Navigation

**
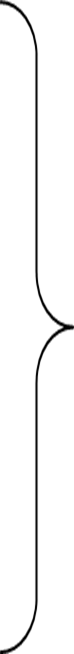
**

“It just seems to be really easy to get around which is a really key thing really.” (P03)

“The app is really easy to navigate through.” (P03)

“It’s very easy to navigate and it’s very easy to search for the exercises that I want which is really good.” (P03)

“To me, it’s easy to use and it seems pretty simple.” (P04)

“I really like the Accessercise app I think it will be really easy to follow when you are starting the session.” (P07)

“It’s quite easy to navigate mainly with the four tabs down the bottom of the screen.” (P07)

“The fact that anyone can go onto this and it pretty much follows the same sort of rules that an awful a lot of apps use so you could navigate around.” (P08)

“I think it’s fairly easy to navigate around yeah.” (P12)

**
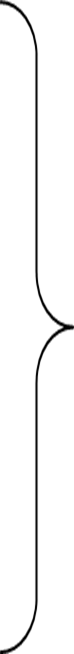
**

“The layout of the app is good they have done a good job.” (P03)

“I like that it’s alright, the layout is good.” (P03)

“It seems all good to me the structure of everything.” (P03)

“The layout for the list of equipment needs developing as it’s messy.” (P07)

“It’s alright but it’s not all alphabetical if it was alphabetical that would be good.” (P07)

“I think the layout so in terms of adding bullet points or numbered guides for each exercise rather than just a block of text.” (P12)

“I think the layout of the app is easy to work your away around it, the colour scheme is fine for me.” (P10)

“I do like the layout and it very much feels like Instagram.” (P10)

“You want to make an app as simple as possible to use. At the moment the layout is a bit dull and it involves a lot of scrolling.” (P12)

Layout

Navigation

**Aesthetics**

Layout

Visual appeal

**
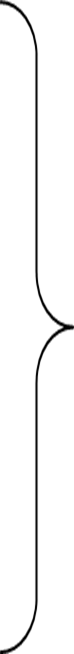
**

“It comes across pleasant straight away and compared to the other fitness apps there are only four options at the bottom and it’s straight forward and clean.” (P04)

“Accessercise has good colours compared to other fitness apps I have seen.” (P07)

“The choice of colour here for the key benefit box is good.” (P11)

“The colour scheme is fine for me.” (P10)

“The app is a bit too white [laughs].” (P01)

“Some of the things on Accessercise are quite bland and boring.” (P07)

“I think it’s a bit of a bland app at the moment.” (P12)

“The developers have used some bland colours on the app.” (P12)

“An area for development for the Accessercise app is having the option to change colours on the app so that it can help someone that has a visual impairment using the app.” (P02)

**
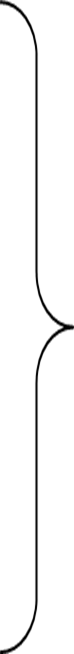
**

Quality of information

“The information about the opening hours and contact details is good.” (P12)

“A phone number and opening hours are good but it’s missing information [e.g., reviews on accessibility, cleanliness and tidiness].” (P10)

“The phone number and opening hours is good to have. “(P07)

“The exercise description is good but could be numbered or bullet pointed.” (P09)

“The fact there’s a description of the exercise there and it’s quite detailed is good.” (P08)

“The description of the exercises is quite good because you can also see what parts of the muscles the exercises are targeting.” (P11)

“I like the fact that the app tells me the key benefits of the exercise.” (P01)

“I think it’s good that they have got the breathing tip.” (P11)

Visual appeal

**
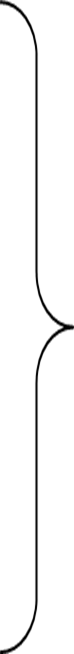
**

“Again, there is not too much to choose from.” (P03)

“It’s alright yeah not too much information.” (P03)

“It’s good, a nice balance of information it’s not overwhelming.” (P03)

“The fact the Accessercise app has basic information and the pages are short that’s good.” (P08)

“You just have to sit and read it [laughs] so the blocked text is a weakness.” (P09)

“There is too much information per exercise with the big block of text.” (P10)

“I think it needs to be broken up because there’s so much information here.” (P12)

“On that filter section just like removing the muscle group section if it’s not like you’re doing flexibility or stretching or something like that.” (P01)

**Information**

Quality of information

Quantity of information

Visual information

Quantity of information

“The images they have used on the app is good.” (P04)

“I agree, the little logo they have used next to each goal is creative and helpful.” (P09)

“Yes, [Accessercise] having logos for exercises is creative and visually appealing.” (P09)

“I like the pictures of the muscle components being worked.” (P03)

“The muscle component section is good being able to see what areas you are working.” (P12)

“The Accessercise app has good videos.” (P02)

“Written description for each video is great especially if you’re deaf.” (P12)

“Audio descriptions for each video is great.” (P07)

“That’s good having voice overs in the videos.” (P03)

“Voice overs are limited on Accessercise they should have more.” (P07)

**
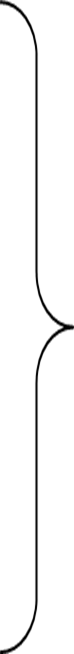
**

Visual information
